# A qualitative evaluation of a multi-modal cancer prehabilitation programme for colorectal, head and neck and lung cancers patients

**DOI:** 10.1371/journal.pone.0277589

**Published:** 2023-10-03

**Authors:** Sharon Linsey Bingham, Sarah Small, Cherith Jane Semple

**Affiliations:** 1 School of Nursing, Institute of Nursing and Health Research, Ulster University, Belfast, Northern Ireland, United Kingdom; 2 Cancer Services, South Eastern Health and Social Care Trust, Belfast, Northern Ireland, United Kingdom; The University of Manchester Division of Psychology and Mental Health, UNITED KINGDOM

## Abstract

**Background:**

Growing evidence indicates patients’ survivorship outcomes can be enhanced through active engagement in a multi-modal cancer prehabilitation programme (MCPP), although this intervention is not uniformly embedded as a standard of care. MCPP aims to optimise patients physiologically and psychologically for cancer treatments, shorten recovery time, reduce complications, promote healthier lifestyles and improve quality of life. South Eastern Health and Social Care Trust (SET) developed and evaluated a system-wide collaborative approach to MMCP across three tumour groups (colorectal, lung, head and neck cancer). Addressing the lack of qualitative evaluation of MCPPs, this novel paper explores mechanisms promoting feasibility and acceptability of MCPP from patients’ and interdisciplinary professionals’ perspectives.

**Methods:**

Semi-structured virtual one-to-one interviews were conducted with 24 interdisciplinary professionals and nine patients. Transcripts were recorded, transcribed verbatim and themes developed using Framework Analysis.

**Results:**

Analysis of findings identified **three** themes providing an in-depth understanding of key elements required to develop and promote system-wide delivery of a MCPP: 1) Equipping the team: Capability and capacity, 2) Timing of intervention and delivery timeframe and 3) Systems and processes.

**Conclusion:**

The system-wide collaborative approach to developing a MCPP was deemed both feasible and acceptable. Success was attributed to visionary leadership, alongside a diverse group of interdisciplinary professionals being engaged, motivated and committed to intervention delivery in an effort to improve patient outcomes. Iterative, responsive troubleshooting during initial delivery is required to facilitate successful implementation. Further training is required for greater adherence to provision of prescriptive high intensity exercise within the programme, which may further promote enhanced patient outcomes. To enable sustainability of MCPP, ongoing training for professionals and funding is required.

## Introduction

Despite advancements in cancer treatment, 15–40% of patients with cancer who undergo surgical treatment experience postoperative complications [1]. As a result, patients may experience increased hospital stays or hospital readmissions, with negative impacts to physical functioning, psychosocial outcomes and overall quality of life [2]. Macmillan Cancer Support and the Royal College of Anaesthetists suggest that cancer prehabilitation should underpin the whole cancer pathway [3]. Taking forward this guidance, the Cancer Strategy for NI 2022–2032 [4] proposes that cancer prehabilitation should be available to all those who would benefit. Despite this recommendation and the growing body of evidence, cancer prehabilitation is currently not embedded as a standard of care within cancer care pathways locally, nationally or internationally [5]. This is due to a myriad of reasons, including workforce shortages, funding **issues**, lack of evidence around best practice models and need for addition information on cost-effectiveness [5–7].

Multi-modal cancer prehabilitation (MCPP) aims to optimise physical and psychological health through delivery of a series of tailored interventions including exercise, nutrition, and psychological support alongside behaviour change support for alcohol consumption and smoking [8], from the time of cancer diagnosis to the beginning of acute treatment. Most supporting evidence for MCPP is provided for the following tumour groups, colorectal (CRC), lung and head and neck (HN) [1, 9–11]. Studies evaluating the efficacy of MCPP have discovered patient benefits even when implemented for just two weeks prior to treatment [12]. Patient benefits can include improved physiological function through cardiorespiratory fitness and emotional resilience, shorter recovery time, reductions to peri-operative complications, gaining a sense of control over uncertainties ensued from a cancer diagnosis, improving quality of life and positive impacts on long-term health through behaviour change [12, 13]. While people regain more control over their own health, the NHS and other healthcare systems have the potential to make economic savings [6].

**Fig 1:**
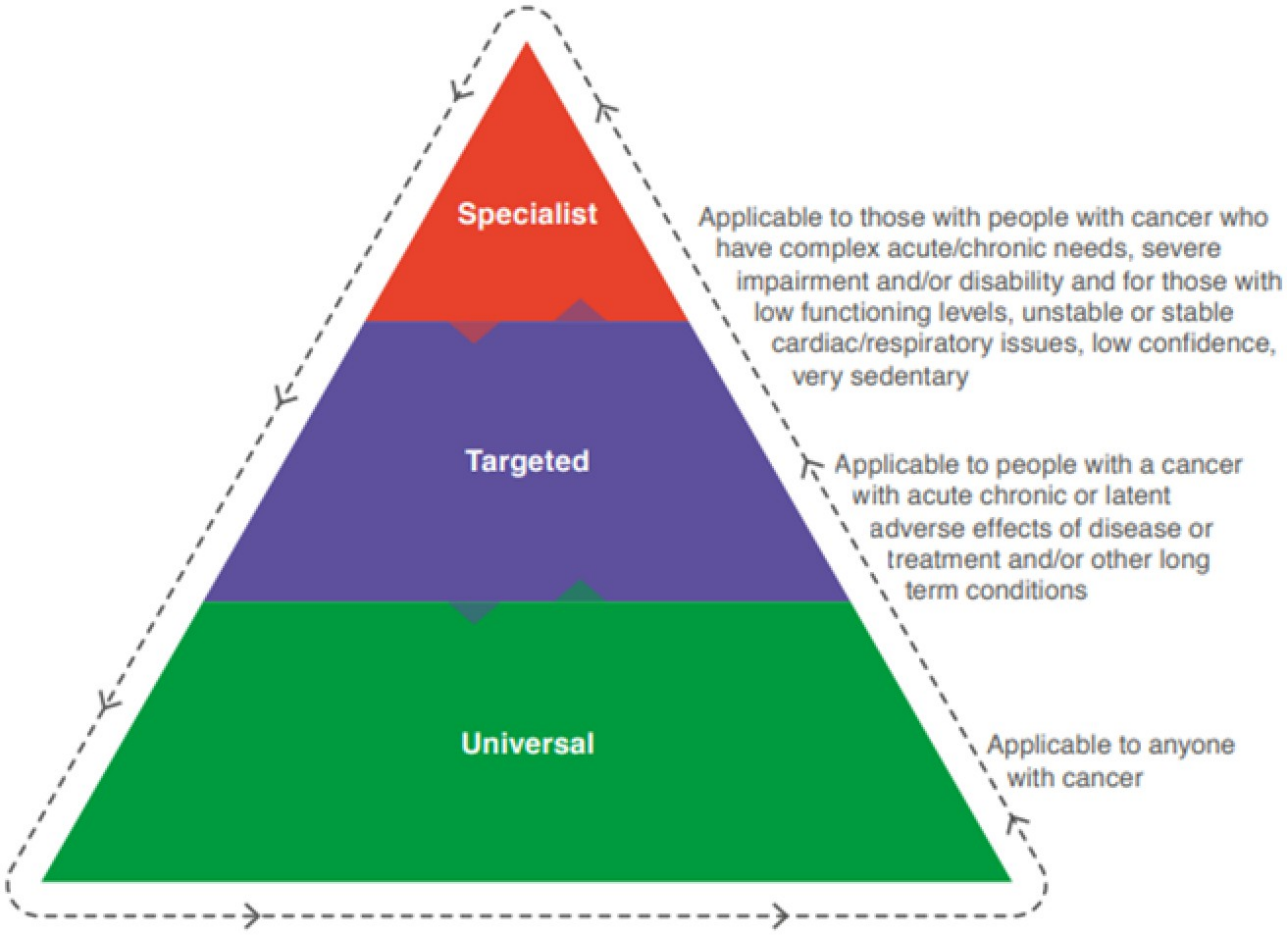
Stepped care framework for prehabilitation intervention [5].

A cancer unit within South Eastern Health and Social Care Trust (SET), United Kingdom, systematically planned, developed, implemented and evaluated a MCPP across CRC, lung and HN tumour groups during the COVID-19 pandemic with no additional funding [14]. The SET MCPP is a personalised and tailored MCPP, with referral pathways for individual’s based on screening using validated measures. The intervention focuses upon functional, emotional and nutritional needs aligned to patients’ tumour group and current lifestyle habits (smoking and alcohol) using the universal-targeted-specialised conceptual framework endorsed by Macmillan Cancer Support, the Royal College of Anaesthetists and National Institute for Health Research Cancer and Nutrition Collaboration [15] (see Fig 1). The universal and targeted exercise pathway aimed to have patients completing 3 high intensity training (HIIT) sessions per week, and the specialist pathway involved a physiotherapist-led bespoke cardiovascular and strengthening session per week with prescribed home exercise for alternate days. Universal emotional support was provided by Macmillan Move More Co-ordinators (MMCs) who had Level 4 Personal Training alongside generalist emotional support training; with targeted and specialist pathways delivered by counsellors /assistant or clinical psychologist respectively. Similarly, universal nutritional advice was provided by MMCs focusing on healthy eating and recognising weight loss, with targeted pathway delivered by dietetic support worker and specialist tier receiving input from dietitian to provide complex assessment and bespoke dietary prescription. Smoking cessation support was provided when necessary, taking a motivational interviewing approach and providing advice on pharmacotherapies. Substance misuse liaison provided alcohol advice, education, and signposting to referral services for patient as indicated. The purpose of the MCPP was to improve patient function prior to treatment and optimise health and wellbeing [14]. From an organisational perspective the MCPP at SET sought to develop and harness collaborative interdisciplinary working across a range of partners, promote good practice, foster shared learning, inform restructuring of services, scale-up and roll-out of similar programmes. Many healthcare interventions are complex in nature; for example, they can consist of several interacting components, delivered across many departmental boundaries, within a healthcare organisation. Successful implementation depends on the acceptability of the intervention to both intervention deliverers and those receiving it (patients and healthcare professionals) [16]. Given the complexity of SET’s MCPP [14] which comprised of interplay between interdisciplinary roles across a multi-sectoral team being planned and implemented in a real-world environment, qualitative methods were deemed appropriate for evaluation of acceptability and feasibility. This would enable an enhanced understanding of processes and mechanisms leading to MCPP’s success and failure, paramount prior to scaling up and further roll-out [16]. To date, the authors are aware of only one MCPP qualitative feasibility study, however the focus was solely upon delivery fidelity and acceptability [17]. This novel paper aims to explore mechanisms promoting feasibility and acceptability of a MCPP from patients’ and professionals’ perspectives exploring planning, development and implementation phases. The objectives were to gain understanding of mechanisms that:

1. promoted and informed planning and development of MCPP
2. promoted delivery of a personalised and tailored MCPP from patients’ and professionals’ perspectives
3. promoted acceptability and feasibility of MCPP implementation in a real-life context from patients’ and professionals’ perspectives.

## Method

### Study design

The overarching theoretical framework used was qualitative descriptive design [18], employing semi-structured interviews to explore stakeholder and patients’ perspectives of the MCPP.

### Sample and recruitment

Potential stakeholders were purposefully sampled to ensure representation of stakeholders involved in the key stages of MCPP; notably planning, development, screening and its delivery, hence recruitment of participants from a strategic and delivery perspective. Representation of participants who were fundamental to the delivery of MCPP, across all elements of the stepped care pathway, aligned to the three tumour groups (HNC, CRC and lung cancer), from clinical and non-clinical backgrounds, namely medical; clinical nurse specialist (CNS); smoking cessation and alcohol liaison team; allied health professionals (AHPs) to include dietitian, speech and language therapist and physiotherapist and Move More Co-ordinators (MMCs) were sampled. It was also important to recruit participants from a system-wide perspective, therefore sampling include participants with administration roles within SET, local councils and Macmillan Cancer Support.

The Principal Investigator (PI) (CJS) identified 30 potential strategic and delivery stakeholder participants, who were then emailed by PI with study invite, participant information sheet and consent form. All stakeholder participants’ who were interested in taking part or wished to gain more information about the study were asked to correspond via email with post-doctoral researcher, external to SET but known to two stakeholders.

The second participant study population was patients, who were purposefully selected to maximise variation in terms of gender, engagement (drop-outs and completers) and tumour group (CRC = 10, Lung = 5, HN = 5) and contacted via email or telephone by a CNS who acted as the Local Collaborator to ascertain if their details could be forwarded to the post-doctoral researcher. Inclusion and exclusion criteria of both sample populations can be reviewed in Table 1. Upon agreement for their details to be shared with researcher, these potential participants were provided with a study invite, participant information sheet and consent form. Again, all patient participants corresponded directly with the post-doctoral researcher (SLB) external to SET, if they had any further questions and confirm consent. All participants were recruited to the study between February and May 2022. Ethical approval for the study was granted by Ulster University Nursing and Health Research Filter Committee (FCNUR- 22–005) and written informed consent was gained from each participant prior to study involvement. Distress protocol was in place for the patient study population, but not required.

**Table 1:** Inclusion / exclusion criteria for semi-structured interviews.

	Inclusion criteria	Exclusion criteria
**Interdisciplinary professionals**	Registered healthcare professionals (HPs) (CNS, AHP, medical staff), MMCs, Health Development and Performance management, Cancer Services management directly involved in the development, screening, referral, or delivery of MCPP at SET Able to provide informed consent	Those meeting inclusion criteria but on leave during data collection period Healthcare students
**Patients**	Age >18 years old Diagnosis of CRC, lung or HN cancer Referred to SET MCPP Able to provide informed consent Communicate in English	Patients deemed too unwell by relevant CNS at time of data collection Receiving end-of-life treatment Dementia or cognitive impairment

### Data collection

To facilitate exploration of feasibility and acceptability of MCPP, the Theoretical Framework of Acceptability (TFA) [19] and the Normalising Process Theory (NPT) [20] guided the development of topic guides used with each study population. The TFA is a multi-faceted framework to guide the assessment of acceptability based on anticipated, experiential, cognitive and emotional responses to the intervention, including constructs of affective attitude, burden, coherence, perceived effectiveness and self-efficacy. The NPT comprises of four components which seek to understand what individuals and groups do to enable an intervention to be normalised. These components are: 1) coherence (sense-making), 2) cognitive participation (engagement), 3) collective action (work to enable intervention to happen) and 4) reflexive monitoring (formal and informal appraisal of the benefits and costs of the intervention). Furthermore, topic guides developed were informed by literature and knowledge from subject experts and piloted with one strategic stakeholder and patient prior to data collection, and stakeholder topic guide was subsequently iteratively updated. Semi-structured interviews were conducted by SLB using a virtual platform or telephone and a reflective journal kept during the data collection process. Interviews were recorded and transcribed verbatim by either SLB or professional transcriber. Average interview duration was 28 minutes (range 11–49 minutes) and 15 minutes (range 4–23 minutes) for strategic and delivery stakeholders and patients respectively. Quotes included in the evaluation had repeated words and unnecessary colloquial phrases e.g. “you know” removed, and the interviewer added detail within [] to improve coherence.

#### Data analysis

Data was analysed using the five steps outlined within Framework Analysis, which is widely used in healthcare research, enabling data immersion, reduction and a comparison of data across and within ‘cases’ before themes emerge [21]. Framework analysis sits outside any epistemological perspective providing the research team flexibility in analysis, while providing a method to account for perspectives from a diverse group of participants [21]. This data analysis approach assisted exploration of commonalities and differences in the qualitative data, before focusing on relationships between different parts of the data (strategic stakeholders, delivery stakeholders and patients), thereby seeking to draw descriptive and explanatory conclusions focused around themes.

Initially the interviews were transcribed, and transcripts were checked for validity by one author (SLB) and read by all authors gaining familiarisation of the data. A selection of the transcripts were inductively and independently coded by two authors (SJB n = 8 and CJS n = 12), ensuring interviews were included from each of the study populations (strategic stakeholders, delivery stakeholders and patients). A preliminary analytical framework was developed through team discussion (SJB and CJS), guided by the aforementioned theories (TFA and NPT), with four themes identified at this early stage (1. Engaging others, 2. Leadership and shared vision, 3. Systems and processes and 4. Moving forward: sustainability). The first author (SJB) analysed line by line the remainder of the transcripts with the aid of NVivo 13, being a specialist software package used to assist with data storage, management and retrieval. The coded data were entered into a framework using the preliminary themes (SJB). Data was then compared and analysed across the three stakeholder groups, relating to strategic, delivery and patient roles. Following team discussion the themes were refined and defined to ensure the correct meaning of the participants had been captured, with three final themes identified.

## Results

Participating strategic and delivery stakeholders (n = 24) (n = 6 no response) represented a range of disciplines, tumour groups, and sectors (see Table 2) and 9 patients represented 3 tumour groups with 11 declining participation as outlined in Table 3.

**Table 2:** Demographics of strategic and delivery stakeholders.

	N (%)
**Interdisciplinary roles**	
Registered Nurse (includes CNSs, (n = 5) Smoking Cessation Team (n = 1), Alcohol Liaison (n = 1))	7 (30)
Doctor	4 (17)
Speech and Language Therapist	1 (4)
Physiotherapist	2 (9)
Dietitian	1 (4)
MMC	3 (12)
Assistant psychologist	1 (4)
Emotional wellbeing support	1 (4)
Health Development Manager	2 (8)
Performance Manager	1 (4)
Cancer Services Manager	1 (4)
**Gender**	
Male	5 (21)
Female	19 (79)
**Attended specific prehab training in advance of SET MCPP**	
Yes	3 (12)
No	21 (88)
**Tumour specialism (n = 15)**	
Lung	2 (13)
CRC	5 (33)
HN	3 (20)
HPs working across tumour groups	5 (33)

**Table 3:** Patient demographics (n = 9).

	N (%)		N (%)
**Engagement with evaluation (n = 20 approached)**		**Support Network**	
Accepted	9 (45)	Very good	8 (89)
Approached—No response	6 (30)	Poor	1 (11)
Initially interested—Withdrew, unknown reason	3 (15)	**Educational status**	
Declined—No recollection of MCPP	1 (5)	Secondary school	5 (56)
Declined—Unwell	1 (5)	Third level education	4 (44)
**Tumour group**		**Frequency of exercise prior to diagnosis**	
HN	2 (22)	Regular	6 (67)
Lung	4 (45)	Somewhat active	2 (22)
CRC	3 (33)	Never	1 (11)
**Age**		**Access to video platform**	
46–60	2 (22)	Yes	8 (89)
61–75	6 (67)	No	1 (11)
76+	1 (11)	**Comfort using video platform**	
**Gender**		Very confident	4 (44)
Male	6 (67)	Fairly confident	1 (11)
Female	3 (33)	Not confident	4 (44)
**Marital status**		**How was the MCPP delivered?**	
Living alone	1 (11)	Telephone	3 (33)
Married/Living with partner	7 (78)	Virtual and telephone	3 (33)
Living with family member/ friend	1 (11)	In-person	4 (44)
**Time from Diagnosis**			
1–2 months	1 (11)		
6–12 months	7 (78)		
12 months +	1 (11)		

Three themes were identified from stakeholder and patient data, 1) Engaging others, 2) Complexities around timing of MCPP and 3) Systems and processes

### Theme 1: Engaging others

Within this theme three sub-themes were identified: [1] Equipping our team: capability and capacity, [2] Leading for success and [3] Collaboration: It’s better together.

#### Subtheme [1.1] equipping our team: Capability and capacity

The MCPP team, a group of strategic and delivery stakeholders from clinical and non-clinical settings, working within and outside of cancer care, had at inception varied levels of knowledge about the principles of MCPP and how it integrated into the cancer care continuum. While representation was diverse, the patient voice was acknowledged in the ‘terms of reference’ but actual presence did not form part of the stakeholder group. Many stakeholders with strategic roles, had attended presentations about the GM MCPP, or had met directly with stakeholders from the GM MCPP team, therefore had greater levels of knowledge surrounding the underpinning concepts, benefits of, and multimodal components of cancer prehabilitation.

*“I had been to a conference that was run by the Royal Marsden and I heard a very impressive speaker*, *he was an anaesthetist*, *his name is John Moore*, *he has actually been instrumental in the setting up of the cancer prehabilitation in the Greater Manchester area*. *I could see from his data that there was a great merit in cancer prehabilitation*, *in getting patients into peak performance or optimised for the treatment that’s ahead*.*”*

(P1, CNS HN, Strategic MCPP lead and delivery stakeholder)

*“Way back the beginning*, *we had a very comprehensive sort of presentation of the Manchester model”*

(P11, Health Development Manager, Strategic stakeholder)

At project inception, proportionately more strategic stakeholders made reference to knowledge gained from current literature or attending courses and conferences than the professionals responsible for delivering MCPP, hence an obvious educational requirement for non-strategic stakeholders. This gap in MCPP knowledge was even more apparent for professionals delivering pathway elements of MCPP from a subject-specific discipline, such as a pelvic floor physiotherapist, smoking cessation team, assistant psychologist with no clinical background in cancer care.

*I didn’t know an awful lot at all so it was very new to me*, *and obviously working within sort of pelvic health*, *I knew it was probably an area that we could be involved in*. *But I really didn’t have very much knowledge about even the surgery that is involved with cancers*, *so a big learning curve*, *really*, *for myself*. *And I had no idea what the prehab involved*, *sort of read wee bits and pieces regarding prehab in different areas*, *but very little knowledge*.

(P22, Pelvic Physiotherapist, Delivery stakeholder)

Despite requiring knowledge about the principles of MCPP, their current discipline-specific clinical skills proved transferable with no or low levels of additional clinical skills training required. Also, delivery stakeholders described a sense of preparedness for this new aspect to their role that was attributed to the co-production process which involved engagement in steering group meetings, task and finish groups, development of the Standard Operating Procedure and their transferrable skills that could be harnessed for the new project.

*“Because I’m an Assistant psychologist … I was asked to be part of the cancer prehab*, *because a lot of the things that we would do in that service would transfer well in with cancer prehab and helping people with their anxiety coming up to their*, *their treatment*.*”*

(P10, Assistant Psychologist, Delivery stakeholder)

For others, despite having previous experience and training in delivering care to cancer patients, additional training and support was required. The was due to both the delivery of care earlier in the cancer continuum and role expansion as part of MCPP. For example, MMC’s had previously received formal training from Macmillan Cancer Support on “Managing difficult conversations” and “Motivational Interviewing” (P24 MMC), nonetheless lacked experience supporting patients at diagnosis. P2 (MMC, Delivery stakeholder) reported,

*“Traditionally*, *with Move More*, *we would have seen those [patients] sort of post-treatment*, *probably in treatment and post-treatment*, *post-treatment would have been our biggest*.”

Role expansion for the MMCs was considered within the bespoke training package provided before the commencement of the MCPP service, covering areas such as ‘Healthy eating—nutrition’, ‘Smoking cessation’, ‘Alcohol and You’, exercises to promote “*core stability*” for patients with CRC cancers and “*head*, *neck and shoulder exercises*” for patients with HN cancers (P1, CNS HN, Strategic MCPP lead and delivery stakeholder). Despite MMCs receiving basic training to provide nutritional advice and emotional support under universal pathway; findings highlighted that MMCs focused less on these elements due to perceptions that this was not the MMC’s main area of expertise.

*“I wouldn’t say it’s [nutritional advice provision] very in depth on any level*, *you know what we’re not nutritionally trained and I suppose like any physical activity coordinator*, *somebody comes looking nutritional in-depth advice*, *you know*, *we refer them on because that’s not our forte*, *we’re not trained in that*, *but we do have basic knowledge and understanding*.*”*

(P2, MMC, Delivery stakeholder)

MMCs were also provided with training related to collecting data using functional and patient-reported outcome measures. Despite the bespoke training package provided for MMC, the lack of experience managing emotional distress often associated with a new cancer diagnosis did impact negatively upon stakeholders’ confidence when engaging with patients early in the treatment trajectory, with MMCs reporting a desire and need for specific training which was initially lacking.

Training proved to be an ongoing requirement, to promote engagement of professionals delivering MCPP and for new team members. When issues hindering implementation of the MCPP across disciplines were identified, such as provision of exercise prescriptions and advocating HIIT within the targeted exercise pathway, further appropriate and timely training was offered to enhance fidelity of the intervention in accordance with the ‘Standard Operating Procedure’ manual. This appeared to have limited impact with MMCs believing interventions like HIIT to be unrealistic for some patients referred to the MCPP.

*“HIIT training is very*, *you know*, *targeted*, *you’re working on a certain percentage of your max heart rate*, *you’re getting a certain ratio rest*, *work to rest ratio*, *it has to be within certain zones… there’s no way you’re putting somebody with maybe a heart condition or who’s obese*, *bad knees*, *hips*, *into a HIIT session and even 30 seconds on 30 seconds off is so general*, *so I didn’t believe it and I’m still very sceptical of it… I was very much like … has to be patient centred*, *has to be what they’re able to do*. *You’re ringing ladies who are maybe 70 or 80 who maybe are bed bound or maybe go out once a week to do their shopping*, *never been in a gym in their life*, *so it had to be home based kind of functional movements that they were used to doing you know*.”

(P2, MMC, Delivery Stakeholder)

On the other hand, patients viewed the exercise programme positively, reflecting on its varied, patient-centred and achievable attributes, including indoor and outdoor options.

*“Yeah*, *the exercises were*, *you know*, *they were sort of geared*. *So*, *I thought there were*, *I thought there were really good*, *appropriate ones*. *So*, *it wouldn’t have necessarily been*, *you know*, *I mean*, *the fact that I would have done some exercise*, *I’d probably just like*, *go out running or something*. *So*, *it was good to have much more specific exercises that were relevant*.*”*

(Patient 7, CRC)

Where stakeholders joined service delivery later or when issues with data quality were identified, the Performance Manager or the appropriate professional stakeholder provided much needed informal sessions which were highly regarded by stakeholders. Ongoing training, responsive to stakeholders needs facilitated embedding of MCPP within clinical pathways or improvement in the quality of data captured. This tailored and flexible training approach aligned to need of professional, was welcomed and beneficial.

“[Performance Management Lead] …extremely approachable and contactable”

(P23, CNS HN, Delivery stakeholder).

*“They were very good*, *like [PERFORMANCE MANAGER LEAD] was very good*. *And*, *you know*, *meeting me on my own and going through everything*. *So*, *like I wasn’t thrown in at the deep end and say*, *right*, *go fill that form in*. *And like*, *there was a lot of preparation*, *it was probably more just for my own*, *wanting to make sure that I do it right*, *and do it well*. *But like*, *we met with [HEALTH DEVELOPMENT MANAGER] and separately as well*, *went through like the SOP*. *And like*, *I supposed I was prepared*.*”*

(P24, MMC, Delivery stakeholder)

Capability was well addressed for many, however, there was greater challenge surrounding capacity. Some of the capacity demands were addressed by facilitating the use of online platforms and reducing travel expectations to meetings. Nonetheless, capacity issues remained for some delivery stakeholders from clinical backgrounds, especially relating to some AHPs, namely SLT, who was not able to continue providing input into the one-stop HN cancer prehabilitation clinic due to workforce pressures.

*“And I’m sure [CNS HN] would agree that you know*, *within the One Stop clinic and speech and language there are big gaps*, *which we struggle to*, *to fill*. *Yeah*., *so certainly*, *resourced just a resourced prehab programme with specialist staff*, *you know*, *doing those roles*.*”*

(P13, Strategic AHP lead for MCPP)

Looking to the future, strategic and delivery stakeholders were clear that provision of recurrent funding was the most pressing reported priority to enable a sustained model of funding for interdisciplinary roles to take forward MCPP.

*“If SLT was funded for it that would be it*, *it would solve everything*. *Yeah*, *I mean*, *that’s sort of is if you like*, *it’s the bottom line*. *To know*, *we just*, *you know*, *we need funding to provide this service*.

(P19, SLT, Delivery stakeholder)

Participants also reported a concern about capacity to deliver equitable services across trust boundaries, as geographical limitations to service provision prevented patients referred from other Health and Social Care Trusts (HSCTs) to elements of the MCPP. Strategic stakeholders concurred that future funding of MCPPs should include provision for project management, professionals’ roles including MMCs, AHP specialities, development of IT system alongside data management personnel and account for project scale-up (incorporating further tumour groups), while taking account for staff leave.

#### Subtheme [1.2] leadership and shared vision: Striving for success

Key drivers to MCPPs successful implementation were perceived to be leadership, a shared and coherent vision and commitment of strategic and delivery stakeholders. Evidence of effective leadership was positively reported by multiple stakeholders (clinical and non-clinical) during MCPP planning, development and implementation phases, which enabled MCPP to become increasingly embedded into an integrated pathway.

*“To be fair*, *[Strategic clinical programme lead] can motivate people and get people around the table and do that’s kind of exactly what made such a difference*.*”*

(P18—Smoking Cessation team, Delivery stakeholder)

From a strategic stakeholder perspective, the success of the project was also attributed to having a shared vision with agreed aims and objectives that centred on using a credible model as the basis for the development of the SET MCPP and senior management buy-in.

“*When we first started to talk about this*, *we did discuss this with the director of hospital services with the assistant director*. *So*, *I think at the very beginning*, *we had buy-in from that level*, *which was really*, *really important… I think that that sort of commitment from senior level has been really important*, *that they were committed and they had given the yes*, *we want to take this forward has been key to it*.

(P11, Health Development Team, Strategic stakeholder)

For some stakeholders, this sense of shared vision developed over time through exposure to the planning and development process. P4 (CNS CRC, Strategic and delivery stakeholder) reported,

*“[It] did take a wee while to get my head round it*, *you know*, *have an understanding of the ethos of it all*. *… I was involved at every stage*. *And then sort of my understanding grew and how that was going to work for us*, *whether it was going to work in real time and how it was going to work with our structure of our clinics*, *and the timeframe that we see those patients and how we in practice were going to get the information over to the patients*, *how it was going to work with regards to the verbal information we give them*, *the written information we give them and how the referral process worked*.*”*

All stakeholders, which included patients and professionals had a shared and coherent vision that MCPP could improve patient outcomes, which was an important facilitator for this new intervention.

*“Oh it’s*, *it’s brilliant*, *it’s*, *it’s a no brainer*, *if it if it helps to optimise them*, *the patient before they’ve had they’re having surgery or treatment*, *any type of treatment*, *that that*, *you know*, *can have a positive impact on their recovery period*, *and also on their survival outcomes*. *But it’s*, *it’s*, *that’s great*.

(P20, Manager, Strategic stakeholder)

*“No*, *I was keen*. *I think the whole thing is*, *is such a good idea*. *Yeah*, *I mean*, *when they asked*, *when they asked me to take part like it was a no brainer*, *because I just think it’s people talk about post-rehabilitation and trying to get back to you know*, *yeah*. *Getting ready for treatment and surgery just sounds like a no brainer*. *Yeah*, *happy to take part because of that*.*”*

(Patient 7, CRC)

For one stakeholder, the MCPP provided an opportunity to not only maximise on the ‘*teachable moment’* but engage in shared care between patients and clinicians. P1 (CNS HN, Strategic MCPP lead and delivery stakeholder) highlighted MCPP as an

*“[It’s an] opportunity to give some control back to the patient … and is a prime opportunity to enable and engage people early in a shared care model of their treatment*.”

Of note, not all patients had a clear recall of the key element and benefits of MCPP. Patients lacking MCPP coherence had an inability to clearly report on their MCPP experiences, either positively or negatively. Instead, MCPP merged and morphed collectively as their pre-treatment cancer experience.

#### Subtheme [1.3] collaborative working: It’s better together

Collaborative working was evident between strategic and delivery stakeholders through the steering group, task and finish groups and working group meetings with stakeholders have clearly defined roles, for example, developing the Standard Operating Procedures, decision making on functional assessments, developing inclusion / exclusion criteria. While collaborative working was considered by some as time consuming, it brought with it many benefits. Primarily, the strength of the collaborative model enabled interdisciplinary working, which enhanced understanding of professional roles and the development of new working relationships, spanning beyond the MCPP programme.

*“My work [is] not cancer focused*. *I was meeting other professionals that I’ve never ever had been dealing with and involvement with Health Development [Team] …also the Councils and Macmillan … so that I just felt it was fabulous*, *the way everybody worked together… I just felt that the working together and the troubleshooting and the problem solving was great*.*“*

(P14, Physiotherapist, Delivery stakeholder)

Strategic and delivery stakeholders reported skill development as a consequence of being involved in the MCPP, and for some participants this collaborative multisectoral working promoted job satisfaction. This was especially evidence for clinical stakeholders delivering MCPP, who reported an increased sense of value in their work, with satisfaction sharing project progress with other healthcare Trusts and Department of Health.

*“I think you definitely feel like it’s another sort of element to the dietetic role that it’s because you are part of the MDT [multidisciplinary team] and part of the project*, *it’s a lot more*, *you feel very valued I suppose and your input very beneficial*.*”*

(P8, Dietetic, Delivery stakeholder)

Good communication was a key element to the collaboration. To build the collaboration, local Councils were approached on an individual basis utilising existing relationships with Health Development Team. While there was generally good Council buy-in to the MCPP reported, there was a point at which Council participation was in jeopardy for one of the regions, necessitating strategic leadership and negotiation to achieve resolution. However, for the MMC working in that particular region, communication with Council senior management was perceived as lacking, with recommendations for clear channels of communication and reporting mechanisms.

*“Greater communication between the stakeholders and council and support for coordinators*, *…instead of just relying on someone doing it*, *actually knowing who the person is that is supporting the co-ordinators*, *if you know what I mean*.*”*

(P12, MMC, Delivery stakeholder)

A number of participants reported that collaborative working was facilitated with the early development of quarterly Outcomes Based Accountability scorecards. This was reported by participants as a helpful method to engage delivery stakeholders, providing them with regular data on measurables for monitoring progress and evaluation purposes. Furthermore, collaboration was harnessed through good communication in working group meetings, facilitating timely identification of problems and co-produced troubleshooting.

“*Having those continued and sustained on a regular basis and for me is never make assumptions that those delivering a service are actually delivering*.”

(P1 CNS HN, Strategic and delivery stakeholder)

Patient participants reported on the important of delivery stakeholders being motivating and encouraging about their involvement in the MCPP. Patient 1 (Lung cancer) described:

*“When you get your diagnosis of cancer*, *you sit there and you get this diagnosis that you spend your entire life*, *hoping you never do*… *you just think your world has ended*, *you know*, *and being reached out to by somebody who is saying*, *no*. *You know*, *we’re going to we can stay positive through this*. *We can stay moving*, *we can keep in touch*, *you can stay moving… And I found that even that alone*, *the reach out*, *somebody’s reaching out and saying*, *no*, *no*, *come on*, *[Patient name] we’re going to do this*.*”*

Patients also highlighted derived benefits from playing their role within the collaboration; through increased fitness, reducing or quitting smoking and alcohol, and a sense of improved treatment outcomes benefits highlighted their biggest benefit.

*“Well*, *it was the exercises that the nurse was doing with me…*.*You know all in all it seemed dead simple…You can get help whenever you want*. *There’s just so simple*, *but the difference it was unbelievable… he [Surgeon] said my fitness was fine to do surgery*.*”*

(Patient 6, Lung cancer)

There was a great sense of patient gratitude for the MCPP, with patients highlighting professionals’ commitment and availability to communicate with them between appointments.

*“I think it’s something that will benefit patients and the Health Service both*. *I thought it was well-managed*, *well-run*, *and just [want to] say thank you*.*”*

(Patient 4, Lung cancer)

Embedded within MCPP for HNC patients was the 1-stop clinic, which included opting into a facilitated peer visit. HNC patients highlighted the benefit of having an opportunity to hear about a previous patient’s experience, with one participant reporting it as “*one of the best things that happened*” and “*very reassuring*” when they felt “*scared stiff*” (Patient 3, HNC). Patients recommended that this element of the collaboration should be embedded routinely as part of the MCPP.

Furthermore, family members were important to the collaborative approach, viewed by the patient and delivery stakeholders as supporting patient participation. Of note, one patient relayed their family member’s view of the value of the MCPP, particularly during the pandemic when patients were often isolated, reporting,

*“I was talking to my daughter-in-law*, *who*, *unfortunately has terminal cancer and has been living with us for a few years now…she said it was nice that I was able to talk to these people* [MMCP professionals], *have the support and know exactly what was ahead … she didn’t have that facility*.

(Patient 8, HN cancer)

Inclusion of family members or partners at appointments was perceived to support patient engagement, particularly when patients relied on them for transport. P12 (MMC, Delivery stakeholder) described,

*“So*, *you know*, *maybe somebody’s saying*, *‘Oh*, *I don’t drive*, *but my husband might be able to bring me in’*, *[and I would reply] well sure*, *he can come and take part in or he can do this*, *or he can do that*.*"*

### Theme 2: Complexities surrounding timing of the MCPP

An overarching challenge to MCPP implementation related to the timing of the intervention, and its delivery timeframe. MCPP was most often introduced to patients at their initial cancer diagnosis consultation. While agreed by all stakeholders as the necessary timepoint to maximise the prehabilitation timeframe, delivery stakeholders reported concerns that patients were often emotionally overwhelmed with little capacity to process further information. When considering the optimal time to discuss MCPP, P13 (Physiotherapist, Delivery stakeholder) reported,

*“Oh*, *as early as possible*, *to be honest*. *I think if the patient gets a lot of information at diagnosis and sometimes it’s*, *you know*, *it’s been a big blow and they maybe aren’t able to cope with that and I think the clinical nurse specialists at that time breaking bad news appointment are so far the best positioned people in order to make a judgement call on what*, *how much*, *or how little the patient can actually deal with on that particular day*. *But as early as possible*, *any kind of rehabilitation*, *the earlier you can get started then that*, *you know*, *you expect better outcomes*.*”*

Delivery stakeholders reported pressure incorporating an introduction to the MCPP into an already busy clinical conversations, sometimes perceiving patients to be already overwhelmed with information.

*“We are under a bit of pressure whenever we’re doing our results appointments*. *So*, *there may be two or three patients come up for results appointment*, *or the patients receiving their diagnosis are being told by the consultant about their planned surgery*. *And then our role is really to fill in*, *you know*, *a bit more of that information*, *clarify that giving them information about their cancer*, *about their surgery … and then we’re starting to talk a wee bit about prehabilitation*. *By this stage*, *their heads are*, *you know*, *really overwhelmed*, *to be honest*.*”*

(P4, CNS CRC, Strategic and delivery stakeholder)

Conversely, patient stakeholders did not report that being introduced to, and screened for the MPCC programme at their initial consultation added extra burden and were happy to participate. Patient 5 (CRC) reported,

*“No not at all*, *no*. *I’m very sanguine about the whole thing*, *I’m a very pragmatic sort of person*. *And*, *you know*, *the*, *I think a lot of people say*, *oh my god*, *cancer and all the rest of it So I just said*, *get this thing out of me*. *Sooner I can recover and get back on my feet the better*.*”*

The often-short timeframe between diagnosis and treatment was challenging for MMCs, especially if the timeframe was less than 2-weeks; reporting that patients often had to sort personal affairs and attend other clinical consultation. Delivery of MCPP was further impacted by COVID-19 guidance on isolation.

*“We’ve had to adapt with the [COVID-19] guidance*, *you know*, *the patients are obviously being swabbed and at different stages*, *you know*, *so if you think about a pathway*, *a patient’s been screened*, *they’ve been referred*, *they might have the time one [data collection]*, *but then they’re getting swabbed and told right your operations next week*, *but you have to isolate for 72 hours before your operation*, *you know*, *so it’s adjustments within the COVID guidance at the time to keep them on a green pathway for their treatment*.*”*

(P5, Doctor CRC, Strategic stakeholder)

Despite this, many stakeholders largely believed that benefits were still possible in the short timeframe, as P18 (Smoking cessation team, Delivery stakeholder) described,

*“I mean*, *lots of evidence to say that even if somebody stopped smoking about two to three days*, *prior to surgery…their bad days post op will be less and stuff*.*”*

Patients reported benefits within this timeframe, including reducing alcohol consumption, reducing or quitting smoking, feeling fitter and a sense of improved wellbeing, change for some which has become embedded as routine.

*“I think it actually did make me fitter*. *The fact that you get into a routine*, *now because I’m back at work*, *I can’t obviously go seven days a week*. *But I would still do that hike*, *maybe three or four days a week now and I think I would always have walked a dog*, *but I don’t think I’d have gone quite so far or made it quite so difficult*. *So*, *I think there’s probably a benefit from that as well*.*”*

(Patient 4, Lung cancer)

One of the key challenges surrounding timing of the MCPP was a direct consequence of the spurious theatre lists during COVID-19 pandemic. This often meant that patients were provided with limited notice for date of surgery, with MMCs having less time than anticipated for the programme. This consequently impacted capture of Time 2 functional and patient-reported outcome measures (post-MCPP and before commencement of cancer treatment). Most MMCs reported that striking a balance between data collection and intervention delivery was often difficult, with MMCs advocating for a longer programme delivery timeframe (at least 2-weeks) to ensure optimal data collection and delivery of multimodal components.

*“Once then you built in the functional assessments*, *by the time they came in to the centre and you did all you needed to do*, *it was looking more like half an hour*, *which was fine if they had 4 weeks to treatment and you were repeating that 4 weeks later or 6 weeks later*, *but whenever you were doing it on a Thursday and they were maybe going in for treatment 2 weeks later*, *you were doing it again the following Tuesday because maybe that was all they could do*, *so you were 10 days*, *so I think we have to be realistic about what that data collection is going to show*.*”*

(P12, MMC, Delivery stakeholder)

To enhance the programme, MMCs advocated for timely communication relating to patient treatment schedules suggesting this could improve collection of Time 2 data and intervention tailoring.

### Theme 3: Systems and processes

One of the benefits of progressing the MCPP at SET was formalising unimodal components into the MCPP system-wide pathway approach, with guidance provided through the Standard Operating Procedure.

*“We had elements of prehabilitation within our service beforehand so this was formalising some of it*. *From a smoking perspective we had an opt out*, *so anyone who was a smoker they had an automatic referral to the stop smoking team so it was formalising that pathway*. *Again*, *given the condition we work with being head and neck cancer*, *there’s a significant proportion of our patients have alcohol related issues or they’re alcohol dependent*, *so that’s part of our initial assessment anyway and we work very closely with the substance misuse team*, *the alcohol liaison team and it’s not something that we’re uncomfortable with probing into that area of people’s lives and making recommendations and onward referrals*, *so those were well established anyway”*

(P1, CNS HN, Strategic MCPP lead and delivery stakeholder)

Another essential process within the planning phase was obtaining a data sharing agreement between the healthcare trust and the council regions, as the MMC who were receiving and collating patient information were not healthcare trust employees.

Introduction of the MCPP was often done by the CNSs, but endorsement from the medical team was deemed important however, sometime lacking. This lack of endorsement was possibly a consequence of some of the medical staff being less aware of the growing evidence based surrounding the efficacy of MCPP.

*“It would be useful for me in conversations with patients*, *I can bring it into consultations … that would be the only thing*, *be useful for me actually*, *because I’m not that sort of [knowledgeable about] Macmillan pathway and all that [I have a] sort of appreciation from speaking to [CNS HN] and [CNS HN]*. *You know*, *it’s not something I fully understand because it’s not really my area*.

(P7, Doctor HN, Strategic and Delivery stakeholder)

The MMCs delivering the exercise component of prehabilitation, highlighted the importance of endorsement of exercise from the clinical teams to promote patient engagement.

*“I found it good in terms of you know you were ringing them and saying I’m [PARTICIPANNT NAME*,*] I’m part of the prehab team and they were like oh yes*, *so they had been told again*, *that was something we tried to drill in from infancy that*, *you know*, *it needs to be the consultant or the CNS because there’s been loads of research in terms of exercise and health but*, *you know*, *if the patient is told by a doctor to go and do x*, *y and z they’re more likely to*. *I don’t know the percentage off the top of my head*, *but they’re more likely to do it than somebody in a tracksuit in a gym going you should do this*, *so my feeling is that it’s definitely happening and the nurses are explaining that this is the prehab pack*.*”*

(P2, MMC, Delivery stakeholder)

One of the clear benefits of the screening process reported by strategic and delivering stakeholders was the selection of concise, validated tools with built-in intuitive prompts and drop-down boxes to aid user-friendly completion.

*“So*, *I think our tools are as simplified as you would want*, *to try*, *with the aim that it was meant to be accessible and user friendly to capture and to allow the maximum amount of use for*, *you know*, *for patients and for the screening and referral*, *you know*, *the referral*. *The screening process is very good in that it’s a very clear*, *well-constructed e-referral system and I think that works really well*.*”*

(P5, Doctor CRC, Strategic stakeholder)

In addition to the screening process, the online referral process developed for the SET MCPP was equally viewed favourably. Completion of one online form per patient, enabling referral to different pathways within the multimodal stepped-care framework, was deemed highly acceptable and advantageous.

*“No*, *it was quite straightforward*, *very simple process and a very simple referral form*. *So*, *it’s all drop down menus and tick boxes*, *and with a wee bit of additional information*, *so there’s no problems with that at all*.*”*

P14, CNS Lung, Delivery stakeholder)

During the planning phase, this online approach, acting as a single point of referral was keenly driven to avoid “*the copious referrals sometimes necessitated when assessing new patients*” (P1 CNS HN, Strategic MCPP lead and delivery stakeholder), whilst enabling a tailored MCPP.

“*A referral form … to just tick and select the components with the prehabilitation and personalise that and with one submit button automatic referrals would generate to the relevant people*, *so that was my vision so thankfully we’ve been able to action that which is one of the great wins in the system that we do have today… The other thing that we are very aware of that we had our MMC who sat*, *not within the trust*, *but within the council areas*, *so we needed to ensure that the patients were happy with their information being sent and we also had to find a safe channel to be able to send the information on*, *so that’s why we had to ensure there was a consent process also captured within the referral form*. *We needed the referral form to do a lot of things essentially*.

Despite CNSs reporting the online screening and referral process as easy to complete and use, early implementation of the form was hampered by missing information and submission of incomplete forms with P21 (CNS CRC) reporting *“I don’t think I realised the importance of filling in every single detail*,”. This issue was rectified by prompts and some real-time troubleshooting at the regular working group meetings.

*“With some additional refresher and with additional reminders that has worked well*.*”*

(P5, Doctor CRC, Strategic stakeholder)

Another supportive process embedded into the MCPP at SET, highlighted by many of the strategic and delivery stakeholders was the regular working group meetings. This provided a conduit for support for stakeholders delivering the programme, an opportunity to iteratively refine elements of the programme in real-time and establish any ongoing training needs.

*“Yeah*, *in terms of support and that we had the right information and that if we needed to go back to them and ask a question*, *yeah*, *they were there*. *We had sort of fortnightly meetings… So*, *in terms of*, *if you got a difficult case or maybe you weren’t quite sure*, *I found those meetings were beneficial because you were able to say*, *“Look I got this referral last week*, *they didn’t want to take part*, *I’ve rang them twice or I got this” so you were able nearly to sort of say*, *“what do you think of this” “How could we approach this better*?*” and that sort of thing*.*”*

(P12, MMC, Delivery stakeholder)

## Discussion

This qualitative evaluation provided an enhanced understanding of mechanisms leading to the feasibility and acceptability of the MCPP in SET; in conjunction with important attributes that could be changed or adopted to augment scaling up of the intervention. Despite the COVID pandemic backdrop, interdisciplinary collaboration across multisectoral organisations forged ahead without funding, maximising upon innovation and creativity to integrate pathways for a MCPP, striving for better cancer patient-outcomes at SET. Success was mainly due to the visionary leadership and a diverse and committed group of interdisciplinary stakeholders working together to adapt and refine a tailored stepped-care MCPP for local implementation at a Cancer Unit in Northern Ireland [14]. Current research investigating the feasibility and acceptability of MCPPs from the perspectives of professionals and patients is exceptionally limited [17] with studies often referring to patient recruitment and participation as indicators of success [22, 23]. The findings from this study extend current evidence by illustrating the mechanisms of success and challenges experienced by those at SET and will be discussed through the lens of NPT components [20], coherence, cognitive participation, collective action and reflexive monitoring.

High levels of *coherence* were evident across interdisciplinary groups. Most professionals were clear on how MCPP could complement and be a vital part of the cancer care continuum. Key facilitators, such as having knowledge that MCPP can positively impact patients’ functional and emotional outcomes [5, 24, 25] has promoted successful implementation of MCPP. Of note, for some, greater coherence could be facilitated through an enhanced appraisal of the recent growing body of literature surrounding the efficacy of MCPP. For example, some doctors in this study were less aware of the multifaceted components of the programme and known benefits, even within a short timeframe of less than two weeks [12]. Rectifying this is important as patients, both within this study and elsewhere articulate that doctors’ communication on the topic influences their uptake and engagement with MCPP [26]. High coherence can enhance patient recruitment and adherence [27]. Patients who understood the purpose and benefits of the programme were more committed to the programme, with the converse also evident. High coherence in the short time-period between cancer diagnosis and treatment commencing can be challenging to achieve given levels of diagnosis-related stress patients experience at the time of introduction to the MCPP, thus an evident need for professionals to be skilled in communication, facilitation, motivational interviewing techniques and providing emotional support [28].

The MCPP at SET successfully engaged a varied and committed skilled workforce across tumour groups including those with strategic, clinical and physical activity knowledge and skills, reflecting good *cognitive participation* [19]. Partnering, through established links, with local councils and community and voluntary sector was advantageous as there was already established a sense of trustworthiness. Similar partnership arrangements were also reported as a key to success when Greater Manchester adopted a system-wide approach to MCPP [29]. Involvement from key strategic stakeholders and delivery professionals early in programme development and in an on-going manner was crucial to success, alongside ensuring that decision-making occurs in a collaborative and shared manner [26]. Cross-sectoral collaborations, however, can be challenging given differing reporting and funding structures [30]. This was somewhat the case for MMCs during the MCPP, which could be addressed by closer participative working with local council employers. One notable perspective less visible during the development phase of the MCPP was that of the patient. Best practice [31, 32] states it is essential to involve end-users of the intervention throughout development to ensure that the intervention is acceptable, engaging and feasible, anticipating the needs of others, as often this not effective. Representative and proactive patient involvement may effectively address some of the difficulties with coherence mentioned above [24].

*Collective action* is characterised by how people work together to achieve outcomes, build confidence and organisational support [33]. Collaboration between engaged and envisioned professionals was integral to the development, implementation and ultimately the success of this programme. Each discipline had clear roles throughout development and implementation, reporting back on progress at working group meetings. Active participation throughout development and implementation contributed to a sense of self-efficacy, with professionals highlighting personal progress working with patients with a cancer diagnosis, screening and outcome tools. Complex interventions delivered in complex systems [34] require high intervention fidelity to accurately determine the impact of the intervention under study [35]. Delivery of the SET MCPP exercise component was critiqued by some healthcare professionals at a strategic level as requiring more specific and intensive exercise prescription, to ensure fidelity of the agreed exercise doses outlined in the MCPP Standard Operating Procedure manual. Murdoch *et al*. [17] study of their MCPP with a CRC patient group suggested that while professionals emphasised that fitness was important for surgery outcomes, patients were not encouraged to increase activity levels. Herein, they noted that the lack of exercise prescription was attributed to a short timeframe between diagnosis and treatment, plus constrained resources with clinicians being too busy [17]. In contrast, the SET MCPP universal and targeted exercise prescription was delivered by MMCs within the local council setting who had capacity to deliver the programme components, yet exercise prescription was still considered to be lacking HIIT. Future MCPP may benefit from additional training for those delivering the targeted exercise pathway, alongside monitoring of intervention delivery, and ensuring professionals integrate skills taught to enhance intervention fidelity [36]. It would be important that any quantitative evaluation focusing on objective functional and patient-reported outcome measures from the SET MCPP are understood in terms of the current perceived fidelity to the programme components.

*Reflexive monitoring* was evident throughout the SET MCPP with monthly working group meetings providing a vehicle to review progress and facilitate troubleshooting. Reflective analysis, real-time feedback and booster training sessions allowed many issues to be addressed, however, some outcome data capture remained problematic. Given the often-short timeframe for MCPP delivery, alongside lack of knowledge of, or changeable, treatment commencement dates presented as challenges for MMCs to collect Time 2 data. Minimising attrition rates of Time 2 data capture is paramount to enhance the evidence base for MCPP [25]. Development and implementation of the MCPP at SET received no additional funding, however a sustained funding model is required to further consolidate, scale-up and integrate pathways for system-wide implementation. Professionals in this study, like that of others [25, 37] recognised the need for dedicated funding which extends to sustaining AHP involvement [25], project management and performance data support [37]. Given the success of MCPP already evident [27], there is now a need to align funding to ensure equitable access of MCPPs to all cancer patients. The NPT components [20] has been useful to illuminate the mechanisms of feasibility and acceptability alongside key areas for refinement of the MCPP to maximise future implementation and to identify important considerations for others seeking to embark on implementation of a MCPP.

### Strengths & limitations

This study benefited from an exploration of a diverse range of patient and professionals’ perspectives. However, the Local Collaborations (CNSs) were potentially more likely to enrol patients considered as engagers onto MCPP and therefore this patient study sample may not be representative of a generic cancer population. During recruitment to this qualitative evaluation effort was directed in trying to engage patients who dropped out of MCPP, but this proved challenging depicted in the patient recruitment rate of less than 50%. It is possible that patients participating in this study are likely to have a positive bias towards MCPP. Further investigation is required to determine the barriers to participation.

## Conclusions

The MCPP at SET was deemed a feasible and acceptable tailored and personalised intervention; with visionary leadership, engaged and motivated interdisciplinary professionals, committed to improved patient outcomes being central to its success. Iterative, responsive troubleshooting throughout implementation contributed to embedding individualised elements of a MCPP. The MCPP could benefit from greater prescription of HIIT to maximise potential patient outcomes. Improved professional and patient knowledge of the benefits of MPCC could maximise referrals to MCPP and enhance patient outcomes.

## Supporting information

S1 File Topic guides. (DOCX)

S2 File Anonymised transcripts. (DOCX)

## References

[1] Hughes MJ, Hackney RJ, Lamb PJ, Wigmore SJ, Christopher Deans DA, Skipworth RJ. Prehabilitation before major abdominal surgery: a systematic review and meta-analysis. World J Surg 2019;43: 1661–68. doi: 10.1007/s00268-019-04950-y 30788536

[2] Durrand J., Singh S. J., and Danjoux G. Prehabilitation. Clin.Med. (Lond). 2019; 19, 458–464. doi: 10.7861/clinmed.2019-0257 31732585 PMC6899232

[3] Giles C, Cummins S. Prehabilitation before cancer treatment. BMJ. 2019; 366:I5120. doi: 10.1136/bmj.l5120 31413000

[4] Department of Health A cancer strategy for Northern Ireland 2022–2032. 2022; Belfast: Department of Health. https://www.health-ni.gov.uk/publications/cancer-strategy-northern-ireland-2022-2032

[5] Moore J, Merchant Z, Rowlinson K, McEwan K, Evison M, Faulkner G, et al. Implementing a system-wide cancer prehabilitation programme: the journey of greater Manchester’s ‘Prehab4cancer’. European Journal of Surgical Oncology. 2021; 1;47(3):524–32. doi: 10.1016/j.ejso.2020.04.042 32439265

[6] Silver JK, Baima J. Cancer prehabilitation: an opportunity to decrease treatment-related morbidity, increase cancer treatment options, and improve physical and psychological health outcomes. American Journal of Physical Medicine and Rehabilitation 2013; 92: 715–27. doi: 10.1097/PHM.0b013e31829b4afe 23756434

[7] Macleod M, Steele RJ, O’Carroll RE, Wells M, Campbell A, Sugden JA, et al. Feasibility study to assess the delivery of a lifestyle intervention (TreatWELL) for patients with colorectal cancer undergoing potentially curative treatment. BMJ open. 2018; Jun 1;8(6):e021117. doi: 10.1136/bmjopen-2017-021117 29880567 PMC6009630

[8] Grimmett C, Bradbury K, Dalton SO, Fecher-Jones I, Hoedjes M, Varkonyi-Sepp J, et al. The role of behavioral science in personalized multimodal prehabilitation in cancer. Frontiers in Psychology. 2021:261. doi: 10.3389/fpsyg.2021.634223 33664701 PMC7921482

[9] West MA, Loughney Lythogoe D, Barben CP, Sripadam R, Kemp GJ, Grocott MP, et al. Effect of prehbailitation on objectively measured physical fitness after neoadjuvant treatment in preoperative rectal cancer patients: a blinded interventional pilot study. Br J Anaesth 2014;114(2):244–51. 25274049 10.1093/bja/aeu318

[10] Vagvolgyi A, Rozgonyi Z, Kerti M, Agathou G, Vadasz P, Varga J. Effectiveness of pulmonary rehabilitation and correlations in between functional parameters, extent of thoracic surgery and severity of post-operative complications: randomised clinical trial. J Thorac Dis 2018;10(6):3519–31. 30069349 10.21037/jtd.2018.05.202 PMC6051851

[11] Vermillion SA, James A, Dorrell RD, Brubaker P, Mihalko SL, Hill AR, et al. Preoperative exercise therapy for gastrointestinal cancer patients: a systematic review. Syst Rev 2018;7:103. doi: 10.1186/s13643-018-0771-0 30041694 PMC6058356

[12] Faithfull S, Turner L, Poole K, Joy M, Manders R, Weprin J, et al. Prehabilitation for adults diagnosed with cancer: A systematic review of long-term physical function, nutrition and patient-reported outcomes. European journal of cancer care. 2019 Jul;28(4):e13023. doi: 10.1111/ecc.13023 30859650

[13] van Rooijen S, Carli F, Dalton S, Thomas G, Bojesen R, Le Guen M, et al. Multimodal prehabilitation in colorectal cancer patients to improve functional capacity and reduce postoperative complications: the first international randomized controlled trial for multimodal prehabilitation. BMC Cancer. 2019;19(1):1–11. doi: 10.1186/s12885-018-5232-6 30670009 PMC6341758

[14] Small S, Bingham SL. and Semple C. 2022 (In press) Standard operating procedure for multimodal cancer prehabilitation at SEHSCT

[15] Macmillan Cancer Support. Principles and guidance for prehabilitation within the management and support of people with cancer. England: Macmillan Cancer Support https://cdn.macmillan.org.uk/dfsmedia/1a6f23537f7f4519bb0cf14c45b2a629/1532-source/prehabilitation-for-people-with-cancer-tcm9-353994

[16] Greenhalgh T, Papoutsi C. Studying complexity in health services research: desperately seeking an overdue paradigm shift. BMC medicine. 2018 Dec;16(1):1–6. doi: 10.1186/s12916-018-1089-4 29921272 PMC6009054

[17] Murdoch J, Varley A, McCulloch J, Jones M, Thomas LB, Clark A, et al. Implementing supportive exercise interventions in the colorectal cancer care pathway: a process evaluation of the PREPARE-ABC randomised controlled trial. BMC Cancer. 2021 Dec;21(1):1–2. doi: 10.1186/s12885-021-08880-8 34688257 PMC8542291

[18] Sandelowski M. Whatever happened to qualitative description?. Research in nursing & health. 2000 Aug; 23(4):334–40. doi: 10.1002/1098-240x(200008)23:4&lt;334::aid-nur9&gt;3.0.co;2-g 10940958

[19] Sekhon M., Cartwright M. and Francis J.J. (2017) Acceptability of healthcare interventions: an overview of reviews and development of a theoretical framework. BMC Health Services Research, 17(88), 1–13. doi: 10.1186/s12913-017-2031-8 28126032 PMC5267473

[20] May C.R., Mair F., Finch T., MacFarlane A., Dowrick C., Treweek S., et al. Development of a theory of implementation and integration: Normalization Process Theory. Implementation Science, 2009; 4(1), 1–9. doi: 10.1186/1748-5908-4-29 19460163 PMC2693517

[21] Ritchie J. and Spencer L. Qualitative Data Analysis for Applied Policy Research. In: Bryman A. and Burgess R., Eds., Anal. Qual. Data, Routledge, London, 1994; 173–194.

[22] Dewberry LC, Wingrove LJ, Marsh MD, Glode AE, Schefter TE, Leong S, et al. Pilot prehabilitation program for patients with esophageal cancer during neoadjuvant therapy and surgery. J Surgical Res. 2019 Mar 1;235:66–72. doi: 10.1016/j.jss.2018.09.060 30691852

[23] Tweed TT, Sier MA, Van Bodegraven AA, Van Nie NC, Sipers WM, Boerma EJ, et al. Feasibility and Efficiency of the BEFORE (Better Exercise and Food, Better Recovery) Prehabilitation Program. Nutrients. 2021 Oct;13(10):3493. doi: 10.3390/nu13103493 34684494 PMC8538645

[24] Tang CY, Turczyniak M, Sayner A, Haines K, Butzkueven S, O’Connell HE. Adopting a collaborative approach in developing a prehabilitation program for patients with prostate cancer utilising experience-based co-design methodology. Supportive Care in Cancer. 2020 Nov;28(11):5195–202. doi: 10.1007/s00520-020-05341-z 32072326

[25] Provan D, McLean G, Moug SJ, Phillips I, Anderson AS. Prehabilitation services for people diagnosed with cancer in Scotland–Current practice, barriers and challenges to implementation. The Surgeon. 2021 Sep 15. doi: 10.1016/j.surge.2021.08.005 34535399

[26] Daun JT, Twomey R, Dort JC, Capozzi LC, Crump T, Francis GJ, et al. A Qualitative Study of Patient and Healthcare Provider Perspectives on Building Multiphasic Exercise Prehabilitation into the Surgical Care Pathway for Head and Neck Cancer. Current Oncology. 2022 Aug; 29(8):5942–54. doi: 10.3390/curroncol29080469 36005207 PMC9406549

[27] Ricketts WM, Bollard K, Streets E, Hutton K, Hornby C, Lau K. Feasibility of setting up a pre-operative optimisation ‘pre-hab’service for lung cancer surgery in the UK. Perioperative Medicine. 2020 Dec; 9(1):1–9. doi: 10.1186/s13741-020-00145-5 32426114 PMC7218588

[28] Brahmer JR, Lacchetti C, Schneider BJ, Atkins MB, Brassil KJ, Caterino JM, et al. Management of immune-related adverse events in patients treated with immune checkpoint inhibitor therapy: American Society of Clinical Oncology Clinical Practice Guideline. Journal of clinical oncology: official journal of the American Society of Clinical Oncology. 2018 Jun 10; 36(17):1714. doi: 10.1200/JCO.2017.77.6385 29442540 PMC6481621

[29] Bradley P, Merchant Z, Rowlinson-Groves K, Taylor M, Moore J, Evison M. Feasibility and outcomes of a real-world regional lung cancer prehabilitation programme in the UK. British Journal of Anaesthesia. 2022 Jul 13. doi: 10.1016/j.bja.2022.05.034 35840361 PMC9875904

[30] Bryson JM, Crosby BC, Stone MM. Designing and implementing cross-sector collaborations: Needed and challenging. Public administration review. 2015 Sep;75(5):647–63. doi: 10.1111/puar.12432

[31] Yardley L, Morrison L, Bradbury K, Muller I. The person-based approach to intervention development: application to digital health-related behavior change interventions. Journal of medical Internet research. 2015 Jan 30;17(1):e4055. doi: 10.2196/jmir.4055 25639757 PMC4327440

[32] Skivington K, Matthews L, Simpson SA, Craig P, Baird J, Blazeby JM, et al. A new framework for developing and evaluating complex interventions: update of Medical Research Council guidance. bmj. 2021 Sep 30;374. 10.1136/bmj.n2061 34593508 PMC8482308

[33] May CR, Albers B, Desveaux L, Finch TL, Gilbert A, Hillis A, et al. Translational framework for implementation evaluation and research: Protocol for a qualitative systematic review of studies informed by Normalization Process Theory (NPT)[version 1; peer review: 2 approved]. NIHR open research. 2022 Jun 13;2:41. doi: 10.3310/nihropenres.13269.1 35935672 PMC7613237

[34] Moore GF, Audrey S, Barker M, Bond L, Bonell C, Hardeman W, et al. Process evaluation of complex interventions: Medical Research Council guidance. BMJ. 2015 Mar 19;350. doi: 10.1136/bmj.h1258 25791983 PMC4366184

[35] Toomey E, Hardeman W, Hankonen N, Byrne M, McSharry J, Matvienko-Sikar K, et al. Focusing on fidelity: narrative review and recommendations for improving intervention fidelity within trials of health behaviour change interventions. Health Psychology and Behavioral Medicine. 2020 Jan 1;8(1):132–51. doi: 10.1080/21642850.2020.1738935 34040865 PMC8114368

[36] Horner SD. Best practices for improving intervention fidelity that every nurse should know. Journal for Specialists in Pediatric Nursing. 2012 Apr;17(2):171. doi: 10.1111/j.1744-6155.2012.00327.x 22463478 PMC3324280

[37] Tew GA, Bedford R, Carr E, Durrand JW, Gray J, Hackett R, et al. Community-based prehabilitation before elective major surgery: the PREP-WELL quality improvement project. BMJ open quality. 2020 Mar 1;9(1):e000898. doi: 10.1136/bmjoq-2019-000898 32213551 PMC7206908

