# Supplementary material for: A qualitative evaluation of a multi-modal cancer prehabilitation programme for colorectal, head and neck and lung cancers patients
Source: PLoS One. 2023 Oct 3;18(10):e0277589. doi: 10.1371/journal.pone.0277589 (PMC10547201; doi:10.1371/journal.pone.0277589)
Supplement: S1 File — (DOCX) [file pone.0277589.s001.docx]

1. **What did you know about cancer prehabilitation before the service started at SET?**
   1. Explore how perceptions have changed and why.
   2. Explore views of patient engagement before surgery?
2. **What is your role in the MCPP?**

Assessment/referral

Planning/cocreation

Did you receive any training? If so what, how and wen

On reflection, how could you have been better prepared for this new role?

1. **Experience of assessment and referral process** for the MCPP:
   1. Optimal time to introduce MCPP
   2. Did you encounter any challenges with screening, referral and consent process?
      1. Are there any components that aren’t necessary (screening tools [ECOG, Distress thermometer, Rockwood scale], social habits - smoking drinking, inclusion criteria, virtual component)
      2. Explore technical difficulties
   3. Helpful aspects to the screening, referral and consent process (online form, multiple referral integrated into one form)
   4. Explore perceptions of patient interest in MCPP
      1. Perceptions of referral levels
   5. Any resistance from patients for onward referral?
2. **Impact of COVID-19 had on MCPP service provision**
   1. Virtual delivery – facilitators and barriers
3. **Explore perceptions on professional development and practice as a consequence of being involved in the MCPP:**
   1. Knowledge and skills
   2. Learning shared with colleagues
   3. Discussions in team meetings
   4. Job satisfaction
4. Explore **areas for improvement to enhance sustainability of MCPP**:
   1. Potential areas for improvement?
   2. What would help with widespread implementation?
5. **Explore barriers for the long-term delivery of MCPP**
   1. Additional resources required? If so, what?

**Final comments**

**Close interview**
