# Supplementary material for: A qualitative evaluation of a multi-modal cancer prehabilitation programme for colorectal, head and neck and lung cancers patients
Source: PLoS One. 2023 Oct 3;18(10):e0277589. doi: 10.1371/journal.pone.0277589 (PMC10547201; doi:10.1371/journal.pone.0277589)
Supplement: S2 File — (DOCX) [file pone.0277589.s002.docx]

# P1

**SB**

So just thinking back to when this sort of before you’ve been involved in the cancer prehabilitation pilot, what did you know about cancer prehabilitation?

**P1**

Back then?

**SB**

**P1**

I had been to a conference that was run by the Royal Marsden and I heard a very impressive speaker he was an anaesthetist his name is John Moore, he has actually been instrumental in the setting up of the cancer prehabilitation in the Greater Manchester area and I could see form his data that there was a great merit in cancer prehabilitation, in getting patients into peak performance or optimised for the treatment that’s ahead, so that for me was something I thought working in Head & Neck Oncology we do have patients with significant comorbidities, we’re seeing an aging population and the stats were very impressive so I thought this has got great credence for us to emulate in some ways within our practice.

**SB**

And now that you’ve been involved, and seen that worked through do you still think that way, has your perception changed at all?

**CS**

I think even from there the evidence is ever growing and ever mounting, so that opportunity not only to encourage people to engage in physical activity, and I think even as well the whole science behind exercises becoming more to the fore, I had another recent presentation and had a read around, there is some thinking that it may become the fourth treatment in addition to radiotherapy, chemotherapy and surgery. Not only in that ability to help people get through their treatment and increase their resilience but also in the effectiveness of treatment and the rates of recurrence, so I’m certainly probably even more bought in than I was at that stage. Nonetheless I’m pretty cognisant of the challenges of trying to implement and deliver a multi-modal prehabilitation service within SET.

**SB**

And so you’re very bought into it there. Do you feel it is essential therefore to engage patients at that early stage or…?

**P1**

Absolutely. The way I would look at it every day could make a difference so earlier is best. Totally aware that there’s a lot going on where someone is hearing that news it can be devastating for some individuals that they’ve got cancer, but it is an opportunity we’ve been hearing about the teachable moment for a long time, so this is a prime opportunity to optimise and utilise that teachable moment. Also it’s an opportunity as well to give some control back to the patient and you know we have walked away, not walked away, seen the need to shift from paternalistic delivery of care to something that is very shared care approach and this is a prime opportunity to enable and engage people early in a shared care model of their treatment.

**SB**

You were saying there you touched on the challenges of delivering a multi-modal intervention. How well did you feel prepared in your role to do that?

**P1**

Well I’m probably a little bit different from some of the others that you may be interviewing in the sense that I was involved in the steering group, the cancer steering group within the trust and very quickly I was asked to lead that group so that certainly gave me the opportunity to be centre of that table along with other key stakeholders, so I think that gave me the further knowledge and insight and background as well, so I didn’t feel the need to go for additional training sessions because I had seen or heard from Zoe Merchant from Greater Manchester, been sitting round the table with the MacMillan cancer support prehabilitation leads nationally so I suppose I had that unique opportunity to be involved at that level from inception of the planning within SET and also let out on trying to gather some of the background information and work more closely with the Head & Neck Team also into what key components we felt we needed and how we could measure it and how we could deliver it, so it was certainly very embedded in the whole process. However there is a key acknowledgement that it is more often the Clinical Nurse Specialists who are introducing cancer prehabilitation to the patients and it is really important that the colleagues who are introducing this to patients are as bought in as the people in the should we say the steering group doing the planning and if there is a mismatch then we certainly need to address that, and we have had need to ensure that our colleagues have the information to enable them to engage proactively with patients as early as possible.

**SB**

So you’ve been very involved through the planning and delivery. What about the assessment and referral process, how well prepared did you feel for that?

**P1**

Again I sat on that taskforce and finishing group and for me the important component within that was some process that was not going to be onerous for the clinicians who were completing the screening component and making the onward referral, so it as kind of I was very fixed on the fact of having a one stop shop essentially so having to make copious referrals sometimes when we are seeing new patients you could have 30, 40, 50 minutes afterwards making referrals onto for example the MacMillan Health & Wellbeing Team, the Dietitians, Speech & Language Therapists, all separate forms so I was really keen to engage with the appropriate people within the trust to see if we could get a referral form that done both and I suppose the aspiration I had was that once you had done your screening that you were very easily able to just tick and select the components with the prehabilitation and personalise that and with one submit button automatic referrals would generate to the relevant people, so that was my vision so thankfully we’ve been able to action that which is one of the great wins in the system that we do have today.

The other thing that we are very aware of that we had our move more coordinators who sat, not within the trust, but within the council areas so we needed to ensure that the patients were happy with their information being sent and we also had to find a safe channel to be able to send the information on, so that’s why we had to ensure there was a consent process also captured within the referral form. We needed the referral form to do a lot of things essentially.

**SB**

Yes. And did you face any challenges with the screening referral and consent process? You said there that you needed to get them into one form and to link with the move more coordinators but were there any challenges you came up against?

**P1**

Yes I suppose as well, this was a pilot and we did want to have baseline data in addition to the demographic so we had to discuss and negotiate with our CNS teams as in to what would be the minimum data set. So it’s like starting from a point of ‘oh it would be wonderful to collect all these measurables’ but what is realistic in an everyday practice situation that is outside the support of a research project, so this was like planning and delivering in a real life context, which has merits but has challenges because it is unfunded, so one of the things that we had as well was as many drop down fields within the referral form as possible. We realised that there were a number of fields that were not being completed by colleagues as well, so we moved towards some additional training and also some mandatory fields. We were introducing some tools for screening of alcohol for example that some of the CNS colleagues weren’t so familiar with. Again we did speak to our colleagues in the substance misuse liaison team. There are options in relation to assessment from an alcohol perspective but we had to ensure it was a very simple tool, so they weren’t feeling all at sea, but again with some colleagues not being familiar with it we built in a link within the form which could take people to that link so they didn’t have to search in to how they would go about doing the screening for that particular tool. ECOG was something that CNSs were very familiar with so we didn’t have to have an explanation built into the screening form.

Another tool we were using was Rockwood Clinical Frailty again that was a new tool for lots of people, so again we built in the numbers from 0-9 and what each of them meant within that dropdown field, so trying to make it as user friendly without making it too complicated or too clunky to aid people understand what the screening tools were, but also ease of completion.

**SB**

Yes and as a clinician then and using some of those new tools, did you find they were all necessary?

**P1**

Yeah you could see the premise of them, yes. I suppose somebody might say but why are we doing the Rockwood Frailty Score? But there was a science behind it on the actual level the individual was at helped you denote whether somebody was appropriate for universal exercise, targeted exercise or specialist, so you might have looked at it and go why is that in there? But whenever you got more fuller comprehensive understanding of it you can see that was important to help you decide which pathway was appropriate for the individual so we were trying to have different pathways which then could be personalised to an individual’s needs, so that tailored approach so people could get the maximum benefit but also the most appropriate prehab delivery.

**SB**

And in terms of, so at the planning stage you would have discussed the best timing to introduce the cancer prehabilitation pilot. What was the thinking at the planning stage and did that change then in practice?

**P1**

I suppose we were really aware that sooner is best. There was no getting away from that. There was some tension with when was the most appropriate time to introduce prehab. Some colleagues felt it was too much at that first consultation. Someone is hearing they’ve got a cancer diagnosis, it’s not their priority at this stage, and that we should leave it for a week down the line. So we thought through that and then it was when most of these individuals are coming back to clinics a week later, they’re maybe coming back in a couple of days for their actual surgery or treatment, and some people it’s straight to surgery so we realised that we needed to optimise every day and every opportunity, so yes this was something that was new to the clinical teams but once we trialled it we realised it was working best.

**SB**

Ok so at that initial appointment then?

**P1**

Yes. At that first appointment

**SB**

And was that challenging? Did you find that challenging?

**P1**

Yes because it was a new way of working and at the first appointment you’ve got quite a lot of that you have to cover anyway and this seemed to be something else that you were now covering but then you realise that and the way that we have encouraged people to do it is introduce prehabilitation, there is a patient information sheet, there’s quite a thick pack with the home exercise programme, and just say to them ‘that’s not your priority today but the coordinators will be in touch in the next few days and they will help you work through that, but it was really important that prehabilitation was introduced to the individual in relation to their care and treatment, and how I would often introduce it would be to go we certainly have our part to do but there’s something that is equally important if not more important that you can be doing now, and these are the things. You know, and then give them a little bit of the rationale behind it. And the more you do it the more you realise this does work now.

**SB**

How did you feel patients engaged with the cancer prehabilitation?

**P1**

It’s like everything, you have a range of degrees of engagement. There are some people who have a very positive exercise towards exercise and it’s something that is familiar to them and it can be also a tool to help them cope with stress so it’s something that is very achievable. It’s not all about exercise, we do have in Head & Neck the one stop clinic so that’s a really good opportunity we can say, you know we’re going to make the appointment, you’re going to come back on Monday, whatever the date that might be, it’ll give you an opportunity to meet the Dietitian. We aimed for the Speech & Language Therapist, we had them for a little while as part of that one stop clinic, and the Nurse Specialist will have more time to talk you through your treatment and what that might mean for you and there will also be an opportunity if you wish to meet another patient who has had something similar. Again it’s all how you frame it and again if you’ve got your consultant colleague there in the room with you and they’re going ‘[HNC CNS NAME] or the other Head & Neck Nurse [HNC CNS NAME], and they’re going to tell you about you know how to get yourself prepared for treatment’ and they’re endorsing it, that again is a lot of kudos towards cancer prehabilitation and that’s how it happens in our practice.

**SB**

And were patients willing to be referred on?

**P1**

I think bar one or two most people have been, yeah. It’s a rarity that people don’t, and quite often people are in that situation it’s not another thing. For some people it can be, they’ve got some appointments, more scans to go to but for most it’s something they can be doing for themselves, which is helping them and I think if you can sell it in that way. And again we had elements of prehabilitation within our service beforehand so this was formalising some of it. From a smoking perspective we had an opt out, so anyone who was a smoker they had an automatic referral to the stop smoking team so it was formalising that pathway. Again given the condition we work with being head and neck cancer, there’s a significant proportion of our patients have alcohol related issues or they’re alcohol dependent, so that’s part of our initial assessment anyway and we work very closely with the substance misuse team, the alcohol liaison team and it’s not something that we’re uncomfortable with probing into that area of people’s lives and making recommendations and onward referrals, so those were well established anyway.

**SB**

You said that a few people opted not to be a part of the prehabilitation pilot. Did they give reasons?

**P1**

I think they just felt so overwhelmed and it was maybe then for those, ideally its for the first appointment then going back at another point in time. So you never really can take an emphatic one size fits all, you can have good processes in place and they are the ideals but that requirement to have some degree of flexibility in and around to meet patients’ needs.

**SB**

What impact do you think covid-19 has had on the cancer prehabilitation programme?

**P1**

Fabulous question! Yes, I suppose at the time that we were doing the planning we were in one of the lockdowns, can’t remember which one, so it meant that our move more coordinators had to deliver a virtual service instead of an in person or you know coming to a leisure centre, so that certainly had benefits and challenges. Some of our move more coordinator teams could do home visits and see people in their gardens outside, so they were creative. Other ones because they were employed with the council that was a no-go so yeah. And I suppose one of the biggest challenges was getting the functional outcomes performed. So for us we realised that our definite measurables were really very gappy at the first part but that was a reflection on the inability to have in person contact with people. However, you know there’s, and I did send a couple of papers onto our move more coordinators in our team there was some work that had come out of The States around virtual exercise programmes in cancer patients and how they were in relation to efficacy and they were essentially quite similar to in person approaches, so it was really encouraging our move more coordinators that there was still merit in this. And you know you can give advice in numerous ways. You don’t have to see a person face to face in person to talk to them about how they’re feeling about their cancer, that emotional aspect of things. You can actually provide nutritional support by telephone, by other platforms. Again we do know that cancer is more common in the aging population who are less IT aware or have the technical skills, again that’s a bit of a generalisation but for some individuals who didn’t have access to Zoom or Teams platform which could have been another way, for those that did have it seemed to work. But one of the other ways that again was being creative was WhatsApp. So again we thought about Teams we thought about Zoom platforms but essentially telephone and WhatsApp seemed to be slightly more accessible.

**SB**

Was there anything unique with covid-19 that actually there was opportunities, there were actual benefits to the programme because of the restrictions?

**P1**

Yes, the Move More Coordinators certainly seemed to have more time to be involved in the planning and able to get used to the different assessment points. So I think there was a unique opportunity to there was some more capacity within workloads for some of the people delivering the programme, so that was a unique selling point, and again it also gave us the opportunity to see what could be delivered at a time that wasn’t in person.

The other people that had more capacity that have been instrumental in the whole plan and setting up was the health development team. They had not been running the same amount of programmes and there was [HEALTH DEVELOPMENT TEAM MANAGER] who you did met yesterday and will be interviewing, he was more or less fulltime on the planning of this project for a number of weeks which was instrumental in pulling together the SOP, facilitating the task and finishing groups and doing a lot of the leg work in relation to coordinating back through with the Greater Manchester team and getting the resources and there was another member of their team also involved in getting the patient resources pulled together and then having the available resources for the move more coordinators and the CNSs. We talked about the Rockwood tools and those tools and they were also printed out and laminated so they were accessible in the clinical area, so that very fact that we were in a pandemic and lockdown really enabled workload capacity for some individuals to really get focused on this.

**SB**

You had mentioned there that was opportunities to share some learning with the move more coordinators and papers that you’d come across around sort of telehealth methods, that’s not even the right word… anyway, the advantages. You had opportunity to do that. Were there any other benefits to professional development and practice either for yourself or for those that you were working with?

**P1**

Oh absolutely and [HEALTH DEVELOPMENT MANAGER NAME], [ADEPT FELLOWSHIP PARTNER] and I had booster sessions with the move more coordinators so absolutely and in addition to that with the move more coordinators we identified what their training needs were, so that was an important component as well. So there was a session set up in relation to nutrition because they were delivering universal nutrition. There were sessions set up from an exercise point of view for the colorectal patients with the physiotherapists in relation to core stability which is important for patients having that surgery. For patients having head and neck surgery, our oncology physio met with the move more coordinators and showed them neck and shoulder exercises and the importance of that so that patients could be started to do those in advance, so it was trying to ensure that those delivering it had the required knowledge and skills but we did need to go back and have booster sessions, because another thing that was maybe not so commonplace for the move more coordinators was the delivery of exercise prescriptions. There seemed to be as part of our monthly team meetings whenever we were drilling down into it and how it was going and what needed to be delivered, it seemed to be more motivational encouragement to engage with exercise as opposed to a prescriptive exercise plan, so and then as you would have heard yesterday we are tweaking the assessment form for the Move More Coordinators so they actually have to denote what the exercise plan is, and that again is a cognitive tool in there so that every time the move more coordinator is with a patient that they have that sense that you know at the end of my first assessment the reason for this is to come up with an exercise plan and so that having to actually put it down and send it through to us will hopefully be another motivational factor to be a little bit more precise in what that action is required from the individual.

**SB**

And for you personally in terms of your professional development and practice, what do you feel you’ve gained from being involved in this project?

**P1**

I suppose I would say for me it’s it was more in and around project management. As you could see yesterday, there were so many people and so many components and that ability to be able to work alongside people that you hadn’t ever worked alongside before, understanding that partnership working is the best way forward and you know just utilising a number of people who have a range of skill sets and bringing them together. So for me one of the big things was yes project management and also the opportunity to share this at Department of Health level so it’s been recognised that the SET pilot there has been a wealth of learning there.

The other thing I would say is I was shoehorning this into a fulltime plus job, so most of the learning is that and I suppose that would have gone as part of the cancer charities bid that there needs to be a project manager in that role and that’s going to be an 8A for three days a week, moving forward for the other trust areas

**SB**

And that’s good learning from this project

**P1**

Yes absolutely, and that we need also to have some time built in for data management too, so that’s built in to the roll out as part of the MacMillan cancer charities

**SB**

So you’ve touched on a few things there but what improvements do you think could enhance the sustainability of the cancer prehabilitation programme? You mentioned there about data management and a coordinator?

**P1**

Totally, those are two key things absolutely. And I suppose that was the learning that we did have, and again the importance of having those booster sessions with the people that are delivering the service so that’s really important.

The other thing that is really important as well is having working group meetings like we did have yesterday. Having those continued and sustained on a regular basis and for me is never make assumptions that those delivering a service are actually delivering. It is important just to check up and make sure all the strands are still pulling together, and even yesterday you got the sense that there seemed to be some teams that for some reason had a reduction in referrals, so it was like let’s touch base and try and understand that. So there needs to be still that sense of ongoing leadership and input.

**SB**

What do you think might be some of the barriers for long term delivery of this programme across the trusts?

**P1**

Yeah I would say the capacity of the move more coordinators. So what we have selected is three tumour groups, certainly one of the larger tumour groups is colorectal and that’s going to be identified across nonetheless, that if we want to roll out to all patients having equitable opportunity to engage with cancer prehabilitation, we have a growing caseload of cancer patients and there will certainly be capacity issues with the move more coordinators, so that’s certainly one. And again a lot of this is unfunded work for our AHP colleagues, so again we have a keen desire and see a discreet need for a Speech & Language Therapist to be a part of Head & Neck cancer prehabilitation but due to workload pressures again that’s not currently sustainable, so we do need some dedicated and sustained funding to enable this to continue. You know cancer recovery plan is certainly sitting within it, but that hasn’t been delivered upon so that to me is something that is absolutely instrumental. There is also vulnerability that the person collecting the data I suppose it goes for any project or any component is down to one or two people and if they move on or they take sick then that creates another vulnerability yes.

**SB**

Yes it can be quite dependent on few people

**P1**

And I think because it’s taking a multi-modal approach and you’ve got exercise so there needs to be delivery across three levels, so that’s not only the move more coordinators it’s the you’ve got your Move More Coordinators, you’ve got your dietetic support and you’ve got your Specialist Dietitian so again that’s a range of three people, three entities and again emotional support is a little more complex again. So you’ve got your move more coordinators, you’ve got referral on to the MacMillan Health & Wellbeing team for counselling, you’ve got your Assistant Psychology, and then you’ve got your Cancer Specialists, and then you’ve got your smoking and you’ve got your alcohol so you can start to see the complexities in a system wide approach.

**SB**

And it needs to have everyone at the table

**P1**

Absolutely. And then we’ve got a couple of bespoke things in there like the Pelvic Floor Physio, then you’ve got your person who is collating the data as well. You’ve someone else who’s sending out the patient satisfaction surveys so there’s another, there’s other spokes there as well.

**SB**

Ok that concludes our interview. Have you any other comments you want to make?

**P1**

Nothing that, yeah it’s just like anything there needs to be dedication to the patient and don’t make assumptions on things I suppose from a project management point of view. But great to see that we’ve been able to set up a service without funding and there is some rich learning from that.

**SB**

Incredible feat certainly. So thank you for participating in the interview.

# P2

**SB**

Okay, okay. Emm so I just have a series of sort of themes I want to kind of explore with you [PARTICIPANT NAME] if that's okay.

**P2**

Yeah.

**SB**

First of all, can you tell me about your professional background and delivering physical activity interventions? What does that look like to date?

**P2**

Yeah, so I, my degree was in sport and then when I first came out of uni, I've been coaching the whole way through uni, private coaching for well over 10 years now, and but more in community setting. So I worked for Sport NI, and my background was really working with sort of underrepresented groups in sport. So your likes of people with disabilities, older people, and women and girls are the three main target groups. So I would have delivered a lot of like interventions, sporting in the community programmes to those, those three main target groups, working in a wide range from schools, to nursing homes, you know, community groups, stuff like that. And then obviously, I started with MacMillan, I was the first coordinator in post in 2016. And so the move really, from sport in the community, you know, to sport and health was, was a big change for me. But then there was, you know, the the MacMillan programme didn't run in Northern Ireland before, so there was nothing really like this you know, specifically targeting cancer patients. So obviously, we have like cardiac rehab and stuff like that. There's GP referrals. So those kind of exercise interventions, you know, run in leisure centres, run within councils, and I wasn't involved in sort of GP referral beforehand.

**SB**

Okay,

**P2**

Yeah, I was the first coordinator then in post in 2016. So it was a bit of a, it was a great learning curve, steep learning curve, but we obviously had move more running in northern or in sorry, in Scotland and England at the time. So a lot of my learning then was going to England to shadow them, see how they run their programs. You know, again, it ran quite differently. You know, a lot of the move more in England was more hospital based, rather than community based. And so myself and the manager of MacMillan at the time at Derwent College, we did a lot of shadowing of different sort of sites to see what the best sort of practice would be if this were to implement in Northern Ireland. And then we came back and really got it up and running I suppose the back end 2016, and then from there just kind of just grew, and that's been sort of my main focus for the last five, six years now is sort of a specific activity and cancer patients.

**SB**

Okay. Fabulous. And what about an patients with other complex illnesses? Have you much experience in that regard?

**P2**

Yeah, like, I think I think we learnt that pretty early on, like, a good majority of people diagnosed with cancer weren't just, you know, cancer. And to be honest, that was a big, one of our biggest challenges at the start, because we would get referrals in and be honest, the cancer was maybe the easiest part of the referral, because, you know, they're maybe obese and they have diabetes, maybe had a heart condition had a stroke, previous heart attack. And, and all those conditions would make conditions we worked with myself and probably the majority of coordinators would have worked with, in that sort of GP referral role. So within local council there is, obviously people get diagnosed conditions like sort of obesity sort of high BMI so basically diabetes, heart conditions, would be they would have the opportunity to be referred to sort of the what's called the GP referral scheme, which is now called the healthway scheme, and it's changing again, so they get access to the gym for sort of 12 free weeks, so I would have got a lot of work with them, in Lisburn council, the council I was working with beforehand. And and that's been obviously get exposed to a lot of different complex conditions. And as you said, then working with cancer patients, sometimes the cancer was the easiest part of the diagnosis, and it still is, you know, even now with the prehab you know, you might get a referral in and, you know, the contraindications are this long, you know, and the cancers, you know, are I was out with a lady last week, and she was four months post her up and stuff and she was like I'm fine, 100%. She had a colorectal diagnosis, but she's waiting on a hip replacement. And like, that's more life limiting to her you know, so she can hardly walk and she's like, No, no issues with the surgery went brilliant it's all great, but can't you know, can't do anything because her hip so yeah, we find that quite a bit, that there's a lot of you know, comorbidities

**SB**

Yes, yes. You have quite a lot of experience there you're bringing into the project. And before the cancer prehab service commenced what was your understanding of the move more coordinators role in delivering cancer prehabilitation?

**P2**

Yeah, so when we had originally set up move more in Northern Ireland. It was our original pathway was we were accepting referrals from the whole spectrum. So from point of diagnosis right to end of life. And we've always had that sort of open door policy. Now traditionally, with move more, we would have seen that was always kind of those sort of post treatment, probably in treatment and post treatment, post treatment would have been our biggest. And it was all like breast was our biggest, you know, referrer in. And it tends to be that across England as well, you know, it tends to be a lot of things, you know, your breast and maybe your prostate are probably on there. Emm with referrals in so we always accepted referrals from point of diagnosis, now it was never as formal as what the prehab service is at the minute. So before this prehab kicked off sort of last March, myself and [HNC CNS] and one of the other coordinators, [MMC NAME], who is now gone, and had had wheels in motion for a bit of a, like a long time really actually the head and neck team because they were very keen to get something up and running. And I'd met with them just on my own sort of one to one basis, saying we take referrals in look this is some of the people we've worked with, with prehab. A lot of our rehab referrals have come in quite sporadically, but would have been mostly like kind of colorectal patients, patients who were maybe overweight, who needed to lose weight for surgery, and that was where we were seeing like, across the board, kind of we were seeing good results, because we've maybe had them for six weeks or 12 weeks. And the anaesthetist was like they lose a stone or whatever it is.

**SB**

Okay.

**P2**

And so going in [HNC CNS] was asking yes, we do prehab, you know, send them on there's a coordinator in every council area, there's access there and that you know, that was maybe maybe a year before COVID, or maybe a year and a half before COVID. But referrals were low like, you know, and head and neck, it wasn't the best one to sort of start with because obviously, they've so many complex needs. And but yeah, the wheels were in motion then. And then sort of just before Christmas of 2020, we had a bit more of a structure. You know, we had this, we had the lung team, the colorectal team and sort of a head and neck team in place myself and [MMC NAME] the other coordinators, we've had a few meetings and we're like, yes, right, we're gonna run this, it was all meant to be in person, it was all good to start the Easter of 2020 in Ards hospital, you know, we had a facility set up with the physio onboard, smoking cessation and then COVID hit. So at that point, you know, we were going to run it like it's sort of a 2hr clinic if my memory's right? And myself and [NAME] the other move more coordinator would have been there to facilitate the physical activity. So the you know the referrals would have come in. There is a prehab service that runs up in the Northern Trust, and it was only for maybe it was only for lung patients. But the move more coordinator up there, [MMC NAME], he would have went in and done like a little 20 minute exercise, we were going to kind of adopt that sort of, you know, that same sort of theme that we would go in and do a bit of exercise. But we will be there for like the patient will maybe come for two weeks or three weeks before their surgery, and we would be there every week. So it was sort of drip feeding them exercises stuff to go home and do then come back with this next week. And we would take more structured stuff, that was always the plan. And then covid hit obviously. So that went out the window, everything went on the back burner, until we could actually get up and running then and then it was like like, let's deliver this online or over the phone. And so like our input from the start of the south eastern trust pilot has been pretty good, you know, we were involved sort of right from the infancy we were sort of brought in and the SOP like the document that was developed, you know, was basically copied from the Manchester model, which I'm sure you've heard a lot about you know, but again when when I first seen it, I know partially I was kind of like, like the show many difference differences here in terms of like they had loads of money, they didn't have COVID. But even the type of exercises and stuff. You know, we had, we had quite a few questions about but it was really good for, you know, between [HEALTH DEV TEAM MANAGER] and [HNC CNS] and the team. We were involved and, you know, our points were listened to and, you know, it's again, trial and error getting going too, but we were definitely involved from the start, which I think was good for us to make the teams as well and, you know, get our input into shaping the service.

**SB**

Yeah, yeah. So just from what you understood the move more coordinators role to be before the project kicked off to what was, was there much difference?

**P2**

I say I say about the exercise, I suppose, you know, in terms of sort of there's there's sort of three different targeted pathways and virtual universe or the universal specialist targeted sort of pathways. I know like we have great discussion around sort of providing sort of the emotional support as well as move more coordinators and the nutritional support on that sort of basic level. Again, you know, we do that as move more coordinators with our clients already and it....

**SB**

Okay

**P2**

I wouldn't say it's very, I wouldn't say it's very in depth on any level, you know what we're not nutritionally trained and I suppose like any physical activity coordinator, somebody comes with looking nutritional in depth advice, you know, we refer them on because that's not our forte, we're not trained in that. But we do have basic knowledge and understanding. And on the emotional side of things as well, like, you know, we've been doing it for years and providing that, but again, we're not psychologists or anything like that, or vice versa, again we referral quite a bit for our clients. So our understanding is that we will be providing all of that. And in our eyes, that's kind of what move more is. That's what we do, you know, and so it wouldn't be anything much more different. And to be honest, it hasn't been, really, I suppose that our difference is, we're now getting the patient at diagnosis. So basically, they're been given bad news, they're getting their red flag appointment, and then we're contacting them within a couple of days, so their mind frame is different. So we're so used to people post treatment being like, oh, you know, I've been through this have had surgery have had my chemo, have radio what do we do now, and at that point, move more is great, because it's like come to our classes, you know, back into just to get socialized and building confidence. Whereas with the prehab, it's like, all I just been given this life changing news, you know, what's going to happen to me? What if this, what if that, you know, so emotionally, it's different to support them, and those in that sort of early intervention point. And I suppose we didn't really know, until we started how that would look. And again, every patient is different. So some patients won't need as much emotional support, some other ones will need a bit more, you know, nutritional diet support because they're maybe colorectal patients and so on. But, like, I think our links with the teams like the girls, the CNSs, and stuff has been great, because if we do, if a patient comes to us and asks, you know, what am I supposed to be doing here? And what are these strengths for, we can just refer back and you know, the girls are great to pick that up. So I don't feel like we feel like we're lost at all. Well, I certainly don't, you know.

**SB**

yeah, that that's and that that is fab. And there's some things that you've touched on, we're going to come back to just as we move through. So you had said there [PARTICIPANT NAME] that you felt emm that you were very involved in the planning and the co creation programme, and it was even something that you were doing, you know, previous to this being a programme as such. Did you get any and you went and visited the various teams, you mentioned that as well. Was there any specific training you received to help you do this as part of those visits? Or?

**P2**

Yeah, we did do my my memory, my memory's not great. But we did do before we kicked off we did. And we had sort of online training with a dietitian. And so sort of we did, like, there was a basic training course that we were on it was the kind of eat well eat well plate stuff that we did before. But we did have good contact now [HEALTH DEVELOPMENT TEAM MANAGER] probably the person to ask in terms of people, but there was a senior sort of nutritionist came on or dietitian came on. And, you know, she was quite good sent us quite a lot of resources. And I've never had a patient that has maybe required that level of support. But I know this sort of the resources and the documents are there, and we have you know, a link to her if we needed it, we also did some training with I suppose like the abuse team. So I know the alcohol and smoking cessation team are involved in the prehab as well, obviously, people get referred there, but there was a lead in the trust, they had a couple of zoom sessions with us as well of like, kind of, I would say it was more like motivational interview training and stuff like that, which again, we've you know, we've done that with, with Macmillan, you know, our training with Macmillan had been for move more, you know, as a coordinator we had to do level one motivational interview training, and then we had to do like our emotional resilience training, and stuff like that. So as coordinators, we kind of all had a basis already in sage and thyme and stuff like that in place. So we didn't really necessarily need to revisit that before starting the prehab pilot. And but we do have a list. I'm sure I have it somewhere. [INTERVIEWER NAME], if you need me to share it with you, but we do sort of have a list of training that we we would do you know. And I suppose when everyone sort of starts with MacMillan, you kinda, they have an extensive list of of training. And you can avail of as all coordinators, we've done kind of them all and there's always refresher courses if you want and we have an online portal, and that you can go on and you know, grievance training and stuff like that and how to shape a difficult conversation. That sort of training can be ongoing, you know, we've accessed that all the time.

**SB**

Yeah. It sounds like you felt very well prepared then for this programme. Is there anything you think you could have that could be provided that would have been helped you more.

**P2**

We did also get a wee training day, like a wee refresh your training day, which was quite good in terms of sort of, because at the minute with the prehab, we're taking sort of, like physical data. So we're taking like height, weight, blood pressure, rep, strength and stuff like that. So we one of the physios from hospital actually met us in Lisburn one day, in the committee centre and we did like a wee refresher day about taking blood pressure and stuff, which was great, because as again, when you move away from sort of GP referral, and taking and capturing that data into move more, we never really captured data. So we wanted move more to look more look and feel more like user friendly community friendly in terms of we didn't want to get referrals in and patients to be stripped down and like you know, us do their weight, or their BMI, and the majority of them were probably going to be in the obese category, you know, they've just gone through this terrible experience, and then us saying well, actually, you're overweight, and you need to do this and you need to do that. So we never captured data, you know, it was more about the social and coming in and building confidence. And so that behaviour change model is what we sort of employed with move more. So it was great to get that refresher, in terms of sort of the hands on practical side, and I think, like, as the prehab now is going to go regional, I think all the coordinators will need that. Because, you know, it's not something that we do every day, you know, you don't do that every day, I know that we've been doing it a wee while it's grand, and like, you know, the blood pressure machine all it's a it's easy, but I think, you know, we, we all need that sort of refresher. In terms of training, I don't, it was definitely dull, I don't start in the prehab. Because we had to build it up so much, you know, and I remember, like, I got the first referral coming in, and the girls were like, you know, we had like a little WhatsApp group, but it was like, right I have to ring this guy, you know, he's just got diagnosed and, you know, um, because of covid as well, like we hadn't had the same contact, even with our own clients. You know, we have all big groups of clients that have been with us for a couple years come to our classes go to all our social events. So it was it was very daunting starting it and I know, a few of the coordinators, now who are going to be coming on board regionally are feeling that, you know, even though I know they'll be grand, because it's what we do every day, but it is just that initial it's a different sort of format, I have to meet them have to do their physical data have to do two time points, and then have to follow up. And but no, I don't think there was any training that we needed that we didn't get, I think if there was anything that we did, sort of, I know, my experience is maybe a bit different. When I talk to other coordinators, they might say something different, because I know [MMC NAME] and some of them had a few more intense client like patients, you know, who had a lot of complex needs with maybe alcohol and mental health issues and stuff like that. So they might have a different view. But for me, [INTERVIEWER NAME] like I feel that I've been okay. And if there was anything I know, I can go back to the you know, we have a steering group call, you've been on them and raise it at that meeting you know if there was anything I needed.

**SB**

and that's good you know that you felt, you know, supported as part of the roll out. So the programme's been emm being delivered since March there nearly a year. Has the programme evolved in this timeframe? From what it started out as?

**P2**

Yeah. Yeah, definitely. 100%, you know, obviously started in March last year. No. Yeah. So COVID was obviously still playing a big part, we were in lockdown, or some sort of restrictions anyway. So we weren't seeing, you know, I know, hospitals, were starting to come back to sort of seeing red flags we weren't and obviously we are all employed by councils. So we had different, you know, procedures to follow than the hospitals at trust base. So we started the programme, and it was online. So it was zoom calls or team calls. And that created a lot of barriers for a lot of people. Because, you know, again, majority of our referrals do tend to be older people who maybe don't have access don't know how to use online. And so that meant it went over the phone, which was fine for a lot of them. Because we do have good resources, you know, we have a sort of a resource pack that every patient will get at diagnosis. You know, it has sort of like a really thick home exercise guide. So over the phone was grand because we could sort of talk them through the exercises, but we couldn't see them. So we couldn't do collect any data. You know, we were very weary of doing sort of sit to stand tests and stuff with obviously somebody on the end of the phone, we can't see them. We don't know if there's anybody else there. Yeah, it happens. Yeah, obviously, you know, you're not there with them. And so it's definitely evolved then you know, coming into sort of the summer of last year, things lifted a wee bit and we were allowed to do things sort of face to face. And we also started like outside, like all our things of all our move more programmes everything went outside, outside walking groups, outside classes, and it was outside meeting referrals then. And again, it's evolved differently for different coordinators so my, my role I've always kind of done house visits with with patients, it's just something I employed. My area is really different as well, so I'm not actually employed directly by the council in Ards and North Down, itself, like, half of our facilities are ran by council and half our leisure facilities are run by a private organization, called Circo or northern community leisure trust, so that's my employer, now we do work together and also they fund part of my role. But my roles just been a bit more flexible in terms of I've been allowed to conduct house visits if I felt like, you know I wanted to and it's something I've always done. So when the prehab sort of went back face to face, I find it a lot easier in terms of engaging with it with the patients, so I would be going to their house or their gardens at the start and doing it face to face and I was getting really good uptake, whereas I know the other coordinators when you get chatting to them, you know, they were actively asking their referrals to come into their centre, you know, to meet them to do the data and stuff. And a lot of people obviously were quite frightened, didn't want to go into leisure centres, which was understandable, and that's sort of, that's a theme that we're still seeing at the moment.

**SB**

Okay.

**P2**

So I definitely seen a good evolvement of mine, because obviously, uptake was quite good because I was going to the patient. And, and then now that's, I just carried that on, you know, and now that just happens in a patient's home. So I think my experience has been slightly different than the other girls, because I feel like it has been a bit better, it's, you know, broke down that barrier of, especially some of the patients who are getting a bit like a two week window, and to be ringing them and you'd be like, you know, can book an appointment. And like, they're, they're busy, because, you know, for a lot of people, it's like, oh, I need to go see such a such a need to organize, you know, they've maybe never been second their life to get this diagnosis. And they think it’lI need to see the Solicitor sort the will out, I need to go and do this. And these are the conversations we were having that we've never even never really had before. And I think it helped that I was going to them, it's sort of helped with engagement it broke down that barrier, of them thinking oh i need to go to the leisure centre that day for different appointments, and then they want to see me in 4 days before going for surgery again, when realistically, if they had a short time frame that we knew their data wasn't going to change that much and it's an extra stress on them, you know, saying can you come on a Monday, but your surgery is next Tuesday? Can you come back in next Monday? So me going to them I think definitely has helped with my sort of my sort of engagement over it.

**SB**

Yeah, in terms of the actual programme itself emmm were there any changes, like for example, in the terms of in terms of exercise prescription, or nutritional advice or emotional support is there any change from March?

**P2**

Yeah, and I think, you know, at the start of the SOP, you know, a lot of the conversations as I've already said there was a rolling sort of exercise and what Manchester were doing and they were doing these HIIT sessions three times a week, and again, I would have loved to went to Manchester to see that because I was very adamant that I didn't believe that they were doing that I still really don't believe that they're doing that. Because, you know, people were batting this turn around and all these meetings that we were attending. I was like there's no like, I very hard to believe that there's these cancer patients, they're just getting a diagnosis who'd maybe never been fit or active and or maybe in years who are doing 3 sessions a week of high intensity hitt training. You know, for us as exercise professionals, you know, HIIT training is very, you know, targeted at you know, you're working on a certain percentage of your your max heart rate, you know, you're getting a certain ratio rest, work to rest ratio, it has to be within certain zones. So it's not, you know, as I was saying, you know, people are watching the body coach and thinking, oh, yeah, like HIIT session, that's, you know, you do 30 seconds on 30 seconds off. But there's no way you're putting somebody with maybe a heart condition or you know, who's obese, bad knees, hips, into a HIIT session and even 30 seconds on 30 seconds off is so general, you know, so I didn't believe it and I'm still very sceptical of it. But there was people on the call adamant that it had to be HIIT session three times a week, and I knew from the offset, but like, I've had one guy, one man who was he was a cyclist, and he's very fit guy who was probably out of maybe 40 referrals I've had, he's the only one that would be appropriate to do HIIT training with right? At that his pain was maybe like a seven, eight out of 10. So he wouldn't have been able to do it post-surgery or pre surgery, sorry, in prehab. Emm so that definitely evolved. I was very much you know, like based on the as we always do it has to be patient centred has to be what they're able to do. You know, you're ringing ladies who are maybe 70 or 80 who maybe are bed bound or maybe go out once a week to do their shopping, never been in a gym in their life, you know, so it had to be home based kind of functional movements that they were used to doing you know, sit to stand basically squats but telling them it's sit to stand, bit of resistance band work, and sort of and more interval training abut obviously not using those sort of terms with with the patients.

**SB**

Yeah

**P2**

definitely evolved more into what the patient could manage, you know, and if it was walking, if they were walkers great, you know, keep that up, keep the heart and lungs... But then we sort of focused it a wee bit more so our patients, so our colorectal patients then would have got a lot more targeted, sort of prescribed core exercises. So we driven them with the physio we had a few calls with the physio before we sort of kicked off in terms of what we should be focusing on, head and neck patients, obviously, then we're doing a lot of like shoulder work, neck stretches and stuff, because obviously that's where their surgery would be and post surgery then that's what they would be working on, and then our lung patients was more kind of aerobics sort of heart and lungs sort of aerobic work. So we had that sort of discussions beforehand. So each of the tumour sites and would get a wee bit more prescribed work, you know, what if I had, so if I have somebody for two weeks, who's colorectal, who are generally pretty fit, like, I'm not going to have them doing any upper body work, it's all going to be core work, they've such a short window, you know, they're not going to, they're not gonna make massive changes in two weeks, but they're going to make some progress if you can target it. That definitely became a wee bit more focused as sort of the weeks went on, and the referrals came in.

**SB**

So sorry, just the sorry to interrupt you there. So when the model started, was the thinking HIIT sessions did that or what it was? and then you moved away from that?

**P2**

I would say people, maybe on some of those calls, still saying that is the model, you know, that it's HIIT sessions and they should be doing them three times a week, but we've reported back on numerous times that they're not doing HIIT sessions three times a week, you know, the prescription will change in terms of if I've got somebody for, again it depends on the length of time you have them for. Say you have somebody for four weeks, six weeks, you know, the first week or two weeks it'll be, they'll be doing, you know, a general circuit or for a group of exercises for maybe every other day. And then as they come closer to the surgery they maybe do it every day, if they're up to it, if they're feeling fine there's no soreness, there's no progression. And that's something now we're trying to capture, SB in terms of beforehand, we've never we haven't captured that, you know, that data, what we were prescribing, we're having a new referral form, or a new sort of update form coming out from [PERFORMANCE MANAGER NAME] now hopefully in the next sort of couple of weeks, and on that there's going to be a little box to capture sort of the prescription of what we are, you know, providing because we are reporting back like, they're not doing HIIT sessions, they're doing X, Y and Z, they're maybe walking three times a week, or some of them are still playing golf or, you know, whatever it is they maybe go to aerobics or swim, and then we'll just sort of put in then what we are we're prescribing them sort of, so we've got more into actual description, rather than sort of oh they have to be doing three HIIT sessions a week.

**SB**

Okay and you have already mentioned that you do give some nutritional advice and emotional support has that changed from the start of the programme, or has it, it was as planned?

**P2**

Yeah, I would say it's pretty, pretty similar. I, I don't tend to give ... Because they've just been given sort of bad news. And a lot of them will, you know, that when they're in the hospitals in the clinic and stuff, they'll get speaking to, you know, their, their nurse, you know, stuff straight away so they can maybe get those questions answered. I don't feel like I overly had to do any nutritional.

**SB**

Okay.

**P2**

kind of advice, you know, again, maybe your colorectal patients who obviously have to come off, you know, sort of, you know, their diet sort of has to change slightly before they go into hospital they have to take some drinks and stuff like that. And an emotional support. I know, I would say emotional support and nutritional support have been pretty constant. You know the same, it hasn't really I don't think it's really changed. Again, it's very patient centred, you know, every patient is different, you know and it'll depend on that individual, and what sort of support they need, but I don't think there's been any massive change. I don't think we're not covering anything you know that they maybe need.

**SB**

Yeah, fab you had mentioned there some barriers with regards to COVID and some you've also mentioned the flexibility you were afforded in terms of your role and how that, you know, maximized your engagement with with people. Were there any other sort of facilitators or barriers that COVID you know, presented, any opportunities actually that COVID presented, or were there other things that it stopped that had an impact in the programme, do you think?

**P2**

I definitely think it has had an impact in terms of like the difference that we can see in the uptake from face to face rather than online, you know, yes, it obviously provided opportunities for maybe for us in terms of time, you know, like if you're, you're doing an online call, you know, I don't have to drive down Ards Peninsula, I can just do it online, you know, so it saved our time a wee bit but I think it probably was outweighed by the fact that like people didn't use it or didn't really know how to use it or didn't have the support in the house to use it, I think I do think the service would have been completely different obviously if covid didn’t exist but it’s something we did run with and we do have that resource now that it’s there if anything ever happens and we do need to go back in or there is a patient who gets referred who I know Laura has a guy in last week who is a long distance lorry driver who’s going to Sweden this week so she’s never going to see this guy you know, but if he has access to online then there’s that opportunity there to engage with him and you know support him. yeah I don’t know if there was anything else that covid really

**SB**

It sounds like you’ve been quite creative around the whole sort of restrictions and moving trying to find ways of seeing patients again so…

**P2**

Yeah so I think we had to be and even with move more we straight away created platforms, YouTube channels and zoom classes and stuff like that just to keep that sort of connection with people, I think that was really important, but as soon as we were able to go outside, our clients were busting to go outside or as soon as we could go back into leisure centres into a sort of small group they all wanted to go back in you know, and the social aspect of it you know is something that they really lacked and I think in our initial plans for prehab it was something that we wanted to maybe explore in terms of yes prehab is very individual in terms of everyone has different dates and so on but if there was an element of shared, say colorectal patients together for small group training, that would have been something that we really wanted to do from the outset.

Now we haven’t tried that just yet with the nature of covid, it’s put a lot of people off, you know we have regular clients who still haven’t come back to classes, so especially people then with prehab. Because again they have to do a PCR test before they go into hospital, they have to isolate before their date, that then restricted our time with them too because they only have two weeks and you’re only seeing them once then the following week they were maybe doing their PCR test 2-3 days before hospital, and they were isolating so again we couldn’t have contact with them face to face, so that was challenging too, and we found then that a lot of patients then I was maybe ringing them for a follow up and they were like oh I’m just lying in ward 5c here in the Ulster Hospital I had my surgery yesterday, and I’m like oh right ok that’s fine. We didn’t know that and we didn’t have that sort of contact. We don’t have access obviously to the Trust health systems, but we then raised it at one of the meetings and the nurses were saying themselves there were people we don’t even know go for surgery straight away you know just the nature of what covid has done in terms of scheduling surgeries, you know beforehand they would have had an idea maybe 3-4 weeks out what the schedule would look like, but at the minute it was basically if there was a free theatre and free nurses the consultants were just going and people were being brought in from a few days before.

So that was challenging enough and that sort of settled before Christmas we sort of found that again, obviously with omicron the variant and things sort of picked up a bit and people being cautious again and not wanting to see us maybe or you know, so people are fearful and I know the other coordinators will say that too, especially as they were asking people to come into the centre so they were maybe getting to see them once, but then they were going for surgery so they were oh we’re not going to go out again just in case

**SB**

No fair enough. How do you think patients engaged then with the cancer prehabilitation programme? What was their understanding when they were initially contacted by yourself?

**P2**

I found it good in terms of you know you were ringing them and saying I’m [PARTICIPANNT NAME] I’m part of the prehab team and they were like oh yes, so they had been told again that was something we tried to drill in from infancy that you know it needs to be the consultant or the CNS because MacMillan there’s been loads of research in terms of exercise and health but you know if the patient is told by a doctor to go and do x, y and z they’re more likely to I don’t know the percentage off the top of my head, but they’re more likely to do it than somebody in a tracksuit in a gym going you should do this, so my feeling is that its definitely happening and the nurses are explaining that this is the prehab pack ,and these people we know like they’ve just been given really bad news and again research will show white noise, they’re not taking anything in at that time, but the nurses are obviously saying if there’s somebody with them, look this is the pack [PARTICIPANT NAME] will be in touch, and when you are ringing them they know who you are, the majority of people know who you are and will say oh yes yes, and just talk through have you go the pack or arrange to ring them back at a different time whereas beforehand, or even now if you get a move more referral they’ve maybe gone to a health & wellbeing event or the information and support manager in the hospital it’s maybe taken 2-3 weeks for that referral to get to us, so we’re ringing people and be like oh I’m [PARTCIPANT NAME] the move more coordinator and they’re like who are you? What is move more? They don’t know, but the prehab I do find that they do know. Now it’s not saying every single patient will be, they’ll know who you are but they’ll be wanting to do it, there’ll still be people when you ring are just not in the right mind frame and that’s fine but I think majority have been good and they know who you are, and the nurses the consultants or whatever are definitely doing a good job on their end when they make the referral.

**SB**

Yeah and you’ve mentioned there some sort of barriers in terms of patient engagement sort of life changing news and maybe not in the right place, the time to surgery or being taken early for surgery and then in terms of the mode of delivery that you’re not able to see them face to face, you’re not able to always do the sit to stand. Are there any other barriers you’ve found to patient engagement?

**P2**

No I’m sure there’s more, the odd time you know, well the majority of the time family can be good but you might get the odd family member who maybe you know it’s an elderly mother who’s been diagnosed and the family are you know, they shouldn’t be doing that they should be resting you know, which is typical with all exercise we find it with cancer patients across the board, once you begin you know twenty years ago you know you were told as soon as you got a diagnosis to go to bed and rest, but we know the research has said the more active you can be the better it is for your recovery, so it’s maybe those little challenges of family around the patient that you kind of have to win over as well as the patient. Like other than that.. you know I don’t find that too many barriers as I said because I’m going into the home and you might get someone who says you know… I had another little lady who you know goes to town on a Saturday with her husband and maybe goes out once a week with her sister during the week but doesn’t has never done any sort of exercise and me ringing her saying you know you’re going for surgery in a couple of weeks, we need you to be doing a wee bit more and they’re like oh no I couldn’t dop that. So I’m like well talk me through what you do and she’s telling me about her house cleaning and stuff, so it’s all stuff she does, it’s just you know shaping it to you know fit it into her sort of lifestyle, but if it was me saying to her no you have to come into the gym, like I know straightaway that will be a barrier there’s no way she’d ever do it you know

**SB**

It takes that creativity. So in terms there, you mentioned there family can be a barrier, have you any experience where family have been helpful

**P2**

Oh yeah and as I said it’s probably the majority of the families are helpful you maybe have one or two out of ten patients that you get the odd one that aren’t. But definitely I think family support is a huge benefit to us and the patient. You know I’ve had patients where I deal directly with the children you know the son in the house than maybe the mum’s own phone, you know ring the son and he’s like great on the ball, or like husband and wife.

I had one last week and it was the husband who had the diagnosis but the wife she had the exercises every day and she was getting him and helping each other, and like that does make a huge difference even at the start as well, you know because patients had to go to the appointments on their own you know they weren’t processing that information by themselves, so now you see maybe they’re allowed to bring someone with them who is taking the information in and maybe hearing how important it is that they do this, and like I’ve had a couple of lung patients again post-covid when we knew patients were coming in , they were getting diagnosed later, they were sicker, their disease was further on, they were end of life pretty quickly, but still being able to make the referral and go into the house and you know and maybe in pain but like the daughter’s wanting them to do it and even them wanting to do it although physically they weren’t up to doing it, but because the support of the family was there you know they were willing to try which you can only ask you know people you know. I could nearly I’m sure all the coordinators will say when I go in you can nearly say who’s going to do what you ask them to do. You’ll go in and they have the folder out and they’ll have all the things marked and they’ll maybe write down all the things they’re doing that day then you go in and there’s other patients who maybe just aren’t in that right mind frame you know and maybe just don’t have that support of someone, and you just know they’re not into it like.

**SB**

That’s very interesting so it is actually hearing all the different wee bits even I hadn’t considered like you’re raising a lot of wee interesting points there.

What do you think and we’re kind of moving into what you think of the wider programme in terms of sort of the level of support provided. I know you have talked about how it as really good being part of those early meetings and planning and you’re still part of those meetings and you’ve mentioned that those have been useful to you, what other aspects of the implementation of the programme have you found beneficial to you?

**P2**

I’m a big believer in the prehab like, and I suppose out of all the coordinators like I you know I believe it works, I believe the earlier we can get an intervention with the patient you know, some patients you know it’s behaviour changes just planting that seed, they might not do any exercises in those two weeks before surgery but it’s the seeds planted and post-surgery they’re more likely to pick them up. In terms of the implementation of it, it’s been very well done in terms of like everything all the aspects have been thought about, the referral process is great, it’s quick , it’s convenient, it comes to all the coordinators. Even now the evaluation process is being you know really well looked after and I think all the different teams involved in the partnership working has been massive. You know some days we go to those meetings and you see move more coordinators maybe doing all the referrals and it’s kind of like if you took us out of this the service wouldn’t run, you know. We provide a lot to the service so I think maybe because we were involved from the start as I said especially me as I said I love doing the prehab especially since covid and people patients are getting diagnosed later, didn’t go to the doctors blah blah blah, we’re picking them up and you’re making a difference you know. I don’t think I answered your question SB as well

**SB**

No, no I maybe haven’t asked it either quite well, but was there any aspects, anything you felt as a move more coordinator you felt you benefited from so like thinking about those monthly meetings, was there any other aspects that you felt that really helped me, communication or whatever with other stakeholders?

**P2**

Yeah I definitely think those meetings are really important, I think that’s probably the big takeaway and I know it’s something that I have discussed regionally with our coordinators at our community practice meetings that if you know prehab goes regional those meetings are vital because you know we not only have access to the teams, you know each tumour site individually invited us to a multidisciplinary team meeting so we went to a big team meeting where we met all the consultants all the nurses they know who we are, and that connection , that two-way connection is so it might be so simple to some people but I feel comfortable enough that if I get a patient in I can pick up the phone and ring a nurse or ring and speak to someone rather than not. And there’s a prehab programme being run out of the Belfast trust here that’s meant to be kicking off this week that we have had no involvement in, and it was basically a static email last Thursday night saying you know this is starting next week are you happy to take referrals? And it was like woah, no pathway set up no nothing and I was kind of like this is not even like our evaluation of this isn’t even finished you know, so I definitely communication is key and those meetings are really important both for us as feedback any issues, but just to get clarity on things like the trust and that to see what we are doing on the ground because again if you take us off the ground you know I don’t know if physios and that would have capacity to run the service the way it’s being currently run at the minute.

**SB**

And is there any I know we’re kind of towards the end of the time, but is there any barriers that you would like to flag either with service delivery or follow up? Anything you want to touch on?

**P2**

Not really. Again I suppose I’ve raised them at those meetings and I’m sure [HNC CNS] has fed back stuff to you already but you know going forward I think the referral time you know we’ve been getting referrals that are you know a week to surgery, and its’ not you’re not going to do anything in a week and as much as there is research that you can see benefit in two weeks, you can do stuff in two weeks which is grand but I think going forward the referral criteria maybe needs to change slightly in terms of not accepting referrals for one week because you know especially when you’re trying to collect data in two time points, so you’re maybe seeing them on a Monday and then Friday and it’s like what’s changed in between those four days? Not much. So that’s the only thing moving forward that I would like to see changed. I think as we go bigger with the prehab and go regional and more tumour sites it will create more problems. At the minute we’re quite small and it’s quite good you know we work well together, but that’s maybe something that again will be trial and error when we go bigger you know how we deal with capacity stuff like that

**SB**

And those are considerations definitely that then for the sustainability of the programme, both for you guys long term but also regionally. Can you think of any ways to make improvements that need to be considered for future implementation either here in SE trust or regional? Like what do you think is required?

**P2**

Yeah I think I know our funding has been extended and there is prehab money is coming in and there are going to be clinical coordinators appointed in all the trusts, my concern that I’ve raised again with MacMillan when we do go regional. Again at the minute so we are a 1-man band. There’s one coordinator in each council area so for example move more doesn’t run if I’m not working. So I’m going off for three weeks in July. If I’m not there move more doesn’t work and obviously referrals I’m in communication with my referral links in they’ll be getting an email saying just let the patient know the referral will be picked up in three weeks etc. and that’s fine for the majority of move more patients because they’re post treatment, but for prehab it’s time sensitive, these people have two weeks, six weeks to surgery, so if I’m away for three of those weeks they’re not getting seen in my council area, but at the minute how we do it is there’s four of us basically cover the area of 3 and my trust, well [MMC NAME’s] Belfast and she gets a lot of referrals she’s in the prehab pilot. We have a little group a WhatsApp group and I’ll say now girls I’m going off if there’s any referrals come in can you pick them up and that’s fine again we had an issue where one of the girls was off sick and we didn’t know. So her referrals weren’t getting picked up so we had a bit of a discussion around whose responsibility is that to let everyone know again because we’re all employed by different councils there’s different procedures, different policies so my fear going forward into a regional structure is how are we going to work that, and off on leave is maybe an easy one to find because you can discuss with the other coordinators but if someone was to go off long-term sick or if someone had a bereavement in the family whose responsibility is then to work out who’s picking up referrals and who has capacity and you know are those referrals going to be getting different treatment because they’re not going to be seen face to face because they’re not in our council area and we’re not going to be travelling to them, stuff like that. So there’s maybe a few things we need to be thinking of long term and regionally as well but I’m sure that’s something for MacMillan or the trust to figure out

**SB**

Yeah, no a very valid point. Absolutely. Thank you for all that you have shared today it’s been very insightful. Is there anything you want to add before we stop?

**P2**

No I don’t think so and if there’s anything [INTERVIEWER NAME] that I haven’t answered, if you need to come back to me please do

# P3

**SB**

There we go. Okay, so we're just going to work our way through some sort of like outline of questions here. And we'll just see where that takes us. So just thinking back and to before this whole Cancer Rehabilitation program started, what did you know about cancer? prehabilitation?

**P3**

I think obviously, being involved in the world of physical activity, I was familiar with rehabilitation, particularly cardiac rehabilitation. But to be perfectly honest, I knew nothing about cancer, prehabilitation.

**SB**

So, yes, that was all very new. And when you did begin to hear about it, and how did how did you hear about it then? in the first off?

**P3**

Well, in the first instance, I was invited along by my ad, to help design and produce the standard operating procedure for the service.

**SB**

OK

**P3**

So I suppose my first task was to find out what cancer prehabilitation was. So that entails a lot of desktop research, and requesting information from a range of key partners from around the table with regards to the literature, the evidence, and obviously, what their suggestions were, in shaping the design of the service. So, bit a desktop research, looking at the Manchester model, which was at the forefront of Cancer Prehabilitation, in terms of service design, and they had robustly evaluated their service. So that was a good starting point for me.

**SB**

Yeah. And, and compared to what you learned in those early days of setting up and writing up that protocol, has your perception changed at all of Cancer Prehabilitation.

**P3**

I suppose again, it's important to highlight I didn't know anything about cancer prehabilitation. So I suppose I had no views on it. In many respects, so it was probably a steep learning curve for me in terms of what, you know, how do we define cancer prehabilitation? What are the key components that make up cancer prehabilitation and therefore, had to be an integral part of the service design. So I suppose my perception now, is that it's a very, very worthwhile service, that can make a huge impact on the lives of people presenting with cancer. So hopefully, that sort of sums it up, without getting into too much detail.

**SB**

Yeah. In terms of you said there, that you had no background and in terms of cancer prehabilitation, but how do you how well, do you think you're prepared for your role, in this program and the planning and preparation of this program and the roll out of it? And I'm sure there's other parts you were saying there about being able to sit down and look at the literature and evidence base and pull parts of this skills there that you brought to the table? Can you tell me a wee bit more about that?

**P3**

Yeah, well, I suppose my role within the SE trust is, you know, in Health Development, and a key part of it would be designing, implementing and evaluating probably a vast number of initiatives, both at that primary prevention stage, but also the secondary prevention stage as well. So I suppose my skill set fitted nicely with what was being asked of me, and indeed, my experience in designing, implementing and evaluating new initiatives. So, you know, I've got the Prince2 Practitioners qualification, I've designed several other services, and I've produced several standard operating procedures. So yes, and in terms of being equipped for it, it fell nicely into, I suppose my skill set. So it was not that it was easy, but there's a sequence of events to follow, which makes it easier. Yes, in terms of conducting that research, completing a literature review. You know, establishing the causes, the sort of the the interventions that are going to make an impact on an individual's health and well being the valuation tools, and so on and so forth. So yeah, I mean, I felt pretty confident and well equipped. I probably did underestimate the time involved in pulling the project together. And therefore, could have done with more resources. And the, the time span was pretty ambitious to design, implement, and put in place an evaluation framework and get agreement with all the partners, but it was timely because of COVID. A lot of our initiatives were sort of either parked, or were running on half speed. So we did have capacity at that stage, albeit, it was challenging.

**SB**

Yes, yep. Was there any helpful training that you received? And above and beyond that, you know, the literature reading the literature? Was there anything you attended? Or, like, virtually? or in person?

**P3**

Um, not really, obviously, through attending the steering group meetings, you got a flavour of what was being proposed, there was an initial brief, which was produced. Which sort of guided I suppose my role and my activities somewhat, but in terms of formal training, probably no, no, it was very much based on your own and your own responsibility to dig into and investigate, you know, what cancer prehab was and what the key components of the service should entail, and look at the evidence. So no, no formal training, other than my background in project management and so on and so forth,

**SB**

which is essentially your role in this project?

**P3**

Yeah.

**SB**

On reflection, is there any way you could have been could have been better prepared for your new rule? I know you mentioned there, you could have done with more resources. But is there any other way that you could have been better prepared?

**P3**

I think overall, if I'm being very critical, it probably didn't follow a very clear sort of project outline. So it was a, it was a bit bitty and there was probably a reliance on me and my team, to shape that quite a bit, which we were happy enough to do. But the key partners were round the table in the form of a steering group, which was important. And they did help shape the service and agree what the service should look like, which was fabulous. Yeah, resource wise, we probably could have done with more administration support. As I say, we had to establish the structures to take forward the project, there was a steering group in place but we have to establish a number of task and finish groups, which was a good approach, but it was a bit woolly if I'm being honest in terms of project management, there was probably no clear management structure in place would which I think would have would have helped in terms of a project manager and who was responsible for various tasks. A lot of that work fell to ourselves, probably ad hocly. But nevertheless, we navigated our way through it

**SB**

Yeah. And yes, it sounds like those are things that would definitely be need to be considered for future roll out there just as you're talking in terms of project monitor and administrative support from your experience.

**P3**

Absolutely. I mean, it's obviously a brand new service area. I think sometimes we underestimated the amount of time and expertise required to really shape and drive the project forward. You know, we had to devise from scratch our project plan. Which again, is fine, I'm not complaining at all, but I suppose a better structure a nice sequential flow of what the tasks entailed. would have benefited probably us as a project team if you like

**SB**

okay, okay. Um, you had mentioned there that COVID-19 actually helped you somewhat in that it freed you up and you had more time to dedicate? And is there any other ways that COVID-19 actually helped or hindered the project from your point of view?

**P3**

Well think in terms of delivery, obviously, a lot of it was virtually delivered via Zoom, particularly in the physical activity component. So it's a bit of a double edged sword in many respects, whereby some people can avail and adapt to the use of technology quite easily, whereas others cannot. So there's pros and cons with that virtual delivery. But yes, I mean, capacity wise, it did allow us to really focus and zoom in on the project itself. And if we hadn't had that time, I dare say, you know, the project probably would have taken twice as long to design produce, and you know, implement. Was there any other advantages with COVID? I can't think of anything else off the top of my head SB

**SB**

Yeah. And any other barriers that you faced as a result of COVID?

**P3**

Yes, of course, we've got to consider the capacity of other teams, other contributors. And I know we had a block with our Dietetic team. And it was an integral part of the overall service in terms of providing that nutritional advice. And unfortunately, because a lot of their staff were redeployed, they had to stand back from the project for a short time. But luckily, they reengaged as staff returned to their, you know, substantive posts. We got there in the end, but yes, that was a that was a challenge for particularly the Dietetic team. Overall, I think there was possibly, how could I put this, overall I think from a senior management team, the cancer prehabilitation service probably wasn't a priority. I think dealing with COVID was a priority. But nevertheless, as a as a team, we decided to progress with the service design. It didn't hinder us too much.

**SB**

Yeah. And it is incredible what you have achieved, it's fabulous. Given the backdrop Yeah, absolutely. What benefits to your professional development and practice are evident as a consequence of being involved in this cancer prehabilitation programme?

**P3**

That's a good question. I suppose it always, it always helps you refine your approach. And there's always learning in you know, not all these projects are textbook as such. So you know, we have to be flexible and have to be adaptable. I think that just reinforces that message, particularly over COVID, and throughout COVID, so the commitment to the project was absolutely fabulous. I think that really shines through the teams and the individuals involved were 100% committed. And that made life so much easier for me to, you know, take this project forward to have champions in each of the sort of subject disciplines from, you know, the cancer teams through to dietetics through to mental health just made my life so much easier. So, the learning for me was, I suppose, just to add to that, to reinforce my approach, you know, to be more flexible, more adaptive. And obviously, it was learning about the service itself. What was. And the various tools then that could be used to evaluate the service. So yes, of course, a lot of learning. And there was a lot of partners involved. So the benefits to me was reinforcing my relationship and my sort of connections with the various teams that were involved in the project.

**SB**

Brilliant, in terms of you, and your sort of satisfaction with your job, how did that impact? You know, did you enjoy participating? Did you get something out of that?

**P3**

Very much so. I mean, it's probably part of my DNA. I like to be organized, very organized. And I like to see the flow and a clear plan of what we're trying to achieve. And because a lot of that was, I suppose, on my shoulders, I was able to influence that and shape that accordingly. So I suppose that allowed me to control and shape the project, based on the evidence and based on other models of really good practice. So for me, it was probably, you know, a real good example of a gold standard service. And as I said, the partner's contribution to that was was fabulous. To have that engagement with, you know, both our own statutory colleagues, and the community and voluntary sector was fabulous as well. So but yeah.

**SB**

Sounds like you really enjoyed the experience.

**P3**

I did. I did enjoy it. Albeit it's a pressurised environment. It Yes, I mean, downs to my portfolio of sort of my experience and my examples of project management, and so on, so forth. So yeah, yeah.

**SB**

Yeah, and that that's really shone light through the project? What, and you've touched on some of this, I think already, but what improvements do you think could enhance the sustainability of the cancer prehabilitation, so sort of areas for improvement or consideration for widespread rollout? And you've mentioned there about having dedicated project manager and admin support? Is there anything else you want to say about that, or elaborate on anything else you can think about that would have enhanced the sustainability of the programme?

**P3**

Well have taught to not structure, I think, you know, having the right structures management structure in place is vital. So whether that's, you know, a regional steering group, made up of the right people. And then a project plan, which incorporates the various elements of the various stages of a project. Standardising the outcomes framework would be very useful across the various trusts, so that we can benchmark and compare and contrast. Probably more user engagement would be useful. So, you know, actually involve those people with cancer and indeed, their families, in how we shape, adjust the program to suit. Clearly the financial side of things is, is a key gap in terms of sustainability. And I know working with our council colleagues, that's always an issue in terms of them, allocating, you know, instructors or coaches to deliver the physical activity component. But I suppose if we look at the various components within the service, you know, physical activity, nutritional advice, and emotional wellbeing advice, they all need to perform it appropriately if we're really to make an impact on this here. The physical activity component I think could be, could be improved vastly, by having, you know, dedicated bespoke instructors to deliver the scheme in the way that you want the schemes to be delivered. I think there was a bit of a transition for some of the instructors who prescribed or recommended rather physical activity initiatives just to become more active to actually exercise prescription. They are two very different things, they complement each other but from my experience, and from the evidence we need highly qualified coaches who are able to prescribe exercise because very often you have a very short window, you know, through which you can improve one's functional ability. So that that could be improved vastly, in my opinion.

**SB**

Was that something that evolved throughout the lifetime of the project so far? Did the Move More Coordinators move towards prescription.

**P3**

Yeah, I mean, it's, it's probably still a wee bit loose if I'm being honest. And I think COVID probably hindered that somewhat in terms of the virtual delivery. I think if there had have been a face to face delivery, it would have been much more succinct. You know, and that spans from the functional assessments, which is very important, pre and post to actually identify how the individuals have improved. But also from the exercise prescription, what were the key pieces within that prescription that made the biggest impact? You know, was it the strength and balance component? Or was it the cardiovascular component? Was it tailored to the condition? Was it tailored to the the tumour site, for example. So I think that could be improved vastly. But also, the support for the coaches, the physical activity coaches, I think, is paramount, from a clinical perspective, and to have that support, particularly around the physiological side of things would probably help improve the service

**SB**

So that wasn't something that was there P3?

**P3**

Not not to the extent needed. I think I, when I looked at the Manchester model, there was a dedicated doctor who supervised the coaches, and answered questions related to, you know, the individual's conditions, the most appropriate form of exercise, you know, maybe some of the barriers or the issues, whether it's the pharmacology, and how that would impact their functional ability, for example, you know, all of that there just to bring it a wee bit more into the clinical world. Yeah. And I suppose from the emotional wellbeing side of things was probably reasonably limited in that we were relying on our assistant psychologists. Now I mean, they don't deal specifically with cancer patients, but probably more training and support around what that looks like. And then capacity for our consultant psychiatrists to be able to, you know, cope with those patients who are identified as requiring more in depth help and support possibly, as well. So we know on talking to our own consultants, there is a gap there or there's a limitation, however, in saying that what they suggested was the first 100 days usually doesn't require an intervention from a consultant psychologist, we're trying to, obviously absorb the information and, you know, process that. But maybe down the line, that that's something we need to follow up on perhaps. So yeah, I mean, it's, is there anything else you want from that or

**SB**

No, no, that's not the you've highlighted quite a lot there. That's very, very useful. And kind of last point, [HEALTH DEVELOPMENT MANAGER NAME] was just around barriers for long term delivery of the Cancer Prehabilitation Programme. And I suppose in effect, a lot of it would be maybe the opposite of what you've said not having those things in place. But yeah, what are your thoughts on barriers going forward for the SE Trust?

**P3**

For the SE trust I think, securing funding is vital. But for me, I personally would like to see a more joined up approach between that whole preventative world and the secondary prevention side of things, and the, you know, the rehabilitation moving forward. I think it's important to link all those pathways in some guise you know, between mental health, nutrition the physical activity side of things are areas for the SE trust, I think the key barrier is, is the capacity of, you know, that whole team involved. So that needs to be considered moving forward between the cancer nurse specialists, you know, screening assessing, making that referral needs to be addressed. I think, skill set of the coaches and adapting to a more clinical approach, I think is vital, but having that link into the community and ongoing physical activity is essential as well. So lifestyle behaviour change. That dietetic support, obviously is very important in moving forward and what that looks like, as well as the emotional wellbeing side of things, which I've mentioned already. I think they need to be funded appropriately. And there needs to be capacity if we are to upscale this service. And I know, we're dealing currently with quite low numbers. But if we want to scale this up, we need the appropriate resources in order to do that there. And then the the outcomes, I think we've got a really robust evaluation framework in place, in my view. And I know it has been challenging at those key points of collecting that information. And clearly, we need to tighten that up in order to evidence that you know, the program more effectively, if you like. And lastly, probably promote the good work that we're doing, you know, so shout about the work that we're doing, try and get a bit of consistency across the province if that's at all possible.

**SB**

Yes. No, absolutely. That Yeah. That will be key won't it too, as you touched on earlier as well, having an agreed way of moving forward so that we can compare, compare and contrast and see what's happening in different places and look at the challenge on resources as a result. Yeah, no.

**P3**

I think at the minute [INTERVIEWER NAME] obviously, we're focusing on three tumour sites. And there is quite a bit of interest from other tumour sites to be included in this. So again, if we're looking at scalability, we need to resource it appropriately. Yes, so some sort of scoping to see what that looks like. And then to to address the funding side of things and the resources side of things

**SB**

because you do that in the population increases, which puts more pressure on the likes of the move more coordinators and the rest of the team. Yeah. Speech and language therapy. Were they part of the roll out?

**P3**

No, no, they weren't. There was a number of inquiries from other tumour sites. I can't remember who they were off the top of my head. But I know there was a number of inquiries from other tumour sites to be included in this pilot. But again, capacity didn't allow as it was a sort of prototype if you like, just to see how it would roll and yeah, yeah.

**SB**

Cool. Thank you so much. Have you any final comments? Um, [PARTICIPANT NAME]?

**P3**

No, just to say it's been a fabulous project. I think it's needed. It's, it's worthwhile. You know, it's changing the lives of people who present with, you know, cancer. And, you know, potentially the savings, although we don't like to talk about that too much. But the savings to the health service in terms of that whole preventative secondary prevention work is enormous in terms of, you know, bed days, and so on and so forth. I suppose that support from our informatic, you know, planning and informatic team has been instrumental in trying to pull together all the various evaluations and metrics. So, you know, that that needs to be considered, you know, robustly moving forward, and not to be underestimated, but it's been a pleasure to work with the various teams and to see it come into fruition. So a fabulous example of partnership working, and it's probably been one of my most enjoyable projects. I have to say, so. It's great.

**SB**

Fabulous, fabulous. Thank you. I'm just going to stop the recording [PARTICIPANT NAME].

# P4

**SB**

That's super good. Okay. So just to begin, [PARTICIPANT NAME] thinking back to before this started in the South Eastern Trust before the Cancer Rehabilitation program started, what did you know about cancer prehabilitation back then.

**P4**

Before it came to our trust? Emm I had been called to an open day, a MacMillan study day, sorry, I think in the summer prior to that, and and there was a talk about prehabilitation at that, that that was the first it was launched in Northern Ireland. And, and, yeah, it was quite exciting. The whole Manchester model was explained. And and then sort of a plan to take it forward from there. So yes, that was the first I've ever heard of it, you know, just prior to that study day. So anything we did with regard to preparation for surgery, I suppose we would never, it was not, it wasn't totally new terminology. But I guess we could say our patients were being prepared for surgery in the past, and that would have been in relation to the Enhanced Recovery Program and some of the steps that were given for that. And that would have included taking carbohydrate energy drinks prior to surgery. And obviously, you would have taken the opportunity to, you know, assess alcohol and smoking at that time as well. But there probably wasn't the same emphasis on it in fairness. You know, as it is embedded in the prehabilitation program, so, so I didn't know about it until probably the summer before. Whenever I initially went to that MacMillan day.

**SB**

Yeah. So you said there, and you're, you seem quite excited there. Whenever you went to that day, do you still have the same enthusiasm for the project?

**P4**

Absolutely. Absolutely. Yes, no, very much so. I think that's exciting. The fact that it's something that's new, but it's new to Northern Ireland, and it's, we were the first to pilot it here. And our patient group were well set for prehabilitation. Because colorectal surgery is major major surgery. And our surgeons sort of would explain to patients that it's sort of seen as more serious, more hard on the body than open heart surgery, you know, so anything the patient can do to prepare themselves for that. So the fact that we're sort of sending a wee bit of control back to the patient and trying a bit of responsibility, you know, this is really, you know, you're dealt with this diagnosis, and what can you do for yourself? So yeah, I think that is exciting. It's just part of now of our discussion with our patients. And the results, and are, we are time limited with the information we give the patient and the overload of information that they get, so yeah, so it's exciting. And it is exciting to get good feedback on the fact that the patients are saying this is helping us. And, you know, so and the information from the, the steering group to say that, you know, there is good data there. So all that sort of motivates you to keep going.

**SB**

Yes, yes. And you were saying like, there was a lot of that you would have done before, but maybe unstructured, on this has that has been good then, have you.

**P4**

Yeah. I mean, we've been talking about health promotion for a long time, you know, when have good healthy lifestyles, and obviously with the Macmillan through Move More Coordinators, and getting patients into fitness after their surgery, this is all after to sort of change, you know, how to encourage healthy lifestyles, but this is sort of embedding this in an earlier stage that they can make those changes, and they can take the score for the rest of their life, you know, because we say patient to you've had this diagnosis, you've recovered for it. Let's try and keep you well, but we're starting we're, you know, we're thinking about that, you know, at an earlier stage now, so yeah,

**SB**

yeah. And yeah, you've touched on a few things there. But that being important earlier, to give control, then potentially improve outcomes then for patients. Yeah. Yeah. How well did you feel prepared for your role in the cancer prehabilitation programme?

**P4**

Emm I felt very well, I was involved in all the focus groups and at the start sort of planning for the what's I'm trying to think of the terminology for the actual document that was put together

**SB**

for the SOP the standard operating procedures

**P4**

though and so yeah, so I was involved in that, obviously, I had to work that round our patient group and our needs, and the three patient groups obviously, are all quite different. You know, obviously, the universal pathway covers both of the all three of them. But yeah, so I was very much involved. And decision making, you know, I was, you know, certainly I was involved at every stage. But yes, it was a big learning curve, when I come into it, you know probably [HNC CNS] and a lot of the team had done more. So I was sort of did take a wee while to get my head round it, you know, have an understanding of the ethos of it all. And then, you know, there was a lot, you know, the improvement team, you know, did so much to sort of get that up and running. And then you could see how the process works. So initially, yes, it was a bit daunting, but I was involved at every stage. And then sort of my understanding grew and how that was going to work for us, whether it was going to work in real time and how it was going to work with our structure of our clinics, and the timeframe that we see those patients and how we in practice were going to get the information over to the patients, how it was going to work with regards to the verbal information we give them, the written information we give them and how the referral process worked and how that was going to work in real time with when the patient actually engaged in the prehabilitation programme. So I was involved with that. But it did take me a while to get my head round, how that all was going to come together. But certainly they, you know, the team that were behind that, you know, certainly would have done the lion's share of the work. And then my expertise with regard to the needs of my specific patient group was brought in at that time. So....

**SB**

And that's good to hear about your role in the tailoring of the intervention for your colorectal patient group. Good. Was there any training beyond that workshop day? Was there any training that you received in your journey through this?

**P4**

No, I think it was really a case of attending the steering groups to understand the patient selection, really. But the patient selection really was all those patients bar the exclusion criteria. So yes, I was involved. So obviously, I was learning as I was sort of involved in those groups as to deciding these patients who were included in the next group. And then as regards training, it was really just following through the referral process, and understanding the patient group that needed to be referred, and those patients who would go in the specialist pathway. And so wasn't a case of having to go to courses, you were just learning as the operating policy was being developed, you know, so then I in turn then had to impart that information, down to the rest of my team. So I suppose the training was sort of as the, you know, the process was, was going along. And then I say it was more my role to pass that on, because I was in the whole colorectal team, you know, I'm one of seven colorectal nurse specialists. There’s 7 of us here, there's one of them's on maternity leave. So there would be a lot of us involved, you know, so, you know, it was my role then to sort of understand the prehabilitation procedure, and then to be able to pass that on. So there was no training per say, the fact that I was involved in setting it up, that I was learning along with the rest of the team, and then I followed that on

**SB**

Fab, on reflection, now that you're a bit down the line with the programme, is there anything that could have helped prepare you better for what was coming?

**P4**

No, no, I don't think so. I think emm because it just grew slowly, I think we learned that some of the a lot of the data wasn't being collected, and it was hard to then capture that information at the right times whenever we're meeting the patients, and sometimes we didn't get the opportunity to get that, you know, and ask those questions. And so that was through experience. And, you know, and sort of doing the remember, because initially patients weren't being referred without the correct data. And a lot of them weren't asking for stress thermometer, we weren't getting a lot of details, because we hadn't been used to getting that information, specific information. So I suppose it was with practice, that we were able to make sure that we were teasing all that information out of the patient at the right time, because we don't necessarily see them again until they're preoperatively. So, um, so I guess it was just really with practice, that we got into the habit of getting all the correct information. So it did take a wee while initially, a lot of information wasn't being teased out. So then there wasn't the rich data that we were looking for. And so there have been a few mishaps along the way where it was put back to us, look we've got this, we've got this referral, but we don't have some of the key information that we need we needed and maybe haven't assessed their smoking cessation status, their alcohol status, maybe haven't done their distress thermometer. Maybe you know, hadn't got their height or weight, you know, all those things that we weren't capturing before. Yeah, We are under a bit of pressure whenever we're doing our results appointments. So there may be two or three patients come up for results appointment, or the patients receiving their diagnosis are being told by the consultant about their planned surgery. And then our role is really to fill in, you know, a bit more of that information, clarify that giving them information about their about cancer, about their surgery, all those information they would have always given. And then we're starting to talk a wee bit about prehabilitation. By this stage, their heads are, you know, really overwhelmed, to be honest. And then again, now with COVID the timeline is not great so you know they may be given date for surgery for the following week, emm and then as we don't have a lot of time to gather that information, we are more efficient at that, and we'll maybe phone them and get the additional information with regards to the prehab. And I think a lot of those gaps have been rectified. But really just come it's now part of our practice. You know, we really had to adapt to try and make sure we were capturing all that information, as you know, but it is it is quite challenging for us, because of the length of time we have with them, the nature of the information they've already had to take on board and then we're starting to say yes but we want to get your height, your weight, we want to get this that and the other? so yeah it's taken time.

**SB**

Yeah. Um, so I'm sort of picking up from what you're saying there that emm the cancer rehabilitation programmes, enter just at diagnosis in that same appointment? Yeah, yeah. Is that the best timing given it's such a short time period for you guys?

**P4**

Well, the problem is, so the first time we may get these patients name would be the MDT multidisciplinary meeting, okay? So and but I can't phone them and say I want to refer you to a rehabilitation programme, because you have a cancer diagnosis, because they have to have that discussion with their surgeon, or that like nine times out of 10 to come in the door, they do have a fair idea that they have a cancer diagnosis, because we give them warning shots at the time of their camera test or you know, when they've had a CT scan, they're really focused on the information they're being told with regards that they have a definite cancer diagnosis. They don't have spread to their lungs or their liver, because they've had a CT scan, most of them are terrified, that maybe they’re being told we can do nothing for you. So so there's not, you know, we've looked into this, you know, [INTERVIEWER NAME], When else can we, you know, see these patients earlier, you sort of have opportunity. So, so there's no way around it, that the first time we see them, really it has to be after they've had that initial consultation with their surgeon. And so sometimes I'll say, look, I give them the pack prehab pack, I say, Look, don't even look at that until the girls, you know, the coordinators and they'll talk you through that. So, you know, I get them, I say, look, what can you do for yourself, and we want you to improve your lifestyle, we want you to improve your fitness so you're in the best possible fitness for a short period of time you have between now and your anaesthetic that might include reducing your smoking, alcohol, doing some simple exercises, I say, but they'll go through that with you. This isn't you know, we're trying not to scare the person to go, what are they going to put me through? So I don't spend a lot of time going through that. But other than to motivate them that actually this is going to be worth it? Because they've already had very significant conversations by that time.

**SB**

You mentioned there emm some of the challenges with the screening, referral and cancer programme process around time. Are there any other challenges and the other thing you actually mentioned there was just getting embedded in the practice, that was a challenge. Once that sort of gradual process, there any other challenges that you've experienced with that screening, referral and consent process that you want to share?

**P4**

I think imparting it to the rest of the team it was just that, emm well they've only got five days before their surgery, so there's no point referring on them? And, and, you know, I wasn't sure, they said they had a few drinks. So I didn't really like to push, you know, and go through the audit and see, and, you know, just it was really my job just to make sure that no, we really need this information. It's rich information for us to get, you know, good outcomes from the pilots, you know, to try and get everything. So I suppose it was really discussing with my team, the importance of getting that and then understand the rationale for that. Yeah, that every all our all these measurables are all equally as important. So that was really just, you know, I was getting feedback. Obviously, I didn't know the referrals other than my own referral. And so I was really I was getting feedback from [HNC CNS] and [PERFORMANCE MANAGER], to say look we're getting lots of referrals, but there's lots of things missing, so I then had to go the team so that was really the challenges for them, the staff, the girls to understand the importance of every aspect of those measurables that we needed

**SB**

Yep. And are there any components? Any of those measurables that you in your experience found were maybe not necessary?

**P4**

Emm I don't know, we didn't think the Distress Thermometer was barely an accurate one, because they are so extremely anxious, but I think we have a date on it. So if it is ridiculously high, then then obviously, then that's our judgment, then if they need referred for more the Associate Psychologist, you know, for that other than that universal emotional pathway, so so I think it's a very, it's by no means an accurate measurement. But I suppose it still has to be a baseline, you know, and the, you know, the, you know, the wider sort of prehab team agree that, yes, this is where they are then, but it's not a good guide. But I think when else can we take it you know? Em you know, so by the time we ask it they've been given that information by their surgeon so sometimes they have come down a peg or two, you know, whenever they walked in the door before they'd had that discussion it would have been higher again, so by the state, by the time generally you feel it is our role that we have reassured that we have gone through a lot of information and clarified things, sometimes they go out more scared. You know, there's no two patients the same. But yes, I can't think of anything that seems inappropriate really, you know, we need to get the score, and we need to get their rails, I can't think of anything really that wouldn't be relative, or relevant.

**SB**

Yep. Are there any helpful aspects to this the screening referral and consent process that you have found?

**P4**

not sure what you mean, help...

**SB**

for example like the fact that it's an online form, all the referral forms kind of integrated into one?

**P4**

It's not, it's not ideal, you know, I had said, look, is there any way that this document could flat could not go if certain variables were not completed? You know, I think [PERFORMANCE MANAGER] has an issue with it. So obviously, it's an Excel document, it's not, you know, what sort of, you know, it's not the best. So it would have been ideal that it would not the document wouldn't go if everything wasn't completed. So that sort of made.... But I think [PERFORMANCE MANAGER] is in the process of doing an additional version again now. So I think she has, her head's been turned and she's been very good, and very good at, you know, looking through each referral, and able to see what is or isn't there that should be there. But that would be my issue that if the everything wasn't complete, that needed to be that the document wouldn't go, and that would give, you know, the nurse a chance to sort of, you know, make sure that everything, all the screening tools have been completed. So I suppose that would have been more helpful. I mean, it is, I'm quite efficient with it, but you know, I can do it. Bang it out in about 10 minutes, but you know, you're like, you know, jittery at the start. And now I'm, you know, we're all saying we can do it fairly quickly, but it's just something else for us to do on top of other things, you know, you know, for our patients referring to MacMillan Health and Well being team they have to be referred, there's so many referrals to be done. So much written work to be done for each patient before prehab came along. So it is an added stress, there's no doubt that it has added to our workload, but at the same time, I would say, looking back, I definitely have become more efficient at it, and like you said it becomes embedded into that consultation with our patients, at the results appointment that you know, the key information we need to get from them, obviously, their paths and everything. So I mean, the support workers have been very good, making sure all those things are all still maintained, so I can put my hand to a pack straight away. And, you know, so there's been good support from the administrative side of things

**SB**

How interested are the patients to engage in the cancer prehabilitation?

**P4**

I think, I think a lot of them probably don't quite grasp it initially, because again, like I say [INTERVIEWER NAME], they're so focused at the fact that they have a new cancer, they need an operation, emm so they're not necessarily going to go Oh, yes, that's great. That's great. They, you know, they're just, I just have them along with it sort of thing. You want to do this, this is going to be great. This is what you can do to help help yourself. There's really I think there's only been like one person said absolutely not. And I think was more concerned about having to address their alcohol issues and things like that, you know, so that's difficult and No, I'm okay dear and I'm quite active and I go for a walk and I do all sorts of things. So but most patients are haven't really been aware of anybody who's absolutely refused it. And I think certainly the girls in the move more team would say, by the time they rang them they'd say no I don't know anything about this. But then, you know, because they haven't taken the information on board, they just haven't they've just been overwhelmed. And in the same way you know, our patients, we have a new stoma to go home and and the community stoma nurse is out because they tell the community stoma nurse they didn't tell me anything, I don't know anything about this, because again, it's just their capacity for retaining information, so emm so, and then there's those very, maybe the younger, more motivated patients who come in, and they're just soak it all up. And yes, oh that sounds great and asking for information, but a lot of our patients you know sort of age category, you know, it's overwhelming for them. And that's the one thing they might not take on board overly by the time they walk out the door that if they were asked in detail, what was the prehabilitation, they probably could not tell you what on earth that meant. Because by that stage, they are literally overwhelmed. So the girls are having to explain themselves probably the time the move more team, because you know that that is not unusual.

**SB**

Do you feel like COVID-19 has had a big impact on the service provision of the programme?

**P4**

I think it has, I think again not from my perspective, because I'm still thankfully getting to see my patients face to face so I can make that referral. I think how that passes on a face to face situation for the coordinators themselves, you know, obviously, they're having to do this over the telephone. And, and that's been very challenging for them. I think they are able to get out again, now and so yeah, so I think that's been a big issue for them. And I think they have worked different ways. Each of the coordinators have sort of managed things in their own way. But there they've been amazing so and so I think that from their end it's been more of a challenge than ours, you know, it hasn't stopped us been able to offer the, you know, the service, and we haven't made it an issue other than the fact that it may be done over the phone. And that's makes it less effective, obviously, because a lot of our patients don't necessarily have access to emails and things like that. And so that is from their end certainly it has been difficult, but we've certainly been able to offer the service regardless.

**SB**

Yeah. And in terms of emm has there been any benefits to COVID-19 in terms of service delivery,

**P4**

Emm with regards to prehabilitation I can't see how there has been any great benefits. As I say we've seen them and offered. Again, sometimes that's less than a week, then the amount of prehabilitation is limited. But even those patients get one session, one phone call, again, the thought is look, this is them, instead of us referring them to the move more coordinators six months a year postoperatively they're actually getting getting in there early beforehand. So they will hopefully continue to sort of carry on with those healthier lifestyles. So even the one session, one phone call one, you know that say all right, okay, well, I'll keep going with us, you know, once I recover from the surgery. So and so I suppose maybe. I don't know that whether there's COVID or not. I don't see that it's had any benefit, but I don't think it has from a nursing perspective. And as much as I still have the opportunity to see the patient face to face. So I can still make the referral, I think the practicalities of delivering have been more challenging.

**SB**

Yeah. In terms of your professional development and practice, has there been any benefits for you be a part of the cancer prehabilitation programme?

**P4**

Yes, it's been nice to follow through with a pilot and to be part of something new for the service. And I suppose we don't always boost ourselves enough. Emm and you know, I've had colleagues from the Belfast trust and the Northern Trust who are now you know, prehabilitation is sort of filtering through to other areas. And they've been then asking for my advice, my information and my knowledge and expertise. So, so you suddenly, you know, from being the novice and not know much about it, you know, you're the one that's showing a bit of expertise to pass on so yeah, so it's nice to be able to say that you've got a new service off the ground, and it's working well. And it's being developed. And, you know, we're starting to see very positive benefits from that. So yeah, it's nice to sort of say that you know you can take some of the credit for that and and pass on, you know, pass that on

**SB**

Yeah, absolutely. And, yeah, it's nice to enjoy your job isn't it? Yeah,

**P4**

yeah. We do. We're very lucky in this particular job and colorectal is great. You know, it's we're never short on numbers. SB, you know, the place is mad, but we have a good patient journey and we see the patients, especially those patients who end up with a bowel you know a stoma. And you know, we see them quite a bit and then we're involved with cancer follow up clinics as well. So the patients don't just see the consultants for their follow up. It's also nurse led. So we do have, we do see the patients right through. So it's good to follow through that journey and that potential five year follow up.

**SB**

And you're starting that relationship then that are in terms of Cancer prehabilitation. It's allowing it to be earlier on,

**P4**

I suppose. Yes, we are. We're just a year into the pilot. And so I guess you would like to think that we will see the benefits of that down the line, you know, that those patients have, you know, have made those good changes to their lifestyle, and have continued with that, you know, because they've been given that opportunity at an early stage. So yeah, here's hoping that we can see the benefits from that in the years to come.

**SB**

Yeah. What improvements do you think could enhance the sustainability of the Cancer Prehabilitation Programme, thinking about improvement within the SE Trust for widespread implementation? You know, you talked there about other trusts?

**P4**

Yeah, certainly. Yeah. For us for the colorectal service. I think it's, it's getting the specialists linked in with the likes of the physiotherapist and the dietetics. You know, I think that's important that the patients can avail of those specialist services when they need it, you know, for colorectal the patients who require that would be those patients who are going to have an ileostomy type of stoma, and those patients who are going to have you have a rectal cancer who are going to have pelvic surgery, and then they potentially have issues with bowel dysfunction, erectile dysfunction. So I guess it's making sure that the resources are there for those patients to avail of those specialist services when when that happens. And to date, I have been aware that they have had referral those specialist physio referrals have gone through. Yeah, but I suppose it's an it's making sure that the resources are there. And then we weren't given resources for prehab in our team as all the teams were. But of course, that's quite quickly, you know, submerged with the busyness of everything, and you know, staff off on maternity leave, we've had somebody off COVID Most times, you know, so any of those extra hours we get, you know, suddenly mean nothing, you know, when you're pulling in other directions, so I suppose it's having enough resources to follow that on, and to be able to make a specific phone call to the patient after you've seen them at that results appointment to focus maybe on the prehabilitation, you know, whenever they've had time to go home and digest a lot of what's been spoken about. Maybe to focus actually, I don't know if you recall we had a conversation about prehabilitation I just want to go through that with you again, it'd be nice to avail of that time, extra time. And as I say, just to make sure because I know the dieticians were resistant to be able to see any of our patients emm preoperatively. And I pushed for those patients with ileostomy. But again, their resources are are limited. So I guess it's making sure that those specialist resources are there,

**SB**

yes. And adequately funded to carry out their part of the programme, particularly that's going to be particularly important in the upscaling of this if there was I'm thinking about the south eastern trust if it was widened out to other tumour groups. The likes of those special services doesn't it?

**P4**

Yeah, yeah, no we want to prove that what we've done has been to benefit to the service to the patients, firstly, and to the service. Yeah, the wider service definitely.

**SB**

Yeah. Can you think of any barriers for the long term delivery of Cancer Prehabilitation? So I suppose you're saying there not having adequate resources is going to be a barrier? Are there any other barriers that you can see?

**P4**

I can't see other than it's just always always time and resources, it's always you know, that that information is disseminated, down to those patients or the staff, who are delivering the service so that they have understanding of what the prehabilitation service is, so they are delivering it correctly. So it's being able to make sure that, again, that we have enough staff to do that, and we have enough specialist staff to do it. And I would be, to me, that is the only barriers otherwise it's all plus, plus, plus, you know, everything about services is a benefit. So, yeah, it's always about time and resources. And they're one and the same really, emm as I say, it is more or less embedded into our you know, our results equipment anyway, know that the referral is made and our Enhanced Recovery Program, our ERAS, you know, the prehabilitation is now tied in with that as well. And that, you know, so when we're collecting data for our ERAS patients that you know the fact that they've had prehabilitation we're hoping that that may demonstrate, you know, again, reduce postop complications. And you know, sort of we're looking to see wider benefits within the service.

**SB**

Yeah that’s fabulous, that concludes the interview. Is there anything you’d like to add?

**P4**

No, I just hope you’ve got everything out of me you need. It’s nice when you talk about it you realise gosh we’ve done quite a lot so it’s nice to see things coming together so it’s good that there’s someone like yourself who’s teasing it all out of us. I think there’s a couple of the other girls are going to speak to you as well, so that’ll be most of the team

**SB**

No that’s fantastic, I’m just going to stop the recording

# P5

**SB**

super. Okay, so thinking back to before you had any, like, a part of the actually just go back, because I'm not clear, when did you come on to the Cancer Prehabilitation Programme? The very start or not

**P5**

So I was placed in SE Trust at the time when the kind of concept meetings were happening happening. And I was meant to be part of it sort of from that year, but with COVID, then it was a year later. So a lot of those preliminary concept meetings, I'd been in a few of them right at the very beginning. And then as that all developed, then I was in another trust for clinical purposes.

**SB**

Okay.

**P5**

And then I was meant to take on my adept project, sort of a year earlier. And but actually, then I started in August, when the pilot had been initiated in the March.

**SB**

Alright, okay.

**P5**

So then the emm framework had all been sort of developed.

**SB**

So whenever you're a part of those initial concept meetings, had you heard, did you know anything about cancer prehabilitation at that stage?

**P5**

No, very little. Emm so for, obviously, it covers the three tumour groups, my area of working is within colorectal, and so I knew about the sort of pre-assessment and enhanced recovery, which are kind of the preludes to pre-assessment, but it was a new emerging area of work, not only within colorectal, but obviously across other tumour sites as well.

**SB**

And what were your thoughts about it back then do you remember?

**P5**

So I think, initially, there was a lot of, in the concept meetings, obviously, it was very clinical lead. So the people that were kind of the forging people were the CNSs and the clinicians, as well as sort of the cancer service direction at that time. So it all seemed very clinically lead at that time, and it seemed to be, you know, have good outcomes. And then obviously, the Manchester paper and everything, then subsequently was published. And they'd obviously showed good outcomes and sort of a framework then of how it can work.

**SB**

Yeah, yeah. And of your time with the project? Have you… are you convinced by it or have your thoughts about it changed any over the time period?

**P5**

No, yes. So I would say, as I've obviously got more invested in the project in the position, you know, obviously, within the Adept Programme, it's one of the key things I was obviously working as part of, but I do think, between the literature and then, you know, obviously, working groups, you know, that we're in and seeing the different specialties that definitely is merit in it, and the outcomes do seem to be better. But I think just and I think I can appreciate that a little bit more, because obviously, I've had a wider look at the literature and evidence base for it than maybe some of the other integral people who are delivering the programme, maybe just don't have that same awareness of how it's working or successfully working elsewhere.

**SB**

Yeah, its the importance of that knowledge and insight, isn't it?

**P5**

Yeah.

**SB**

And how well did you feel prepared for your role in the cancer prehabilitation programme, maybe just talk a bit about your role in that as well just?

**P5**

Yep. So my role was very much and sort of my position has been very much self directed. So the way I've come on board is through this adept programme, which is a isolated thing, which is a leadership management scheme. And so there's eight of us within NIMDTA, which is the training body, all from different specialties, all with their own, all with our own projects. So we choose our part projects. Once we were successfully allocated, you know, to say, yes, you've been, you're successful in your interview for adept. And then we interviewed for you got to rank your projects, and then you get to pick your project and my project was the prehabilitation. So I had I gone into the Adept Programme knowing that was the project that I wanted as it was very clinically based and it was clinically relevant to my working in my work, but my input and my role of what my purpose would be or how I would help with the project or anything wasn't defined, you know, all all I was given was, a sort of role as an adept person? And then I had this kind of I made all the introductions you know, and it's really with the help of [HNC CNS] and with [HEALTH DEVELOPMENT MANAGER], you know, because I've been involved from that sort of health development and with [HNC CNS] being so well integrated and aware of all kind of the different academic and clinical elements then I'd be able to sort of pick out a position. And then it was good timing in that, whenever [HNC CNS] and I kind of met at the start, and I sort of was saying this is who I am, this is what I can do, I had a sort of unique element that I had a clinical background and basis, which is one of the areas at this stage of the pilot they didn't have.

**SB**

Okay

**P5**

so they'd a lot of clinical input at the start. But actually, when you look at the working groups, you know, other than the sort of CNS and sort of specialized physio, the clinical staff, you know, that would be patient interacting day to day, or they actually didn't have that kind of working group setting. And then obviously, I had some academic research knowledge as well, because it's sort of one of the remits within our practice, you know, that we'd be doing service evaluation and things like that. So it was good timing in that then I was able to come on, I said I'd help with any, any aspect, and it's kind of just developed, you know, between different areas that we've seen the project need, or the more that I've identified or developed areas that I've then taken on and said, Oh, I'll have a look at that, or whatever. So I think the clinical aspect has been unique, more unique to me as in I can see how it applies, or how in real time there may be barriers or problems that may not be able to be, I can see why, you know, when I was coming up and saying this patient is being pulled too early out of the programme, because the lists are all ad hoc, at the minute people are getting no regular lists.

So I had a wee bit more insight to why those things that we didn't really know why were happening were happening, because then I knew a little bit more with the clinical background of that, and then because the pilot was up and running, and then we knew we're going to be working towards a service evaluation element, then I took on then more of a role towards the evaluation analysis point of view, because obviously, every most other people within the pilot were employed, or are brought in for a particular role in the, in the running of the pilot, you know, in actual, you know, a measurement or whatever their role would, would be, but I was then able to take a step back as an overseer, not just one particular part of it. Then I became more heavily involved with sort of the evaluation element for the service evaluation, and then obviously, moving towards the regional development side of things. So it was all sort of when I first came in, it wasn't really clear where I would input but then, as its developed, it sort of my basis has sort of been the clinical plus sort of the evidence and academic basis to say, yes, I've had a look at this, I think that's a good evaluation tool or not, or that sort of thing.

And then within the COVID, or within the things that were working well or not working well. So to say, and this is why you know, this is the practicalities of this patient getting to their surgery, this is a good time to catch them or not. And then really the evaluation and analysis side of things. Because I I'm able I'm not kind of an integral measurement, you know, a measurement within the pilot, you know, I'm not part of the screening or referral pathway. So I could take a step back and do that analysis in an overview side of things.

**SB**

Very good. Very good. So you really have had your finger in so many different elements, which is, is brilliant. What are your thoughts on the current assessment and referral process?

**P5**

So I think that has changed over the time period. So I think when I initially came in, and had done more of the evidence and the literature review element I had thought we were doing so little as in I thought there would be a lot more technical, you know tools and, you know, so a lot of my questions at the start without being like the new person who's like, you're doing this wrong, you're doing this wrong, you're doing this wrong. And I say, why are we using this tool for this? Why did we not go with this tool? Or why did we do this rather than this? And so I questions like that at the start, because I was sort of unclear to how we got to some of the screening and referral tools that we were using. so then once I kind of understood that a wee bit more about you know, it was actually really the usability and the time burden and practicality element of things that you know, more access, you know, more that the CNS and MacMillan side of things, it was what they were happy to do, you know, and the techniques they were happy to use, and without giving them you know 20 page questionnaires and things.

So I think it just took a little bit for me just to come step back and have a little look at that side of things that I was like, right, I just need to accept that these are the tools that we're using. And then once I sort of was happy that these are the tools we've chosen and are within the pilot, then I went away and looked at, you know, like, when we realized that there was gaps, and some of the data gaps and things that we weren't capturing very well, then we looked at how we could could we simplify them further. So it did seem to be always trying to simplify and so even then, so at that point, I went away and looked in the literature to see what of 30 seconds at the stand be enough is that a robust enough tool in isolation rather than grip strength and rather than the other metric, psychometric or isometric measurements that would that be enough? You know, if we only asked of one thing of the two time points so I did that, and then came back and said yes, you know, some of the papers out there, that's all they're measuring, and it is a robust enough tool. So we went back and did more booster sessions and sort of education on that side of things.

So I think our tools are as simplified as you would want, to try with the aim that it was meant to be accessible and user friendly to capture and to allow the maximum amount of use for then the, you know, for patients and for the screening and referral, you know, the referral. The screening process is very good in that it's a very clear, well constructed e-referral system and I think that works really well. And with some additional refresher and with additional reminders that has worked well overall

**SB**

Ok

**P5**

It's coming to the referral and the measurement, obviously there's been more challenges with getting into that for a number of reasons. And that can be, you know, patient factors, my impression is patient factors. And then, and maybe the practicalities of that and being more challenging than anticipated.

**SB**

Like in what way,

**P5**

So as in when if patients are only being contacted by telephone, or, you know I think in principle, a lot of this, and what we, what we find out over the time was between the four council areas, there was a bit of discrepancy and inequality with how even as a single move more person within the same organization, the remit and what they were allowed to do was very different. So one of the move more coordinators were able to do home visits, one, you know, one had more access to classes and council things, whereas others were isolated to only telephone or electronic communication with Zoom, you know, so obviously, that was a bit of discrepancy between then what they were able to deliver.

**SB**

Yeah. You mentioned there a number of the components and I suppose some of your frustration in the early days about why those were chosen. Are there any of those components that you feel at the minute are not necessary.

**P5**

So I think I think all the elements are necessary, I think it was maybe just I, my queries are more about refining those a little bit more, you know, so say like the anxiety tool, obviously we've gone through the distress thermometer, which is then you know, we'd had input, I think at the concept side of things from psychology as a valid tool. But my query was when you're when you're dealing with cancer diagnosis and cancer treatment and things you know, like something like this state tree inventory, where you are asking them about their anxiety and distress, but it helps delineate between, you know, their diagnosis, you know, their baseline anxiety, and then the treatment or diagnosis anxiety. So, it just a slightly more refined tool that you know, if you ask someone on a normal day, if they're a highly anxious person, the Distress Thermometer might not be able to pick out that minutiae of is it actually your baseline anxiety and that you're just a very anxious person in any engagement with hospital any engagement with anything outside the norm compared to then is this actually in relation to your diagnosis or treatment? so it was more the refinement and the choice rather than the actual elements themselves,

**SB**

Ok just going back to in terms of your preparedness for your role, which is quite broad without there being, did you go on any training? And would there have been any training that might have helped you?

**P5**

So I think I did a bit of, you know, so I really used the literature for my knowledge base. so that was, again, it was all very self-directed, and the processes and that side of things, you know, I think [CNS HNC] as more of a mentor, and that side of things has been invaluable. So she very much directed me to the right people, and being able to make the connections, you know, with early on, you know, she made the introductions like health development team and that kind of side of things. So I think it was really those early connections with understanding very early who, who was who, and, and the processes of things has really helped. because they haven't been delivering the programme as such the practicality element of it, I don't think I needed anything more. And I think then my baseline clinical understanding of how, you know, the patients come pre op, operative, and post op, then I already had that knowledge. So I was happy, you know, I'm sort of well suited to understand what the normal timeline pathway is, you know, because I've been in training for 7 years, you know, that's well established, and it doesn't really vary, you know, within the trusts or within each hospital, it doesn't change dramatically, especially in cancer, because there's a very defined pathway times and, and treatment time, so it doesn't vary grossly between different areas you work.

**SB**

Yep. And you said that you're not delivering any of this directly with patients P5, no?,

**P5**

At the minute no, no. So I've been really just the prompter, you know, so I've been the person speaking to the CNS, or the consultant, but because my, I'm only doing it out of hours work, it really isn't remit so I've been doing education with junior, you know, like the F ones, the ward nurses, the CNSs and then the consultants who would be the ones delivering the diagnosis and planning the resection.

**SB**

Yeah, have you met any resistance from them as you have been communicating the project?

**P5**

No, I think from a junior staff level, it's very much lack of knowledge. So within this was their first introduction to prehab, didn't know it existed, didn't know what was happening. the CNS is obviously knew about it, because they're an integral part of the screening process of things. And so it's just really been trying to make sure the recruitment from the shared vision point of view has been there. And that's taken a lot of reinforcement. And then the clinician side of things is variable. So the prehab concept meetings had a lot of clinician representative, and I don't think that has really factored through necessarily to be an active engagement now. So I think it's very much my feeling overall, is this is very much driven from the CNSs who would be meeting the patients duly with the consultants, you know, so if they're there with diagnosis or at their surgical planning, they'll meet both their consultant and the CNS on the same day. But I would say, it'll be the CNSs who are introducing the concept of prehab. And they're the ones following through, you know, with the information pack and thereafter. And that was one of the early learning points that I'd said about, you know, patients may be more likely to engage if it's prescribed as their management from their actual operating consultant or their management consultant, you know, if it's coming as we would, it's an expectation of their role in their treatment, and it was coming from a consultant, I think, or or Reg or whoever they're seeing at clinic, I think that would have more of a patient impact, and I think that's one of the areas that could be improved was my impression of that.

**SB**

Okay, so that isn't common practice then you think it's more the CNS.

**P5**

I think so. They will have an awareness of the of prehab and I would say, but I think it's variable to how much they either think it's worthwhile and or there, it's even on their on it's not even that they have a personal or professional feeling of it being useful or not useful. It's even just being on their on the radar as this is something that these patients are engaging with.

**SB**

And that's actually a question that I will explore maybe throughout these interviews. What impact do you feel that COVID had on the Cancer Prehabilitation Programme, from your point of view,

**P5**

So I think things have been positive and negative. So I think positively, there's a drive for innovation, you know, so I think that COVID has allowed there to be development and innovation with different working groups and these and health development teams and things that wouldn't have been there before, you know, a lot of these pilots and different things would have been coming to fruition a lot later. but obviously, there are many kind of challenges it's imposed. And I think probably the key ones for us have been the delivery of the programme. So patients how they're actually being able to engage, because if you think back, if even as you look through the pilot, there's multiple stages of peak, you know, pandemic peaks, and lockdowns and things. So, you know, a lot of the impact is, you know, patients are being told to isolate, they're worried about it jeopardising their surgical treatment, you know, they didn't want to go to leisure centres or see people they don't need to see. you know, so there's been a lot of that kind of impact in that patients just want to make sure they don't get miss their treatment. so therefore, anything over and above that, they don't want to risk and also then in how you deliver it. So obviously, a lot of the time, then you are minimizing face to face. So if it wasn't able to be delivered over Zoom or your home DVD, then obviously that's another implication.

And then obviously, we've had to adapt with the guidance, you know, the patients are obviously being swabbed and at different stages, you know, so if you think about a pathway, a patient's been screened, they've been referred, they might have the time one, but then they're getting swabbed and told right your operations next week, but you have to isolate for 72 hours before your operation, you know, so it's adjustments within the COVID guidance at the time to keep them on a green pathway for their treatment. And then I think the biggest impact is the time in prehab. so if they're being screened and referred, the implication of that is that your treatment is usually within the next two weeks, but because of sort of the changing picture of COVID, consultant lists, like consultants used to have their list planned for two to three months in advance, you know they can see every time what their list would look like, you know, the next month, and but with the sort of ad hoc nature of lists, then being released, then patients could be called up with two or three days’ notice, and out of normal practice historically, but within COVID was very, it's very normal practice now.

**SB**

Yeah. And in terms of data collection, how do you think that's been impacted by COVID.

**P5**

So we think this sort of electronic format is probably very good, I think that has kept that and I don't think COVID has had a significant impact on that element of things. I think from the referral data, it's made it slightly more challenging to complete the functional element of things, just because a lot of that would have been ideally in a face to face. And obviously, the nature of a functional or physiological assessment is much easier, one on one or face to face, so I think that's been probably the biggest impact

**SB**

In terms of your professional development and practice, what benefits has there been to being a part of this Cancer Prehabilitation Programme.

**P5**

So I think two-fold from colorectal being one of the tumour groups, you know, the fact that I have been part of the pilot and have the evidence base knowledge of how it can be successful, you know, I personally would be promoting prehabilitation for my patients going on in future. And I think, from a leadership management side of things through the which is the point of my year has been actually rather than the outcomes of the pilot, but the process has been invaluable, you know, so actually being part of these kind of working groups in the multi multi disciplinary and multifactorial kind of level working to meet the challenges and in a timely way quickly analyse that and adapt and change our process to try and then mitigate the impact on the pilot has been really good.

Plus, then working with people, the challenges that faces and the different aspects for that have been really, really good, you know, I really enjoy all that side of things so even though as I was saying at the last group, you know, instead of just working on outcomes, and sort of, even though we want to have as good and clean data and outcomes that are actually tangible, but actually, the process of this all there's been a lot of learning from, and I think that's learning that isn't necessarily isolated to the pilot, but for me, in a professional way to how I work with people in future, or if you were to carry out another sort of evaluation or or plan for service provision that you would take forward, so because obviously, I'm early in my training, or you know, or career pathway of, if you look at it, you know, I've been working for nine or 10 years, but I have another 30 years, you know, to go so so actually, these are skills that I can use longer term.

**SB**

Yeah, yeah, absolutely. And you mentioned earlier, just the opportunities to share with other colleagues and, you know, sharing about the programme and the importance of it

**P5**

I would see myself as one of the, you know, the champions of the project in the clinical setting. So you want to show that the work that you're doing is actually impacting on the patients, and then therefore, get more buy in for then this to be something that is sustained, but also that then they're going to advocate and buy into for each patient, then.

**SB**

Yep, yep. And just on that, in terms of sustainability of the cancer prehabilitation programme, what improvements do you see needing to be made to this programme? For south eastern trust but also in terms of widespread implementation,

**P5**

So think, really, I think we have the, the framework is good, I think it requires a lot of sort of background working so it needs an a really good support team, you know, from the data capture and analysis and troubleshooting element of things, you know, so it actually takes a lot more sort of data capture team than even I would have thought, you know, that is responding to obviously the screening and making sure that they're directed appropriately, and the referrals are going out. And then I think the big thing rolling out is just making sure that there's that community provision, you know, for these patients are being enrolled and then potentially referred on, but that they're being picked up appropriately in a timely way. And you know, within the move more community, as this would get bigger is that they have adequate training, I would very much feel there's a bit more training to be done with the move more side of things, just from a not their baseline obviously, their move more capabilities are well established.

But I think just from a understanding of maybe a bit more of the clinical application, and, you know, when I first came into the programme, I my understanding was I thought that move these move mores that were involved with our programme were all baseline physios, or, you know, I thought their training was much more of a health care, physio background, rather than from the MacMillan element of things, so obviously, they've gone through all their training and their MacMillan side of things, but I think there needs to be a wee bit more that is specific to the prehab side of things that would be helping them with patients that are more complex, you know, that have multifactorial comorbidities, and that aren't special, you know, they fit specimens that with many a person with a cancer diagnosis that, you know, have many other considerations, whether that's social or physical, or their comorbidities.

So it's what then they will be happy to do within that. And then I think the other big push is that we're really making the physical element of things functional, much more prescriptive. so yes, as the universal side of things works with, you know, obviously they'll get their smoking cessation and their alcohol which works quite well. And then the move more obviously delivering on the universal more than just the physical prescription you know they're offering the psychological support, and also dietary advice and support. So they are very broadly you know they're doing a lot. but it's just that then the physical side of things, I think does need a wee bit more thought in that these patients are complicated. And, therefore, one prescription isn't going to fit off all patients. But they need to be prescriptive in that the patients do need to be pushed outside their comfort zone and recruited rather than being you know, a lot of it is self-directed, and they're sort of left then to their own comfort zone, you know, they're still kept in their comfort zone of what they're happy to do, you know, walking twice a week, or whatever it is. So I think it's a descriptive side of it

**SB**

Do you think the move more coordinators currently involved in the programme could they have been supported with more specialist sort of role or is it just that they needed more training, or is there…

**P5**

I think a little bit of both but I would say that overall throughout the programme they have been there is a very support offer there, you know whether that’s with clinical concerns or within their own capacity if they think they’re overwhelmed from a referral point of view so I think the support has been there through the pilot and

**SB**

In terms of like actual physio or exercise specialists

**P5**

I think that’s something we could do with a little bit more of how to prescribe for complex patients you know that someone working day to day in a very clinical field with you know whether that’s a rehab setting from community or that is an acute service I think it’s more that confidence just to be able to push on patients that have complex needs, and that they can do that safely.

**SB**

Any barriers for long term delivery of the cancer prehabilitation programme that you can see?

**P5**

I think one of the significant things would be funding, so obviously anything to be sustained needs funding and I think it’s that… I think our issue is going to be the workforce, so I think the concept buy-in regionally so that needs to come from the clinical element I think and filter down, so I think you have to have the clinicians that are going to be giving the diagnosis and first interacting with the patients need to want their patients to be you know referred on to these programmes, and then it’s really for the wider community, the CNSs are already different groups are being overwhelmed within their normal clinical tasks or their own normal tasks, so I think it’s the provision that they’re taking these roles on that it is manageable within their current roles and demands, and then when you’re moving out to the community that there’s obviously enough manpower and provision in the move more if we’re broadening the different councils as it moves out regionally that obviously they’ve enough bodies to deal with that. Then we need the data and the backup and admin of the data backup of that to be able to assess and capture the information that’s being caught.

**SB**

And which of course goes back into the rationale for it and making people see how important it is and yeah, really important.

P5 thank you so much. Have you any final comments you want to make about the cancer prehabilitation programme?

**P5**

I think well I haven’t been involved in many pilots but I do think it is a very well thought through and structured programme and it has good evidence basing and there are successful models that have worked ad I think our model has achieved a lot of success but here are probably a few tweaks form the analysis side of things that could be improved on that will then help that regional rollout, the recommendations and the learning from that so I think even though there have been elements that have been challenging or haven’t worked just quite how they have been thought from the concept, I think that’s valuable learning that then we can adapt and create a model that has practical and clinical application would actually work successfully

**SB**

And what if you were to name a few of those tweaks if you wanted to see a few of those priority tweaks, what would they be?

**P5**

So I think from the outset you would want to make the concept kind of I think needs to be really patient based, so I think you have to recruit all your vital stakeholders, your CNS your move mores, they really need to understand the point of it to know why their input and their role is going to be so important for these patients, I think it needs to be more prescriptive as a management plan and that needs to come right from the beginning from the consultant level and from the CNS and that needs to be prescribed to aid the patient engagement and recruitment and then from the actual outcome analysis we really need to be getting our functional assessments so that we can then show improvement in sustaining the programme and that will then obviously buy into further investment with workforce and finance to show that the programme is working, but the only way we’re going to show it’s working is to show better patient outcomes both functional and psychological, and we have to capture that in an accurate way so I think it’s just reinforcing that we have accurate full data to show outcomes for longevity.

**SB**

Thank you so much, I will stop the recording

# P6

**SB**

That's all ready to go. Okay. Just to begin, if you think back to the start of this project, what did you know about Cancer Prehabilitation? Before the programme in the south eastern trust?

**P6**

I would say a reasonable amount, because we were already getting referrals from the head and neck cancer service. And we were, I suppose, providing that that service, for a couple of years, maybe before in relation to assessing patients as to whether they needed to come in, you know, to pre optimize their care, usually for detox from alcohol prior to surgery. So yes, I did have an idea about it.

**SB**

Okay, okay. That's ... so interestingly then components of this Prehabilitation program were in existence before the programme itself.

**P6**

Yes, yes.

**SB**

Okay. And what are your thoughts on Cancer Prehabilitation for patients? How important do you think it is?

**P6**

Well, I suppose from our point of view, if people are going to be admitted for major surgery, and they're alcohol dependent, it's, it's vital. Because if those patients are not properly assessed, and there isn't a treatment plan in place, you know, to manage any detox issues. I mean, if somebody pulled out say, somebody went into severe withdrawal following surgery, and pulled out their trache potentially they could die. So I would say in that case, it's, it's quite vital.

**SB**

Okay how well did you feel prepared for your role in the cancer prehabilitation in terms of the alcoholic liaison service role?

**P6**

Again you see because we certainly with the head and neck, because that was something we had been doing. Now some of the others, I would say it's more problematic. The lungs and bowel, but that's, that's more to do maybe with with I can come on to that. But that's more to do with trust boundaries.

**SB**

Okay. Okay. Can you tell me a little bit more about that?

**P6**

Yeah. Well, you see, we get maybe we would maybe get referrals from cancer prehab, maybe more for respiratory patients. And those patients aren't coming to the ulster hospitals. So therefore, we are having to rely on phoning them, which we find sometimes it's not ideal because people can tell you a lot of things over the phone, but if you see them, you realize maybe you know, you're not getting a clear picture. Those people aren't coming to the ulster and they're not coming to the ulster for surgery, either. There may be, although they're South Eastern trust patients, because I know that was the criteria, but they're not coming for that reason. You know, they're having their surgery in Belfast trust and we don't cover Belfast trust.

**SB**

Okay. Okay. So there ... that sort of limited your ability to?

**P6**

Yes, it did. Because I mean, we we weren't able to offer detox advice, we wouldn't have been going to see the patients while they were in hospital because we don't work in that trust.

**SB**

Okay,

**P6**

So that, and the fact if any of those patients had been coming up here to a clinic, we would have done our best to see them. But some of them, some of them weren't. They maybe what used to happen, say for example, they may have been up and seeing the Macmillan nurse, but then by the time we got the referral, there were no plans for them to come to clinics in the Ulster it was all going to be in Belfast.

**SB**

Okay. Okay. And just while we're on this topic, do you see what would be your thinking on a project like this moving forward in respect of that?

**P6**

You mean,

**SB**

What do you think would be important to happen in order to?

**P6**

Yeah, okay. Well, you see, one of the issues is not all the trusts provide the same substance misuse liaison service. And Belfast trust, for example doesn't really I mean, they have two nurses who work specifically in ED, so with patients in ED, and with patients in the liver ward and that's their only remit, so they wouldn't accept any referrals.

**SB**

Okay,

**P6**

They then have a team which is called mental health, mental health integrated liaison.

**SB**

Okay.

**P6**

But they they have one nurse who did our job in the past. And she's retired and she works one or two days a week to cover the whole hospital. And unfortunately, our experience of these liaison teams is that the addiction that the specialist knowledge gets lost, and they really function like a mental health crisis team. I would doubt if they would accept referrals from cancer prehab they wouldn't see it as their remit. But obviously that would be something you'd have to ask them based on how I know how they operate. I doubt that those that they would see it as their role to be involved in this.

**SB**

Okay. Okay. that raises a very important point in terms of moving forward then, about ensuring that there's consistency across the trusts, then that's very interesting. So for you, you, you're you had experience then in terms of the head and neck patients you had been receiving referrals previously; for the lung and colorectal, had you been receiving the same referrals?

**P6**

No, no.

**SB**

Okay. And did you feel okay about that? Did you feel prepared to take them on and?

**P6**

I mean, I think so. Because I have to say that I mean, our referrals through the cancer prehab would predominantly still be head and neck patients, I suppose I think maybe partly, that's due to you know obviously, people who you know, drank heavily. And I know we're not smoking cessation, but you tend to find people who drink heavily, a lot of them are heavy smokers as well. So the two together greatly increases the risk of developing an oral cancer, whereas maybe the other two, maybe not as much, because we wouldn't get as many referrals. And we have seen, I mean, there have been some, I think some of the ones that maybe with bowel cancer would have been seen here. But it's predominantly predominantly the head and neck.

**SB**

Okay

**P6**

And they're maybe more complex because of the nature of the surgery. So we would have been maybe a bit more concerned, you know, I think the risks are higher with with them in terms of withdrawals than maybe the other group of patients.

**SB**

Okay. Okay. And can you tell me how involved you were in the planning of the south eastern trust cancer prehabilitation pilot?

**P6**

Yes. I met with [HEALTH DEVELOPMENT MANAGER] and another girl - hold on I’ve just got a folder here - and think I had maybe gone to initial meetings, and then I was asked, you know, to obviously be part of, you know, to offer what our service could offer. So, yes, I did feel that I was involved in that, yes

**SB**

Okay. I'm sorry, I missed that last wee bit there

**P6**

There were a number of meetings, yes. With, yes.

**SB**

Yep. and were you able to shape the referral criteria or process at all?

**P6**

Yes, because I was asked, what would what would be an easy way to do it? And I was able to give advice on the audit see, which is basically a tool to... I mean, it's, you know, it's not totally accurate. But I mean, it's a tool to assess somebody's, you know, level of alcohol dependence or whatever. And that was, that's how they do it. It's basically the person's asked, you know, how often do you have a drink intake you know, of alcohol? How many drinks would you have on a standard day? How many, how many times or how many times a week, would you have more than six drinks? And obviously, if somebody's scoring 12 out of 12, the likelihood is that person's probably got a physical dependence on alcohol.

**SB**

Okay. And, in terms of the pilot, were you in receipt of any training specifically for the prehabilitation or was it just through the meetings and information sharing?

**P6**

*inaudible* on this standard operating procedure but no, no training, no.

**SB**

Okay. And on reflection, do you think there's any way you could could have been better prepared for the Programme?

**P6**

Not really because, like I say it was just formalizing, and certainly with the head and neck what we were already doing.

**SB**

Yeah, yeah, that's okay. And in terms of then, you were saying there that for the assessment process you use the audit see? When did .. can you tell me a wee bit about when you received your patients in terms of the referral? And when that assessment was completed? And did you do that assessment or did the referrer?

**P6**

Yes, the referrer would have sent I know, there wouldn't have been a huge amount of details on the referral, we would have got, like, for example, I've got one here this morning. So we would get the patient's name, Health and Care number, date of birth, address, contact details, obviously, the referrer's details and basically, it's just the audit see. Although, the girls would maybe send an email and say, this person has been drinking whatever amount. So then what we would do is we would go through on the systems like ECR, we would contact I mean, we can see the mental health notes in the south eastern trust. So we would check that system to see if the person had been ever known to addictions, if they were from another trust, we would contact the other trust to check. You know, if they had, we would look through I mean, you'd basically look through everything you look through what medications they're on, all hospital admissions, and had they had detoxes before their bloods, their ultrasound, so we'd look at all of that, and have all that information. And then usually, we would maybe contact the patient by phone, initially, but then we would arrange to see them and usually with the head and neck patients, we would either see them at pre assessment, or the max fax clinic.

**SB**

Okay. Okay. And that pre assessment, when did that happen in the process before...

**P6**

Well, I think that's where they come up to be assessed, if they're fit for surgery? Where they would see the anaesthetist, so we would go we would go up there then and do an assessment at that point.

**SB**

Okay. And that potentially, then could have been quite a short time period between you seeing them, and then maybe surgery.

**P6**

Yes, maybe a few weeks, and then we would we would follow that up, you know, we would see them again, closer to the time and then we would have a plan in place about I mean, we would have decided, usually after the first assessment whether the person needed to come in early or not. And we would always err on the side of caution. And then we would maybe decide how early they would need to come in. You know, there's all kinds of factors like for example, if you have somebody, probably a lot of these patients, because they have been drinking so heavily, will have other comorbidities like for example, if you've somebody has cirrhosis of the liver, you have to be very careful with when you're detoxing them, so that can add to a lot of complexity as well. So we would usually have seen them maybe more than once before they would...

**SB**

Okay, come on. Okay,

**P6**

And we try to see them face to face, because we have had incidences on the telephone where people say, Oh, no, I only drink a bottle of wine at weekends. And then when you see them at the clinic, it's quite obvious that they're drinking a lot more than that, you know?

**SB**

Yeah. It's a very integral, your role is very integral then to the the prehabilitation programme, you can see how important that is. Did you face any challenges with a screening referral or consent process?

**P6**

Yes. What I would say sometimes, and it still happens is we're getting people referred through. But we're not being ... we're maybe not totally privy to what, what, you know, like, for example, I had people referred to me who were then told, you're not getting surgery, and your your prognosis? I mean, you're not going to survive. And I think sometimes that's maybe a bit unfair on the patient of us turning up, you know, because if somebody is basically going to be dead in two months, I mean, is it really appropriate to start going on at them about their alcohol use if it's been a big crutch in their life, and then the other ones sometimes would be that we wouldn't be party to what the patient has been told. And you're kind of not wanting to sit do you know, I think sometimes it could be better that we could we could be better informed about what they're aware of. Because sometimes the referral comes through looks as if the patient has already been being told what the plan is, but they haven't like, and I even mean like surgery. So we're being asked to come and sit when you come and see them. And we're going to see them knowing that they are going to get surgery, but the patient might not know that. So I think the communication could be a wee bit, you know, maybe tightened up a bit that way.

**SB**

Okay. And what what way, would you like to see that happen?

**P6**

Well, I think before we would go in to see a patient, we would need, I suppose just to be told, maybe I didn't know. Or maybe it would be better if we saw the patient after they've seen the consultant and started because sometimes, and I do appreciate busy clinics and time pressures, and sometimes it's suits that they'll say will you go and see them first. But that's not always the best thing. So I think maybe it's the timing of when we would see them.

**SB**

Okay

**P6**

*inaudible* person that's going to give them the information.

**SB**

Yeah, so that's really actually important point here. So that it would be helpful you think, for alcohol liaison to see them after their consultant appointment. Okay. Has there been any helpful aspects to the screening, referral and consent process that you have encountered?

**P6**

Helpful aspects?

**SB**

So, for example, how the referral is received? do you get inform is, is there pieces of information? One of the sort of thinkings here is for some people, the online form can be beneficial? but maybe that doesn't apply to you in your setting?

**P6**

When the referrals do come in online, that's how we get them. But I mean, I suppose the thing is, there's not much information on the referral, but then we're not expecting people, you know, we're not expecting the people referring to be doing a big assessment. You know, so we're able to I mean, we can look that up, obviously.

**SB**

Yeah, yeah. And there seems to be, there is there's a number of various different assessment tools used? Do you only see the audit see? Or do you are you able to see those other outputs?

**P6**

Well, we would see the audit see. I mean, if we put the patient's health and care in we can then see on ECR their, their whole medical history, their presentations to hospital, their blood results. Obviously, if there's any pre if they're known to addictions, but we would have to look all that up.

**SB**

Okay. Right. That's okay. And how have you find in terms of patients interest level of, you know, engaging in the Cancer prehabilitation, and I suppose that just speaking from your point of view from the alcohol liaison

**P6**

We have found that patients are quite anxious about seeing us, I think they think we're going to be lectured or worst case scenario, I think some of them think that they will get surgery if they admit how much they're drinking. And so the first thing we have to do is put their mind at rest, and explain to them why we need an accurate idea of what they're drinking. And it's to do with safety, you know, and we do offer the patients, but I suppose if somebody comes in for major head and neck surgery, it's not always I mean, we give them the information. But it's not, their focus is probably more their anxiety about their surgery. And we, our role has kind of started, maybe to be cause we're seeing them every day, checking their detox, it's given giving them that bit of support and you can do the brief intervention as well. And you know, talk to them about the alcohol.

And now, we wouldn't get many people agreeing to be referred on to services in the community. We tend to not get so many agreeing to that, but we do give them the information. And we do signpost them. And we do explain what's available and what it could offer them. So they do get that but I don't know whether it's just because the focus of what's happening for them, the very immediate thing is the surgery and the recovery from the surgery and even though they may have got there because of the alcohol do you know what I mean, it's not their total immediate focus.

**SB**

Yeah, yeah. So I suppose what you're saying there then is that treatment is their main focus and addressing some of those alcohol issues isn't but do do you find that they engage with you for the purpose of getting them to the surgery?

**P6**

Oh, yes, they do. And they they engage, and that's a good opportunity, then if we are going to see them, you know, every day prior to the surgery, the detox, then they will, you know, we we would bring it up and we have just I mean, you have to be realistic. I mean, some people, yes, they're happy enough if it's in relation to the detox, but they're not that keen to maybe to discuss or acknowledge the alcohol. And I mean, there are people who just aren't planning to stop drinking, unfortunately.

**SB**

Yeah. Yeah.

**P6**

I mean, we would do motivational interviewing to try to move their motivation on and again, I mean, I suppose what might be a bit missing for us is we're not we don't really know what happens, whether those patients stay off alcohol or not.

**SB**

Okay. Okay. So how, at what point do you stop engaging with them?

**P6**

Well, you see we're a hospital based only service

**SB**

Okay.

**P6**

Whenever that we wouldn't be seeing them when they're, and that's the purpose of signposting them to the services that are available in the community. But we wouldn't, we wouldn't see people after, I mean, you see them obviously as outpatients in the hospital, but we wouldn't we were not a community base, we wouldn't be seeing them after they've been for the *inaudible* .

**SB**

Ok and then if they're, if they're not willing to have that onward referral to addiction services or whatever, then that's not being, yeah, the journey is not continuing. And what impact has COVID-19 had on your service provision in relation to the prehabilitation

**P6**

Well certainly start it did because a lot, I mean, people just weren't coming in. You know, and we noticed a big drop off in the referrals. Certainly at the start anyway. And then maybe in the second wave but then since that it's steadily, steadily increased. And I mean, we've, I suppose with with COVID in general, the first months of it, we were not really seeing people face to face, but then and we were contacting them remotely, but I don't think we got any prehab referrals at that time. In fact, we didn't we didn't *inaudible * I would say probably not very much and we've seen, you know, we've continued to offer the face to face to those patients in the detox advice. And

**SB**

Has there been any benefits seen with COVID-19? And in terms of you did say there about telephone contact with patients, but that wasn't going so well, that was there any benefits you did see as a result of having to change practice or anything as a result of COVID-19?

**P6**

No, not really in relation to.... I mean you need to be seeing them and you need to have the input so no, no,

**SB**

That's, that's no problem. what about then benefits to you in terms of your personal or your professional development and practice as a consequence of being involved in the cancer prehabilitation programme?

**P6**

Yes, well I think it's actually been, it's been a very good learning in managing people with a lot of complex comorbidities. And I supposed we've learned obviously, I mean, that wouldn't have been, certainly wouldn't be my field, I wouldn't have known very much about it. And I suppose it's been part of that multidisciplinary team and, and, you know, I mean, we wouldn't normally maybe be having anything to do with anaesthetists and we would with dieticians, because a lot of the alcohol patients you know, their dietary intake's poor in many but yes, I think it has been a good learning for us and I think it would be a very good learning say for any new staff coming into the team because it's quite different from what a lot of addictions would be doing, you know, in terms of the physical aspects, but then you're always going to have the psychological and again these patients you know, we're seeing them and you know, they probably feel like they've been hit by a bus and it's even being able you know, because they've maybe got a very devastating diagnosis. So you have to be really careful how you approach it you know, you cant just be running in gung-ho shouting you have to stop drinking, which there's no point in doing anyway because we discovered because it doesn't work that way. So it's being sensitive to the person's needs as well but trying to build up that relationship. And I suppose what we probably liked about it one of the things with liaison as you know, you you don't get much time maybe with a lot of our patients I mean a lot of people we maybe only see them once you know, whereas these patients, you're seeing a lot more of them, and you do get a chance to build up the relationship then.

**SB**

Yeah. Sorry,

**P6**

Which is important for, you know, to, to be able to give them the support.

**SB**

Have you enjoyed this aspect of your job?

**P6**

Oh, yes. We all have. Yeah. Yeah.

**SB**

Is there many in your team P6?

**P6**

There's four of us.

**SB**

Have they all been involved in the project

**P6**

They've all been involved, yes.

**SB**

Very good. Well what improvements and you've touched on this slightly at the start, when we were looking at that case at the start, what improvements do you think could enhance the sustainability of Cancer prehabilitation Programme? So you'd mentioned consistency.

**P6**

There's just such a lack of continuity across the trusts in terms of the service that we provide. And even for the teams that still are working in like, give you an example, the Western trust, and I know, there's obviously there's cancer service in Altnagelvin, they've one nurse, they've one person on their team. And they're not there's no way they would be able to provide. I mean, you know, and that person has to have leave. And whenever they're on leave, there's just no cover. So that I mean, and it's the same, there's one nurse in Altnagelvin, and one nurse in SWA And then this I told you about Belfast trust, and then the northern and the southern trusts, theirs is just like a mental health liaison service, I don't think they would accept. So I think it could be quite it, I think it would be very, very challenging. I don't know how you would extend it outside of the south eastern trust.

**SB**

Yeah, yep. Yep, for alcohol liaison that's going to be a major barrier moving forward. What do you think.. what improvements could be made to this programme, in your opinion, so increasing staff numbers, in terms of alcohol liaison, anything else? And you also had touched on about the timing.

**P6**

The timing would be good. And I do think it would be good. See this, again, I don't know how you would do this. I mean, we don't we don't have really any admin support. So we, we don't have time, but it's just to see, you know, I think there's maybe a lack of certainly on our part, a lack of knowledge as to how the the people did after, did they manage to abstain from alcohol? Do you see? And I don't know how you would how you would do that? Because then that would have to be resourced, because we wouldn't have time to do it. But if it's ... part of you would like to know, and I know a lot of ours is focused on, you know, the detox, but and that's great, because we can see that and we know that. But then it's afterwards. That's the other bit of it that we're maybe we're not getting.

**SB**

Yes. Just capturing that at a later time points. And that will be interesting. I'm not sure about what all sorts of data will be collected in terms of the patient evaluation, I'm not involved in the kind of quantitative data side of things. So it'll be hopefully there'll be some outcome measures that will be of interest.

**P6**

That would be helpful.

**SB**

Yeah. Very good. Anything else you want to add [PARTICIPANT NAME]? I don't have any sort of more areas of questioning. But is there anything else you'd like to share about the programme, that I haven't asked about?

**P6**

No, I don't think so. I mean, I think it works very well. You know, I think it is something that I mean, we've certainly enjoy doing and we've had some very complex patients. And, you know, working through that, I think it's and I think I do think the patients benefit from it. Now, that was one thing during COVID actually, that probably did maybe make a difference. Because people were were maybe coming into hospital. And they were maybe being brought in seven to 10 days before their surgery. And they weren't allowed visitors, and because we were able to go in and see them and we weren't, and I know obviously they were being well looked after by nurses, but a lot of the you know, we were able to go in and spend a better time and get to know them. And I think that was good for the patients as well. It gave them that bit more support. Because then when they didn't have their families coming in and they were waiting to have major surgery, I mean, it's a very difficult time for them, you know? And I know it had to be that way. But, you know, so I think our role during COVID maybe was even more important.

**SB**

Yeah very interesting, thanks for sharing that. Yeah very interesting. It is interesting listening to the various different roles about how they’ve found the benefits and drawbacks of covid, and there have been some benefits.

**P6**

Oh yes, I know. I mean in other areas of our work there’s maybe been some benefits but definitely not with the prehab patients.

**SB**

Yeah, ok well I am just going to stop this recording

# P7

**SB**

I'm going to start the recording. Fab. Okay.

**P7**

So I have a fan heater on. Is it too noisy? Or can you hear me ok?

**SB**

Yeah, no, I can't hear it at all. So that's perfect. Yeah. So, P7, what did you know about cancer prehabilitation, before the service started at the South eastern trust?

**P7**

Awk I was aware that there's sort of some of those things, emm I worked in Scotland for a period of time in 2014. So there's a bit of prehabilitation stuff, or you know, early, enhanced recovery and sort of bit of prehabilitation that I had come across in surgical journals, but no, not a great deal.

**SB**

Okay. And from what you did know, to what you know now has your perception changed over time?

**P7**

It has

**SB**

okay, in what way?

**P7**

Emm there's probably a better, there's certainly a structure to it a bit more sense, you know, in terms of what, you know, the difficulty always was, was how do you how do you implement the change for these individuals? You know, because there's a lot going on, and so it seems that there's a clear pathway to tap into I suppose.

**SB**

Okay, and what are your thoughts on cancer prehabilitation? Do you think it's important to engage patients?

**P7**

Yeah I do, no, definitely, definitely. I think it's such a difficult emm, you know, whenever people come to see us, you know, you're you're turning their world upside down. You know, it's bad news, you know I've family members had cancer as well, you know, so. So there's a lot of emotions going around with everybody. And, I think that sort of empowerment, you know, emm, you know, and helping them to control it, because they feel they have a small part to play, it doesn't work for everybody, but there's certainly some people who can see a benefit to that, you know, a lot of times it's really the specialties or, you know, you're you've got this investigation, you got to meet this person, you've got to, you know, so so it's always the hospital, a hospital, a hospital, Whereas I see a wee bit of empowerment, I think helps a bit more positive coming into the ward, you know? If that makes sense.

**SB**

Yeah, that sort of sense of control, maybe that they have a little bit of control over the outcome. Yeah. What has your role been in the cancer prehabilitation programme P7?

**P7**

Emm I miss signposting it, I suppose a wee bit, I was involved with some of the meetings early on. And eh, but but really, I just, you know, I just facilitate probably.

**SB**

Okay. So, you don't you're not participating in the assessment of assessments? No, no. Okay. Okay. And emm did you receive any training as part of your involvement in the prehabilitation pilot? No.

**P7**

Not really no.

**SB**

And is there anything that could have prepared you better for your your role in terms of signposting on or

**P7**

I think it's emm its what would be useful for me is having... a lot of it comes from from [HNC CNS NAME] and [HNC CNS], the CNSs and directing people, but certainly for your own knowledge. It's, it's good to have a.... it would be useful for me in conversations with patients, you know, I have a good understanding of it, that I'm, you know, I can bring that into the consultation, you know, because it because the consultations, you're trying to fit in a lot of information and try and do it in a concise manner, emm try not to overwhelm them. So this is sort of that that would be the only thing, be useful useful for me actually, just just sort of, because I'm not that that sort of Macmillan pathway and all that sort of appreciation from speaking to [HNC CNS] and [HNC CNS]. You know, it's not something I fully understand if you know what I mean because it's not it's not really my area.

**SB**

Yeah, with so you feel like would have helped you better to know that more clearly.

**P7**

I say, I think so. I think as a surgeon, I think it I kind of put myself in the place, it's easier to describe it or sell it if you sort of know it, now a lot of it is that there that I think that would be useful because it makes your consultation more genuine, you know?

**SB**

Yep, yep. Okay, um, in terms of then you signposting patients to this programme, when does that happen P7?

**P7**

Very early. So the first consultation is often bad news and you're kinda trying to get a sense of things. It's really only by the second possibly the third consultation and probably by the second actually, at that stage you sort of have a clear idea if they're going to be going for surgery treatment emm and that that's the time you know, that's where you're sort of trying to empower them, and you know, start getting them prepared for this. So that then in around that time, so the way I would normally work is patients come up to us and you know, we'd see them and just say, look you know, this is this is you've this cancer, this is, this is the type of treatments we would offer, you'll meet with the anaesthetic team, there'll be a bit of discussion with the allied health professionals and CNS girls, and then then sort of coming back to me, it's really just to decide how they would like to proceed, you know, so that's probably the best opportunity. And we've had a plan in a few weeks ahead, so.

**SB**

Okay and what's the timeframe between that initial bad news appointment and the what's going to be the plan appointment?

**P7**

So, it could be a week or two weeks it sort of varies. A lot of it depends on stages, some of them, some of the people have been sent to me, I've had the bad news conversation, but what do you find is that whenever they come up to see us, emm you know, you're discussing through scans, you're trying to just get a sense of where the patient's at you know, so I keep it very emm, I keep it quite broad, I sort of just say, like, you know, there's a surgery option, there's a radiotherapy option, see how their consultation's going. Emm you know, if they feel like they want more information, I'll give them a bit more information. You try not to overwhelm them, because you're, you know, that people are coming to these appointments, they're very apprehensive, you know, they're up and first time they're meeting you, you know, so there's a whole lot of things going on. So we, we kinda just keep it keep it in sound bites, small, small amounts of information, just let that filter in. And now I say I've heard [HNC CNS] and [HNC CNS], sort of talking about that, you know, the prehabilitation and that probably that first consultation. It kinda goes in I don't know if they take it fully on board. But like, it's it's certainly you know, all of these things are useful. They're all good.

**SB**

Yeah. Yeah. And I suppose the earlier those conversations happen then there's a greater length of time between the intervention starting and surgery?

**P7**

There is, there is, it is emm and we do do it, probably doing it, we're probably getting better at it. Um, you know, that there emm as you become more aware of it. Yeah,

**SB**

Yeah. And maybe for other projects, or when this has been rolled out to other trusts that's maybe some learning, P7, that has taken time.

**P7**

Yeah, no, no, definitely see, so so like, you know, you go back to a few years ago, you know, this wasn't part of the conversation. So I suppose that that's kind of where I was sort of thinking, you know, if you even if you can, I sort of have a sense of what they're doing. But if you know what it is, that entails, it's easier then to say, look, it's easy to add into a consultation, you know, and earlier in the earlier in the pathway or in the journeys definitely better. There's no there's no doubt about that, you know

**SB**

But it's that balance then with where the patient's at and being very aware of that as a clinician.

**P7**

Yeah, I am just always conscious of I, I have a sort of how I'm delivering a lot of hard information, you know, that that's the thing like I'm, you know, so I've had very different role, and they approach me very differently than they will, you know, whether you.. CNSs you know, they'll have a different conversations and stuff. You know, but, uh, certainly, it's certainly empowering people and sort of having as part of the, you know, some people will say, you know, we're gonna fight this we're going to do this. So I think I think you can kind of you latch on to that and use that.

**SB**

Yeah. Which yeah, very interesting. And how you have when you have introduced cancer prehabilitation into conversation, how interested have patients been?

**P7**

So far, so it varies from patient to patient. Some of them emm ya know, they'll sort of say stuff. It's hard. I've only done a couple of occasions so you know, so I don't have a great sense of it, emm some will respond well, others won't. Or you know, you can almost sort of tell by the demeanour of the person you're talking to you know, and you'll have you'll have the younger or the healthier folk, you know, are coming in and they're well kept and they're they'll go for it, you know, whereas you've other folks who aren't in great shape and it's just it's just adding to another list of problems they have you know, because they're coming in with medical issues, and they're not fit you know, so its sort of a wide spectrum of patients. That's where so that's why sort of you judge it, each consultation is different. You just you just kind of judge the situation.

**SB**

What impact has COVID-19 had on the Cancer prehabilitation Programme from your point of view?

**P7**

I don't know, has it?

**SB**

Okay.

**P7**

Probably, I think there was a bit of video again, you see, it's... I don't know.

**SB**

That's okay. No, that's no, this question will mean different things to different people. So that's, that's fine. What benefits have you experienced in terms of professional development and practice as a consequence of being involved in the programme?

**P7**

From what do you mean, as in professionally for me?

**SB**

Yeah, like being part of that team emm being part of the working group? Or have you been involved in any sort of aspects of that in terms of information sharing with colleagues, or

**P7**

I think, I think where from where we, you know, a few years ago, emmm you know, it was wee bit ad hoc, and there was a bit of this, bit of that, what, what we're finding now is that there's a cohort of us, we're all pretty much saying the same thing. And I think that's very powerful for patients. Because they're, they're coming in to our service. And they're, they're not getting too much in the way, they're not getting conflicting information, it's very clear what we can and can't do. And you do notice it in the ward, you know, you're noticing the patients in the ward, because, because the operating is it's just one aspect it's the recovery and how they cope with it. And, you know, so that, you definitely notice that there, you definitely notice a change, you know, we're less not languishing, but people we're, we're just getting better at getting people processing it, being prepared to get up and get going in the, in the postoperative phase, little things like that, you know, I, I've definitely noticed that change. Emm, you know, there's not as much people emm there's just a general acknowledgement and, you know, let's get going. Let's, let's get moving. Let's, you know,

**SB**

Yeah. And do you think that comes across in certain members of your team? Do you do you think they have the experience of being part of the cancer prehabilitation they have enjoyed that? Are they.. is there some job satisfaction about seeing patients do better? Or?

**P7**

No, definitely, there is a whole lot of aspects, you know, but but as well, you see, if patients are coming in to it physically a wee bit better, and mentally a wee bit better, it makes a difference. You know, with prior to this, you know, you would have had people who you've told terrible news, you spend your time you're going off for scans, you're waiting for information, and then you've the daunting prospect of a big surgery. Sometimes you'd find people would just the day after the surgery, and then it's beginning to hit home, and there's not really and I sense the move and try and recover. And that's the phase where I would be like, you know, we need to get you up because want to get you out of hospital, you know, you say that to people you know, they're, you know, the only thing I have control over is getting you home, getting you out of hospital, no control of your results. You know, so to trying that there, but but it's you're needing to do that less so now, I think because they're, they know themselves they have to get up and get going.

**SB**

Okay,

**P7**

They're there. You certainly notice more folk like that, you know, they're more inclined to say, oh, no, I need to get up out of bed, I need to do this. And that comes from, you know, whether it's the anaesthetists right back to the prehabilitation. You know, all these little things that you're, you know, from from day one, you're building, building building building building. Yeah, no, no, that's all you can do. Yeah.

**SB**

What improvements do you think would enhance the sustainability of the Cancer prehabilitation Programme?

**P7**

You need lots more input you know, the problem with these, this cohort these these head and neck cancer patients is, it's very labour intensive. So you need a lot of you need a lot of input from allied health professionals, you need facilities, you need the CNSs you need so much and we don't really have all of that at the minute we should we've sort of people working very hard. So, you know, there's aspects that so many aspects could be developed, you know, good input for speech and language. You know, dietitian important input, you know, more CNS. All of this stuff's really important, because you really do turn people's lives upside down. And we don't we don't have that at the minute.

**SB**

Yeah, yep. And in terms of like alcoholism liaison service would you use

**P7**

Very much yeah, no, like they're actually they're so stretched. They're brilliant. Like they're really stretched, you know? They'll never turn you down you know, but that's a group that absolutely.

**SB**

So yes, more more, more investment and staff to support the whole programme.

**P7**

Very much so you know, look, you know, I look at it, you know, the end result is, you know, I don't think we're having people in wards for weeks and weeks and weeks, I think we're getting people in and out in a reasonable time frame, problem is those things are hard to quantify. You need lots of volumes of patients over a long, you know, so it's not an instantaneous result, all you can do is say, well, my perception is, you know, I think it is a benefit to patients.

**SB**

Yeah, yeah. Are there any other areas for improvement that you could think of or

**P7**

For Max-Fax consultants? No em awk like, there's you know, allied health professionals, better facilities? I don't know, you know, whether I don't know how easy it is for people to tap into the service. I suppose that would be useful, you know, you need to have it easy for patients. You know, and I don't know very much about that aspect of it.

**SB**

Yeah. No, that's fair enough. And I think a point you raised earlier, just about making sure going forward that consultants are aware, fully aware of the components of the programme and can sell that well to patients.

**P7**

it's like a Cardiologist would know what a cardiac rehab thing is, you know, probably have a sense of what it entails. You know, I sort of think I know what it is but I don't really,

**SB**

yeah. Okay. No, P7, that is brilliant. Thank you so much for your time. Is there any final comments you have with regards to the program?

**P7**

No, no, I think it's good work, I think it's a great programme, and I'm glad it's going well. Well, that does it does work. It does work. It's just proving these things which is hard, but no, it definitely does. Definitely is. Yeah,

**SB**

yeah, no, that's super. So I'll stop the recording here.

# P8

**SB**

Okay, that's us, so okay, so just before we get into the questions about how the prehabilitation programme works in the South Eastern trust, what did you know about Cancer prehabilitation before it started in south eastern trust?

**P8**

Emm bits and pieces about it. I had been in the Belfast trust and I know that had just sort of through hearing about it in terms of trying to optimise things before surgery. And I had heard sort of like the dietetic role in terms of trying to build someone up and make sure nutritionally that they were optimised before their surgery. But that would have been, that would have been it really, and I know, some of my surgical colleagues, before some of the sort of lower gastro surgeries that had been happening, the consultant would have referred them into the dietitian for some nutritional advice and sort of optimisation before their surgery as well. But that would have been the height of it.

**SB**

Okay. And that was that was in the Belfast trust,

**P8**

Yeah, so the that was the South Eastern trust for those ones, but, and I can't really remember in Belfast now what exactly the patient cohort was, but just through overhearing your colleagues and things that the patients were referred into them.

**SB**

And how long have you been in the South Eastern trust then?

**P8**

How long have I been back here? It's probably about three years back I started in South Eastern and then went to Belfast and came back to the South Eastern trust

**SB**

Very good. Well, what are your thoughts on the cancer prehabilitation programme then?

**P8**

*inaudible* dietetic

**SB**

Just in terms of what what do you think about it? You said there you knew a little bit about it at the start? What are your thoughts on it now?

**P8**

I think, do you mean sort of general terms as opposed to the head and neck patients that I work with,

**SB**

You know, suppose reflecting on the head and neck patients that you work with? What's your sort of thinking about it,

**P8**

Emm I think it's an absolutely fantastic service. I personally wonder how patients would have coped beforehand without it. I knew, obviously, with head and neck that they would have had opportunities to meet previous patients who've been through similar surgeries, but I just think the access to, you know, the head and neck specialist nurses, and to myself, I mean, sometimes it can just be a matter of the patients are actually eating and drinking okay and are managing fine, but just I know that they feel a lot more at ease before their surgery, having that reassurance and knowing that they've been told that. And likewise then the opposite end of the spectrum, you know, when patients are really struggling to eat and drink, and they're able to come and see the head and neck nurse you know, have medications adjusted, see myself given the dietary advice, and be started on supplements, and really just given that support, to try and help them be in the best position they can before their surgery.

So I think it's really it's a really valuable service for the patients, and I suppose they have commented to me at different times, you know, especially then, it's really good that where possible I'm the dietician seeing them at their pre assessment appointment, and then it's me seeing them again when they're admitted to the ward for their surgery. So even in terms of the continuity of care, it's a familiar face as well. And it takes away that nervousness before such a big surgery as well emm but yeah, in general, I think it's, it's great.

**SB**

Yeah. And it's good to hear sort of how fundamental you see that to care.

**P8**

Yeah.

**SB**

And what has, so you've sort of give me a wee bit of an idea there about your role in the cancer prehabilitation? Can you tell me a bit more about when you see the patient? Are you involved in the assessment? Or are they assessed prior to coming to you, or how does that work?

**P8**

So they're already generally assessed, I think, for the majority of them, that would come to me knowing that they're going ahead with their surgery. And I think the odd time I would maybe see a patient where they're sort of still in two minds as to what the treatment might be. And, and, and they may then but I mean, depends on what they're going for, even some of the going to have radiotherapy as their treatment. It's still, you know, still important for them to be to see a dietitian beforehand so it's still beneficial. And they generally get their treatment plan and I kind of know then roughly what their surgery is going to be or what's what's planned for them. And the nurse will have seen them and then as well and talked through and sometimes they're actually just coming up that day to see the nurse and talk through their surgery, and then they will pop in to see me either yeah generally after that, and we'll go through sort of optimising things before surgery what their current symptoms are, how that might be impacting on their eating and drinking. And then if they're what they're weight's doing as well, if they're meeting their nutritional requirements, and then also it gives an opportunity I always ask the patient if they want to discuss, you know, potential implications on eating and drinking and whether they'll have any form of enteral feeding after their surgery. And again, it gives them an opportunity if they want to, to ask questions or to talk through that element of what to expect, generally after surgery, because for most of them, they'll have already been told if they're going to have a tube passed at the time of the surgery for feeding afterwards. And generally, they do have a rough idea as to how sort of impacted their swallow may be after surgery as well. So it gives an opportunity to talk through that as well.

**SB**

Okay, and you said there that on most occasions, they do know what their treatments going to be. On some occasions, for those that don't know, does kind of the outcomes of the prehabilitation, impact that decision at all or are there other issues?

**P8**

So sometimes it can be as simple as you know the surgeons have a plan, but sometimes when they go in, it can be a lot, you know, the cancer can be a lot more widespread or things can become a lot more major sort of might be a minor surgery initially, it might turn to be something a bit more a bit more of an implication, which have longer recovery. So you generally will have a rough idea of what's going to happen, but then it may change by the time they're they're opened up. And generally I don't see the prehab impact on it as much like I wouldn't have I work with another patient cohort where actually their fitness for surgery is a huge thing is sort of, to me more of a bigger impact on whether they'll be offered surgery. Because I suppose these patients already decided for majority that they're going for surgery, you know that that... You know, the consultants have already made that, you know, pre assessment call beforehand. And then so that that's more of why it's prehab because they're working towards their their surgery. So yeah, generally, it wouldn't be I wouldn't I haven't many patients that are heading towards their surgery and then because maybe they're losing weight, or you know, their fitness changes. I haven't had any I haven't done any but that's not to say that there may be maybe some but certainly since I have been doing it there hasn't been anyone that I can remember.

**SB**

Okay, so the patients are assessed before they come to you. So just so I know, I'm not gonna ask you questions, then about your perceptions of the referral or the assessment form, you know, if that's what about in terms of the project setup? Were you involved in that?

**P8**

No, I think it was more. See, I only took this role on I had a colleague who retired beforehand, and she sort of retired I think, was it just a couple of years ago now, but it was that the prehab clinic hadn't started by then. So at that point, I hadn't known how much she had been involved in in terms of the set up. So she maybe was…

**SB**

Okay,

**P8**

…a couple of different things she was involved in. I know, certainly, there were, um, she was involved in like referral forms and things, but it could have been for something slightly different I'm not 100% sure. But since I've kind of been here, it's been more so the way I would get referrals is through an email from [HNC CNS] or [HNC CNS] the head and neck nurses who are booking appointments in for the prehab. So they've identified already that the patient is appropriate to attend a prehab clinic. And that will be the way but to be honest, she could have been involved in the setup of it. I'm just not sure.

**SB**

Okay. Okay. And then just checking as part of being involved in the pilot did you receive any training specific for the pilot?

**P8**

No, I had shadowed my colleague previously with moving into the area of head and neck cancer, but no, no specific training in terms of the how the prehab clinic had worked? No.

**SB**

Okay. Yeah, yeah. Yeah. And is there anything any way you anything that can be done to make you feel better prepared for your role in the project?

**P8**

Um, I think probably just to have access to people who are part of, you know, a prehab clinic for I wouldn't even say for other specialties because actually, the role area of head and neck is very specific. Now I would liaise sometimes with colleagues in the Belfast trust in the Northern Ireland Cancer Centre who are seeing head and neck patients, maybe for radiotherapy. But that, again, is very specific, and there's very different issues at that time. I was given the opportunity in work to do a master's module on modern cancer management. And I chose head and neck as my area to just do a bit of research and do my assignment on but that was sort of personal like personally trying to gain a bit more knowledge and information and experience in that area. And certainly, yeah, I think probably it would be beneficial to have you know, even, yeah, so I've just recently moved into the role of oncology in general. And it's something I've been discussing with my team leader, even having sort of competencies and sort of knowing you know where I'm at. And actually, if it is similar to what other you know head and neck rehab clinics are offering that kind of way, but, generally I think this has been quite successful, we haven't had any sort of issues at this point, I suppose it still is in early days.

**SB**

Yes. Yeah. So now, those are good points for going forward with the project. You know, and more recently, maybe that would be something to look at. So I'm just getting this clear in my head, Aoife, so the people that come to you have already been assessed, there to you, do you do any other sort of assessment as part of the outcome measures for the prehabilitation programme.

**P8**

So I had *inaudible* fill in sort of a outcomes, I would take their weight, their height, percentage weight loss, and sort of fill in a screening tool for those and send it off to somebody who is collating all the information, but I think that the aim of it would have been take them at that time point. And then also, when they're admitted, they're automatically refer completed their MUST tool for at board level when they are admitted to check their weight, height, BMI. And I think the long term goal would be then, you know, say, in three months after their surgery, if they were coming back up for follow up, then check again and see at that time point, you know, as to how they got on and how they've managed after their surgery. But at the moment, there isn't funding there for that follow up. So at the moment after their surgery, what happens is I refer them out to the community dietician, in whatever trust that they're in, and they would be followed up that way.

**SB**

Okay, okay. Yeah. So potentially, there's sort of missing data there then.

**P8**

Excuse me, sorry?

**SB**

Potentially missing data there then

**P8**

Yeah, yeah. And to be honest with you, I have kept meaning to do my own analysis on the information that I've been sending to the sort of performance analysis person. And even compare it, because I would have all the ward data as well, but just with COVID and everything that hasn't been something that I've been given sort of, you know allocated time to do.

**SB**

Yeah. Yeah. And are there have you encountered any challenges with this screening referral consent process, and you mentioned there that, obviously, people are being going to other trust areas. So there's, there's a problem there in terms of follow up. But are there any other problems that you've encountered

**P8**

Generally because I've been checking my own assessment, I'm happy enough with that, sometimes at ward level, there's just you know, sometimes that can be a recalled weight am or can be a weight maybe that's been taken, you know, from a letter on ECR you know months prior to that. But generally, I'm quite good at knowing then you know, if they attended pre assessment, I know where to find that on the computer system and can correlate it with mine. And then they'd be weighed, hopefully, on the ward at some stage that will be accurate. And so no sort of real challenges with the with the MUST process, with the MUST screening tool from myself or from the nursing staff. Generally there have been challenges recently in terms of the follow up of these patients. so sometimes if they are discharged on an enteral tube feed, you know, I'm referring out to the community, they'd be best placed to follow them up at that point, but it can become a bit sort of unclear at times, if they're still attending the consultant in the Ulster and they're coming up to appointments in the Ulster and and yet their dietitian's in the Belfast trust, Northern Trust, Western trust. So sometimes that's a bit difficult. but it's something that we've been, you know, that I know, the consultants and the nurses and our dietetic team leads are all aware of and it's something there's ongoing discussions about, and generally, I know that the Ulster have like a gastro hub, where patients will come up to, but as of yet, just, it's just to do with funding really, there's not that, you know, I suppose in an ideal world, I may attend a follow up appointment with their consultant, with the nurses after their head and neck surgery, and then they'd have access to a specific dietitian, but just say, even with south eastern trust patients at the moment, they're being referred to out to the community dietician, you know, they're not, they're not being followed up by me either. You know, it's all trust at the moment.

**SB**

Okay, so it yeah, so it's kind of hard to see where the path goes then.

**P8**

Yes, yeah. I don't get that follow up with them, because some of them I know will go to the City for further further treatment in mainly radiotherapy and others will, you know, that that that will be them it just depends on their, their biopsy and things when after surgery so that yes, it's that's the lack of funds at the moment for follow up.

**SB**

Yeah. And have there been any helpful aspects to that screening referral and consent process, like, for example, the way the forms presented.

**P8**

Emm it's very easy to fill in, I suppose that I suppose for me, maybe as well, it would be easier if I had someone you know, if we were doing a piece of work on this, it would take me to go back and to look up all the patients that previously you know, at the moment, I am not, sort of am filling their prehab appointment information, but I'm not pulling their when they're admitted that initial point when they're admitted to see, okay, have they lost much weight, you know, was that advice obviously successful in terms of helping their symptoms or whatever it may be. So, I suppose Must would really capture that, but even at the minute in terms of their weight to see if, you know, if the plan has been successful in terms of maintaining their nutrition, I'm not putting that set of data with the prehab data, so I'm not actually looking at it at the moment, I'm not analysing that, if I was doing a piece of work on it will be in retrospect, going back to look at that. but I often do think that with these patients, a lot of it is their symptoms as well, and how well, you know, even things like their quality of life at home and social eating, as well sometimes gets impacted. And that's not being captured, you know, through the MUST screening tool.

So, sometimes I do think like, even if it was a question when they come to me initially, you know, in terms, whether it be we ask them things like their quality of life or their symptoms, and, you know, maybe changing to that softer diet and, and avoiding those foods or, you know, go into onto supplements and liquid diet, may actually then you know, at the time that they have their surgery have helped them and actually improved their symptoms.

But I should say, as well, with these patients, a lot of them coming to me already knowing you know, it's common sense. If they're sore at one side of their mouth, they'll have started chewing at the other side and will already be going for softer foods. So sometimes they're already kind of on the path to helping their own, you know, symptoms, and even improving their uptake

**SB**

But that's interesting, just you're kind of thinking about the limitations of the MUST tool and maybe going forward, there will be additional questions that would be included then?

**P8**

Yeah, I suppose just in terms of their overall eating and, you know, I suppose some of them do just think they're eating and drinking, lots of them describe it as just failing their bodies, you know, they're not enjoying eating and drinking at this point its sore, they're only doing it to get nutrition and eating to try and optimize things. So yeah, a lot of it would be but then I suppose they're still at you know, they're still experiencing that type of thing before they get their surgery. And likewise, then after their surgery, they're still gonna be you know, majorly impacted and have those restrictions. So, in some ways, I'm thinking yes, it would be good to know, generally, is our plan working, to try and maintain things, at least before they come in for their surgery. But by the same token, I suppose their symptoms may still be the same, if that makes sense.

**SB**

Yeah, yeah. Yeah. Okay. How have you found patients interest to engage in the prehabilitation?

**P8**

Generally, they're always very engaging with seeing myself. I should say, previously, there was the speech and language therapist alongside I don't know if [HNC CNS] had explained that

**SB**

That didn't work out sort of funding wise did it?

**P8**

Yes, yep. So at the time, it would have been very good that we had more of a joined up approach in terms of, you know, they were able to tell me, you know, exactly, what type of texture they could manage, and they were able to suppose I mean, I can only go so far with the information that I can provide, after their surgery, you know, trying to give them a heads up of what may be ahead of them. And obviously, the speech and language therapist is the one that assessing their swallow. But but that was quite good to have, you know, both of us at that stage. But generally, a lot of them are very thankful to to see somebody and they're always happy enough to you know sometimes it can be long enough, if there were like, say, a couple of patients booked in, you know, we're trying to one sees one while one sees the other and move between, but I suppose they can be here, sometimes a couple of hours, and they're always happy enough to wait and they'd rather speak to me as opposed to heading on home. So yeah, yeah.

**SB**

And that's a very interesting point you're making about the importance of the collaborative working between you and speech and language, because that maximises what you can do then?

**P8**

Yeah, definitely. I think even they can do do swallow tests and get them going on, like exercises for improved muscle function before their surgery as well. So yes, that joined up approach that they're doing everything they possibly can to try and optimize things.

**SB**

Yep. Have you met any resistance from patients to be part of the prehabilitation programme?

**P8**

I think there was maybe one gentleman who had declined input but again, because I think I maybe tried to ring them then afterwards emm but again, that will be identified before they even come to me. There's been nobody that they have booked in, that hasn't, you know, that has been resistant I would say. everybody's been yeah, they would already have said you know the person would have already declined it if they're not coming.

**SB**

Okay, good. How has COVID-19 impacted your service provision as part of the, you know, the in terms of part of the prehabilitation?

**P8**

So I think, when the prehab was designed, I think they had nearly wanted, you know, three or four patients per clinic. So I would say mainly, it is really one or two patients that we're seeing in each clinic, and I think there was a period then where sometimes it was every other week that we were seeing people, I suppose around, you know, before Christmas time, just to do then, again, sort of directly links with the amount of patients and with those consultants who are actually doing the surgery, and when their surgeries gonna be booked for. So you know, the time definitely, I think they have noticed a decline in referrals, you know, through through GPs, and through dentists because people weren't physically attending. And so then referrals weren't as high, but then even at that they were delaying maybe slightly later in the presentation, which may have then limited their treatment options, and again, then, the surgeries were fewer. So there was less people being booked in that way as well. but certainly, yeah, I think they sort of envisaged that it would be three or four patients.

And I suppose more recently, is the there's quite a quick turnaround time. So I don't know if that's part and parcel of people presenting that bit later that actually, they may be coming to see me on a Monday, and they could be coming in the following Monday for their surgery. I think whenever I thought prehab Initially, I thought I'll be seeing them and it could maybe be a month. And to be fair, that was the way it was initially with quite a few patients that there was maybe three or four weeks to their surgery, whereas in more recent weeks, that has definitely been a quicker turnaround time. So I'm not sure if that is just part and parcel with later presentation and trying to sort of do the surgery as quick as possible. We definitely have a reduced number of referrals at the moment. I'm not 100% sure on that.

**SB**

Yeah, no, I was just wondering there with you'd said you anticipated three to four per clinic, but now that we're not in the same restrictions? Are we are you are you seeing that now?

**P8**

No, it's still one or two, I have to say and still they are, like I suppose from the one from last week, they were admitted within one to two weeks, you know, for their surgery. So it still is at the minute quite quite quick. And again, only one or two for the clinic

**SB**

That's interesting just from what the thought was at the start. In terms of you being part of the cancer prehabilitation programme, have you experienced any benefits in terms of your professional development practice, being part of the team,

**P8**

Yeah, I think definitely, I personally prefer knowing who my patients are to come in, you know, at ward level as well, and having that information having built up that relationship too and I think you know working as part of the MDT as well, I think doing that cancer module that I had talked about, really emphasised, you know, all the literature that I was reading was really emphasising the importance of the MDT approach and actually, you know, being able to, I suppose, in follow up, which we're, you know, lacking at the moment, but certainly all through the patient journey, you know, from diagnosis right through and the importance of prehab beforehand, but, you know, it's helped me to like I am very well supported by the head and neck consultants and the head and neck nurses in terms of these patients as well. And then when they go to the ward as well, you know, there's a lot more input from the Ward nurses too. So definitely

**SB**

Have you enjoyed being part of the project?

**P8**

Awk yeah, I definitely I think when you have your own sort of patient caseload and and again, you know, when I do have students in and I'm able to show them what I do, you really do have a sense of worth and value and that actually, you know, it's nice following a patient journey through you know, sometimes it is frustrating that the other side we you know, we don't get to see it, but I know that the patients are really thankful and they like seeing someone they know, you know, there's so many AHPs and nurses and everyone coming to see them on the ward, they like knowing you know, they're able to know your name and able to have your and especially just having your contact details beforehand. because you can see nearly the nervousness at the start of the consultation, when they talk about eating and drinking. I remember a man actually coming to the consultations and told me that he you know, he was maybe like a week out from his surgery and I think it was maybe just after Christmas that he was having his surgery. And he told me that the day before that he had had his Christmas dinner because he was so afraid, you know, after the surgery, what way his swallow was going to be so they are quite apprehensive, but I think you definitely feel like it's another sort of element to the dietetic role that it's because you are part of the MDT and part of the project, it's a lot more, you feel very valued I suppose and your input very beneficial.

**SB**

That's great. just thinking, now in terms of improvements to enhance the sustainability of the cancer prehabilitation programme and I know you've you have mentioned there earlier about the speech and language input and how that complements your role. Are there any other areas for improvement, do you think?

**P8**

I mean, I know in other prehabs that they do have the likes of a physio, but I don't really see that being, you know, I know from the other area of cancer that I work in upper GI cancers, I think it'd be very, very beneficial. So I don't see it being just as important for prehab before, but I know that lots of patients are referred to the move more coordinators anyway, so that they're having that sort of element. I'm trying to think I suppose maybe just actually been given maybe a bit of time and I suppose it goes to like funding and then even capacity within my own role to do a bit of analysis round you know, in in terms of how many patients are on certain supplements, in my head I thought all my patients will be started on nutritional supplement drink before the surgery. But actually, I'd have to say quite a lot of them already are managing, okay, with diet and with food first, which is a good thing. And a lot of them, then it's just smoothie, sometimes actually, it's just healthy eating advice that they need before the surgery, you know, it's not even trying to help them put on weight, it's just trying to help them maintain everything. So I suppose you know, being able to do a piece of work around that. But at the moment, it's just not possible within our department to do. I'm trying to think what else, generally the computer systems and all are fine. And there's weights and all here to weigh patients I suppose just the timing of things as well, sometimes I do feel that if it was, you know, a wee bit longer before their surgeries. I did have a man recently there that I seen on the Monday, started him on supplements, and he was coming in the following week. And actually, he still hadn't, he had just received his supplements the day before he came in, so that was a bit limiting. That's just to do with the timing I don't think that's in anyone's control.

**SB**

Yes, where do they get their supplements from or they have to be sourced.

**P8**

So I generally do a referral I'll ring their GP after and get them added on to their script. But then sometimes it can take you know your own GP surgery, it can sometimes take like a day for the GP to pick that up, I think some of them actually up to 48 hours, and then they go to the pharmacy, then they're maybe waiting because the pharmacy don't actually have that brand of supplements and that can delay things

**SB**

Yeah. Okay, so that's interesting, just then the other partners that need to be considered when the timeframe is so short.

**P8**

Yeah, yeah, there are I was able to get a few from we have a sort of a stock smaller stock here in our dietetics department, so I was able to go and get a few there and give him some just to get him started on. But sometimes, yeah, I mean, sometimes with the best will in the world like I'll ring the GP as soon as possible and say like, this is really urgent, but this just the natural delay of things.

**SB**

Yes. Yes, absolutely. Can you think of any barriers for the long term delivery of Cancer prehabilitation Programme in South Eastern trust, or even barriers, then, on a wider scale, if this was implemented across Northern Ireland,

**P8**

I think actually just the way we are at the moment, funding is a really big thing. And you know, yourself as the research is nearly done to creating the business case, to get the, you know, the whatever whole time equivalent, we appoint whatever whole time equivalency to post. So I know that there's a lot more in terms of even the follow up and that and as I say we are having more discussions at the moment in terms of the tube feed patients that are discharged. But certainly, they have a bit more of a joined up approach. So I think funding is a big thing.

And then I suppose at the moment, I'm the only person that's my department that is given the you know its part of my job, and I'm given the time to do that. I think at the moment, it is a rolling thing. So you know, I know whenever I'm off on leave or anything like that, it's not covered, we don't have the capacity at the moment to cover that, the prehab clinic. So I don't think there's been any days have been cancelled because I haven't been about I just try to give sort of as much notice as possible. But certainly, I suppose more funding, probably the biggest thing that that someone could do be cover it or and again, I'll be following up with I'll maybe see the patients at prehab. And say if it is four weeks until their surgery I generally like to ring them again in between, you know, maybe two weeks down the line to check in on them. And again, sometimes that phone call may be pushed or may not I may not have capacity to do that. Because, again, there's only so much that I can't even remember off the top of my head what exactly the point I think it could be point two or something I'm not 100% sure for inpatients and outpatients. So definitely I think funding will be a big thing. But otherwise, no, I can't think of any other barriers.

**SB**

And that’s quite significant. There’s a lot of expertise and knowledge you have gained as part of this project but if there’s no one else working it alongside with you that’s a problem it could potentially get lost if you were to move on or

**P8**

And I suppose that was kind of the case with my colleague, she retired so I was given a couple of weeks to shadow her because it is such a specialist area but you know I would say there would be a lot more information that she could have given or passed on to me had it been me shadowing her for six months say as opposed to the little time that we were given that could be afforded.

**SB**

Well that is us towards the end of the interview, are there any final comments you wish to make about the project or your participation in it?

**P8**

Generally I think it’s been very worthwhile. I suppose in some ways I do think that these patients would have been coming in a lot worse condition and needing a lot more sort of a lot more intense dietetic follow up but actually it’s a good thing, it’s a beneficial thing that we’re there if needed and some of them are, most of them are managing ok but generally it’s a very worthwhile service and I think being able to provide dietetic input to patients before you know their surgery I think if you were to, I’m sure you are looking at patients’ opinions on the whole thing but I think it’s’ been very positive in my experience so far, and being part of the team has been very positive as well.

**SB**

That’s good, thank you so much. Just going to stop the recording here

# P9

**SB**

Okay, so emm what did you know about Cancer prehabilitation before the South Eastern Trust programme?

**P9**

Nothing at all. I'd never heard of it before had to Google what prehabilitation meant

**SB**

Yeah. Okay, what are your thoughts about the project now

**P9**

I think it is a really good project emm theoretically, since it started, you know, I'd had a look at the Manchester that's got a big one that that's ongoing and seems to be really good. I think we might have been scuppered a little by COVID, on our limitations and how much we can actually… certainly most of our patients seem to go to move more coordinators, and the vast majority of our referrals get referred to move more coordinators. But because some of them are being seen virtually, some of them are getting into the leisure centres. Some of them might want to patients might want to go to the leisure centres and not be able to or the other way, they might know that it's best for them to go to leisure centres, but not feel comfortable because of COVID I think we're probably at the mercy of that a little bit that we may not have the same results that we're hoping for.

**SB**

Okay, interesting. Yeah. Do you view this intervention as important? In terms of patient care?

**P9**

Yeah, yeah, I think, from looking at the research from other places, it does look like it has a positive outcome. And I have some oversight on the patients’ surveys back to us on how they thought the programme went. And I think we might not see the physiological changes that we were hoping for. But in the patient questionnaires, you can see how just having somebody to talk to really, really helped them. So even if we're not going to see the the hard data that we would like, I think it is still important to do because you're still affecting those patients lives.

**SB**

Yeah. Okay. And can you tell me a little bit about your role in the cancer prehabilitation programme?

**P9**

Yep, absolutely. So I ... starting in the very beginning, there was meant to be one of the cancer emm oh I don't know what he's called, but he does a lot of cancer reporting on the cancer system, he looks up looks after the cancer system in our trust, and he was hoping to be able to, you know, create a database to record all these patients in and stuff like that, unfortunately COVID the way it does, he got dragged off to do something else. So I was brought in to come up with a referral form for the not a form a referral process for the CNSs to be able to refer patients to the move more coordinators, and a way for the move more coordinators to report their results back to us. So that was really what I was involved in at the start of this. And it was just doing making excel forms, which again, wasn't not ideal, and we're still using them. It's, it's not a robust way of looking after this at all. But just with the timescales that was what we what we went with.

And then moving on from that original that that I was involved with, I then started doing like weekly check ins with the move more coordinators, and because I hold all the data, I get all the referral data and I get all the move more assessment data. I combine that all into just one giant Excel so everything's in one place. And because I have oversight of that I link in with the more coordinators once a week, usually usually once a week, just to give them little reminders on if a patient questionnaire is due so if I can see that, oh well this patient was meant to have surgery on the first of April or sorry the first of March but I don't have an assessment for their pre surgery yet. I'll just send a wee prompt to the move more coordinators and then the other area is the Bouvier report cards the outcomes based accountability Jill in my team looks after those now but I started the initial scorecards for cancer prehab and yeah Jill in my team looks after that now and I have very little to do with the day to day looking after of that it would just be QA.

**SB** 5:08

And were you involved in the early planning and the creation of the pilot,

**P9**

Not so much. So I think I did come in a little later, they had definitely had meetings with the move more coordinators before I joined because I know the move more coordinators initially had some concerns about the amount of data that they were asking to be recorded. And they wanted it to be like as easy as possible, so there, there had definitely been some sort of meetings before I joined because they kind of told me that the move more coordinators had these concerns with whatever I was going to build for them to record the data on. And there was definitely already data access agreements and stuff like that in place, which probably would have taken a good bit of time before I joined. I claim to know nothing about those. They're far too in depth for me. So I think, certainly I was involved before there were any patients on it, and but not from the very, very initial stages no

**SB**

Were you able to shape what is now? Do you feel that you're able to influence and have a say?

**P9**

Yeah, absolutely, definitely in some regards, I was able to say what would and wouldn't be possible, I think, ideally, we would have had a bit more time we were just we were just so so time bound at the start to get to get referral, a referral process set up that, you know, ideally, what you would want to do would be to go to IT and get them to put together a little automated system. But I did have a chat with the Belfast Trust because they're starting off doing something new, and they have an IT rep on the call. And he was like yes, fantastic. We can we'll build something that's no problem. You know, we were they made a comment. They wouldn't do it the way that we were doing it, because that's how they would do it about 10 or 15 years ago. And I was like, I'm not offended at all. That's totally fine. We were completely time bound we didn't have time to involve IT. But since then the Belfast Trust Lead has actually come back to me and say IT cannot help them without a chunk of money and a chunk of time. So they're they're going to end up doing it the same way that we're doing it. It's a bit of a workaround. It's not ideal. But yeah, it's just just the way it is. But no other I think, we're a victim of circumstance with time definitely. But other than that, I felt like yeah, I was able to influence certainly,

**SB**

Yes, good, good. Now just sticking with it just because you brought it up now I think it's important, just like in case we forget to talk about it again later. But just in terms of improvements for the future, do you see that IT development is key to improve the project?

**P9**

Yeah, absolutely. So my concern has been this whole time and I brought it up a couple of times if I get hit by a bus tomorrow, all the ...

**SB**

Let's hope you're not?

**P9**

I know. Well, yeah, absolutely. it's eh we have everything automated, so they were really keen to have things automated. So the referral has a button on it that says press this button, and it'll send to whichever contacts the CNS wants to. So the CNS never has to attach the email and go through the list of contacts and who to send it to. it just gets sent automatically, and it will always cc me in. So if I get hit by a bus no one's going to be able to collate it. You know, there's, it really is a single point of failure, which is, is the main issue, I think if we had if we were able to get IT to build us something, or some sort of referral system, that would take the huge weight off my shoulders. And I think that's certainly what Belfast Trust seem to know that that was what they needed to do as well get yes, they're not going to be able to do it. But, but for improvements, that's my main area of improvement, we need something a bit more robust than things going through just me.

**SB**

Yeah. And you said there. So the CNSs they complete the referral forms, and they can just just click one and it goes to the that's all set up.

**P9**

Yes, absolutely. So it's just a little bit of code that’s in the in the excel, so they have a list of god maybe about 20 options that they can select from. So there's the the move more coordinators, but it might depend on what location they are, or if it's just exercise or nutrition, or if they need like specialist physio, or if they want to be referred to like the stop smoking service, there's loads of different places that they can be referred to. So it's just a drop down that the CNS select on the one that are the five or 10 that they want them to be referred to. And once they've selected those, there's a button at the bottom that just pings the referral out to them.

**SB**

Very good. Very good. Um, did you receive any training in relation to the project?

**P9**

No.

**SB**

Okay. Is there any way that you could have been better prepared for your role in the project?

**P9**

Um, I don't think so, you know, they've been very good with things that I would have had no experience with. There are many things that I probably didn't need to know like, the different so when the move more coordinators are filling out some assessments, there's like, I'm gonna get it wrong now, but it's like an EQ5 or something assessment and things like that, that I didn't really need to know what they were. But they were quite good at, you know, if I asked just explaining what it was, but I don't think there would be any way that I could have been better prepared really? I felt yeah, I felt comfortable enough with with how I was going into it.

**SB**

That's good. Good. Are there any challenges that you've experienced? I'm kind of thinking about the screening referral consent process? you're at the other end of that, are there challenges you can see, from your point of view in that process?

**P9**

Yes, so I think we're probably on the other end of it now, we did have a lot of challenges with the form not being filled in completely. So a lot of the work that I do, because I'm also looking at the outcomes, at the end of it, I do a lot of sort of validation on the data as it comes in, just because it gets sent to me. So I generally will open it and I'll have a wee look through to see what's been filled in and what hasn't, and there was there were real issues for a long time with things not being filled in properly. And it did take it took it took a wee while to get it sorted, and then thought that I think there were two meetings, maybe where there was a specific team. And really, it was a specific couple of people in the team, who just were leaving a lot of stuff out. But it does seem to have been resolved now. there's nothing really you could have done to prevent that. You know, it is people are so so busy. You know, it's understandable that if they don't see things as important they're not gonna fill it in.

**SB**

Okay. So was that the basis of the lack of filling in was that was it that they didn't see it as important do you think?

**P9**

Yeah, well, it took a while for me to get any sort of response to why were there bits that were continually not being filled in. And the reason was, the reason that they gave was that it wasn't, they didn't feel it was appropriate at the time to be asking them so many questions given that the patient has just got a cancer diagnosis. And I didn't really feel like I was in a position to argue with that not being clinical, obviously. And so we did, you know, have a lot of chats with [CNS HNC] and [ADEPT FELLOW] and, you know, it kind of went I suppose, above me to somebody a little more clinical, who could maybe encourage encourage them a little bit more that yes, well, we do need this whole data for everybody. We can't have it just for a few. And then the only other thing that I think a real challenge has been emm with the timescales of the patients who are taking part in the pilot or in the programme. I did see I think in the Manchester model, it looks like they're meant to have is it two weeks that they're meant to be on this programme? And, you know, that's just the minimum.

We have a lot of patients who have days between referral and having their surgery, and it's a waste feels like a waste of their time, if they're not going to be getting any benefit out of it, you know, the move more coordinators are having to faff around filling out forms. And in that case, I think maybe just I do think they get the benefit from speaking to somebody, but maybe it's not appropriate that they're actually having all this data recorded for the programme, maybe it's more important that there's a touch point with them, and that they're just told, you know, maybe you're not appropriate for this prehabilitation, but after your surgery, come the this leisure centre, you know, they have so much experience with that. So we do have an awful lot of data recorded that I think can't really be used, it doesn't really show us a lot. But again, just with timescales, it's possibly something to think about in the future, probably in some sort of restriction like that

**SB**

Just going back to whenever you had to prompt move more coordinators to complete more fields. How did they how did they respond to that? Were they okay with having to go back or…

**P9**

Yet? So that was actually not the move more coordinators, that was the CNS. No, no, not at all, that was the CNSs, where there was an issue with the referral data not been filled in. I have to say the move more coordinators have been fantastic. They fill everything in, they are so careful. They're amazing. Yeah, I did, there was I did, you know, send a couple of emails back for response whenever I saw one come through that was quite blank. And I didn't get an awful lot of responses, I did feel like I can't check every single one that comes through, it's not my job. It's definitely not my job to be checking and QA'ing every single one that comes through. So I did go back a couple of times and ask, could it be completed? I spoke to [CNS HNC] and [ADEPT FELLOW], during we have occasional meetings. And they just kind of reiterated, can you go back again and ask and did and yeah, didn't get any response. So as it stands, the ones who are being referred currently are all filled in. But we've got an awful lot of missing data for people who were referred before that was that was in place. [CNS HNC] does seem to think that they will have that data somewhere that it might be recorded on a different system or something. But tell me who I have to shake down to get it? I don't know. *laughs*

**SB**

Yeah, that can be interesting. So you mentioned there about I like I think that the excel form sounds amazing. So and that was I think that will definitely be a helpful aspect to the form to the likes of the CNSs, are there any other helpful aspects you want to point out about the screening referral and consent process?

**P9**

No, I don't think so. Although it's very helpful for them. It does mean it's kind of two sides, the same coin, it's great for them, because everything gets pinged off automatically, but the bad thing is it just gets sent to me. It's not something that gets sent to a load of people, but other people can access it. And just the nature of of what it is, anything else that was I don't think so everything was fairly standard. And it hasn't been other than the missing data, there really hasn't been too many issues with it, thankfully. we do have issues with getting assessments back from the move more coordinators sometimes and again, it's just it's one. And I know she's very busy.

And the last thing she wants to do is to be sending things back to me all the time. but I think for the other move more coordinators, they are quite happy. They seem to be quite happy with just being able to ping the excel back to me. It's not something that they have to sit writing, you know, a load of things and scanning it in or anything like that, that they might have had to do before so you know, it's nothing innovative, particularly but I think they were worried at the start that it was going to be a lot of work and I I don't think it's as bad as they thought it was going to be they certainly commented that whenever we showed them that they thought it was okay and it was manageable. It wasn't as bad as they thought it was going to be. So

**SB**

Very good, good. So whenever the excel form is filled out and they select say they select that the person is going to be referred to three different people that just comes to you. And then do you make the link?

**P9**

No, it goes to each contact for the three things that they've selected, and me. *inaudible* Yeah, I'm the only constant. And I'll always be CC'd into everything. even if they hit that button and don't select it to go to anybody, it'll still go to me. They just don't know it

**SB**

Yeah. so just you had touched on this at the start, but what impact has COVID-19 had from your point of view?

**P9**

Yeah, so I think COVID is going to be the thing that might scupper this for us to really show improvements. so each move more coordinator has been doing different levels of face to face interaction with the patients, we have only from today, we're going to be recording how the the exercise prescription if we take exercise, which is the main one that patients get referred to a move more coordinator for they in some cases were just doing it entirely on Zoom. And then at a certain point they were going back to the leisure centre, there were some patients who were getting just reading booklets, there were some patients who were getting texts, you know, each move more coordinator was given a different level of interaction to their patient. And this, of course, changed depending on there's still some more coordinators who aren't seeing people face to face, some of them are there's no equity in what... it is a post code lottery? I know that's awful, but it really is depending on what post code you go to, that's how not good I'm sure that it's all good, but to what level of interaction you get with your movement coordinator.

And, we have only just started recording that detail of will this patient be seen face to face will will they not so that's going to be huge, I just if we can have everybody going into a leisure centre, and we knew what they're doing, we we can see this patient is you know, working their butt off for an hour but whenever people are doing exercises at home it's difficult to quantify. And I I just don't think it's going to be enough value. we're also we didn't record physiological data for a long time. Because, you know, they couldn't do people's blood pressure. They weren't seeing them face to face, so we've lost so much data there as well. There's quite a lot that's just not going to be usable, I think and the stuff that is usable might not show us what we want and given that you know patients are going to be they're going to feel awful after cancer treatment they're probably not going to be jumping around the place with suddenly you're fighting fit, I think we might get a shock when we look at the physiological results not a shock we know I just don't think it's going to show the the great success that the other programmes have

**SB**

Yeah, yeah. And has there been any any benefits to COVID if it affected the programme any way positively.

**P9**

That covid has sorry?

**SB**

Yeah, yep. Yep.

**P9**

I think you get people who are certainly a lot more comfortable doing stuff virtually people who are I have absolutely no evidence for saying it but it's just I just think yes you'll get people who won't want to go into a leisure centre to do exercise they're embarrassed but here there's this girl who is sending me YouTube videos of what to do and they'll they'll do it in the house on their own

**SB**

From your point of view P9 in terms of managing this has been any benefit to COVID

**P9**

I think I would have been the same either way because I'm so removed from it. I don't think no I don't see that there's been any benefit or or not really

**SB**

Do you think you've had more time or less time

**P9**

Because covid? I have no time *laughs*. Yeah, it's rather than it being just I think my expectation at the start was that I would create this referral form, I would create this assessment form and that I would step away from it but you know I seem to be doing presentations and stuff like that, you know, more and more. But that's the nature of what you do when you hold the data people are more likely to come to you and be like, oh, patients who were in Belfast, what was their average starting weight? You know, you get ad hoc requests all the time, all the time. But that's just the nature of this business. Yeah.

**SB**

And that's interesting going forward Yeah. For other people in your position that are gathering that data. It's interesting, just that they know what that role will entail. Yeah.

**P9**

Yeah, absolutely. I mean, you do, once you show people a slice of data, that's they just, it's the same in any, it's nothing to do with this programme. It's absolutely the same across any any industry as well, you show them their data, you show somebody their data in the chart, and suddenly, they have 100 questions that they didn't have before. Yeah,

**SB**

Yeah. Um, have you experienced any benefits to being part of this programme? Being involved in, you know, the development and setup, have you.

**P9**

Yes, it has been really good. So whenever I make the these forms, it's in a type of code called VBA, which is in excel, that's just what automates excel, and I haven't done it, I haven't done it in years. So it meant that I got to relearn that, and that was really good, I really enjoy that. So it was definitely worthwhile in that respect. And then I just think it's been really good learning about, you know, my job, I don't get certainly nothing patient facing, of course, and, you know, you feel a bit removed from the the hospital world. So actually being able to feel like you know, the patients, I might not…. the most, I will see, I'll see a patient's referral come in, and I'll see their three assessments from the move more coordinator. But you know, seeing the little comments about, you know, oh, so and so was feeling much better today. She's had her operation, she's very optimistic. It's nice, you know, it's not something that I ever get as part of my job. So there's a bit of a job satisfaction in it as well, that you don't get with with other programmes.

**SB**

Yeah. And what about the sort of the working group how have you found that in terms of professional development?

**P9**

Yeah, really good. Really, really interesting. It's been great for? I haven't had any, anything come up yet. But it's just areas of the hospital that I would have had no clue who to go to before, if somebody had come to me asking, you know, who can I go to to find out about colorectal data? I wouldn't have had a clue. But now you know, I do have those connections that I wouldn't have had before. So yeah, it has it's been really useful. And even with the move more coordinators as well, you know I suppose it's just good for professional development, just to have those contacts outside of your own your own agency or your organisation. And for them as well, you know we're all quite friendly, if they needed anything they'd be happy enough to. Yeah,

**SB**

Absolutely. So what improvements do you think could enhance the sustainability of this programme going forward?

**P9**

So moving away from me being the single point of failure *laughs*? If we had IT, I think that's, that is really key, but that comes with a whole host of, you know, needing money, more than anything you need, its funding, I believe it does cost to get IT to come in and do anything in these projects, which is a pity. And certainly, it would take them a lot more time that it takes me just to cobble something together. But I think for sustainability, that's what it needs. And then I think having a better look at the processes. I think we, as I said, I think we are collecting data that we're never going to use, and it could be there could be a better process against who is eligible for the programme, and who maybe gets a more informal you know, contact with the move more coordinator, but you're not part of the programme; they shouldn't miss out.

Yeah. other than that no, I dunno it, I suppose it was challenging. We had a move more coordinator who left and we've had a new one who I was speaking to today who's just started. And it wasn't, it was slightly challenging because it meant, obviously, we've got this excel that pings off to people's emails. So it just meant I had to update that email, but then I have to send it out to all the CNSs and make sure that they're definitely using that from now on, because if they use the old one, it's going to send it to the old coordinator. So it's not a problem, and but it's just one of the things that's again, not really sustainable because at some point, you know, another move more coordinator will change, there'll be another change in email address, and it's tough for CNS to keep on top of which one's the most recent one. Again that’s probably IT though. If we had IT involved they could come up with a way around that I’m sure, so that is my biggest one, yeah getting involvement from IT.

**SB**

Yes you’ve made a series of really good points as to why that needs to happen yeah.

Are there any barriers you see for the long term delivery of cancer prehabilitation? The funding obviously is going to be one…

**P9**

Yeah absolutely without more funding I mean I know, we’re all working and not being funded. I’m in a number of these programmes, so I do the data for there’s like a cardiovascular disease prehabilitation and diabetes remission – really similar, they’re all population health projects, but this one is certainly the one that takes up most of my time and if the other projects started wanting the same amount of time I’d say that would be challenging

**SB**

Why does this one take up more time?

**P9**

That’s a really good question. The other ones don’t have the same email referral and things coming back to me, the other ones all look after their own data. Because this one is unique in that it deals with outside agencies I think that’s probably been the reason why it, there’s a bit more on me in it. The other ones have you know like a sharepoint site where they can all just type up their own data and just upload it there whereas our move more coordinators because they’re external aren’t allowed access to our sharepoint sites or anything like that, so again IT I don’t know if that’s something IT can work around, but I’m sure there’s other areas in the trust where we’re sharing data and we’re all using the same patient data with an external agency, and I’m guessing it’s possible. Maybe it’s not and I’m just being hopeful, but it's the engagement with IT would really help.

**SB**

No, fab. Thank you so much P9, have you any final comments before we stop the recording?

**P9**

No, I don’t think so. I think it’s been a really good project and I think we’ll learn a lot from it even if we don’t see the, and it might be disheartening to people not seeing the results that we were expecting but certainly I would want to be focusing on the patients who are coming back with comments about how good it is and things like that instead, so I am very conscious that people might be disappointed when we look at the results, but yeah it has been certainly beneficial to patients in other ways.

**SB**

And I think that all has to be measures, put against the fact that the covid backdrop and it has been very challenging, so it’s still learning regardless.

**P9**

No absolutely, and I think it has, it certainly helps some of the patients. There was a news report that a couple of the patients did, I think it was on UTV live or something like that and it was really lovely hearing them talk about it. So we maybe don’t have great data, I think it was two patients who were on it, although we don’t have great data for them it obviously meant something that they were willing to go and do a news report, have a tv camera in their garden, you know

**SB**

Absolutely. Well I am just going to stop the recording then

# P10

**SB**

Yeah. Okay. So just to begin, what did you know about cancer prehabilitation before this service started in the South Eastern Trust?

**P10**

I didn't really know that much. I am part of the emotional wellbeing support service. So I regularly provide service users with about six sessions of emotional support, because I'm an Assistant psychologist, and because of that, I was asked to be part of the cancer prehab, because a lot of the things that we would do in that service would would transfer well in with cancer prehab and helping people with their anxiety coming up to their, their treatment. So I didn't really know that much about it, I was actually just sent a referral out of the blue. And I didn't even know what it was for. And then I asked the people you know, why have you sent me it? And they told me, basically what I just said, so I didn't think all that much about it beforehand.

**SB**

Okay. And what is your view of engaging patients before surgery?

**P10**

Well, so I only have a bit of a limited experience with it because I've only had one, I've only engaged one with one patient, well, I've engaged with technically I've engaged with three but I've only went through the full process with one. So that was before surgery, I've found that the person that I engaged with was very, she was feeling very angry, she found it very difficult. And I think it was because her surgery plans had been cancelled for a wee while because it was there were more tumours that came up she had lung cancer. So she was feeling very angry and annoyed about that and found it very overwhelming and she was transferred her treatment plans were changed to radiotherapy I think was changed to. So. So it was trying to work with work with her feeling angry and, and anxious about what the future would hold. It was very open ended, I think. And she was trying to do things like organising her affairs and stuff. But she found it very .... her mood would would change quite quite a lot. So she would be feeling good one week, the next week she would be feeling very, very low. But that was my experience with that broadly, just in terms of our mood, and how she was, you know.

**SB**

Do you think it's important to engage with patients before surgery? Do you think it's a good point in time?

**P10**

Yeah, of course. Yeah. Because there's probably there's a lot of from what I could tell, engaging with that person. But even as I said, I had phoned two other people to try and to work with them. But it was one had already had the surgery, it was actually two of them already had the surgery at that stage. So but they from, from what I had been told that they were very, very anxious, and they were really struggling to, to manage the upcoming treatment process and what it would involve and how they would, you know, regulate their own emotions and stuff around that. So I think it would be useful. Yeah. Especially even around the the evidence basis and stuff around anxiety and helping with that pre-treatment anxiety and stuff. I think it would fit in well with that. Yeah.

**SB**

And would you give me a wee bit of an overview of your role in the cancer prehabilitation programme?

**P10**

Yeah, so I am an assistant psychologist I, on a weekly basis, I would be in contact with people who are experiencing mild to moderate low mood and anxiety because of physical injuries and illnesses and things because of the pandemic, they're quite restricted, and they weren't allowed to leave their home and stuff like that. So that's I provide a structured six sessions, emotional support for them over the phone. So because of that, it was thought that that would transfer quite well to the cancer prehab in terms of helping them talk through their, their, their emotional reaction to it on how they were going to manage their anxiety and things like that. You know, so that's what I do yeah.

**SB**

Okay. And were you involved in the sort of development of the assessment referral pathway?

**P10**

Not really, no, I was a wee bit, in terms of this, no, not No, I wasn't involved in that. No, no.

**SB**

Okay. And were you involved in sort of the planning stage of the project?

**P10**

The cancer prehab, no, I I wasn't involved in that either. Maybe I should, again, see there was a breakdown in communication. Maybe I should have been involved in that a wee bit I don't know what happened. It maybe was just a breakdown or a bit of a breakdown in communication and communication. But I wasn't involved. No.

**SB**

Okay. And in terms of the I know, you've said that you've been involved with other patients with cancer. for this programme, specifically, did you receive any specific training?

**P10**

For this for the cancer prehab? No, no, no.

**SB**

Okay. And is there any way on reflection I know you've mentioned you've only had contact with a few patients? would there have been any way you could have been better prepared for this role?

**P10**

Yeah, I think so. I think. And so coming from what I would do on a daily basis, it's all very it's all very based on motivational interviewing and on helping people manage, you know, as I said, mild to moderate, you know, low mood and anxiety and stuff, which is, which is train you know, I know how to work with that, and that can be a big part of, you know, the cancer prehab, it's all very relevant. But I think I would think more training maybe would have been useful in terms of very specifically, you know, working with people who are experiencing the pre-treatment anxieties and stuff, because I've never really, I've never really worked it, in terms of my role normally wouldn't really involve people who out of the ordinary wouldn't be struggling with their mood you know it's very specific to cancer treatment. I think maybe some training around that, and what I would what would best what, you know, what, what I should work with, you know, in terms of how I should help them best, would have been useful I think,

**SB**

That's an interesting point, particularly going forward.

**P10**

Even even knowing what their pathway .... oops did I freeze there? Sorry

**SB**

Yes, you froze, and I spoke and then I was talking over you so sorry.

**P10**

No, you're ok go ahead. No, I was thinking even emm even to know what, what their treatment what, you know what, what they've been through to get to this stage. Because really, I was just getting a referral email with their name and who referred them and not even I wasn't even, I wasn't even told exactly what cancer that they were being treated for. So I was just it was just a person who was I was told was being treated for cancer. So maybe some sort of training in and around you know, what, what process they were being brought through. Maybe some common anxiety symptoms that they might, you know, that people might find themselves in in terms of pre-treatment.

**SB**

That's, yeah, that's very useful P10. just there, you began talking about there some of the challenges with the screening, referral and consent process, and one of those being a lack of information for you as a clinician offering the support. So what would you like to see what what would you like to see different in that process?

**P10**

Yeah, well even I think? Well, now, now, I've been, I see I wasn't even part of the I think they have they have a monthly meeting with the team. I wasn't even part of that. Now I don't think that I don't think it was on purpose. I think maybe people just didn't know, you know, so now I've been invited. I'm like, I haven't joined one yet. But I'm gonna plan on joining one but as I said, maybe being just understanding the full process of the person being referred and taken in and what that involves for them and who they will be in contact with. And, and then even, you know, right to the very end, do they have follow up sessions with people? I don't know, I have no idea what goes on, you know.

But even that, I think, would be good is a database for where I can keep notes and stuff. So with typically with my other service users, I have a database a shared a share point where I can store confidential notes so that for each of the sessions, I would type up notes, you know, but I don't have that anywhere for for the cancer prehab patients, I just keep them on my own in folders on my own, you know, trust system, which probably, I don't know if that's even, it probably be more appropriate for, you know, a SharePoint or something where I can save them to. So something like that might be useful. As long as the training you know of the process, training for myself in terms of what they're what, what I should be working with, like models and stuff I don't know I don't. Yeah.

**SB**

Okay. And in terms of information, you touched on what information you would like to see coming through with the referral. What do you what do you get with the referral now? you said name, and sometimes you're not even sure what the tumour is?

**P10**

Yeah, it's like a referral, like an Excel spreadsheet. So there's their name, their contact details, obviously, I think there's who referred them. There's some other assessment details that I think that people, I think it's an assessment form that somebody else has used or something and they just forward it on me. But it's, it's not. It just says, sometimes it says the general area of their cancer or something like say, say, neck cancer or something, but it doesn't say maybe what treatment is being planned or what area of the because I'm sure it's very intricate. I'm not a doctor, like, but it would be. Maybe it would be helpful to know some of that stuff. Because obviously, depending on where on your body, it's gonna be, it's gonna have a big effect, different effects, you know? Yeah, so I found all that, it was just a wee bit maybe I haven't looked into the spreadsheet enough. But it was. It was kind of that was it was, basic information from what I could see.

**SB**

Yeah, yeah. Yeah. And were there any other problems with the referrals process? You'd mentioned earlier that you weren't able to see all the patients in time was that maybe a problem?

**P10**

Yeah. So to start off with I had I didn't have access, there's there's an email, an Outlook email, that all the referrals go into, and even my own work referrals that are separate to the cancer prehab, but for some reason, I wasn't included in that email, somebody else had been administering it, or administrating it before they left, you know, so they left and I wasn't included, I didn't, nobody told me to pick it up then. So because generally with my other referrals, people phone me and tell me about them. So I didn't even know it existed. So the thing was, then I had received my first referral through email, and it just came out of the blue, I didn't even know what cancer prehab was, so I just picked it up and I worked with my usual model that I usually use. And then about, I think it was in January time, the same person that referred the first one said, is there a waiting list with these referrals or anything, what's happening with them, and I didn't know I so I had to get back then and said, I don't really know anything about this is there are there more people waiting? And then they had I had to go through the whole process of trying to get set up on the system. They told me that there was a system, I tried to get set up and eventually I was set up on it and I had access to these whole list of referrals. That included my own referrals that I already knew about, but also some cancer prehab that I didn't even know were sitting there. They had been sitting there for months. So what I did was I contacted the person that referred them to me. And just to make sure if it was still appropriate, because probably they had already received their treatment and gone through the process, unfortunately. And it turned out that unfortunately, the majority of them I think there was about four of them. They weren't appropriate anymore, because the time had passed. And one was appropriate. So I've, since I contacted them and arranged that but it's just they were they, they fell to the wayside because of the breakdown in communication.

**SB**

Yeah

**P10**

Again I don't think it's a fault of anyone. I think it's just maybe trying to get it all pulled together. I was just slipped into one of the cracks, you know…

**SB**

Absolutely. And luckily have identified that crack. So yeah, for moving forward. Yeah. So absolutely. Very, very important. With the patient that you did engage with how interested were they to engage in the prehabilitation session?

**P10**

Yeah, they were, very interested. I think before she had her diagnosis and stuff I had to she had, she had struggled with some mental health problems in the past, which I think a lot of people do anyway. So I think she was she was open to it. Yeah. And we done six sessions. And she was very she was very, she engaged very well. And mostly, we kind of just discussed, you know, how she was managing the, the problems in the in the treatment process, you know, that was delayed and there was a lot of every week she was well, for over six weeks, she was always telling me about a breakdown in communication between her and her, the people who are treating her and how long it was going to be. And she didn't really know how long was going to be and it kept changing there was a lot of uncertainty and stuff. So yeah.

**SB**

Was she able to get all six sessions prior to surgery?

**P10**

Yes. Yeah, I think I think I didn't even know then at the end of it was she and as I said, I think her treatment was changed. I don't know if they were planning on doing a surgery at the end of it. I think halfway through the sessions, they started she was she was like to take radio, she was doing radiotherapy or something and she was taking some sort of medication.

**SB**

Okay. Yep. this maybe doesn't apply so much to you just because of the lack of referrals, but what impact has COVID-19 had on service provision in terms of the cancer prehabilitation? In terms of that one person, was it face to face? Was it?

**P10**

No, it was on the phone, so we generally I would, I would just talk to people on the phone. That being said, or if they were open to do zoom, I would do zoom, you know, I'd be okay with that. But generally because we've transferred the model from the emotional wellbeing service onto the cancer prehab all the the emotional wellbeing service is all done on the phone. So that's really why we just stuck with the phone. Yeah. So what was your question

**SB**

Just about the impact COVID-19 has, but that's maybe not

**P10**

Even for her I when I was talking to her, like, I don't know if it's relevant but she was I think she was a wee bit frustrated not being able to meet face to face, she was just she, she mentioned it a number of times, because she was also receiving help from MacMillan Cancer as well, they were like a befriending service. But again, they were only on the phone. So she was, she mentioned a few times how frustrated she was with not being able to meet somebody face to face. You know, have that part, that connection, I suppose. And have the craic with somebody, you know, walking through the town or something even or just, you know, sitting in an office or something I don't know. But that was I think she really did a lot over the phone and maybe she felt that it wasn't as effective.

**SB**

This next area is looking about your professional develop development and practice as a consequence of being involved in the cancer prehabilitation programme. I think that's probably quite limited, because you I suppose hearing from you you're not feeling that involved, you're kind of being left behind somewhat. And and you're only looking to join the working group now you know, that invites now out. has there been any sort of in terms of your engagement with that patient and the topic? Do you do you feel you you've had some professional development as a consequence?

**P10**

Yeah, of course. Working with people from... It's great to work with, with people from a whole range of different not only backgrounds, but you know, coming from a different referral process, because they have like, she was very specifically and the other person who I had I had talked to on the phone but didn't need treatment. They're very specifically coming from, you know, the cancer prehab it's a very specific thing they're managing a very specific physical problem whereas I would usually be working with people who are just have mental health problems or, you know, just as a result of just in general in terms of life, you know, but I think it was it was good experience. Yeah. And working it was very specific. I think, even to make it a better experience would be some sort of training around that. Yeah. If they had some sort of training that would help you specifically work with those with people who are, you know, have this thing coming up as big treatment this cancer treatment, even the experience of having cancer and how that affects your mood? And how to help improve your mood around that would be really good. I think I would have thought would be a good thing for me anywhere. you know

**SB**

You've mentioned a few things as we've gone along about improvements that you think which could enhance the sustainability of the cancer prehabilitation programme, is there anything else you want to discuss around that? you've mentioned the likes of training, and maybe sorting out the system to ensure people aren't falling through the cracks?

**P10**

*inaudible* well I think training would be the first port of call and have a good, you know, include everyone in the system from referral to you know, so they all know what's going on. I think then, obviously, well I think they're doing already aren't they, they're having those monthly meetings being included in those would is really good. I don't think that I can't think of anything else. I do regular supervision as well. And this comes up, but that this is this comes up on it as well so I've got that.

**SB**

Do you think there's anything if this was being rolled out to other trusts, would you have anything that you would like to see happen to make sure it works better in other trusts?

**P10**

Maybe, maybe? Maybe, you know, the way I'd said that the EWS the emotional wellbeing model that we use is just basically been transferred on to cancer prehab maybe maybe a more specific model that's designed. I don't know if it'd be much different, but it might be helpful to think through them. That a more specific model, you could, you know, maybe a clinical psychologist would would be able to work with that one. In other trusts then I think just the access, I think it's useful to have access to one email system with all the referrals are being sent into again that's a referral process isn't it?

**SB**

Yeah, but that's still it's still part of the implementation. Yeah. Is there similar roles to yourself in other trusts would this transfer across do you think?

**P10**

Yeah, yeah. That's why I'm an Assistant psychologist. Yeah, I think inmost of the trusts, they have assistant psychologists. And, yeah, our background and our training and stuff would lend itself really, really well to this I think. You could even you could even think about a role like an assistant psychologist role solely for that, almost probably, if you had enough referrals, you could probably have, you know, especially face to face, zoom, phone calls. you probably would, you could you could fulfil you could fill a whole role there just basically in cancer prehab if it was if there was enough people to go through it, yeah. I can't think of anything else

**SB**

That's alright, are there any barriers you can see for the long term delivery of Cancer prehabilitation?

**P10**

I'm not too sure about waiting lists and stuff, but I don't know if waiting lists will have a big impact, you know, like how waiting lists are going to they're all over because of COVID. There's a huge probably backlog of all these cancer treatments. I don't know how there may be a big influx of people really struggling? Well, there probably will be, you know, a huge influx of people who are going to be struggling with their mental health around this because of serious waiting lists and having been on the waiting list for a long, long time. Like that lady who I worked with, she was really overwhelmed and very frustrated with the changes and lack of certainty around it. I could see that really becoming a problem down the road, because we're all told aren't we that there's going to be a big impact and how it's going forward. So it might, it might be a bit overwhelming for the staff.

**SB**

Yes, and possibly require additional resource, and possibly, but I think just your point there in terms of that potentially could be more people. But that could also be more people with a later diagnosis.

**P10**

Yeah. that changes things. A later diagnosis, because that's obviously to change the outcomes of it, and all that type of and all that stuff. So that and that is going to have a big impact on how any psychological treatment or psychological or pre-treatment anxiety is just going to be huge the variation. So again, that that would be around training on how to be prepared for that for all, not only just, you know, people like myself, but probably everyone in the whole referral pathway.

**SB**

That's been really interesting. I think you've raised quite a number of really helpful useful points and improvements and things to consider for the future of the project that really, really has been useful. Have you any final comments, you want to make anything that I didn't ask that, you know?

**P10**

Don't don't think so I think we've covered it all. I think it's a really interesting service. Like it is very interesting. Like if I had more training around that and I was involved more and I know it's hard because of COVID and we're all very strict, restrictive in terms of being able to go places. Well, if you're more involved in the process, it will be a lot more fulfilling, I think, you know, than just getting the phone call, like just getting this referral on email and then ringing them you know. Yeah, I think being more involved in the, in the referral process and stuff will be useful. Yeah. And training, I think I’ve mentioned that

**SB**

Okay, here, thank you so much. I'm just gonna stop the recording.

**P10**

Yeah.

# P11

**SB**

The recording. Okay, so just to kick off, what did you know about Cancer prehabilitation before the service started in the south eastern trust?

**P11**

Before the service started, I knew a little bit about cancer prehab, because I had been involved and we put a piece of work that we had undertaken just prior to setting up the initiatives, we had done some work around what the evidence was, what was best practice. So from that, I did know some some research and some evidence and some practice that was sort of in England, and sort of further afield. But prior to that, and that involvement in trying to start off this initiative, I would have known very little around the whole physical activity, the benefits and that cancer prehab. I mean, obviously, I would have known that the fitter you were, and the healthier you were, the better your outcome. But I didn't realise in that short window that actually the difference that physical activity could make with regards to an outcome. So yeah, yeah.

**SB**

And what is your perception of the project now that it's been going for the last year?

**P11**

So I've obviously been very involved with the project from the very start. So I suppose one of the things for me, that has been that's been really, really encouraging has been the partnership work. I think the partnership work has been absolutely fabulous. When we first started to talk about this, we did discuss this with the director of hospital services with the assistant director. So I think at the very beginning, we had buy in from that level, which was really, really important. And I think one of the key things that has made this work is that we involved all of the partners, whether that was through, you know, our council move more coordinators, cancer services, psychology, dieticians, were involved from the very, very beginning. So people were on the journey together. And I think that has been key. It has been bringing people together. But I think that that sort of commitment from senior level has been really important that they were committed and they had given the yes, we want to take this forward has been key to it SB. I think as well that for our partners, especially our move more coordinators with the council I think making them feel that they were such well they are such a vital part of this. But actually having them at the sort of weekly meetings, the the monthly meetings, inviting them along into the senior cancer services meetings with the assistant director with staff has really empowered the girls to feel that they are such an essential part of this. And they're not been excluded from those discussions and those meetings that are ongoing. So and I think having the partners that we have around the screen, or around us, has been around the screen and not around the table have have real belief that this is the right thing to do. There has been very little resistance from from, you know, the cancer has been no resistance from from from the partners. And I think that everyone having the same vision, the same aims, the same objectives have been crucial.

**SB**

Yes. And P11, you said there that you were involved in those sort of senior level meetings and working group. So what what was your role in those meetings or in the project development

**P11**

In the project at that time, we had an assistant director and I was the head of health improvement within the trust. So I would have been, well all via zoom, but I would have been involved in the meetings with our director of hospital services, and with the A.D around trying to well just really given them an overview of what how the vision aim and that we had for the cancer prehab service. And basing that on the model in Manchester. So it was really going along to their senior management team meetings, giving a informal presentation, and really getting their support and their buy in their, their sort of okay to say yes, we're happy that you take this forward because we're coming from health development, a different directorate. So we needed to get their buy in to say, you know that we will. It's a partnership. So I'm not going to say that we will lead on this, but initially, I suppose we were pulling together the partners and sort of putting our vision around how we can replicate the Manchester model. So it was key that we had those meetings at senior level

**SB**

Absolutely. And fantastic buy in that you did get. And in terms of selling this cancer prehabilitation programme up, when you look back is there any way you could have been better prepared for your role in doing this

**P11**

I, I could be better prepared for my role. I think that I don't think that I could have been any better prepared. I think that the, the model that we used, we had Manchester to base a lot of our work on. And we have also another a number of other initiatives where we're using operating procedures, we have tasks and finish groups. So I suppose for me, we were taking the learning from what we had done with other services that we had sort of that were new services, and the partnership and how we were having the regular meetings, we tried to replicate that with cancer services, because that had worked really well with other services. So I suppose I took the learning from other things that we had done, and then tried to really embed those within the cancer services, the prehab initiative

**SB**

And in those early days, this is slightly off off point. But did you have any meetings directly with the Manchester team? Was there any zoom meetings? Or?

**P11**

Yes, there was we did have. I'm trying to think now, we did have a very initial meeting, this has gone back a long time, before we decided what we were going to do. And we did have a meeting with. And I can't remember even who was there. I know that the assistant director was there from hospital services for the cancer that covers the cancer services. And we had a presentation from I think it was two girls, if I can remember rightly, from who were rolling out the Manchester model. And they then had told us some of the areas where they had for improvement. Some of the areas that they suggested that we they had tried and didn't work. Now their model was slightly different, obviously to ours. And we knew that we couldn't implement the Manchester model exactly as was being implemented in Manchester. But we did take the learning from what had worked well for them and what hadn't worked. And try then to embed that into our operating procedure and the way forward.

**SB**

Interesting. And can you think about what some of those improvements they were suggesting, and that you did actually implement?

**P11**

I think I can't

**SB**

I'm putting you on the spot, I'm really sorry

**P11**

I can't remember SB, this is awful. I think a lot of it was around, or some of the discussion that we had was around the outcomes. And around that, you know, from the very start of the initiative, that we were very clear about the outcomes that we wanted to measure and how we were going to measure it. They did if I remember right, it emphasized quite a bit on the data collection, and the data collection methods. And, you know, really made us consider, you know, what, what validated tools right there. And we they were able to share some of the tools that they were using. But they made us really then think about who was going to collect the data? How is the data going to be collected? And how was the data going to be presented. And I think from that what we did is that we involved our informatic team at the very, you know, it wasn't exactly at the very beginning. But as soon as we started talking about data, we took the learning from Manchester, and we got the informatic team involved. And as you know, then [PERFORMANCE TEAM MANAGER] does a lot of work around the data collection. Now we're not fortunate, we don't have that input from informatics and other initiatives simply because of capacity….

**SB**

Okay

**P11**

Because [PERFORMANCE TEAM MANAGER NAME] got quite a small team. But [PERFORMANCE TEAM MANAGER] has been instrumental in us collecting the data and then having that data, actually to show the outcomes. And then we do report cards as well with our, you know, our outcomes on the report cards. So one of the learnings was around the data, what data we would collect and why we were collecting, who we're sharing with it, and why we were going to input that into some kind of a whether it be a spreadsheet or database. I think Manchester really stressed the importance of that. And probably we hadn't considered how much work that was going to be SB or who was going to do that. Because we didn't have any additional staff here. We didn't have money for a project coordinator or a manager. So we were doing this on staff, with staff who were already in post. This was an addition on to other staffs work, so we had to think about how we were going to collect and share the responsibilities,

**SB**

And it's incredible what you have achieved on essentially goodwill. And

**P11**

Well it has been good will, we haven't with this project, other than, obviously, we've spent some money on training with the move more coordinators and equipment. But other than that, there hasn't been that that's been it.

**SB**

Yeah. It's fabulous. So it is really, really incredible. And thinking about then just over the last year, what sort of challenges have you come up against with the project P11?

**P11**

I think obviously, one of the challenges is COVID, where the move more coordinators are, have been, are not able to do a lot of the work face to face or in group sessions. But ideally, this would have been either done, you know, the initial assessments and the the, some of the the, the, the, the the initial assessment that meeting that building up the relationships, the physical activity programmes or initiatives would have been actually done one to one or in group sessions, or they would have been signposted to leisure centres where they would have done whatever they had agreed to do with the move more coordinators. So obviously, because of COVID, none of that could happen, and everything has just been done via telephone or via zoom. And I must admit the move more coordinators and their enthusiasm. They have gone above and beyond in trying to connect and get the engagement with the client.

So COVID has been a huge challenge for us over the last year. I think as well we have seen, we had sort of worked out how many referrals we would have got into the service. Very roughly we didn't know for certain, but obviously sometimes COVID impacted on the number of referrals we were getting into each of the from the service from the cancer areas and into the service. And probably over some months we saw a peak. And then others we saw decline depending on what was happening with COVID and when patients were being seen or. And we also saw that for some of the cancer sites, patients were actually presenting quite late. Now whether that is COVID, I'm sure [CNS HNC] would have more research into that than I would have whether that is COVID related, or whether people just sometimes are late in presenting. But some of the cancer sites, patients were presenting quite late and the window for prehab was either very, very, very slight or non-existent because they were going straight into treatment or surgery. We were very lucky that during the whole time we we only recently had one move more coordinator who has left and someone else has now taken up their post. So we've had consistency, which has been has been really good.

The other thing we have been a challenge for us is with one council area, not one... Yeah, one council area around the funding for the post. Because initially, initially we had said we were doing this as a pilot, and that we would like to pilot this for one year, obviously then we extended and we did have some discuss a lot of discussions with the council who were actually going to pull their the person from even though they knew there was no objections from the council about this not being the right thing to do. And this the outcomes were successful. They it wasn't around that it was an it was an objection as to the funding of the post. The person, you know, should the funding come from council should come from health. There was a lot of debate around that. And a lot of there was no a lot of conversations, a lot of debate. And then we were very lucky because there was more money put into the MacMillan Move More coordinators.

**SB**

oh fabulous

**P11**

And that was put down to each council area. So that issue now has been resolved. But there was goodwill from that council saying, well, we will extend for a further six months. But you know, we do want some confirmation of funding. So there was that sort of negotiating with Macmillan trying as well to influence them to try and get the funding agreed and down to councils. So there was a wee bit of uncertainty SB there but I think because we had good relationships with all of the councils, I mean, it's very rarely for councils actually saying you know, there there's the move more coordinator. And yes, go ahead.

**SB**

It probably speaks as well to how you have conveyed the importance of the work?

**P11**

Well, I think what we did is we we met with each council separately. And we talked to each council about the … our sort of vision of a cancer prehab service and that we did talk about alternatives. SB, we talked about, you know, if we did have funding at that stage, we didn't know, you know, if I'm going back two and a half years ago, we were in the planning stage, we didn't actually know if we could get funding. And we would employ for a two year period our own sort of like physical activity coordinators. I mean, this was all up in the air, but when we spoke to the councils and we spoke to them all individually, the councils were saying, and we knew, but we do have move more coordinators here funded by Macmillan, we can actually negotiate and look at their workload, look at their capacity, look and see how many referrals you're talking about for each council area, and then just trying to work with the trust and the move more coordinators to deliver the service. And I think again, it was that early negotiation with the with the councils I think, as well, it's our pre our relationships with the councils were good anyway. And I think, you know, we were saying, well, you know, if it's, you know, for some of the council areas we're not talking huge referrals. And if your staff are willing to deliver the program, we will pay for the training, we will pay for the resources. So there was a bit of give and take

**SB**

Yeah.

**P11**

Around that, and I think it raised the profile for the councils because they're able to use the report cards as well. I mean, we're happy that they share those, you know,

**SB**

Yeah

**P11**

They go back up to their chief executives, they use it for community planning, they can do what they want with that it's a partnership. So I suppose there's been gains for both.

**SB**

Yes. And just just on that, then what about the gains for the partners around the table P11 from your point of view, what do you see as the gains from this this project? Not only obviously, there are gains for patients, but from the partners point of view from the stakeholders?

**P11**

Well, I think obviously, as you said the gains to the patient, as you know, is there to see. I think for the partners, I think that I think for the move more coordinators, and for the councils it is that additional training, the additional resources, I think, as well that for the girls actually delivering the programme, it is that they had definitely have built relationships within the trust, to a degree that they wouldn't have with any other service. I mean, it's very rarely that you would have council staff who are delivering a programme sitting down at a senior management team meeting with directors and assistant directors, and consultants. And they and their input is as valued as anyone else’s round that table.

**SB**

Fabulous

**P11**

I think for the trust and the council, obviously, it's building on our we do have strong relationships with the councils. But I think for the partners as well, you know, we do have our report cards that go out each quarter. This is looking at how we're improving health and reducing health inequalities. The report cards are used internally within our hospital services as part of our early treatment centre and the work that we're doing under the early treatment centre. And the report cards can also then be shared with our partners and can be used for things like community planning and locality planning and neighbourhood renewal So there's, I think there's lots of gains.

**SB**

Yes, yes. And yeah, just I think it's incredible that the learning that various different practitioners that aren't sitting down together have been able to get in that, you know, through that working group as you say, yeah, it's not not the normal way of things.

**P11**

No, I think one of the other things that came up SB with the council because obviously it's Newry, Mourne and Downe, and South Eastern Trust sits within the Downe locality of Newry Mourne and Downe council. So the Newry and Mourne side sits within the Southern Trust. So there was discussion, so when this when the cancer prehab was sort of operating and we were seeing patients going through. Obviously there was talk within the council around equity of services for those living in Newry Mourne and Downe district council. And the services that the council was supporting was not equitable, because this service was only open to South Eastern and Downe part of the Trust, or the council. So we did at that stage then approach the southern trust, because obviously, we worked very closely as a trust. So we did approach the Southern Trust, and we spoke to our equivalents within health improvement. And they then spoke with their cancer services, and hospital services, sort of consultants and managers and directors, et cetera. And what we were really looking was, could they replicate in the Newry and Mourne side what we were doing in the Downe side of the trust? Of the council?

**SB**

Yeah

**P11**

and I suppose SB, it was the middle of COVID. Because we had been able really to get this off the ground just before COVID. So we had all the prep work done.

**SB**

Yeah

**P11**

We were approaching the Southern Trust, then sort of first wave of COVID. And it wasn't the right time, they were saying, look, all our staff have been in health improvement are being repositioned or redeployed cancer services, were saying, We cannot look at this at this moment in time. So that is something that we would be very keen that this will be scaled up not only to Southern Trust, and the Newry and Mourne side of the council but actually regionally Because this really is an inexpensive way of delivering a fabulous service.

**SB**

Yes, absolutely. Absolutely. And it's interesting how whenever a service breaks those council boundaries, that, you know it pricks interest and hopefully that'll be the catalyst for it moving forward in other areas.

**P11**

Yeah.

**SB**

You had mentioned some of the, you know, the issues as a result of COVID and it's impact on the likes of the move more coordinators. Is there any benefit that came with COVID-19 in terms of getting the project off the ground?

**P11**

I suppose with. Well, I suppose one of the benefits of COVID-19 was zoom, we all went to zoom. And before that, you know, we were not using zoom, we were not using any kind of platform for meetings. So I suppose, you know, when I think back to prior to zoom, or prior to COVID, you know, with the cancer, we were getting this established, we were meeting on a weekly basis every morning at nine o'clock every Friday morning at nine o'clock. And there may have been meetings in between that depend on task and finish groups, then we would have had overall steering groups. And the the attendance of those meetings was always like full attendance you know, the screen was full? Yeah, I think that Zoom has been one of the benefits of getting this to where it is today. If we were to try to arrange those meetings every Friday in the Ulster hospital for nine o'clock, we wouldn't have had the attendance. And I think it would have been much more difficult. So definitely been able to use Zoom has been, it really has, I think got this off the ground much quicker. So I think that's been one of the one of the benefits of COVID as such.

I think another thing has been that it has made us sort of sit back and think going forward, how do we deliver the service? Because I know within other services that we would deliver, we are looking at, do we do a blended approach going forward? You know, what is the best way going forward? So with the move more coordinators, as well, that discussion will take place? Because as a trust, we're still quite reluctant to do group, you know, to be promoting anything that's sort of group and face to face, etc. But I think going forward, it will be, you know, is a blended approach, the best way to take this forward. Can the initial assessments, for example, be you know, telephone or zoom or is that face to face? Because I think that you know it has reduced on travel, it has meant that the girls are probably able to see more clients. But again, face to face is hard to beat when you're trying to build a relationship. And a lot of the girls will see the patients then hopefully post….

**SB**

Yes.

**P11**

Surgery or our treatment.

**SB**

Yes. And I suppose there's there is that potential benefit as well for the patient who doesn't want to risk getting any sort of infection

**P11**

oh yes definitely. With COVID.

**SB**

Yes. So there's, yeah, there's an added benefit there, not only for travel.

**P11**

But I think it's made the meetings much more effective and efficient that people aren't having to travel. You can start a meeting at nine o'clock. You know, we're down now to about 40 minutes for those meetings on a Friday morning, maybe 45. Now, there's meetings that take longer, but I think there was a lot of work with task and finish groups that was able to be completed much quicker, because you were saying can you do a zoom between one and two, you know, it was much easier to arrange meetings.

**SB**

Yes, absolutely. Yeah. And in terms of your professional development of practice, and as you know, I can't even speak, in terms of your professional development and practice what have the benefits been from being involved in the Cancer prehabilitation Programme.

**P11**

My professional development, can I say that I used it as example, when I went to this interview? *laughs* of partnership work. I suppose for me, obviously, I've learned a lot more about cancer services. Because because I'm a nurse, but I'm not I'm a midwife, but I didn't work in cancer services. So I suppose my own and I am on still on the NMC. So I suppose for my professional development, I have learned a lot more around the work of the cancer nurse specialists. And also then, how this how psychology is an essential part, now I know there is counselling or there's other services. But we have sort of used psychology as well and our assistant psychologists around emotional support. And we have also used then our dietetic colleagues and physio. So I suppose for me, I always knew there was a, you know, a multidisciplinary team that wrapped around a cancer patient. But I suppose for me, the learning has been that sometimes that doesn't have to be your clinical psychologists or your counselling, sometimes it just needs to be someone who needs just a wee bit of extra emotional support.

**SB**

Yeah.

**P11**

So and obviously, for me, sometimes I think, as a nurse or midwife, you know, sometimes you're very much a doer you what you want to get done. So for me, this has been the process. And, you know, from start to finish, for my practice development and the importance of, well, we always would have used outcomes and operating procedures. But I suppose for me, I think it's just I'm quite practical, I want to get started and get it done. For me the learning has been actually, you do need to take the step by step and you need to get it right. And that might take a little longer than what you thought, but but I suppose you know, as I said, for my personal development, I have been able to use this example. And that for me, it's certainly a great win because it's such a strong example of partnership working.

**SB**

Absolutely. And you're saying all that P11, with a smile on your face. Have you enjoyed being part of the project?

**P11**

Oh, I love it. I love what I do. I do. I have enjoyed the cancer prehab. I mean, I you know, [CNS HNC] has been instrumental as well, I think when you get someone who is quite strong in her leadership, and her direction and her focus. So I think that [CNS HNC] has been absolutely fabulous, as have all of the cancer nurse specialists, and the AHPs and everyone else around the screen. I've really enjoyed it, we have I've really enjoyed the involvement that health improvement have had. And that strengthening of our relationships between cancer hospital services, and health improvement. And I've really enjoyed even the work on the operating procedure, the discussions with the councils, even though sometimes they haven't been easy discussions. I have enjoyed it. And I've enjoyed seeing, I've enjoyed seeing something that has happened relatively quickly, from conception to delivery.

**SB**

Absolutely.

**P11**

I get quite frustrated with things that are suggested regionally or whatever and two years later, you're still talking about it. that drives me insane so I lose interest. And think, oh, just let's do it ourselves. I think sometimes. Sometimes you have to bite the bullet and do it yourself. You know, we did have discussions with other trusts about this other trusts were aware of we were doing this, they thought it was a great idea. But if we had waited until everybody had come aboard, we would still be waiting today. So I think for me the enjoyment is that we saw this we saw it as an opportunity. There was the buy in from all the partners and now it's been delivered and the outcomes are being measured. And you know, so for me that has been one of the great positives is how quickly it has gotten off the ground.

**SB**

Yeah. What improvements do you think would enhance the sustainability of the Cancer prehabilitation Programme? Thinking about for the South Eastern Trust, but also thinking about widespread implantation?

**P11**

Well I think for south eastern trust the thing that we have to remember is and this is always our problem SB, we have to think about recurrent funding because the funding that currently is going through MacMillan is until, I can’t remember is it 2024/2023 with the move more coordinators. So that’s going to sort of hang over the top of us and we need to be looking at recurrent funding for the move more coordinators

*gap in recording*

**SB**

The recording has paused here. Hi P11, sorry about that I think my thing has thrown us off, just internet connection, apologies

**P11**

No not at all

**SB**

So we were talking about improvements to enhance the sustainability of the programme

**P11**

So the improvements obviously is around first of all to sustain the I don’t think there’s any improvements required with the move more coordinators but we need to ensure we have recurrent funding for move more coordinators. I suppose the improvements to sustain the programme, we’re still looking as you know around the data, the data collection and the data collection points, so we need to really clarify and be sure that we are able to collect the data, that staff are aware of what data is required, that we have the right data points, and we need to ensure then around sustainability that we are sort of meeting the outcomes that we agreed to at beginning of the initiative. So I think it’s looking at that whole data collection and data points and outcomes. I think around sustainability as well I would love to see this scaled up across the region. I think that patients regardless of where they’re living should have access to this service. So I think that part of our working going forward will be with the likes of MacMillan through regional meetings with cancer services, you know heads of service etc that we really should be showcasing the work in the south eastern trust, the process, the partnership working and the outcomes and hopefully then it can be scaled up because that is that should be done, and as I say SB there is very little funding required, except for that recurrent funding for your move more coordinators but staff are in place and the partnerships are there, and when we look at the models of care going forward, we look at community planning it is all around partnership working and actually no additional funding but what we can do as a partnership and I think this shows that if you have that strong partnership you can do things with very little money

**SB**

Yes and that’s really good take home message on this. What do you see as barriers for the long term delivery of the cancer prehabilitation programme?

**P11**

I think the barriers would be that if you I suppose barriers would be if you were to lose staff. If you do lose staff, if staff do move on, especially the move more coordinators the time then it’s taken to recruit and then you have that backlog of patients or probably in this case those patients will have gone on and had surgery or treatment so it’s not even a backlog it’s just that the patients don’t get the service. So I suppose one thing is around recruitment. I think I mean I do think that we have such a strong model if we were to lose key people key partners around the table, if key partners were to move to new roles, I think the model is embedded and I don’t think that the model would

**gap in recording**

**SB**

Sorry P11 has dropped off again

**P11**

Sorry SB

**SB**

Sorry P11, not sure what happened there but you froze. You were saying there that the model has become well embedded so you don’t think if stakeholders changed that that would have the same impact?

**P11**

No I don’t think so because it’s got a very clear operating procedure, you have you know you’re going to have, not everyone is going to leave at the same time of all the partners, so I think if you have the commitment from the partners, people know that this is the right thing to do, this is a good thing to do with an evidence base and very clear outcomes and that I think this has now just become part of our cancer services

**gap in recording**

**SB**

Hi P11, sorry I don’t know what’s happening here

**P11**

No, you’re ok, you’re grand. I suppose SB I was just saying that in case I drop out again is that the other thing we probably need in the future is

**gap in recording**

**SB**

So the other thing that you feel you need in the future is…..oh this is not good….are you back there sorry the other thing that you said you needed was

**P11**

I think we probably need to look SB at other cancer sites. Because as part of the pilot we agreed to focus on a number of cancer sites but we don’t look at all of the cancer sites, because we wanted to look first at you know we wanted to try this would this work, would this operating procedure, would what we have in place work? And we decided that we didn’t know what capacity and demand would be so we decided to focus on a number of cancer sites but probably going forward we’re offering this to colorectal or a lung patient why are we not offering this to a breast or other cancer sites? So we probably do need to look in the future around do we open this up to all cancer sites?

**SB**

Yeah. No, thank you so much for all of your insight here P11, and apologies again for the coming in and out at the end. Have you any other comments, I’ve no more questions for you but have you any final comments or anything that I didn’t ask that you want to raise?

**P11**

No I think that you’ve raised most things. I think as I said initially, I think the key to this has been the partnership working without a doubt and those relationships that have been formed. I mean we have relationships now with other people across internally that we wouldn’t have had so, you know and if we can replicate this in other cancer sites that would be really what we would like to do. But that’s me

**SB**

Fabulous. I’m just going to stop the recording then

# P12

SB 0:03

Okay, so just as a lead in here, can you tell me about your professional background and delivering physical activity activity interventions prior to the involvement in the prehabilitation programme?

P12 0:24

Yeah. So April 2017, I took on the role of Macmillan Move More coordinator, Move More programme was initially a rehab programme for anybody at any stage of their cancer diagnosis. So we would have had referrals from a range of outlets. So for example, it would be health professionals, CNS's, consultants, physios and then also self-referral, and that initial that referral then would have taken sort of four of us, and the initial one to one to sort of find it, where they were, and what sort of things that might be keen to engage with. At that point, then we would have also done kind of almost like a holistic needs assessment where by we sort of have a conversation around other services provided by Macmillan, and other charities. So for example, we would have been referred only maybe to see jobs and benefits advice, maybe somebody returned them to work, or counselling or like scar therapy or complementary therapies. So it was kind of it was always a timely service, I would say 70% 75% of the people referred to us had, or were just finishing radiotherapy or chemotherapy, you know, they were coming to the end of their treatment plan of such. And so then we were worked out were they were at so for example, if somebody have a had breast surgery, we looked at what sort of exercise would be good to build upon exercise already given by the physio in the hospital, they would have come to a range of activity. So we ran, I would have ran Pilates circuits. For those people, maybe that couldn't their class times they would then have come into the gym, or would have attended walking groups and stuff. So yeah, that that that was my experience prior to the prehab.

SB 2:27

Yes. And then what about, I mean, physical activity interventions prior to the move more? Would you have worked with anybody with complex illnesses such as cardiovascular diseases, or?

P12 2:39

No, I worked as an events coordinator, so I was seconded into the post as Move More coordinator. And I had always, I'd always worked in fittness, I worked in fittness from i was 16, and had done different kinds of, I'd taught classes, I had taken on PTs and that sort of thing.

SB 3:00

Ok

P12 3:01

And then I held a role with the Northern Trust, which was basically like looking over the, they had a programme the Managed Obesity Networkk, there was a weight loss programme. So I went and completed my GP referral, my cardio rehab, cardiac rehab, sorry, in order to better understand, I suppose comorbidities of referrals common to the Motivate programme. But I wasn't hands on, I basically coordinated physical activity side of the house so I brought on, I recruited and selected physical activity instructors, and they're basically detailled them to different groups and different individuals according to their sort of skills and abilities as well.

SB 3:55

Okay, so that's a good good range of, you know, background in terms of like, Yeah, very good, very good. Well, before the cancer prehab service commenced in SE Trust, what was your understanding of the Move More coordinators role in delivering the cancer prehab programme?

P12 4:14

So it sort of as it started, I suppose, that was a bit like, oh, we may be doing this, we may not be doing this, there wasn't an awful lot of engagement with Council. So this was would have been, we are obviously, our terms and conditions, we're employees of the Council. So there was a lot of sort of, I suppose direct contact from Trust representatives to ourselves about being involved and about what it will entail and, and that sort of thing, but really, from a Councils perspective, it was, well you do if you want to do it, if you don't want to do this, it's not in your current job description, so you don't have to do. Now, obviously, as we, as I say, we answer to our terms and conditions are with the Council. But Macmillan obviously were our funders up until a point, were really keen that we got on board, because the Move More programme was initially to come to an end in December 2021 I suppose it was put to us that in order to sustain the programme past 2021 or December 2021, that we will get on board with us because there may be money that comes from a suppose trust. In the end up, you know, money came from that side of the house I suppose, it didn't come from any trust representatives it came from, you know, the argument that Macmillan made that this was a health programme, I think.

SB 5:48

Ok

P12 5:49

So, my understanding of the role, I suppose, was to do the initial one to ones like we had always done. At that point, it was the middle of COVID. So there's no functional assessments or no face to face, it was a lot done via zoom and all the rest. Very quickly, then it was decided that there would be, a basically an agreed time frame so that we would contact them within two days of getting the referral, make contact with the person and have a chat with them about where they were at, what's sort of things they were interested in and how we could get them active, for example, if somebody had been referred for and with colorectal cancer, the focus would have been, always the focus would have been on on cardiovascular exercise, but also then, like muscular strength and endurance exercises that were specific to their cancer site so would have been core exercises for colorectal and that would have been, like head and neck exercises for head and neck cancers and that sort of thing.

SB 6:59

So tailoring it.

P12 7:01

Tailoring

SB 7:01

Yeah. Okay. And in reality, from what you thought, in the early days of what your role was going to be, is it any different now?

P12 7:12

I'd moved on. So I'm no longer...

SB 7:15

it's really different for you! [laughs].

P12 7:16

Yeah [laughs] I suppose I didn't enjoy, you know, I didn't take to the prehab. And, you know, people were given the Breaking Bad News appointment. And within two days, we had to contact them about physical activity. And I just, I mean, we only had maybe a couple of referrals a month, say, you know, at the very most one a week or one a fortnight but I suppose i just felt like I was contacting people. You know, I mean, my first initial referral was actually a woman it turned out that I knew, and I knew her kids.

SB 7:53

OK

P12 7:53

Now, her kids are adults, you know, similar age to myself. She said to me, [PARTICIPANT NAME] I've told the kids but I don't know how to tell my mom and dad, you know, this is a 60 year old woman who had been told a few days prior to it that she had colorectal cancer. So then I had to talk to her about physical activity and bless her she was so good. So let me arrange the next day or a day later to do a Zoom with her. But she was going in in two weeks for surgery, and she was saying to me, you know, she was essentially trying to get her affairs in order. So I suppose my initial my very first one wasnt a great experience. And I just felt, I mean, I had great results with people that maybe had four to six weeks to treatment. But these people that had two weeks to treatment had other things, they didn't want to speak to me that didn't want to do zoom. You know, that was the other thing was that at the time, it was launched, we weren't in face to face contact. So a lot of it was done via zoom. So people were maybe at the Breaking Bad News appointment, I can only assume that they were saying, "we're gonna refer you to an exercise specialist for a prehab programme." And they were saying yes, now all the experience all the training that we have been given by Macmillan will tell you that appointment is essentially, once you hear the word cancer, it's essentially white noise.

SB 9:21

Yeah. Yeah.

P12 9:22

So yeah, I just struggled with it. I understand that, you know, people are much more clever than may have detailed research, recorded the benefits of it.

SB 9:43

Okay, well, it's really good to hear this [PARTICIPANT NAME]. It's really good to hear that sort of very perspective on this. As I said, all the people I've interviewed so far, everybody's views are so different. And this is really interesting. And so just thinking about how you were prepared for this role. You said, um, you're involved in the early days in sort of the planning of the programme.

P12 10:06

Yeah.

SB 10:09

So is there anything that could have been done differently?

P12 10:20

No, I don't think there was. I mean, I think it did what it said on the tin and it had great results for those people that, you know, wanted to engage those people that felt the had time to engage and stuff. So no, I don't know. I mean, there's a lot of discussion we met every month about how we make it better, you know, so, you know, at one point, it was right, we'll be clearer with what we're offering in that breaking bad news appointment. And then on down the line, it was a, you're not asking them to do they want to be part in a physically activity, you're telling them that, you know, they are being part of it like that in itself went against what we had done for the last four years. So there's no way that I think he can really I mean, we were given training on how to carry out the functional assessments, the functional assessments eventually come back after about six months, and we're required to do those. So there were sit to stands and grip tests and all that sort of stuff. But in terms of the actual having a conversation, I mean, that's what we do. That was our bread and butter, but suppose in practice, it was slightly different. Because of the time, you know, I'll say most of the people that we would have dealt with in the past would have been, at least through surgery, or at least a couple of weeks down the line from that bit Breaking Bad News appointment where they had got their head around it .

SB 12:43

. Right,

P12 12:44

yeah. So, say six months down the line it was that was a, you know, that have to take part, this is really good. And I understand where the medics are coming from that are saying, you know, this is really beneficial and stuff. And it's certainly 100%. I mean, I had, I had a couple of people that had four or six weeks to treatment. And they would tell me that actually, six weeks living with progressive after six weeks of living with a progressive disease known that they have this, that they were feeling better after six weeks, even living with the disease. So I mean, that was great.

SB 13:19

Yes.

P12 13:21

If I was to take, if I was to say, how many, you know, as a rough guesstimate, how many were like that, you know, maybe not even a quarter of people were like that, the majority of other people were, you know, they weren't wanting to come in to class because of COVID. So they're exercising at home, they were walking, they were recording that stuff. And, you know, we had to make contact with them a couple of times. And, you know, sometimes they just didn't take your call, because I think you're ringing, and saying, well how are you getting on? You know, what are you doing to keep active and stuff. And our line always was like, you know, you've got this diagnosis, that would be very easy to sit down and just wait to treatment and not doing anything, however we want to keep you as fit and active as possible.

SB 14:24

Yeah.

P12 14:25

So yeah.

SB 14:27

No, no. So this started during COVID. So it was there no patient data collected, the outcome data was up just on hold then.

P12 14:41

What do you mean,

SB 14:42

do you know the functional assessments, the

P12 14:45

Yeah, so functional assessments didn't start? I think we had training maybe in July time and then functional assessments didn't start until until we were able to see people face to face again, maybe it was like May or June time, it was probably the summer,

SB 15:01

okay,

P12 15:02

before, it was launched in March, and its probably the summer before or March or April, and it's probably the summer before we were doing functional assessments.

SB 15:12

Okay. And were you able to collect all the various different assessments that were?

P12 15:18

not for everyone.

SB 15:19

OK

P12 15:19

So each Council is slightly different. So out of the four coordinators involved in the pilot programme, only one of them were able to do house calls or two of them were able to do house calls. So I was one of the cases we weren't allowed to do house calls.

Okay.

So in order for me to collect that data, they had to come in to the centre. And now they come in to the centre and it would be one to one, were masked and you all appropriate COVID precautions put in place, but some people just didn't want to do that, you know, especially those people that maybe had been told, like that was the thing at the time they had maybe been told to isolate for 10 days prior to surgery.

Yes.

So if they were getting a referral, say on a Monday or Tuesday, and their appoint, their surgery was the Thursday, two weeks later, the time I rang them they had two weeks. So I basically had two days from that point, there were, you know, it was very hard to get times fitted in as such. And then that probably wasn't removed until maybe the, I don't know. I can't remember. Yeah, in terms of that whole. But even whenever all the restrictions, not now all the restrictions have just been removed. But even whenever restrictions started to ease people were still very fearful. So it was a hard time. I mean, we were this was all based, obviously, on the Manchester pilot programme. I mean, they had a budget, they had funding, they had people that were brought in to do it. But most importantly, they were doing it in normal times they were doing it pre pandemic, whereas we were trying to roll this pilot programme out and saying oh, come and take part in physical activity, even though, you know, you're coming into the community and, you know you have that risk and stuff. So I thought it was very hard. I mean, I agree with the principles of the programme.

Yeah.

I think it's great, what everybody has done, you know, to date. I think, if we were doing at now, it would be a lot easier you know, and having those conversations and getting those people in, you know, to centres, maybe at that point, you could, you know, be able to go out to see people and on a home visit because it's my understanding that the likes of [MMC] that was able to go into homes had a better uptake. Because she was able to go to them as opposed to getting them to come in to us if you know what I mean?

SB 17:40

So you see the people that came in to you, would you have used all the assessments?

P12 17:46

Yes,

SB 17:47

yes. And if they didn't come in to you, what assessments could you use remotely?

P12 17:52

So via zoom? Yeah, we were able to do we asked them to do a sit to stand. Okay, so that was the easiest to explain. They were doing the sit to stand, obviously they couldn't do the grip test stuff. And we were able to do that. And then you could obviously then review it.

SB 18:11

Okay, okay. Um, were there any other assessments that you did?

P12 18:20

No, not really I don't think, I think you were just kind of doing that. You couldn't do the Grip test? Sit Stand, Grip test Shuttles...No, you couldn't do? You could ask them. There weight and height? Yeah, normally there would have been weighed and stuff so he could possibly record that.

SB 18:39

Okay.

P12 18:40

Its really hard to remember,

SB 18:43

So how over the six months have you seen the cancer prehab programme evolving in that time? What changed from the start to when you left,

P12 18:54

Probably just what we were talking about, you know, suppose confidence was building about taking part in community activities, you were able to bring people in and build a rapport with them. Even from the rehab point of view, doing things via zoom unless those people had engaged prior to that, it was very hard to build that rapport and really get to know people and really properly tailor a proper programme for them. So with COVID restrictions lifting and conference building and stuff people were able to come in. So for example, at my class, I had two or three prehab people that maybe we're waiting for five or six weeks, that were able to come into my class twice a week.

SB 19:34

Yeah,

P12 19:35

you know, and do programmes

SB 19:37

and was there exercise prescription as part of the development of the programme?

P12 19:42

Yes, so exercise prescription, I mean, exercise prescription, in the loosest form from the very start in that we were saying to people, right, we need you to be active you know two, three, four times a week and this is what we need you to do. So you know two or three times a week and this is what we need you to do at home and in terms of coming into the centres, they would have been doing like a circuts class, which would involve high intensity, they would have been doing Pilates which would involved strength and conditioning. And then outside of that, we would have asked them to walk as well, or to do what they had always done kind of thing. So yes, exercise prescription from that way in terms of prescription, you know, not specific in terms of heart rates, and zones and all the rest, but certainly exercise prescription terms of frequency, intensity type and time.

SB 20:38

And did that build over the programme? Did that become more intentional as a programme went along? Or was that in place at the outset?

P12 20:45

It became more intentional, because you had people coming in who were able to do that. So yes.

SB 20:50

Yes. Okay. And what about nutrition advice did that change over time?

P12 20:55

We I, I don't really recall, given nutritional advice. I, we were sort of get trained on the healthy eating plate the same way we're doing in school as such. So for the likes of colorectal patients that are overweight, you could have a conversation with regards to sort of, I mean, I had one guy, that and everybody will have their case studies, but I had one guy that was basically too heavy for treatment. And he was with me for like eight weeks, or 12 weeks, actually. And he he was living off 800 calories. But he had dietetic support, as opposed to just nutritional advice, because it was so specific, the reason why he couldn't have a surgery was because he was too heavy. And they were worried about him. Other nutritional advice, you know, like one of the men that came to my class, and he was lovely, and he was so good at but he was, he would have been a thin man you know one of those men that was always gonna have been active, you know, like my grandfather used to say, on the same size in my treasures is what I was the day I got married, and this man would have been the same. And I suppose I just had a conversation with regards to uping his activity, and making sure that he was eaten properly to replace that. That we didn't want them losing any weight prior to surgery. And that make sure that whenever you're taking part in particulary the circuits class, that whenever you're going home, you're having something you're go and treat yourself, and have a wee scone and have something that's a bit more high in calories just given the activity that you've done?

SB 23:41

No thats grand, you have mentioned significant barriers as a result of COVID-19 and service provision of the prehab programme. Was there any any other barriers you haven't mentioned in relation to that, but or was there any benefits you find was COVID-19 the way you're talking I'm not I'm not I'm not thinking that that's the case. But maybe there was one...

P12 24:04

No, no, not for this particular programme. Look, not for anybody I would say, you know, there, I can see benefits to families and to some people of routine, it slowed everything down it give people back time. Because you're there's nothing on and stuff. However, for the prehab or the rehab side of the house. No, it was it was awful. You know, even the rehab people you know you had people what you realised very quickly was you know, maybe I had people come and see me in a walking group come and see me in a class, what you realise very quickly was they see me more than the seeing their family so you see whenever those classes were taken away, I spent two years ringing people on a daily basis to check in and see how they were and that they have access to you know, getting to the shops and doing all that kind of stuff and maybe referring them on to other we attempted during COVID during the lockdowns, you know, council had services in place to.... so I was maybe linking those people up with other services, because actually the ones weren't going out and they weren't able to go here and go there. They weren't able to do their shopping online. No, no, before I go on a rant.

SB 25:15

That's fine. And you had mentioned already, we're just thinking here about how patients engaged with the cancer prehab programme? And do you have mentioned already that and some struggled because of the time element? That was a barrier, like they had other things to sort out and do... what about whenever you initially contacted the patients, what was their understanding of what you were talking to them about? Did you find the and you said at the start there about it was kind of the whole thing where they're given their diagnosis and it's kind of white noise after that, did you find people did remember anything from their their initial equipment

P12 25:55

So if you were ringing people and they were maybe saying, you know, something like, "Oh, yes. Remember that found something like that?" And think it was very, I don't think it was particularly clear.

SB 26:08

Okay,

P12 26:08

Because I think in that initial Breaking Bad news, they were getting so much information. And, you know, they're getting this massive pack. They were going home and going "what about this? And what about that?" So a lot of people, you know, I would say maybe a quarter of them have an understanding of the prehab programme. And a prior understanding, I would suggest a prior understanding made them more likely to engage. So I mean, we did, we kept I mean, it was a pilot programme, we kept coming back, saying, right "The message of the breaking bad news appointment needs to be more clear" and the CNS's and the health care professionals all tried, they're did that. But But I suppose it really came down to, you know, the diagnosis that people were getting on how they were managing that information.

SB 26:55

Yeah, and that will vary from person to person. What did you think, supported patients to engage? Were there? Were there things? Like you had mentioned there, the patients that had longer time to surgery? Was that a facilitator?

P12 27:10

Absolutely. What you were able to do was get people in, you know, or because they had a longer time, and maybe weren't in that 10 days of isolation initially, because they had their weren't thinking, not next week, but the week after I'm going on for surgery. They were engaging with the programme, and they were coming in. I mean, I had great results with people, as I say, with people that had, I would say, more than four weeks. Because there were, you know, they were coming into me, they were having a zoom call with me or FaceTime with me, you know, once or twice a week, and coming in with me, and then having a FaceTime call, having me check in with them and that sort of thing. It was really the ones that were maybe only had two weeks whenever you were making contact with them, that I really then had to weigh up, what, what is this doing, what, what are the benefits to this person? If they are doing four sessions, or five sessions, they're telling me they can fit four sessions of exercise in two weeks, you know, that way?

SB 28:07

Yeah. Yeah. And did you find there were, like, if the family was encouraged? Would that would, you've seen sort of like that, as a facilitator? Did you experience that were families maybe encouraged the person to be involved?

P12 28:26

The thing is that with the rehab programmes with Move More, you know, back before it was it included prehab always sort of said that if you can be active, if you can bring somebody along, if you don't want to come yourself. I mean, it was breaking down barriers to increase engagement with the programme. So we were I supposed versed on how to do that. So, you know, maybe somebody's saying, "Oh, I don't drive. But my husband might be able to bring me in", "well Sure, he can come and take part in or he can do this, or he can do that." There was never, in the rehab side of the house, we have what's called a Buddy system, whereby a lot of people, you know, a lot, I mean, even even the prehabs were saying, oh I've never done any are there rehaps yeah prehabes are saying, " "I've never done any exercise before. Not sure I'd like that. I'll bring my husband along, or I'll do this or I'll do that." Do you know, so yes, is the answer to that.

That's fab. I didn't realise that. So partners or a friend could have come along also.

Yeah,

SB 29:31

yeah.

P12 29:32

Yeah. And that certainly as as time went on, and as restrictions left and and were people were coming in the, you know, in the centre, if they couldn't get in because they didn't drive or whatever, you know, we would have just said "Well, sure, bring your husband along, you know, if they want to come along and you know, come with you engage in exercise with you,"

SB 29:54

yeah, fab. What did you think of the level of support provided by the others stakeholders in the programme. So like the likes of the CNS, or the other people in the working group? How do you? What did you think of the support they provided to the Move More Coordinators?

P12

Yeah, in terms of support and that we had the right information and that if we needed to go back to them and ask a question, yeah, they were there. We had sort of fortnightly meetings so if you had of had, the thing was, we were kind of answering to our line managers in Council who didn’t have a great understanding of the programme, other than what we had told them. There was no engagement, prior to the conversation about us getting involved. So in terms of, if you got a difficult case or maybe you weren’t quite sure, I found those meetings were beneficial because you were able to say, “Look I got this referral last week, they didn’t want to take part, I’ve rang them twice or I got this” so you were able nearly to sort of say, “what do you think of this” “How could we approach this better?” and that sort of thing.

SB

That’s really good. And you had mentioned there being able to communicate with the CNS’s with regards to emotional support, if you had identified that a person needed more, so was that helpful, that direct link to the CNS’s?

P12

Yep absolutely.

SB

Were there any barriers to service delivery in terms of referrals, you have already mentioned in terms of delivery, not having that face to face and the assessments and aspects of that, but in terms of the referrals or follow up, have you found there were any barriers?

P12

To taking on referrals?

SB

Yeah, the referral system coming through to you, did that work well?

P12

So I suppose one of the big things was we all got, so there is 4 co-ordinators, sorry there was 3 prior to Claire coming on board, we all got the emails, we clicked in to the emails and said it is Zoe from Bangor, for example, right [MMC] this is yours, are you able to see this person, so we had have worked it between ourselves that if anyone was off on leave or off sick… Now there was a period in September when the functional assessments were back up and going, we were sort of just getting back up and going when [MMC] who has x kids, COVID hit her house, so we probably had 4 weeks were she wasn’t at work and then I got COVID and my x kids and husband got it, so I think I had 2 weeks were I wasn’t at work so a lot was on [MMC] and at that point the referrals would have been coming through quite regularly, and it was agreed that, for example, if I got a referral for a Bangor area, that I would ring them, I would ring them, I wouldn’t see them face to face as that was outside of my Council area, but I would ring them and offer them a Zoom and offer them support until such a time that their co-ordinator came back. So I suppose we worked it out ourselves. It was only going to be as good as what, you know, that’s the thing with anything that has a very small number of staff you, and the problem was with COVID, once you were out, you were out for 2 weeks at that point, so, yeah.

SB

So what learning have you gained from delivering the programme?

P12

That I don’t want to do it. [laughs] Only Joking

SB

Any positives? [laughs] No no, and that’s fair enough, and I think that’s a very important that you have learned and been able to move on.

P12

My learning from that, my learning, or I suppose my suggestion would be that we are exercise specialists working within Council and this is a very specific field, and whilst you have had training we had no support, so whenever I got the referrals through, like that very first one where I knew her for years and I knew her kids, they were the same age as me, and she was saying, “How do I tell my mum and dad [MMC name]?” I really struggled with that and there was nobody there to support me, you know that way. We had Zoom meetings every other Friday were you had 8, 6 or 8 people on there, but you didn’t really want to say at that point because, because it was a role that sat, Council were really our employers, but we were nearly answering to everybody, we were answering to Trust on the prehab programme, we were answering to Macmillan on the prehab and rehab and it was just overwhelming at I think at times in terms of the referrals, the complex referrals that came through. And because at that time Macmillan had downgraded, they did have, you know there was no member of staff that was dedicated to us and providing us with support at a time. I think, I think if I had to say something, there would be, if I was to make suggestions it would be that there was appropriate support in place for Move More Coordinators. And for Trust representatives not to rely on Council to do that, because there has been no great engagement of Council, with regards to what we need to do to for our own resilience.

SB

Yes, and that’s a very important point. Yes, and thank you for making that. Just lastly, I know you have an appointment at 10 o’clock here. The last part here thinking about any improvements to enhance the sustainability of the programme that you can see from your experience? So that’s that’s definitely one going forward, but is there anything else you want to say before finishing?

P12

Eh, no, I suppose probably the the house calls is probably one that really seemed to make the difference, certainly when we were doing our own kind of evaluations, [MMC DOING HOUSE CALLS] seemed to get a better uptake whenever she was able to meet people face to face and build that rapport. And I think going forward, with the lifting of restrictions, there is the ability to do that, there is growing confidence, that even if participants are not able to do house calls, there is growing confidence that people will come into the centre to engage and I think that will make the difference, that face to face. And I think between that, greater communication between the stakeholders and council and support for coordinators, have an actually, instead of just relying on someone doing it, actually knowing who the person is that is supporting the co-ordinators, if you know what I mean.

SB

OK, yeah, yeah, no that’s very important. You had also said earlier that those patients were better off when their treatment window was 4-6 weeks down the road.

P12

Absolutely, 100%.

SB

Would you prefer that that was part of the criteria?

P12

Absolutely, I think going forward and opening this up to more cancer sites and having more referral coming through on a monthly basis. We need to look at what realistically, what are our expectations of these participants because, while we are sitting here making decisions for them and saying that they have to take part of this activity, they have to see us or make time for us, even a quick call two to three times per week, in real terms people are sorting out their affairs, you have 70 year old people who are going to have lung surgery or bowel surgery and you are telling them to take part in physical activity, I think if there was more, like a 4 week plus, would be good for this particular type of programme for the community based, you know for them being signposted to us as opposed to the other ones. Maybe there is room for that in the other streams or the other bits but for us, I think, for us I had really good results 4 weeks plus. Good case studies as well, but anything under that and you are really eating into peoples time for sorting their affairs, you know that way.

SB

Yeah, in terms of data collection, the timepoints, what are your views on the timepoints for collecting data?

P12

Well the timepoints for collecting data in the initial stages of the programme was really just sit to stand, get your kitchen chair out and tell me how many times you can do that, so it took a minute or two and it was fine. Once then you built in the functional assessments, by the time they came in to the centre and you did all you needed to do, it was looking more like half an hour, which was fine if they had 4 weeks to treatment and you were repeating that 4 weeks later or 6 weeks later, but whenever you were doing it on a Thursday and they were maybe going in for treatment 2 weeks later, you were doing it again the following Tuesday because maybe that was all they could do, so you were 10 days, so I think we have to be realistic about what that data collection is going to show.

SB

Yeah. Did you find that difficult to get that squeezed in?

P12

Yeah I did. I suppose you had that its important, it’s a pilot programme, but I suppose what is more important, is it seeing people for exercise, or is it seeing people to get them to do Grip tests and so I think going forward you won’t have that issue because it has been a pilot programme but there will be some sort of data collection, but I think if it was more user and participant friendly I think that would definitely make a difference.

SB

Yeah. No that’s grand, I should have asked that question earlier in the interview. I have no more questions, is there anything else you would like to say.

P12

No. That was good timing, 9.59.

SB

I will stop the recording then.

# P13

**SB**

Okay, so emm P13, just before we start talking about the programme itself, what did you know about Cancer prehabilitation before it started at the South Eastern Trust?

**P13**

Well as I don't work in cancer services, specifically, in terms of actual cancer prehab, not not very much at all. My involvement was mostly with pulmonary rehabilitation within the respiratory world. So, obviously, as a physio you understand the terms, you know, rehabilitation, and want to have the patients as well as they can be for what they're about to go through. But in terms of the actual specifics of cancer prehab, we hadn't been involved at all prior to that,

**SB**

And was prehab something that was done in your other area of work, in the pulmonary realm

**P13**

No not prehab as in preparation for treatment or surgery, rehabilitation in the respiratory world was more about teaching patients how to manage their long term condition and live with it

**SB**

Okay. Okay, so what was your role in the cancer prehabilitation programme?

**P13**

Well, away back at the start with the three tumour sites, I got involved, because I was the respiratory strategic leader at that stage. So in terms of the lung cancer, emm my involvement back then was with [NAME] the lung cancer nurse and [NAME] the consultant we pulled together as sort of a working group looking at that particular site. And what we wanted out of prehab, what we thought prehab might look like, and what sort of different areas that we thought needs to be part of prehab and really sort of looking at what we thought from a long point of view and respiratory point of view, and try and work up sort of a sort of a an operating procedure of how that might look. And we moved on quite quickly. And were sort of almost ready to go into pilot and and try it when COVID struck. So that kind of put pay to everything. And then as we managed to pick things up later on, then [CNS HNC] and her working over sort of over group steering group pulled everybody together, the three tumour sites, and we then sort of slightly different route in that we grew this, you know, generic prehab, which included all three tumour sites, and I then as opposed to being respiratory, I then stepped in, I was kind of representing AHPS on that group.

**SB**

Okay. Very good. So you were very much involved in setting up? And what about in terms of developing the assessments were you involved in that

**P13**

Well I suppose the working, as a group, we always discuss what assessment tools we might use, what outcome measures we might want to look at, I suppose I was more involved in bringing back from the likes of of the AHPS, you know, what we have experience of and what we can train the move more coordinators to do at the, you know, for the non specialist pathways. So certainly having a look at what assessment tools are available, what outcome measures we wanted to use and building those into the operating procedure, and then the locating them and finding from mostly physios, to be honest, the finding staff to help train coordinators for those things that they might want to have a bit more support with.

**SB**

Okay. And did you receive in sort of the process of this? Did you receive any training? Were you involved in any sort of meetings even with like the Manchester model people at all

**P13**

Away back, away back? Yeah. We did have a presentation on that. And we know that the group shared information and shared, you know, anything of relevance with each with each other. But yes, no way back the beginning, we had a very comprehensive sort of presentation of the Manchester model.

**SB**

Was it delivered by the Manchester people?

**P13**

No, no, no, no, just I wouldn't have been involved in that. Yeah.

**SB**

Okay. Okay. And what is your what's your sort of thinking around and thinking about the assessment referral process? When do you think is the best time to introduce cancer prehabilitation and provide the patient with the information pack?

**P13**

Oh, as early as possible, to be honest. I think if the patient gets they do get a lot of information at diagnosis and sometimes it's, you know, it's been a big blow and they maybe aren't able to cope with that and I think the clinical nurse specialists at that time breaking bad news appointment are so far the best positioned people in order to make a judgement call on what how much, or how little the patient can actually deal with on that particular day. But as early as possible, any kind of rehabilitation, the earlier you can get started then that, you know, you expect better outcomes.

**SB**

Yes, yeah. Did you encounter any challenges with the screening referral and consent process?

**P13**

Well, I wasn't now I wasn't directly involved with it I was just kind of facilitating the AHPS. But just on sort of feedback, the the it's everything's time consuming when conditions have very limited time with that and I suppose if somebody's had a bad diagnosis, and then you're trying to do all this screening that might be necessary to get them into prehab. It's, it's, it maybe isn't the best time for some people. So that is, that's I'm sure that was an issue for some of the the nurses. And I think the biggest issue, the biggest thing I could sort of think come out of the maintenance was that sometimes the patients, you know, they get their diagnosis, they get screened, they're referred for prehab. And the guys are making contact with them reasonably quickly. And then all of a sudden treatment starts the following week, or a few days later, or 10 days, and there's been no time for that. So that's kind of a problem in that, and that's good for the patient in that they're getting started. And the time from diagnosis to starting their treatment or whatever is short. But that doesn't allow for the prehab.

**SB**

Yes, yeah.

**P13**

So. So that's, that is that's an I think that's, it's an issue we have, but it's an issue you like to have, because you didn't want them waiting for ages to start the treatment. From diagnosis.

**SB**

Yeah... I don't know. P13, but is there for some patients? Is it better that they waited in the respect that they would be more ready for their surgery? Are there advantages to waiting for some patients?

**P13**

Oh, well, I, I don't think I'd be qualified to answer that I can't imagine I think even just from an emotional well being patients get a diagnosis of cancer, they just want to be on a treatment pathway. Yeah, certainly I know I would probably feel like that

**SB**

Yeah, no, no, yeah. Were there.... just I know because you're not doing these yourself. But emm getting feedback from your AHPs, was there any helpful aspects to the screening referral and consent process that they reported?

**P13**

To be honest, actually, most of the the AHPs are going to be involved in specialist pathways. So for example, in the specialist exercise pathway, I think in the whole time, we had very, very few referrals, most of them were able to go out to the move more coordinators. So we actually, I think, one or two and certainly no problems reported back to me that again, it's just time consuming, and maybe doing a wee bit some something slightly different to what you would normally be doing. So it just takes up that bit more time, but no real issues at all. And as I say our referrals to the the specialist exercise pathway, were very, very small.

**SB**

Okay. And was that less than expected?

**P13**

Yeah, I think into the pathway, yes. Most of them are able to go out to move more coordinators, which is great, because that leaves capacity within the professional services then to deliver for the complex patients. And I think I think from listening to the nurses, and the lung cancer nurses in particular is that the patients are presenting much later, whether it's the effect of COVID, whatever. So they are very frail, and some of them just aren't suitable, you know, for the pathways that the AHPS would be offering.

**SB**

And do you think that's COVID related that they're presenting later?

**P13**

I'm not not sure. It certainly appears it certainly was certainly would have had an impact. There's probably all sorts of factors that just need researched and need looked at but I'm sure it has an impact. I'm sure it has had an impact.

**SB**

What impact did you think COVID had on the programme itself?

**P13**

Well, I think we would have been much further down the line, certainly. You know, from a lung point of view, we were kind of ready to give it a go. And from that stage, we were looking at, you know, I was involved in the lung cancer. So it was the it was that sort of tumour site that we were looking at, and we were kind of ready to go, that paused everything. But actually, I think the effects of that pause meant that we all came together and then this standard operating procedure, you know, including the move more coordinators and all that collaborative working with health and wellbeing and and [HEALTH DEVELOPMENT MANAGER] and the councils has been a very positive, very, very positive aspect of working on this group. that has been hugely beneficial to the patient and to the to getting this up and running. So I think for us to have gone ahead and gone on our own. It might have worked, it might not, but it would have been much more difficult. This is a much, much better model.

**SB**

Right. interesting

**P13**

So COVID probably bought us that time if you know, yeah,

**SB**

Yeah. So So was this was lung prehab was that a sort of development on it's own, was that going to go ahead on its own, then outside of the others or

**P13**

Well no we were all sort of working towards, we were all we were all sort of working towards prehab, but there was models up in Antrim Hospital that myself, and the nurse had gone to see which was directed solely at the lung cancer site. So we had gone up to see to see that. And then we had looked at that, and as a group of consultants and the AHPs and [NAME] the nurse, and we've kind of looked about and what we actually see that we would like for lung cancer patients, and tried to look at our own model, but it was very much we were focusing on the lung cancer. And we had come up with the model. So I think probably because we had we had there was another model out there that had been operating in the Antrim Hospital that we got just got were further ahead. So we were just about ready to start. And we probably would have got started and and and then pulled back in again later later on. So I think that's probably where we were, it's not that we were going off on our own, I think it's more that we were just a bit further down the pathway.

**SB**

Yeah. What benefits have you experienced in terms of your professional development and practice as a consequence of being involved in the programme?

**P13**

Well, I think one of things would have been my work as I say, it's not cancer focused. So I was meeting other professionals that I've never ever had, you know, been dealing with and you know, [CNS HNC] and the nurse specialists and involvement with health development in a different way. But also with the councils and MacMillan, I would never have had that in my other my other job, so that I just felt it was fabulous, the way everybody worked together. And, you know, we were in a huge catchment area there. And we had the MacMillan coordinators, you know, Newry and Mourne, right the way around, I just felt that the the working together and the troubleshooting and the problem solving was was great. It was it was a lovely working group to be part of so it was yeah.

**SB**

Yeah. So you would have been you regularly attend, do you still regularly attend the working group meetings is that ongoing?

**P13**

As I say, I've been seconded now into a different role. So I keep my eye on it. And I do I have been at the last few meetings that [HEALTH DEVELOPMENT MANAGER] and [HEALTH DEVELOPMENT LEAD] run. But there's a new AHP for hospital services, so it's probably sits better with her and I'm back now as physio lead, so I'm no longer AHP world if you like so.

**SB**

Oh right ok

**P13**

So, [PHYSIO NAME] is there, I was there as the respiratory strategic lead to start with, and then that kind of merged into the AHP representative. So there is somebody else, and sort of the cancer region stuff has all been passed to her. I keep my foot in just to keep an eye on what's going on. Because it is interim, everything's interim. So

**SB**

Yeah. And have you enjoyed being part of the programme?

**P13**

Oh, absolutely. Yeah, as I say, working, meeting new people to work with and, and just learning. I mean, just having that sort of growth mindset, we want to learn those learning about an area which I really knew very, very little about, and the potential out there and what they're trying to achieve. Yeah.

**SB**

What improvements do you think would enhance the sustainability of the prehab programme going forward?

**P13**

Sustainability, resource, you know, it was a fund it wasn't a funded pilot, it was a pilot that people did and you know, having to go and ask for me the AHPS that would you take the so on, there's no extra money. There's no extra staff. There's no nothing, no time and a very busy workforce already. We were we were worried that we would be overwhelmed with referrals, but actually it didn't. And that was good. And I'm sure [CNS HNC] would would agree that you know, within the One Stop clinic and speech and language there are big gaps, which we struggle to, to fill. Yeah. So certainly, resourced just a resourced prehab programme with with specialist staff, you know, doing those roles and getting a real grip on prehab. And rolling that into post post treatment, you know what you want is you want to see that carryover, you want to see that the patients, if they engage in physical activity, if they engage in smoking cessation, if they engage in all of those, those sort of strands or workstreams that are offered that post-treatment or post-surgery or whatever, that they're still engaged, and that they're still keeping going and having the having the the ability to be able to provide all of that.

**SB**

Yeah. Yeah, that wraparound service and having the resource. thinking about if this was rolling out to other trusts, what do you think would help to keep in mind for going forward for other trusts?

**P13**

I think, thinking outside of the trust, you know, that it doesn't all have to be delivered in the trust that we do use the voluntary sector, we do use the MacMillan coordinators. And I know there is quite a wee bit of prehab goes on in the Belfast trust, but I think a lot of it is delivered in pockets within trust. And that's probably difficult. And there's probably quite a wee bit of the of the work is delivered very, very well by our Move More Coordinators, and leaving capacity, you know, for those more complex patients and more that require more complex prehab or post hab. So I think it's making, you know, think outside of the box and outside of the trust as well. Yeah.

**SB**

Can you think of any barriers to the long-term delivery of the cancer prehabilitation programme? I know you've mentioned there resource being an important one for going forward.

**P13**

I suppose resource with us, but also capacity with the Move More Coordinators, and they are, you know, funded on a I presume they're funded on time periods, and then they're refunded and refunded. So it's the capacity of them to take this on emm barriers.... I suppose one of the biggest difficulties is that it's not a barrier, it's the it's the time to be able to deliver prehab between diagnosis and treatment and, and having that long enough to make a difference. You know, that's that, that is that's difficult. But I, you know, I personally feel that if you can get the patient engaged, and even if you're not actively having a prehab session, if you can't, if you haven't even got time to do that, but if you're engaging with the patient, letting them know what services are there, even if it's only a bit of advice over the phone, that hopefully the outcome will be that they'll re engage with you when their treatment's over.

**SB**

Yes, yeah. So it's starting that relationship early and making the most of it yeah. Yeah. No. Something else you said there that long term delivery that just, yes, you touched on earlier, P13, about the goodwill that you had, whenever you went to start, why would you get involved, there's no money, there's no nothing. But there was goodwill. And I think that's very interesting thing coming out of this project, that there seems to have been a lot of that. And that will be from, you know, relationships that you've had with your staff and, and right across the board. In terms of aspects of the project. I think that's been that has come out and there's been a lot of in the South Eastern Trust.

**P13**

Yeah, and I think that's, I think that's not just in prehab. I think that's a South East Trust thing. Let's give this a go. See if it works, and if it works, we know it works, we'll have the evidence and we can put the evidence forward to try and, you know, build resource down the line, but it's easier to get resource if you can show that something works. And I suppose that's where we come from, you know?

**SB**

Yeah, no, I think it's incredible what's been achieved. Have you any final comments that you want to make anything that I haven't asked?

**P13**

No, I don't I don't think so. It has for me, personally, it has been very positive and it was that collaborative working you know, you do see it but to see it working so well, for directly for the patients was great. Yeah.

**SB**

I'll just stop the recording then

# P14

**SB**

And so just to start off with what did you know about cancer prehabilitation before the service started in the South Eastern Trust?

**P14**

I knew there were there was prehab happening in England, mainly over in Christy in Manchester I knew a lot of what was happening over there. I knew the northern trusts also have a prehabilitation for lung patients as well. So I just I really just knew that it was to optimise somebody's wellbeing prior to commencement of treatment. So that was mainly about it really

**SB**

Okay, and what was your thinking about it as an intervention

**P14**

So lung patients at the very start of this, I really was struggling to see where it fitted with the lung patients, emm certainly I'm still struggling to see where it fits with the palliative lung patients, but I definitely can see a role for it with the curative intent patients. but you know, we do, we do offer sort of a lot of what prehabilitation offers, but more as a supportive care for our palliative patients. haven't really been able to refer many of our patients that are palliative intent at all. It's been mainly surgical resectable patients, and sometimes they're almost too good for prehabilitation. Sometimes we think we're probably at their optimal fitness levels, but I've managed to get a few referred through but yeah, very small numbers for the lung patients.

**SB**

Okay, and do you, what is your sort of thoughts on engaging patients before surgery do

**P14**

My thoughts in referring them? I have no problem engaging them before surgery, it's just trying to to ensure that they're well enough to go through prehabilitation, a lot of them are quite deconditioned, which are probably the ideal patients for that to try and optimise them. And some patients are just not well enough to go through anything, which is why they're more palliative intent. But but as I said, you know, we do already have those supportive services in place, which offer quite a lot of rehabilitation does. So that sort of supporting the patients from a mobility perspective and functional capacity perspective, and just managing symptoms as well, as well as their breathlessness and smoking cessation, and nutritional support. We offer a lot of psychological support for our patients as well. so the main aim that I could see from the prehabilitation was to optimise functional capacity. And, you know, yes, I mean, engaging patients about that we can we do certainly try and do that. Not all all our patients are suitable though. And usually, I would have a discussion with the consultant prior to enrolling them in the prehabilitation programme. But we don't see a lot of the surgical patients we sometimes don't even meet they're discussed MDM, and then they go straight over to the Royal so we don't actually get to meet them. So we can't really engage them. There have been a couple that I've discussed with over the phone and referred them on that basis. But it's I think it's better to get a face to face with the person so that you get a feel of who they are and what what they're likely to be able to achieve. Okay.

**SB**

Now, just before we move on to the assessment referral process, we'll come to that. what is your role been in the cancer prehabilitation programme? And then from conception right through what what sort of roles have you been involved in

**P14*8

My my role is to recognise somebody that would be suitable to engage in a prehab programme and discussing it with them and doing their screening beforehand and then referring them onwards to the prehabilitation programme, and sort of recognising are they on the universal the targeted or specialist pathways?

**SB**

Ok, and were you P14. Were you involved in the planning of this prehabilitation programme?

**P14**

Yeah. Oh, yeah. The implementation of the pilot? Yes.

**SB**

And and did you in terms of the assessments that were chosen, were you involved in selecting those?

**P14**

Emmm *inaudible* I'm involved in those. No. But, I mean, obviously, it was a continuous discussion throughout. So you know, I think that was basically being discussed between the three tumour sites, what are the best sort of prehabilitation measures and looking at the evidence base and from other trusts and other areas and regionally what's happening so I think that was that was the main sort of deciding point on that.

**SB**

Did you receive any training as part of this programme to roll it out?

**P14**

There was no training really required. In the South Eastern Trust we always did it at ward level you know whenever we're working on the ward so there wasn't much training involved with that. And the only thing that I had no experience of was the Audit C for the substance misuse. But it was fairly straightforward when you read about it. So

**SB**

On reflection, is there any way you could have been better prepared for your role? Or do you feel that you were okay?

**P14**

I think I was ok, I felt well prepared enough. The only thing I would say is the lack of time, from my perspective, it's not that it's a completely timely process. It's just sometimes you're seeing a patient at Red Flag clinic, they're given bad news with regards to diagnosis, and you've got all of the supportive stuff to go through in terms of your role as a lung cancer nurse, and then you then bring in the role of the prehab. So sometimes, not only is it a wee bit overwhelming for the patient, but then, you know, I might have about two or three other referrals do as well as the prehabilitation referral. So there's, it is quite timely whenever you meet a patient for the first time, and just trying to find that time can be quite difficult to take.

**SB**

Yep. And is that the best time to introduce the prehab to the patient? That initial appointment?

**P14

Yeah. I think there was no other real time that I could have done it, because if they were coming up for results, and they were for surgery, then I wasn't gonna be seeing them again, basically, because they'd be going over to the Royal, where they would be pre assessed as well by the the nurse specialists who were there prior to surgery. So I don't know if they implement some kind of prehab as well over in the Belfast Trust, but I know they're certainly looking at trying to implement prehabilitation over there at the moment. But, yeah, I think that that's really the only time that I can see them. And I mean, we do see a lot of patients a lot along their pathway and lung patients see patients from diagnosis right through to end of life. And so it's usually our palliative patients that we would see frequently, we tend to not see the surgical patients, which are ones that are more likely to go through prehabilitation. And even our chemo rad patients all go via centrally to the the City hospital, so we tend not to see a lot of them and the lung cancer team over there would take them over. So the palliative patients are the main patients that we would see in the south eastern trust

**SB**

So that that first appointment, then is critical that results appointment is the time to introduce it. And just thinking about any challenges you've experienced with the screening, referral consent process then timing and time, lack of time. That's something you've touched on already. Is there any other challenges you faced with that process?

**P14**

Yes there is actually one of them I didn't get any feedback about it actually I put through somebody on the audit see, who was at risk of alcohol withdrawal, and I did get an email back from the substance misuse team to say that they don't, they wouldn't follow it up, because the patient has been transferred to another trust. So then I don't know what happened to that patient. So in terms of the alcohol withdrawal side of things, so maybe something that that needed to be addressed I did sort of I did email [CNS HNC] and [HEALTH DEVELOPMENT MANAGER] at the time about it, but I never got any feedback about it so I'm not too sure what happened.

**SB**

Okay, that's, that's that's good to note, then just about follow up communication, then whenever somebody's moving outside the trust. Yep. Yeah. And what about the screening tools? Do you feel they're all necessary? were any of them difficult to implement? I know, you said the audit see was a new one. But you thought it was quite simple

**P14**

No it was quite straightforward, very simple process and a very simple referral form. So it's all drop down menus and tick boxes, and with a wee bit of additional information, so there's no problems with that at all.

**SB**

How interested do you find the patients were to engage in the prehab programme.

**P14**

Sometimes I would, I would say, some of them very interested. And once you say, you know, we're going to try and get you fitter for treatment, it's really, really important as part of your treatment pathway to make sure that your lungs are functioning well, and to make sure that you're as well as you possibly can be. And others, I just got the impression that I'm just saying yes for the sake of saying yes, because you're given me so much information. I think it was information overload more than anything, but there have been some very, very engaged patients I know that for sure. And that, you know, a lot of patients that have done very well on it so but likewise, I do I do feel like I'm bombarding them with an awful lot of information at that one appointment.

**SB**

Yeah, yeah. No, I can imagine and what sort of impact do you think COVID-19 has had on service provision for the prehab programme?

**P14**

And well, I think the move more coordinators have been superb, and they've been doing the zoom, and trying to keep the prehab going as best as possible, and I imagine it's had some impact on it. But I'm not too sure, really, you know what in terms of the move more I know they're now moving back into leisure centres and things like that and trying to engage patients in their homes a wee bit more, which is fantastic. but I would say there has been some impact, but I I don't know exactly what that is.

**SB**

Do you think in terms of your role do you think there's been an impact on the programme? Do you feel COVID has had any impact on your, your role within the programme

**P14**

Within the programme? I suppose the main patients that I've been seeing It's, well, obviously, there was a reduction in referrals during the COVID pandemic, and there was a reduction in patients coming forward with lung cancer symptoms per se, as a whole. And so therefore, you know, we weren't seeing as many patients, so there probably wasn't as many referrals coming through. so I would say that would be the main impact.

**SB**

What benefits do you think you've experienced in terms of your professional development and practice as a consequence of being involved in the programme?

**P14**

*inaudible* before treatment, and and you know, the benefits of that, and maybe just to see a wee bit of feedback would be great just to see how patients have engaged in how well they've done pre and postoperatively. Because I don't think we've had an awful lot of feedback so far. And so it would be really, really good to see that.

**SB**

And hopefully, I'll be moving on to entering in some patients and relatives as part of this. And hopefully, that feedback will be will be coming.

**P14**

That would be brilliant. Yeah, that'd be really good to hear.

**SB**

And in terms of your were you involved in the working group,

**P14**

Yes. Yes.

**SB**

In terms of that, was there any benefits to your professional development from that?

**P14**

I don't think in terms of my professional development, there was no additional training. And I suppose just really the the mindfulness of knowing that, that we're going through this process for for the good of the patients, but no, I don't think there was anything specifically that improved my professional practice as a result of it.

**SB**

Okay. And have you enjoyed being a part of the programme?

**P14**

Yes, I have. But I think as I said earlier, I just find it really, really hard to see where prehab fits. And I think that's really been helpful for me to recognise where exactly, my patients fit into the prehab programme and I think it's really, it would be, it'd be very interesting to see if any of the palliative if there's anywhere that does sort of prehabilitation for patients going through palliative treatments. So obviously, with good performance statuses, but I think I've been burned once I think I referred one palliative patient, and unfortunately, she didn't actually make it to treatment and she died before prehabilitation could, I think maybe the Move More Coordinators had had made contact with her, but I don't think they actually got as far, which is really a testament to the type of patients that we see they can become very sick very quickly. So it is very difficult to refer those palliative patients on. So it is still curative intent patients, the surgical resectable and the chemo radiation patients. And it's funny we last week, there was a girl, [NAME] from one of the one drug companies did a talk and she's sort of introducing, very much advocating for prehabilitation prior to adjuvants or immunotherapies. And that's all for the chemo radiation patients over in Belfast Trust. So I'll be looking out for those types of patients as well. So

**SB**

Yeah, very good. Okay. And what improvements do you think would enhance the sustainability of the prehab programme? I'm thinking South Eastern Trust, but also if it was being rolled out wider?

**P14**

Being rolled out wider, you probably would would need more people on board, I would say and to be able to refer and just, you know, because it can be quite timely as well. I think the other thing is is would there be scope to it, it's very difficult to bring everything into one appointment for the patient. So maybe need to think about another appointment to discuss that separately, but I wouldn't want to necessarily bring them up to hospital for an appointment just to discuss prehabilitation. So you know I would sort of tie in with another appointment with their consultant or something like that. sustainability goals is going to be the same as everything cost and all that kind of stuff as well, if they're not there you can't sustain the service

**SB**

And what do you think in terms of barriers for the long-term delivery, what do you see as the key barriers?

**P14**

Well, from my perspective to time, it's just the time, the types of patients that you have as well and how sick they can become quite quickly so those would be the main barriers I would say

**SB**

Thank you, P14. I don't have any more questions. Is there anything else you would like to say about the programme or any issues you'd like to raise that I haven't asked about?

**P14**

No I don't think so. I mean, as I said, you know, it has sort of made me a lot more mindful of the types of patients that go forward for the prehabilitation and I can definitely see a role for for those types of patients. And maybe thinking a bit more about our palliative patient population. And do we sort of do the prehabilitation more tailored to their kind of needs? I don't know. I don't know. But as I said, we do have supportive services that supports them anyway, prior to treatment

**SB**

You raised a lot of interesting threads. It's looking across the tumour groups, it's like the challenges within the tumour groups. It's quite interesting just to hear those and it would be good to document those and share that learning moving forward. So thank you I'm just going to end the recording here.

# P15

**SB**

What Did You Know About cancer prehabilitation before the service started at South Eastern Trust?

**P15**

Oh, we've seen about it before in the British Thoracic Oncology Group, it's been reported from the Manchester group. So we would have known specifically from that involvement within lung cancer, but not really out with the other. On the other tumour sites, we've been focused mostly on the lung cancer side of things.

**SB**

Okay, and what's your view of engaging patients in the prehabilitation stage?

**P15**

The difficult thing about prehabilitation and different tumour sites is it depends on the, I suppose the suitability of the patient and the tumour that you're looking after, we would unfortunately be in a case where a very small number of our patients would move forward for radical treatments, and a lot of them would present with existing comorbidities. So prehabilitation has classically been used towards people moving towards surgery, or trying to make them you know, ready for an anaesthetic or something that has something radical to it. Whereas I think there's a lot of things starting to move now that people can be made better for things like systemic cancer treatments as well. But the engagement on it can be very difficult, because if your, your average age is somewhere between, you know, 65 and 75. And you have pre-existing lung conditions, many people will have been offered prehabilitation before or rehabilitation with COPD. And they will make the association that they have with the rehab and the prehab will run the same kind of things. Plus, you've also given them a diagnosis that is likely to be terminal. And therefore the engagement can vary for numerous reasons, whether it's comorbidities, how they take the news and how ready they are to move. Also, some of the treatments, particularly in lung cancer have to be given within two weeks for small cell, for example, and you have very little opportunity to do that prehab it's almost ongoing rehab rather than prehab.

**SB**

Okay, so that makes it all very challenging with your tumour site. Yep. What has your role been in the cancer rehabilitation programme and thinking from it started right through to now, what sort of roles have you had in the development.

**P15**

So I'm the I'm the lead for lung cancer and I would have my red flag clinic. So certainly, through my clinic, I try to identify ones with our lung cancer nurse specialist that we think are not at their optimum shape, and could potentially improve prior to any particular treatment, or we think it may benefit them in the, in the in the longer term. we've been probably quite focused into ones that we think are pretty sure are first of all, engaging, and second of all, would benefit from it, there's probably a lot of people we've screened out just on the basis that they're just not well enough you know, from that perspective. I try to get it through some of my colleagues, but the problem with lung cancers is sometimes a third of them will present to hospital, you know, with advanced disease. So you're you can't really use prehab in those situations. And they will present other tumour sites as well. So there's sometimes a little bit of a convoluted diagnostic pathway. And so my role really is to identify in the red flag clinic, I've tried to put it through the MDM as well. And [LUNG CNS], who has been our link, obviously, here in the South Eastern Trust, would be there and she would identify patients that say, for example, would that patient benefit from prehab, and we'd be quite selective as to who we can then offer it to.

**SB**

Okay. And have you been involved in the working group or in the sort of early days, the development of it in the South Eastern Trust?

**P15**

Yes. A couple of other tumour sites that were there, so like head neck and the GI surgeons as well, so

**SB**

Okay. And in terms of that role, what would you have been? What was your sort of input at that stage?

**P15**

I think probably the most important thing was to say that prehab’s a good thing, but it's not necessarily always a one size fits all approach, we sort of find that there were different pressures, for example, the surgical teams are very much all about optimising someone before a surgeon sees them. Whereas on our site, we were saying, well hold on a minute, what about more by people who are going through systemic or radiotherapy treatment options, which is where we would see more of our patients going, so if you make the whole focus of a prehab, prior towards a surgical diagnosis, or a surgical procedure then the the focus can be very different, particularly, for example, in the gastrointestinal side of things where dieticians can be very important to the head and neck side of things where swallows can be quite important. In lung cancer, it's really, it's really looking at the whole the whole picture, you know, of the whole individual, which is what prehab should be, but there are definitely a lot of focuses in certain tumour sites that are higher than others. I think that was just to try and sort of, you know, highlight that from our different sites. That's where the benefits would be.

**SB**

Do you feel as the programme developed that you were able to tailor that and you had a voice that that you're able to shape that as to what you wanted to see?

**P15**

I think so. I think certain ones will always shout louder depending on what they can get and if we're not there with regards to seeing the same kind of benefits there then sometimes one site can shoot off a wee bit more than the other. It's, it's tricky, because you're always against the time pressure, you're always against the diagnostic pressure, and some things, for example, take a wee bit longer, and it's whether you start prehab earlier in the diagnostic journey. So for example, if you had head and neck patient, you can see something, you can biopsy it here, or you've got a bowel thing, you can get it with one single test, we sometimes need multiple tests, which can happen over several weeks. And the multiple tests lead on to, for example, a pneumothorax means someone can't do any further physiotherapy, they have to, and sometimes they're hospitalised so some of the risks are higher. So I think, no, I think we it was good to have everybody there. But it's, it almost felt like it was being pushed a wee bit more towards pre surgery than pre cancer treatment, that was just my opinion though.

**SB**

Yes, maybe it would definitely sounds like it would benefit from having more open sort of thinking around that. Okay. Given that there's a very short timeframe between diagnosis and surgery and treatment, when do you think is the best time to introduce this and it from talking with others, it seems it quite often happened that sort of delivering bad news appointment is not the best time. Is there an alternative time? Or what did what did you find?

**P15**

I don't think I don't think you have an alternative time, you have to give it as early as possible the and the only time you can offer prehab to a tumour is when you have a confirmed tumour. So that's the Breaking Bad News one. So what we tend to do, for example, as we'll take the hit and give them the bad news, and then we'll usually have one of our lung cancer nurse specialists take them off and do the holistic care. And as part of the holistic care, you get batch on prehab to that, so when for example, when they're talking about benefits and supportive care community side of things, they can also then say we can also offer you prehabilitation, before you consider any treatments. And that seems to work out quite well doing it that way. You don't just jump in with the bad news clinic and go, oh, by the way, you can do this as well. We batch it onto our holistic care, and that works very well.

**SB**

Okay, but that and that that happens all at the same appointment.

**P15**

**SB**

Okay. How have you found the patient's sort of engagement with the cancer prehab whenever you … you're not raising it, then is it that's the CNS is raising it.

**P15**

So we'll identify the people that we think can do it, we'll come into conjunction with them, whether we think they would be good candidates or whether they, you know, we don't think they're going to be able to cope with certain parts of it. Now, there's always good parts, for example, we have good links anyway within the holistic care that involves dietitians, and it involves, you know, financial support and other things. So whenever you've got the dietician and some of the physio support that's already there, some people could argue that's almost part of the prehab programme, it's just not specifically delivered. But there are some patients that really are just not well enough to go through the whole package, or they'll decline it to somebody because they've had so much bad news that they'll not say, yes, some of them will come back afterwards, we tend to focus it towards people who are, are motivated and are gonna engage with the programme, if you know what I mean. And when they do, they do well,

**SB**

Yes

**P15**

Stage four, stage four lung disease will usually, most people will die within weeks or less than three months without treatment. So obviously, with a good proportion of patients, it's not even worthwhile having that discussion in those situations.

**SB**

Okay. And what do you think? What impact do you think COVID-19 has had on the programme, from your point of view?

**P15**

I mean, like everything, it's, it's, it's really messed everything up. The only thing that I would say, COVID-19 has been good for is that it has identified opportunities for the virtual environment. So I know that obviously, part of prehab can be done through zoom calls and things like that. And that, again, benefit, a very good proportion of the population. And I think people engage better with that. The problem is, I think once you get above 65, or 70, we find that that virtual experience seems to tail off quite significantly, patients are not quite as keen to do it. They're either not as tech savvy, or they just like seeing people in those situations. So again, I think if you look at the tumour sites, you'll see different levels of engagement based on I suppose your population that you're looking after how well they will engage with technology, and then what they'll do with it. So again, I think it's we know what all the bad things are with a pandemic, and how it’s reduced that, the good side would be its offered, given classes that they can do on a zoom class. And if it's simply information and advice, and working somebody through something, I think it can work really well that way. And if that's the bit that probably is the positive.

**SB**

Yeah. And what about in terms of the development, the planning and the development stage, do you think did the virtual help bring more people around the table or not have happened anyway?

**P15**

I think sometimes it's quite hard we in respiratory obviously, we've been slaughtered by the COVID pandemic. So it's been hard for us to be engaged with them at times. I've certainly been to face to face meetings, and I've been on some of the Zoom meetings as well, again, the COVID pandemic has affected us more than we just can't be available for all the meetings that we would like to I know [LUNG CNS NAME]'s been able to get to some of them as well. But it's really, it's put additional pressure on our service that that probably be slightly different to the other services.

**SB**

And therefore did that virtual make it easier for you to be there or not really?

**P15**

Sometimes, there's times we've been so busy during the day, if you have a meet during the day no can't join into it because we're we're on the wards or doing other things. I find virtual meetings in the evening time sometimes have been easier just for me, just simply because I'm more likely to be able to attend them. But the day-to-day stuff that we've been doing has been just much more busy. And I think the tumour sites might have noticed less surgery during the pandemic, whereas we've had more inpatient involvement and with COVID stuff, so I would say probably not to me, but I'm sure other people have provided them with better opportunities. Yeah,

**SB**

Yeah. What benefits have you experienced in terms of your professional development and practice from being involved in the programme?

**P15**

I think the most important thing is that, we have to remember when we were dealing with cancer, we must always make sure we're looking after holistic care, and it's very easy to get sold by the pathology, what the stage is, and what their treatment is and just forget about that. And that's where the lung cancer nurses are in our group are brilliant. But you know, we can make people a bit better if you choose the right things. And we're not even whenever we look after people like COPD, we don't always use our resources just as well as we should do. So going through these kind of programmes, first of all, it reminds you that there's a big team looking after people. Second of all, that those teams are always willing to help. And, you know, if we actually all work together, we can improve certain patients cancer journeys significantly. And probably the most important thing is if we find the patients that really will benefit from it, and we use the resources well, I think that's what will make the greatest difference. But if we stretch them too thinly across a big long area, we’ll probably not make that same impact.

**SB**

Yeah, yeah. Have you enjoyed being part of the prehabilitation programme?

**P15**

Mostly, sometimes, sometimes whenever there's lots of meetings, it's, it's harder. But I think, again, I've worked closely with [LUNG CNS], who's been very good and she's kept us in the loop. And I think it's always nice to see what other people are doing as well, because you kind of forget when you're in one tumour site, it's all about your tumour site, but it's nice to see other people's ones as well. And then they can appreciate the differences and the struggles that we have between each other.

**SB**

Yeah. What improvements do you think would have enhanced sustainability off the cancer prehabilitation programme I’m thinking for the South Eastern Trust, but then also thinking if this has been rolled out across the other trusts?

**P15**

I think the question sometimes we ask ourselves, is prehab tumour site specific or is it cancer specific? Because if you think about cancer care, in general, it's a …. you have two ways to look at it, every patient is diagnosed with cancer is given a, a whole pile of leaflets, and they're part of that can contain very basic prehab advice, which could be you know, for everybody, which I think would be a really good thing. And then the thing that would make it better then is if we have tumour site specific priorities. So for example, if it's GI or nutrition, or you want people to be get a better walking distance, or going through surgery, and then you'd have to have some degree of collective requirements or standards that you would expect across the region so that we could all follow the same kind of thing, because what we're finding at the moment, I would imagine is, is this kind of little scattergun approach where one sites doing really well one other tumour site’s not using it quite as much for different reasons. And then another trust might not be using it at all. And it's like all things, you have to find out what works, audit that, show how it can make a difference and then spread that through start small and build up. And you may find other places like for Manchester and lung, they’re doing really quite, quite a big thing, you know, so, but they have a huge centre with huge resources, but we can't do huge things with small resources.

**SB**

And I think I… just listening to the other interviews as well it's incredible what has been achieved on goodwill without financial resources. It’s phenomenal. in terms of barriers for long term delivery of the cancer prehabilitation, what do you see these as?

**P15**

It'll just be it'll be the same things over everything in the NHS at the moment has been stretched to its limits, whereas people are being asked to do more for either the same amount or less, and we will only get improvement if you have a dedicated cancer team. In these situations you'll need specific resources allocated primarily to cancer and to prehab. In the same way that cardiac and respiratory services, for example, physiotherapists, they're starting to recruit people now into cardio respiratory, you know, there that will cover things like cardiac rehab, why can't we now start to recruit into cancer rehab? I think that's, you have to take those things on goodwill. you're doing it to try and do something better, and formulate that into a business case and a job plan across all of the all of the aspects of the prehab service, all of it.

**SB**

Yeah, that was good point. I don't have any more questions for you P15, is there anything I haven't asked that you want to share as part of this?

**P15**

No, I think you’ve asked all the appropriate questions, I think it is, I would just say sometimes it is hard for us to try and always get the right patients at the right time. And I think part part of that is due to our experience, part of that is due to resources. And part of it is I suppose, managing expectations as well. Because if we if we do put a pilot programme in place, you want to keep something going. But we always know that if a pilot programme starts and then stops, it's very hard to get it going again. So we've we've definitely done our best to try and look for ones we think there will be a definite improvement rather than trying to go spreading things too thin. And I think that's a good way to be. But hopefully, it'll keep going and hopefully it'll it'll build.

**SB**

Yeah, I think yours is quite unique in that it's it's such a short timeframe. But I think it's proven quite challenging for the move more coordinators and the likes of them to try and get the data collected and get the X. Yeah, it's quite *inaudible*

**P15**

You would have to do *inaudible* as well, you know, to try and get them engaged in their age group. There's wee things that are there, but those are all manageable, and as we come out of COVID. The other option is, you know, you could have people on site on certain days where they, you know, if they're close to we have two red flags on Thursday morning, could they maybe do something potentially, at the clinic to give them out things? Give them leaflets say do you want to come on Friday? Do you want to come on Monday? And I think having something set up with an offer rather than would you like to be part of this, have a read and a think about this. You can say we have a space, if you would like to come on Monday or we would like to contact you on this date. If I give a patient a date for a test, they go out of the clinic happy as Larry because they know they're getting something done. If they go out with uncertainty, they will always say no. So yeah, I would always say give somebody a very clear option. The chances are they will take it if you go with would you like to and maybe all that bad news is swirling around? And then they don't do it so that that would be the thing that would help a lot.

**SB**

That's a good point. Thank you, P15. I'm just going to stop the recording then.

# P16

**SB**

Super. P16, what did you know about Cancer prehabilitation before the service started at the South Eastern Trust?

**P16**

I had been to a regional meeting at Mossley Mill, maybe six months before that, or maybe a bit more when Macmillan launched the prehabilitation? So actually I’d quite a good background knowledge of it

**SB**

Good, good, and what were your thoughts about it.

**P16**

I think it's a really good idea because lots of our patients will certainly benefit from being that little bit more fit coming into major surgery because they've been living life, not expecting to have that big operation. Certainly, that should be addressed and can be addressed to their benefit.

**SB**

And now that we're a wee bit further down the line from the start of the project, what are your thoughts now on the prehabilitation programme has it been of benefit, do you think?

**P16**

I think obviously things are a bit difficult at the minute because we're not really doing any benign surgery, and we're just doing cancer surgery, people are only really getting about a week interval from being told they're having an operation to getting the operation. So there's a little you know, there's a little window and people contact them from move more and everything, but there's not really long enough time to make a difference. And I think to really get to show a difference, you need a bit of a longer time period. So I have used it, you know, for at least one of my patients who needed a bit more intervention and left, left him a good like 12 week period, to lose weight and to get on board with all the prehabilitation stuff, and that definitely had a good benefit

**SB**

Good, good. And what is your what has your role been in the cancer rehabilitation programme thinking right from the early sort of planning and through to now, what's your where has your involvement been?

**P16**

Emm well coming from the from being I suppose being the colorectal representative at that regional launch, then I moved into the group that was led initially by [AD CANCER SERVICES Name] and then by [CNS HNC] to talk about it so I was the colorectal surgery representative on the clinical representation along with [NAME] our nurse, and just trying to design our programme together and get it implemented.

**SB**

Very good. Do you feel like you were able to tailor that and shape that for your patients?

**P16**

Yeah, we're trying our best to Yes, there is a sort of generic which is suitable for everyone. And then we're trying to I suppose add in the more generic elements that will help our patients like pelvic floor exercises or, you know, more specific physio for our patients. And maybe you know, the more specific dietitian input for some some people. Yeah.

**SB**

When do you think is the best time to introduce the programme to patients? Or when have you been doing it and has it worked well for you.

**P16**

We've been doing it... So all our patients or our cancer patients come up to see a surgeon for a results appointment, whenever we're going to talk through, you know, what operation they need. And we've been introducing it at that appointment, which is a longer, you know, half an hour to an hour appointment with a colorectal nurse specialist. So usually we're talking about all of that clinical stuff and what the plan is. And then it's afterwards when the nurse specialist is talking to them. That works well for the people who have maybe come from a gastroenterologist or being told at the scope appointment that you have a cancer, but when sometimes very few, it's happened very few times, but sometimes people are coming up and just finding out they have cancer on that day. And that's not that's not ideal. So sometimes we'll leave it and bring it in a later date.

**SB**

And do you introduce the programme and then the CNS goes over the detail or is it the CNS?

**P16**

The CNS would take the lead on it, I think as it's part of their information giving, there's all that Macmillan information. And it's part of that, you know, because I'll explain the clinical side of the operation, and then the nurse will go through that kind of our enhanced recovery after surgery. But leading into that the prehabilitation goes with that. So it's a kind of a wraparound, before and after. Here's how you can make it work the best.

**SB**

Okay, okay. are you aware, from your point of view of any challenges with the screening referral consent process, emm it's maybe less appropriate for you if it's the CNS that's doing that, but…

**P16**

No I suppose the only difficulty is just the very short interval to surgery. So if I'm saying your operations next Tuesday, there's not much to be done there.

**SB**

Yeah. Are you aware of any resistance from patients about the programme?

**P16**

I would say most are quite well motivated. I suppose when they find out they need a big operation. They're quite well motivated. But yeah, some people don't engage but they won't engage with any intervention just some patients....but generally it’s very positive

**SB**

What impact do you think COVID has had on the programme from your point of view?

**P16**

Well, I suppose oh, everything has moved to zoom and telephone calls and they're not getting that face to face with their move more coordinator, physio or whoever, you know, the dietitian. So a lot of it can be done by telephone, but you just lose that face to face. And I suppose it has accelerated maybe moving to using those things where it is appropriate sometimes and maybe more a bit more written information being developed, and maybe something that they can access online, some sort of Portal or app or something, I think would be a good thing. If there wasn't covid, I suppose everyone would be turning up at the Leisure Centre, and maybe meeting you know you're losing that kind of maybe meeting other people in the same boat as you and the social effect of it and being together, and also that accountability of like, I have done, someone watched me and I had to do my exercise. And I had to weigh in that kind of way. It's more *inaudible*

**SB**

In terms of being involved in the planning and development during COVID time. Were there issues coming together or pushing the programme forward? Were there any barriers at that point? Or?

**P16**

I think just the conflicting demands in every direction, because everything had to be organised on a weekly basis. And so time yeah, it was it was difficult to make the meeting every time because things were changing it was like shifting sands constantly. So I would say from that point of view, yes, it was difficult to keep on top of it, because there's just so many, the normal, the normal kind of pattern of your practice was didn't exist. You had a lot of extra work. Yeah.

**SB**

Did zoom help that in terms of being able to go to meetings? Because you didn't have travel or not?

**P16**

No, because usually, if I couldn't go was because I was doing a different session.

**SB**

What benefits have you experienced in terms of your professional development practice being involved in the cancer prehab programme. thinking in terms of like knowledge, skills, working with other colleagues, maybe that you wouldn't have worked with before,

**P16**

I think it's been really good to develop those relationships with the leads from the other the head and neck cancers, the lung cancers and form a bit of a relationship there. And just being involved in pushing something forward and getting something set up. You know, all the troubleshooting involved. I think it's been good as well having been able to set up the adapt, adapt programme fellow to bring [ADEPT FELLOW NAME] in, because you could see there was a gap here that everyone's trying to fit this into, they're already doing their work, we need a person who can dedicate some time to this and making out things and gathering data and being able to, to look at everything for us. So I think it was good to be able to do that through NIMDTA. So I, you know applied for that and did the interviews and everything to get that.

**SB**

Very good. Very good. So [ADEPT FELLOW NAME], you feel that role has really been vital to the rollout of the programme in the South Eastern Trust

**P16**

Yes it’s made a big difference to me in the demands of my time certainly it remains to be seen how it all turns out, but hopefully for the best yeah

**SB**

Yeah. Yeah, that's interesting. I didn't realise that that's how that had all came about, and was *inaudible* so great.

**P16**

It was just a coincidence, because I got an email saying, Does anybody have an idea for an Adept Fellow? It's a leadership programme thing and I was like, I know, something that needs a Leadership Fellow. So it just worked out well

**SB**

Yeah. And that’s good learning for sharing for projects going forward

**P16**

Yes. And it seems that that was affected by COVID, because she should have started the year before and then it was delayed for a year. So that this was that did have an impact on holding us back from the implementation of the project.

**SB**

Yeah. Okay. Have you enjoyed being part of the programme?

**P16**

Yes, I would say so. Yeah.

**SB**

What improvements do you think could enhance the sustainability of the cancer prehab programme?

**P16**

I suppose it's the funding and the time required for all of the multidisciplinary team that is required, and more especially that kind of specific tailored things that certain you know, that our cancer patients and other cancer groups might be different, would require so I think if we could get funding for specific pelvic floor physiotherapy for rectal cancer patients, that would be really good. If we could get funding for like a preoperative clinic about sexual dysfunction, bladder dysfunction after radiotherapy and after pelvic surgery, that would be really good, but that's just a work in progress.

**SB**

Have you any sort of advice or learning that you want to share in terms of this programme being rolled out across other trusts?

**P16**

Apart from you just have to start doing it. Otherwise it'll never happen. And I suppose it's just considering what you're… what all cancer patients will benefit from and getting a generic programme and then having bolt-ons or add-ons for your specific patient group that that are going to make this, not everybody needs all aspects of the course rollout. You know, the smoking cessation was much more important for the other two cancer groups and not really for our patients and other things were the opposite. So it's just kind of maybe having a table of things to select from for the options.

**SB**

Yeah, very good. Um, what do you think are the barriers to the long-term delivery of this programme? I think you touched on a few of them in terms of funding.

**P16**

Yeah, I think the funding is the thing because this has been done as a pilot and what's going to happen when that that runs out? Some things I suppose may still run under the auspices of McMillan. But yeah, we need it will be good to build on that.

**SB**

Properly resourcing it, yeah. I don't have any more questions, P16, is there anything else that you would like recorded with any final comments at all?

**P16**

No, I don't think so. I think we’ve covered it all

**SB**

Super I’ll just stop the recording

# P17

SB 0:03

Okay, um, so just to start off, and what did you know about Cancer Rehabilitation before the service started in SE Trust.

P17 0:14

So before the prehab, service started, I had only been in post a short period of time. But as as a Macmillan health and wellbeing coordinator we have information support services regionally and in other trust areas prehab had been piloted, I think, specifically in the Northern Trust. So I was familiar with the processes behind it. And even our service itself is essentially, it does prepare patients for helping them to live well, or giving them an opportunity to live well. So, I had had a relative understanding of it. Yep.

SB 1:02

Great, great. And just obviously, being involved in the whole project, what is your thinking about prehabilitation now?

P17 1:13

Yes. So I think it's, it's fantastic. It's great that we're able to pilot it here in the SE Trust and seeing the impact it has on patients whenever they do engage with with a course of rehabilitation and seeing the effects that that has on their recovery is brilliant. And I think there was a study in Manchester, and that was the one that we were sort of basing our model on, and you see all the research and the data that's come out of that its proven to be a really, really good way of getting patients back up on their feet. So it's brilliant.

SB 1:58

Brilliant, and very important part of the service support package in there and for cancer care.

P17

Yeah.

SB

What is your role in rehabilitation programme in SE Trust.

P17 2:15

So I think there's, there's obviously different elements to the support offered to patients throughout prehab. And I think our involvement has been a lot smaller than some of the other elements. But essentially, our role is to offer emotional wellbeing support to patients. I think there's a form that patients have to fill in, and if they score a certain level, then they're referred on to our service. And we do very low level emotional wellbeing support over the telephone. And based on that conversation, if it's deemed that the patient needs a bit more support, then we would refer them to our counsellor.

SB 3:06

Okay. Okay. And in the development and planning of all of this, were you involved in setting up the emotional sort of component of the project?

P17 3:18

Yes. So there was various different prehab meetings, where all relevant stakeholders were involved. And it was agreed that that would be our involvement. We offer emotional wellbeing support to all of our patients, generally. Anyway, so it's nothing new or different to what we would offer. So it was just making that connection at that particular stage for the patient.

SB 3:44

Did you receive any training as part of the sort of development of the programme?

P17

No.

SB

No, no. And I know that you will have worked with the likes of the move more coordinators. Were you involved in delivering any training for them? As part of the … [participant indicated non-verbally no]…No, I just wondered that.

P17

Yeah. No, just that I've been chatting to them to. Do you think there's any way you could have been better prepared for your role in the Cancer Prehab programme?

P17 4:19

No, I think it was, it was a pilot and we have to work with whatever we have in the SE Trust. But I think we had a good setup. And I don't think there's anything more. I think [CNS HNC] had considered all the different options. And we were all involved in that process. So I think coming off of COVID was what made it kind of a bit trickier. And, yes, I don't think there's anything that we could have done to be better prepared. I think moving forward, there's probably elements that can be tweaked. And that's obviously what what this this is for?

SB

Yeah,yeah. And so we just want to talk a wee bit about the assessment and referral process for the programme. And you would have been receiving referrals. Yes. So what how did that work for you? And was there any issues with the referral process?

P17 5:25

No not at all. So we, we generally receive referrals for our day-to-day support of patients anyways, so they gave kind of joined inbox email where we would get our referrals. And that's where the referrals came through. It was clearly marked as prehab. It was obviously password protected, and all the information that we needed was on the referral. So there was there was no issues in relation to the receipt of those referrals.

SB 6:02

And then, in terms of patients you received, through the referral process, were they interested in engaging in concert prehabilitation?

P17 6:15

in relation to our support for majority of patients, at the point of our contact with them, weren't, weren't as engaging as I would have hoped. So it was the majority of phone calls, it was making contacts, kind of reminding them of the prehab process and how they came to us, and then exploring the supports available to them. And, and making that offering, but at there were quite a few that declined to avail of any further in support.

SB 7:00

And what why do you think that was, was there feedback?

P17 7:06

I don't know. I think obviously, whenever they've done their assessment, and they that scores given, they probably may not feel that it's necessary for them to have that support. It's just that that's what we've sort of set the bar at. So and then, if if patients are agreeable at the point of referral, things may change, by the time we've made contact with them. So we would, we would find that a lot with our general referrals anyway, so if a CNS would refer into our service, and they may say, this patient needs, would like a referral to counselling. By the time we've made contact with them, they may have changed their mind for whatever reason. So it's hard to say exactly what it is. And then there's, there's some that we would have referred on to counselling. But we wouldn't follow that up with the counsellor or with the patient. So it would be interesting to know whether they did follow through with that themselves, or if the counsellor was able to, to set up regular contact with the patient. So yeah, so.

SB 8:28

So that's possibly an element of the prehab that’s unknown.

P17

Yeah.

SB

Okay. And did you receive more or less referrals than you expected? When you know, you were planning this programme?

P17 8:42

And I wasn't sure what to expect. But I would have hoped for more referrals. It would have been good if there was a larger cohort to sort of gather that data from, but there were still good enough numbers would have been, I would have wanted more personally.

SB 9:05

Okay. And in terms of just thinking about the screening tools that were used for referrals for yourselves. Do you think they were appropriate? You were saying there that you got people that didn't want support in the end up, so was the screening tool picking up too many and being too sensitive? Do you think…

P17 9:27

…it could well be, but I think whenever we would, were designing it, we were trying to capture as much as we could. So yeah, maybe that that is a learning, but then it's given the patients the opportunity as well. So if if you're not giving them the opportunity, then you're going to miss a few as well. So it's a tough balance that one there.

SB 9:56

Yeah, yeah. What impact did COVID-19 have on the delivery of your service as part of the prehab.

P17 10:08

We, so there was a lot of face-to-face appointments and meetings that didn't happen as a result with COVID. So that makes it a bit more difficult to to build that rapport with patients that you would normally have the opportunity to do. Our supplementary services that we would refer on to so the likes of counselling in relation to prehab… they were suspended for a period of time. And then whenever they were coming back up and running, there was a larger waiting list. So yeah, there definitely was an impact as in relation to the smoothness of the project running.

SB 10:57

How did it work and just out of interest, whenever you received the referral, if there was a waiting list would that mean people wouldn't have been contacted before their surgery potentially.

P17 11:08

So I have relationships with the SE trust. So if I send a referral through urgently, then they would make contact within a week, or a week and a half at the latest. But I can't, I don't have the information in front of me. But say for example, if there's a patient that's referred outside of the SE Trust area, for example, Belfast, then that's a different story, because I don't have as much sway with the counsellors outside of the SE interest area. So, yeah, because head and neck is a regional service, as well. So you would get patients outside of the SE Trust.

SB 11:54

Okay. And what benefits have you experienced in terms of your professional development or practice from being involved in the programme?

P17 12:04

So for me, I'm not a clinical member of staff. So to be involved in something where it's a nice clinical focus, and it's good for me and my my development, just to see how that works, the all the work and the moving parts. And to find out a bit more about the clinical element in relation to what patients go through. So that gives me a better understanding wherever I am supporting patients as to what that side of their treatment and recovery looks like.

SB 12:39

And what about were you involved in? Are you a member of the working group that meets quite regularly?

PB 12:45

Yes, yeah. I don't know if they've met in a while or if they have met, and I haven't been involved. But initially, whenever it was starting out, I was part of a group. And yes, I have met with them for several months.

SB 13:01

Okay. Was working group helpful? Was it helpful to hear those conversations to be party to all of this sort of as a project development forward?

P17 13:26

Yeah, definitely. There were a lot of decisions made in the working group meetings. And it was good to see the Health Improvement Team and cancer services here work so closely together, as well as some by HPs, as well. So that was good to have the different professionals working so closely together, finding a way to support these patients. So so yeah, that was good.

SB 14:01

Yeah. And have you enjoyed being part of the programme?

P17 14:05

Yeah, it's been good. It has indeed.

SB 14:09

So you mentioned at the start about there being some possible improvements that could be made to the programme. Would you like to just talk a little bit where you see the improvements could be made both for potentially moving forward and SE Trust, but even thinking about as this project rolls out, where are there improvements needed?

P17 14:31

Think, I'm not sure if you know much about our service, but we we support patients with all of their non-clinical needs. So the way our service is set up, is that we’re supposed to receive a referral for any new diagnosis from this clinical team, particularly from the CNS of a patient then we make contact with the patient and inform them of all the supports and services available to them throughout their journey. So there's there's kind of similarities in the way that we would support patients. And our support covers things like Move More, benefits support, emotional wellbeing support, complementary therapies. And we deliver those supports in partnership with many different partner charities. So, for example, if we make contact with a patient, and they say that they would like to engage in physical activity, then we afford that referral and to Move More. So I think that moving forward, there's a way that our service can gel better with the prehab side of things. I think, if if we are receiving referrals for all patients, then we should be covering that with the patient. Anyway, if you know I mean? Yeah,

SB 15:56

yeah. So you're thinking that patients could be offered more, earlier? Is that what you’re thinking?

P17

Yeah.

SB

Okay. And, and just one of the the things I'm hearing from other people is part of the challenge of that is, when the patient receives a diagnosis, there is so much information. So is offering them more adding to the challenge, you know, in that early, you know, pre surgery stage.

P17 16:28

So that's, ya know, and it's a fair point. And it's something that we would get from our clinical team quite regularly. There's some tumour sites where there's a, there's a lot that their discussing with patients. And there's, there's some tumour sites where there's even more than they have to discuss the likes of lung, the likes of head and neck. So they do feel that something additional is, is a bit too much for patients. And I think that is fair, but I think there's ways that you can frame the information in order for patients to receive it. And I think there's…. Yeah, I think I think it can be done. And it can be done in a way that patients will receive the information. And then, even if they don't take it all in, we make that phone call with them. And we make that connection within a week within two weeks, and then we can kind of explore all those options with the patient. And we generally find the patients are very open to having that conversation at that point whenever we do make contact with them. And it could be that they don't want to engage in anything. But because we've made that connection, they'll come back to us in a month, two months, during treatment, post treatment, to ask questions or to want a referral to Benefits Service for an entitlement check or want to explore complementary therapies or whatever it may be.

SB 18:15

Yes, you've opened that door.

P17

Yeah. Yeah.

SB

Which is so important. So important. Are there any other improvements you would like to say to the programme from your point of view?

P17 18:26

No, not, not that I can think of that's, that's the one thing that I felt in relation to our involvement with the programme. But no, did anything else pop up for anybody else?

SB 18:40

No, no, nothing that I can think that would link with you but its very striking the way that you have worked to date has fitted really well with this project and the service that you did offer or do offer, has fitted really well and could enhance the project further, which is that's really fabulous to hear. And what do you see as the barriers for the long term delivery or prehab programme?

P17 19:11

I think with everybody would say the same, funding, capacity. And to be honest, it hasn't added that much more to our day-to-day delivery. With other other teams that have been involved, it probably is a bit more, the likes of Move More, they probably it's more involvement for them. So we we probably could sustain this. Our service could sustain this delivery, moving forward. But I don't know if that will be the same for for other partners that are involved.

SB 19:53

Yeah. Have you seen the benefits of this project for anybody you have worked with?

P17 20:01

I haven't with any specific patients that have come through our service. But I obviously know the data is there. And I know that we'll we'll have the data to support that. And the way that our service generally works is we don't know what happens to patients, we we we connect them with, let's say, complementary therapies, they'll have a series of complementary therapies, and that should help them with any side effects or symptoms that they're experiencing. But we won't know that for sure. We will refer them to get benefits entitlements check. And they may receive some financial support, but we won't know that information. And we don't go searching for that information either. So So yeah, we we do what we're supposed to do as part of our service and then we hope everybody else does what they're supposed to do. And if not, then the patient will come back to us.

SB 21:09

Okay, okay. Yeah. Have you any final comments? I had no more questions for you. But any final comments you want to make or issues that you want to raise that I haven't asked about?

P17 21:19

No, I mean, I'm not sure if you if you are aware, or what are the plans moving forward? Once this evaluation is complete? Is there any sort of plans in place for anything moving forward?

SB 21:34

I'm just gonna stop the recording and I’ll answer your question.

# P18

SB 0:03

Okay. So what did you know about cancer prehabilitation before it started at SE Trust?

P18 0:10

Okay, so my, in the first time I heard anything about it so, so I managed the stop smoking service. And we had gone to Manchester, and told the hospitals or to hear more about the Cure project, which is a fabulous stop smoking service within a few of the Manchester Hospitals. And then I had a couple of the managers there had come over to do deliver a seminar for us around stop smoking. And one of the girls who came over was actually that cancer lead for Christie Hospital in Manchester another Manchester hospital. And then of course, you get talking and, and linking in, then I'd heard about [name] who actually runs the prehab, the cancer rehab. So then I had gone to [CNS HNC] and said, Have you heard anything about this? And then that's where kind of and then we set up a meeting with [Manchester cancer lead] and [CNS HNC] and a few of the other leads, and then that's kind of where it kicked off from us as the way I remember it.

SB 1:07

So wow, I did not know that. So very interesting. I have an amazing how you just meet one person that leads to another?

P18 1:18

Abosolutely

SB 1:18

Yeah. So what was your sort of thinking on prehab before you were involved?

P18 1:26

You know I hadn't known a huge amount about it, but of course, so we had linked in very closely around a smoking cessation and cancer patients. And obviously, we would be always trying to help smokers to stop smoking before surgery, we would have got referrals from pre op department and stuff. I had never really thought to be honest about physical activity and nutrition and stuff. I haven't specifically thought about that for prehab. But actually, it makes perfect sense. And a lot of my work would be in prevention and secondary prevention. So this actually whenever you read the pathways, and whatever, it all makes perfect sense to actually try and build somebody up and before surgery or before treatment. So it all sort of makes sense. But I really didn't know a huge amout about it, per se.

SB 2:13

Yeah, yeah. And can you tell me a little bit? Sorry, there's a bit of an echo there. And could you tell me a wee bit about your role in the cancer prehab programme.

P18 2:23

So I would have gone to some of the initial meetings, the steering group meetings. However, I was redeployed for part of this past year, so I wasn't able to make all the meetings. And then obviously, we did the pathway for smoking cessation pathways within prehab. So I did my bit but probably a lot of the other members of the group did a lot more work on it to be fair, because I was because I was out of my own job for quite a while. But I've kept, you know, kept in touch with what was happening and stuff. Yeah.

SB 2:59

Did you feel that you were able to shape and influence the project in the way that you wanted to see that to benefit your patients?

P18 3:07

Oh, yes. From what from a smoking cessation point of view? Absolutely. And I would have felt, I mean, if there was anything else that would have queried, I would have felt quite able to say whatever. Yes, I felt about it. Yeah, very welcoming group and very dynamic, so that were quite keen to hear other people's views. Yes, for sure.

SB 3:26

Um, is there any way that you feel that you could have been better prepared, it sounds like you work quite well with having those early conversations and hearing from other people delivering the programme, but is there any other way that you feel that you could have been better prepared?

P18 3:40

Um, no, it was. So it was wrong. It [CNS HNC] and [HEALTH IMPROVEMENT MANAGER], were fabulous. They sent, you know, they kept sending us information about the project. The only thing that I'm thinking about recently, for a lot of our new programmes and stuff is having a service user at some of some of the some of these meetings maybe might have been useful. Although I do think there were there were conversations going on, but it might have been useful to have somebody at these meetings to give their perspective. And obviously, because PPI is everywhere now, we should probably have had that. But apart from that, no, the information we got back on the was was fine.

SB 4:21

That's that's very interesting to hear that and PPI is so important. And did you encounter any challenges with the screening referral or consent process? So in terms of the referrals you were receiving through to smoking cessation? Did that go smoothly? Or what was it like for you?

P18 4:42

Sorry, you went you went? You froze way back there. I missed that question. Could you just go over that again? sorry

SB 4:47

No problem. Just did you encounter any challenges with the screening referral and consent process? So receiving referrals into smoking cessation? Were there any problems or did that go okay for you?

P18 4:59

No perfectly fine. Know, to be fair, we already had a pathway set up. So we just had to tinker it for up to the we were always did head and neck closely involved with the head neck patients. But the other two tumour sites, we hadn't had a lot of proposed. So we just were able to adapt it to that to those. So, no, it was perfectly fine it worked similar to the way I've had previously.

SB 5:21

Very, very good. Were there any helpful aspects to the process that you find in terms of how you received the forms or was not exactly the same? As before?

P18 5:34

I would say it was, it was the same as before. I suppose what was helpful was some of the other staff then kind of even having a, an awareness of the stock and service and what we can provide. But, ya know, our referral processes are quite standard. So yeah, we didn't tinker with that much too much. No.

SB 5:54

Okay. And how interested did you find the patients that are coming as a referral from the prehab?

P18 6:02

And I think you get a variety of responses. And I do think that having a health professional advise somebody to come to the likes of smoking cessation absolutely increases their their likelihood to engage with us. And having said that, obviously, if somebody is going through a cancer diagnosis, that they can be an anxious they can be agitated. And that's always reasons for them to carry on smoking. So it's, it's a bit of a challenge. But I do think having that health professional kind of advise people to even consider something like smoking cessation, it does increase at least the initial engagement.

SB 6:41

Yeah. Okay. And did you find any resistance from patients, or patients from particular tumour groups was more successful? Do you think and some more than others?

P18 6:54

I think, to be honest, the referrals haven't been huge, it would be difficult to kind of be too general around that. And what I would say is, I think the head and neck patients seem to do seem to do much better within smoking cessation services. And there's probably a variety of reasons for that. But the numbers aren't big enough, I don't think for us to be able to kind of really give an answer. An answering that, you know, comparing between the groups. Yeah.

SB 7:31

And are the referrals less than what you expected them to be going into this project?

P18 7:37

I think they're much the same. Bearing in mind that a lot of our referrals had really reduced over COVID. They're building up slowly, anyhow. I think this just refreshed the fact that to the staff that we can take the we are taking referrals for cancer patients, so it kind of just it probably increased them a little bit... the number of referrals.

SB 8:01

Okay, you had mentioned there that, you know, COVID has had an impact. And can you just talk about that impact on your service? Well, were you redeployed because of COVID? Or was was? What was the...

P18 8:19

So I usually deployed however, I'm on the service. So the team that we're running the services that has been delivered the whole way through COVID. And I was redeployed because of COVID. And what was the other question, then? What How has COVID impacted the service?

SB 8:33

Yeah.

P18 8:34

The referrals have definitely gone down. Obviously, the staff in the wards have other priorities. For a start. The referrals had produced then with lockdown people actually, who had stopped smoking had actually then started again, because they were in the house. So there was all sorts of impacts due to COVID. But we're building back up again, which is a good thing. The staff are really keen, we're starting back on the ward soon as well. And that'll hopefully help with our delivery as well.

SB 9:04

In terms of a stakeholder in the working group, did COVID bring any benefits from your ability to engage with the working group, I'm suspecting no because you're possibly then off to the side. But did anybody go in your place to that working group? Or?

P18 9:23

Um, I don't think I don't think I don't think so. No, I think I would have attended but then I was always able to get information back. What I would say is because we're now so familiar to use ZOOM, at least I was able to attend some of the meetings whereas prior to prior to COVID, I don't think any of us would have had known how to link with ZOOM. So it probably had a positive impact in that sense. But no, the girls were busy delivering so I didn't ask them to attend that the working...

SB 9:53

Okay, okay.

P18 9:56

meetings. And anyway, our so our manager was our manager was probably there and she is very familiar with smoking cessation. So she would have been able to talk on our behalf.

SB 10:07

Okay. What benefits have you experienced in terms of professional development and practice from being involved in the programme?

P18 10:15

Oh ....well, I have met give a few more staff that I would that I wouldn't have met prior to this. I think that has increased my knowledge in cancer services. I mean, I'm a nurse, but I had never particularly worked in cancer services before. So I was able to understand things like, from a lung cancer perspective, you know, that quite often people present late, which gives a narrow window then if you're going to do any prehabilitation... the head and neck, I've always had a fascination around the head and neck clients, so it kind of increased my knowledge, a bit about the head and neck pathways and things. So it was actually it was it was very useful to me. Yeah.

SB 11:03

And just on that point, that you're in there, but the very short timeframe between even a referral and potentially somebody going for surgery, we yous able to step in in that short timeframe or was that difficult?

P18 11:19

Yes, we were able to step in. And actually, I mean, lots of evidence to say that even if somebody stopped smoking about two to three days, prior to surgery, they will they will be they need less anaesthetic, there'll be, you know, their bad days post op will be less and stuff. So smoking cessation is one of the ones that you can't even a few days can make a difference to outcomes. So yes, we were able we were able to go to go. And usually, quite often, it may have been only a few days beforehand, but at least we'll be able to get that across to you know, to advise somebody not to definitely to at least try a couple of days before they have their surgery, and it will impact on their perioperative on the postoperative care.

SB 12:04

Thats so good to have yous as part of that model.

P18 12:09

Yeah. Yep.

SB 12:11

And, and and what improvements do you think going forward for this project are required? For the the project in SE Trust or thinking if this was to be rolled out recently, What do you see, from your point of view would help make it better?

P18 12:37

Oh..... I'm not exactly sure I think is wrong perfectly well, obviously, there will be evaluation reports on stuff and maybe even stuff from service users. As far as I'm aware of the service users were actually really impressed, particularly with the physical activity component. So and hopefully COVID, you know, as we get back face to face, I do you think those relationships are important for somebody to engage with a with a with any of these services? So I think that the face to face aspect will make things a lot easier for it. From a structure, processes point of view. I can't I'm not really I haven't really thought about this [interviewer name], to be honest. So... I was very impressed with how it all gelled together. And the work that had to go in together to all work was was amazing.

SB 13:29

Yeah.

P18 13:30

I think the guys were fabulous. And the way that there was so much work involved, I hadn't realised how much work would have been involved and in getting this up and running. I mean, in the, in the Manchester model they had, they had a lot of financial support. They had, you know, people specifically on this programme, so consider we were doing it in-house and you know, with very little finances, I think it's, it's been fabulous.

SB 13:57

Yeah, it's incredible. And the amount of goodwill that has pushed the project along.

P18 14:03

And to be fair, to be fair, [CNS HNC] can motivate people and get people around the table and do that's kind of exactly what made such a difference. Yes, exactly.

SB 14:13

Yeah. And just this side point, was there anything, you said you had looked at smoking cessation services over Manchester, and then you had discussed with them about their prehabilitation? Was there anything you change to your current practice as a result of engaging with them at that time that maybe rolled into this project? Was there improvements made before the start ... do you know what I mean?

P18 14:44

No, probably not. Probably not. I think it. Yeah, because our service took such a dip, we have we with COVID our struggle has been to get things even back to were to the to the previous baseline. So in order to improve, we need to get back to our baseline, and then we're going to, and then we're actually going to be introducing the Cure, the Manchester smoking cessation project. So that's in the planning at the minute. So we will be doing a lot of changes, but I couldn't honestly say that we've made any changes to to the cancer prehab programme.

SB 15:30

Yeah. And that programme that you are planning to implement will not bring improvements, potentially, to the prehab programme, will it change how that works?

P18 15:42

Well, so the care project is specifically that every smoker who is in acute care will be offered medication to help them quit and will be seen by a specialist within probably 24 hours of admission. So that that will be ... Yeah, so we did, we were involved in the national British Thoracic Society audit just before Christmas. And the preliminary findings are that probably 80% of smokers are admitted. And then leave hospital aside, haven't had any conversation with a health professional about smoking and are not getting products. So we're a huge mountain to climb, but we can know what and what direction we need to go in.

SB 16:26

Yeah.

P18 16:27

And obviously, then that will cover patients who are admitted with cancer diagnosis, or even admitted for investigations. So I am actually not will, that will, in some way to cancer services, for sure.

SB 16:41

Absolutely and potentially pick up people earlier than the actual.

P18 16:46

Yep, yeah. Yeah.

SB 16:49

Okay. Um, and just finally, have you any sort of like thinking around what are the barriers for the long term delivery of this programme in SE Trust, the prehab programme?

P18 17:00

I would imagine finances, finances always comes into these these programmes. And there has I think, with the physical activity staff, there are move there's movements around those staff being then maybe moving into a different council area and stuff. So I think you would probably, you know, if you're going to do it wider, you probably need dedicated staff specifically for this. And no, it probably needs if you probably need dedicated staff, and maybe admin staff and, and things like that, but it seems to be going it's definitely got we've laid the foundations, I think it just needs to build upon my now...

SB 17:42

Yeah.

P18 17:42

is my understanding.

SB 17:44

Yeah.

P18 17:45

Yeah.

SB 17:45

Thank you so much, surely, um, have you any final comments? No, I haven't asked you that you were thinking about before the interview? No, I'm

P18 17:53

I'm just so impressed with how everybody how everybody has worked so hard to even though I was only up maybe one in three meetings. Whenever I did comeback there has been so much work going on in between times, it was just fabulous. Everybody worked really, really hard. And it was the right people on the working group. And everybody seemed to know that was you know, the way, I don't know about you, but I go to meetings and there's not a big lot comes out whereas every meeting, it was structured, everybody knew what they had to do. And we all were seemed to be pulling in the same direction. So just well done to everybody who was involved in that.

SB 18:26

Yeah, fabulous and not as thought as a threat across in the interview is just people are kind of blown away with how well people worked together.

P18 18:35

Absolutely. Absolutely.

SB 18:37

That's super. I'm just gonna stop the recording here.

# P19

SB

So [PARTICIPANT NAME], thinking back what did you know about cancer prehabilitation before the programme was introduced in SE Trust?

P19 SLT

Prehab was probably in the last few years for me and our services really just started, you know we have had increased awareness of it and there has been an increase in research into it… so I mean I knew about prehab, it wasn’t something that we, our service that we provide at the minute, it is purely inpatient, pretty much post-surgery, once they leave, you know we are only employed to work as acute therapists, so once they leave they go to community, we don’t see them again, well not very often. If they come back to the Trust they tend to be picked up by our community therapists, I might input just a little to provide advice and support, so there was really no sort of opportunity or anything or funding within the acute service. I mean my post is not a funded cancer post, so our head and necks are just see as part of our general caseload, they are picked up just along with everyone else on the caseload.

SB

Ok so you do SLT for a range of different conditions and HNC is one of those

**Signal break – interview stopped and restarted.**

SB 0:18

Okay, so yes, you were explaining there about how your role is much broader than prehab in cancer care. Okay, so um, what is your view of engaging and patients with cancer before surgery with regards to speech and language?

P19 0:48

Okay, so and the vast majority of our patients with cancer are probably are head and neck cancers. When we do see other cancers. Are you wanting to focus on purely the head next?

SB 1:00

No.

No, generally like the lung and colerectal too. But um, yeah, but speech and language, obviously you're probably rule was with the head and neck then?

P19 1:10

Well yet, but we do see other cancers in the hospital. So we do see them as well. And, yeah, definitely prehab is something that really we should be absolutely involved in. It's really vital. Just from the little bit that we did just getting the opportunity to see those patients before now the head and necks were surgery. Before they had their surgery to be able to answer any questions with regards to their swallowing or communication problems, to have a chat about what we would, their our expectations and theirs, you know, after surgery, it really was from I feel that psychologically those patients were better prepared. From my point of view, not thinking better prepared for surgery, but better prepared for post surgery input and engagement.

SB 2:07

Okay, yes, yeah. So can you tell me what your role has been on the cancer rehabilitation programme?

P19 2:16

Okay, it was relatively limited. So because it was, because it wasn't a funded, there was nothing funded, so it came out of it was extra additional hours for me. So I did it very temporarily. And which is unfortunate. On one hand, you know, on the other hand, though, that really these you know, it needs to be funded, we can't go on doing these things unfunded. So we made a decision, though, that we would do it, briefly. So my role was in The Prehab Clinic. So seeing the patient, so I saw them individually, or whoever they came in with. And we did, we got a baseline assessment of communication of their swallowing, of their, their sort of quality of life around swallowing and, and communication, if that was appropriate, and did an assessment if necessary, provided some advice on if they needed to modify their diet at all. And gave them some prehab exercises to take them for them to go away and do in the hope that, you know, they, we could maximise their current function, for swallowing and communications so that when they went for their surgery, they were in as best a place as possible from a function point of view. So muscular and things.

SB 3:54

Yeah, you're saying really

That was really my role.

Yeah, you're saying they're from a functional point of view. But all of that I'm sure contributed to the emotional. Psychological...

Absolutely..

resilience. Yeah,

P19 4:05

Yeah. Yeah. I think I don't know whether, I mean, I know that the [CNS HNC] and [CNS HNC], the cancer nurses are super about taking their patients, they go on a real journey with them, you know, and preparing them psychologically for it. But I saw I find as well that maybe it's just being a speech therapist, and people come in and they, they think before they come in, they think I'm going to have to talk to this person, that you know that they are very, we also have the time often particularly on the wards to actually sit with them, talk them through whatever their current issue is, you know, and really kind of sort of empathise with them. But yeah, part of their role really was in managing or trying to optimise their function. A lot of it was preparing them and getting their engagement pre-surgery so that post-surgery, they knew exactly what they were supposed to be doing, and it wasn't going to be a massive shock.

SB 5:05

Yeah.

P19 5:05

And you know, even that even the fact that they have seen you, they recognise you. It just helps you not a stranger coming into the room to know that they're like, Oh, I saw you before, you know. And even that is just, it's, it helps.

SB 5:22

Yeah, yeah. Can you tell me were you involved in this sort of the early days in terms of the planning and development of the project? Were you part of your working group?

P19 5:38

Yes, there was this awh God and trying your now. It stopped with COVID. Everything kind of got put on hold for a while. But yeah, there was a there was a Trust group, but looking at prehab, which I was party to. And, you know, we had decided then that we were going to look at the head and neck was one of the strands that we were going to look at, you know, and then yes, it was party to how we were going to develop that prehab clinic. And particularly with the with the head and necks, it was really just the cancer nurses, and Speech Therapy and Dietetics were involved at the prehab. And we were kind of all around the table setting trying to work out how we logistically as well, we were going to manage.

SB 6:26

Yes. And do you feel that you're able to contribute to that and and get your point across and feel heard?

P19 6:34

Yes, I think so. I think yeah, I mean, we're in a relatively I feel we are in a quite a good position here in that while currently, we are unfunded, there's a lot of recognition, thankfully, and awareness of how vital speech and language therapy are, particularly to the head and neck. I mean, do you know of all the AHPS really swallowing and communication is there always going to be affected by head neck cancer? So it is, we're really lucky here that it's very much recognised, you know, and, and that comes down right from the consultants who are so pro speech therapy. So in a way, we're really lucky that there is a real awareness that, gosh, we really need you and what are we doing? Can we do to help get this service up and running?

SB 7:27

Yeah, yeah. So can you tell me a little bit about and the assessment and referral process? So would you've got your referral from the CNS, or how did that work?

P19 7:39

Yes. We would have got an email from CNS, usually after they'd been in the patients have been in and they had seen the surgeons and they'd have made a decision that yes, we're going to proceed with surgery, then CNS would have sent an email to myself from the dieticians and said, look, this person's coming in, we're going to book them in for whatever it was a Monday afternoon was prehab session, so we're going to book them in for such and such date. And we used to just go along.

SB 8:11

Okay. Okay. And would that is so I'm just trying to kind of get this all straight in my head. So I'm going to be telling other people have talked about the Breaking Bad News appointment would that have been the same day that you would have seen them or slightly,

P19 8:26

No, it tended to be the later days. So they would have seen the consultant, usually at a previous week or two before. And so they knew what they knew that they were coming in, they had cancer, they were going to have to have surgery, they'd sort of made that decision. So a lot of the time some of them actually had a fair idea of when they were having their surgery. They have potential dates. So it was a no it would have been after that.

SB 8:56

Okay, yep. So and So yous actually had a clinic as such them for prehab.

P19 9:03

Yeah, yeah. So we had a clinic a Monday afternoon, where we had a couple of rooms in outpatients, and we used to work it to that we tended to have two patients come in for the afternoon, one after the other. Usually, CNS saw them first, and then tended to be speech therapy next, and then dieticians afterwards. And that was kind of the way the clinic ran. So we were kind of there just and we just picked up, you know, as soon as one was finished, because we were all in little rooms in a row. So we kind of worked really well you know, logistically, it was it was a really good, a good way of working. So we're all together, which was really nice. So we had the benefit of CNS, were there so could give us any updated information that maybe we weren't aware of, or following on from their chat they can they stuck their head in and said, listen, this maybe isn't going terribly well or whatever, you know, having a bit of trouble adjusting or whatever. So it was really helpful.

SB 9:58

Fabulous it sounds like that, that sort of that system of referral and your prehab clinic worked really well, did you encounter any challenges with it?

P19 10:12

Not really, I suppose the only challenge was I'm just trying to think of a few issues was just somebody might have been booked in for prehab and then you realise that you know what I'm actually on leave that day or do you know there was things like that would have cropped up. Or sometimes, if you, if we had two patients booked in, there's a lot of hanging around. So while CNS are seeing them, and you don't know, some people are in and out in half an hour, some people are an hour so you're kind of, there's a bit of hanging around there. But, you know, for me, as long as I had a computer, I was okay, because I can, I could just work away on all their stuff. So it didn't really matter. But, you know, if you were unable to do that, there's a better tooing and froing. Or, you know, we'd go over and they'd say, gosh, you know what, this person isn't turning up, we've just got a phone call. So we'd like your back over again. So, a few logistical issues, but on the whole, it kind of worked terribly well. It worked reasonably well.

SB 11:20

Okay. And, the patients you received, I am, I'm asking this question. I probably know the answer. But, they were appropriate for your service.

P19 11:32

Yep, yep.

SB 11:33

Yep. They're all yeah, um, we're screening tools used in that process? That you know,

P19 11:40

Do you mean to for the, to decide whether the patients would be applicable to come for prehab?

SB 11:47

To Speech and Language?

P19 11:50

I don't know, you see, it's, we're a little different in that anybody any head and neck cancer patient who is going to have surgery is going to have speech and language therapy input? So it's a bit of a given?

SB 12:03

Yeah, no, I'm just checking because there was a number of screening tools yours done, I just wasn't sure if that was...

P19 12:10

sure whether there's a screening tool that involves a little bit that has something about speech and language in it. But as I say, like most of the patients, and it was the patients coming through prehab were the major head and necks. So they were the ones that were going to be in, they were flapped, they had a trackie. So you knew they were large, big surgeries, so they were going to need us there wasn't going to be any question about that they might not need you, you know.

SB 12:35

Okay. And how interested did you find the patients were engaging with you?

P19 12:41

On the whole? Great, really good. Yeah, I, on that. I mean, there's, there's the odd patient that, but it's more I think, it's just personality type as well, or the type of patients you get patients who are, I do you remember when man coming and going, you know what? I don't need, I don't want to know any information. I just would literally want to turn up for my surgery, do whatever you need to do and afterwards, tell me what I have to do and I'll do it. I don't want to know any of the details beforehand. So somebody like that i your like, absolutely fine. Listen, we're here, if you need us, here's the phone number. You can ring if you have any queries in the future. But you know, at that point in time, he obviously was not ready to take in any information. But, more than happy to, for me to get a baseline of his if he had any difficulties at that time, you know, so even everybody certainly engaged enough that I could get an idea of where they were at that time, whether they needed any advice or support, whether they wanted it like that man was not going to be doing any exercises. Absolutely not. But, you know, that's just that's just the way he was managing it and the way he was dealing with it, and you know, what, if that's what it takes them

SB 14:08

Absolutely... Yeah, it sounds like me having my first child. I didn't want to know a thing.

P19 14:18

Yes. Yeah. [Laughs] And yes there would be a bit of me would be exactly the same. I don't want to know what you're doing. Just do it. Talk to me about anything else.

SB 14:29

Yeah totally.

P19 14:30

Yeah i totally understand.

SB 14:32

Yeah. So did you receive sort of the amount of referrals through that you would have expected? Or was that more or less?

P19 14:42

I think there's actually probably less than maybe I thought there would be. Now we sort of have thought you know, to in an afternoon because by the time they get through everybody, and you know, you're really the first one usually come in at maybe I don't know, half one or something. So, by the time they saw the CNS, I maybe didn't get to them to half two, then the dieticians were half three, maybe. And then you've another one, you know, so it kind of only left time for two. But there were times where, you know, I do remember there being the odd Monday afternoon where there was nobody was coming through. But usually that was because either when in the on Monday mornings, there is a full, like an MDT cancer meeting across the royal, sort of a regional meeting...

okay

...and they discuss, they discuss patients plans, so they all discuss whether this patient is going to go for surgery, whether they only need radiotherapy, or whatever. And so sometimes you just might find that actually those people on that list, actually, nobody's coming for surgery. So they didn't come for prehab.

SB 15:51

Do you know what I mean? Yes. So there was a bit of a fluctuation them?

P19 15:55

Yeah, yeah. Yeah. It sort of didn't really matter too much. Because normally, on a Monday, I don't work on a Monday afternoon. So because it was additional, it just meant well, that's fine. I just I don't need to stay, you know, so it didn't really impact on me too much.

SB 16:10

Okay. Gosh, so you were doing this in addition to your normal job?

P19 16:15

Yeah. Yeah. Yeah.

SB 16:18

And one thing that struck me through these interviews [PARTICIPANT NAME] is the amount of goodwill and how the project has, has largely been facilitated, because there's been so much goodwill and desire to improve.

P19 16:36

You know, we know that we knew that prehab is really important. And, you know, for us, if we can do anything to promote SLT involvement, it's only going to be beneficial for the I mean, the patients are the winners not the winners, you know, they're the ones who are going to benefit at the end of this. And, and, you know, it's, yeah, it's a bit of a no brainer, you know, you really want to help out as well, for something like this pilot, you wanted to help out and do it. Now, it, I did it for maybe, I don't know, a month or six weeks, then circumstance, circumstances changed on me to be home on a Monday. So and at that stage, I had had not had enough, that's too harsh. But, you know, I had sort of done it and thought, no, do you know what I've got the information I need to sort of to be able to carry on, and it's now up to them up there to sort budgets and try and fund this. So it will be interesting to see what happens.

SB 17:39

Yes. And really, hopefully, this evaluation adds a lot of weight to that.

P19 17:45

Yeah

SB 17:46

And what impact did COVID do you think have on your ability to provide speech and language support that mean, did that? Did your prehab clinic happen face to face, regardless of COVID? Or was your involvement pre-COVID?

P19 18:02

It? No, it only started last my involvement, I think started about last March, April time,

SB 18:11

We were in restrictions, we were in lock down at that stage.

Unknown Speaker 18:14

Sort of, but outpatient clinics have been restarted up for a lot of people. So people were starting to get used to coming into the hospital for clinics also head and neck cancers had got back up on their surgeries, which they've been really cut back. So, you know, they'd worked through, you know, getting green pathways and things in the hospital for these patients. And so, it at that it was kind of a good time for prehab to start. It had gone on the back burner for the previous year. And it was just a good time. I think everybody thought yeah, patients are coming back in there's no reason why we couldn't start this. So I think it was last March or April, not the one before.

SB 18:57

Yes, it was it was last March.

P19 18:59

Was it? [laughs]

SB 19:01

And yeah, but I've totally lost track of lockdowns, they are just a blur there is so many.

P19 19:08

Yeah and i kinda need to count back my years. Years of COVID. So yeah, yeah. Last March, April,

SB 19:16

Do you think there was any, like benefits from COVID 19 being an issue, you know, on on service provision, was there any way or on the development of the project itself that COVID, did evoke any changes?

Unknown Speaker 19:35

I suppose it I mean, it put it back, obviously, I think it probably would have happened sooner. And if it hadn't, you know, and obviously COVID had a major impact on cancer services. You know, in general, so that was going to obviously then have an impact on people coming in for prehab clinics too. I don't know if particularly with regards to the prehab clinic, I don't know whether there were patients who just decided actually, they would rather not come in, you know, for an appointment at that time, maybe I mean, we certainly, you know, everybody's now zooming. So and there's a lot of zoom consultations with patients. So, you know, we maybe could have done that instead. I don't know whether whether with [CNS HNC] whether she was offering that was an option. I'm not sure.

SB 20:31

Okay.

Unknown Speaker 20:32

I don't know if on the actual prehab clinic, whether COVID apart from just obviously it just put it off. But once we got up and running, I'm not sure if it impacted on it.

SB 20:46

What benefits have you experienced in terms of your professional development and practice from being involved in the cancer prehab programme for that short time, and thinking just as well about, you know, being involved in that working group, or in your task and finish group in terms of getting the head and neck stuff off the ground. What sort of benefits of using?

P19 21:07

I suppose in what way it has strengthened my links with the greater team, the wider team, the MDT. And I think it has I know, certainly when SLT pulled out of the prehab, and there was a bit of, oh, no, this can't happen, really, they need to be involved. Is there nothing we can do to know? So I know it has increased awareness of Speech and Language Therapy with these and the benefits of having the service and what these patients... tell me what else, repeat tell me that question again,

SB 21:52

Just like what benefits have you experienced in terms of your professional development on practice from being involved? In the prehab programme? So you know, yeah,

P19 22:01

Yeah.

SB 22:02

You've touched on a number of points

P19 22:06

Yeah, it's probably strengthened as well, my links within Speech and Language Therapy, and other cancer therapists. So I know, with the cancer strategy, now, the pre there's a prehab strand there so good, you know, I'm getting information about it. I mean, I suppose from... from my own sort of development, you know, I always knew it was going to be of benefit to these patients. I suppose it gave me a better picture of these patients when they came back in for surgery. They were a familiar, I was familiar with their pre surgery function, which helped.

SB 22:58

Yeah, thats a good point...

P19 23:00

... post surgery, you know, so I knew where they were coming from, and which for me, was really useful. And you knew, I also knew what difficulties they'd had pre surgery. So I knew post surgery what to expect and what I could do with them. It helped my I suppose, my, my rehab then post surgery, having seen them beforehand. Yes. Probably informed that a bit better.

SB 23:34

Yeah, yeah. And whenever we started talking about this, and we were talking about prehab was and your sort of view of prehab. You talked quite passionately about prehab, and the importance of it to patients. Have you enjoyed being part of that project?

P19 23:54

Oh, absolutely. Yeah. I mean, head neck would be a real area of interest for me. I've worked with them for a long time, the head and neck cancer. So it is a real area of interest for me. So anything that I can do to try and increase SLT involvement with these clients, you know, I am really keen on and really passionate for me these these, these sort of these patients tug at my heartstrings a wee bit, you know, so and I'm, and they're the ones that I really love to be involved with, I get a lot back from them. So, and I quite like the whole, I mean, we see such a range of clients, you know, from elderly, dementia, frail, respiratory problems, neurology problems, all sorts of things. You know, and we're involved with cardiac patients, but these head and necks are, I really enjoy them?

SB 24:50

Yeah, that's fab

P19 24:52

And when you're with them, you tend to be with them for quite a while, which is really nice, and you get to know them even if you only see them as an inpatient when they're in hospital, I mean, some of them are in for weeks. So, we, do you know, and they can, the thing that's preventing them going home is the fact that they can eat and drink. It's their swallow. So we're really heavily involved with them.

SB 25:12

Yeah.

P19 25:13

Which is really nice. You get an opportunity, and often in hospitals now, you don't get an opportunity to be that involved with your patients, because they're in and out so quickly.

SB 25:21

Yeah. Yeah. And what improvements do you think them could be made to enhance the sustainability of the cancer prehab programme? I know you have touched on the fact that SLT needs funding. And that would be a major...

P19 25:37

If SLT was funded for it that would be it, it would solve everything. Yeah, I mean, that's sort of is if you like, it's the bottom line. To know, we just, you know, we need funding to provide this service. And if we had funding to provide it, it wouldn't be super. I mean, it really, really would be so beneficial for these patients. So a real a real advantage, I feel to their journey, they sometimes, do you know, these patients come and they see their consultants, they see the surgeon who talks them through what they're going to go through surgery wise, you know, the CNS is do a fine job of kind of supporting them psychologically through this, and also very practical advice. They're amazing. But particularly these head and necks, the, as I said, the communication and the swallowing difficulties are so integral to their, what is going to happen after they all have them that to suddenly see a speech therapist for the very first time after your surgery. It you know, is it's tough going. And it's of it is of real benefit. I mean, a lot of these patients have trackies, when they come round after surgery, they could have a trackie for a week. So they are they have no speech, they're not able to talk. So there is no speaking valves or anything on it, because it's just post surgery, and it's temporary. So they come around, and they have no voice, none. And you know, even being able to, I sometimes think as well, that kind of goes over their head a little bit. But the fact that we can say to them, Look, you're going to be able to talk, you're going to need a pen and paper, can you write some of these patients coming in can't write. So you got to think of other ways that that because they need to be able to communicate, they need to be able to say, I'm really sore, I need more. I need to be suctioned, whatever. So you know, it's it even from a real practical point of view that you know for the first week, they can't talk. So they're going to need something. And we need to get that in place. So that from day one, it's there. You know?

SB 27:53

That's so incredible, about how they need prepared to cope.

P19 27:57

Yeah, they will totally do. I mean, it's all very well saying to somebody, you know, you're not going to be able to talk afterwards, you're just going to have a trackie, you can't talk for a little while, but then it'll come back as soon as we take it out. And you think, okay, that's fine. But actually, when you think about it, you're not gonna be able to speak, you are not going to be able to say a peep. So are you going to mouth at people can you write, do you want a whiteboard? Or you're going to type in your phone, iPad, what's the best? So that people actually really think about it, and also that their families think about it, that, you know, they're not gonna be able to phone you. To tell you how things you know, you cannot FaceTime them because they can't talk to you. So it's really to think about how are you going to get around this? And I think, you know, that's really where we can we can come in and help from, from the very start.

SB 28:46

Yeah, yeah, absolutely. Are there any other improvements, you can see from your point of view?

P19 28:55

Specifically for prehab? I don't think so. I think if we got a prehab clinic, or regular booked in prehab clinic up and running, that, as you know, it's you're always gonna have teething problems. But I think that if we had a dedicated funded prehab clinic with CNS, dieticians, and SLT I think it could be, it would be a real advantage to the head and neck service.

SB 29:37

Any other barriers that you can see to the programme preventing the long term delivery of the programme funding obviously is ...

P19 29:48

funding obviously and then yeah, and then obviously you want the you want the necessary skills or therapist needs the necessary skills to be able to so you want the right therapist. Yeah. So you need the right grade of therapists with the right experiences? Well, and speech therapy is a very small world. And there are many of us out there, that that can sometimes be difficult.

SB

Yep, and actually one thing that you mentioned earlier in the interview was that when you were on leave there was no one to take that clinic so that would potentially be a barrier going forward.

P19

Possibly, yeah. You know you might have to, because it was kinda temporary, and it was short term, you might just have to sort of organise yourself a bit better. If you knew prehab was on a certain day then you either book your leave well in advance and say to the CNS’s look I’m not going to be here this Monday and next Monday so maybe book for another day or whatever, but yeah I suppose if you only have one therapist there then there are things like leave or training courses that will always get in the way at some point. And if there isn’t anyone else available to fill in.

I don’t have any more questions for you, is there anything else you would like to say, any issue that I haven’t asked you about.

SB

No, I really didn’t have any expectations as to what you were going to ask me about so I just hope I’ve been of some use.

# P20

SB 0:03

Okay, can you tell me what you knew about cancer prehabilitation before the service started in the SE Trust.

P20 0:13

So I have read the documents and been at the regional workshop in relation to prehabilitation. When just in the cancer arena, there was a lot of talk and discussion about the benefits of prehabilitation. So I've been involved at the very early stages.

SB 0:31

Was it something you knew about before they kind of started talking about it in SE Trust? Did you come across it before?

P20 0:37

Yeah. So at that stage, I was in my clinical manager role for cancer services, in SE Trust. So So I brought that back then into the trust. And we started to explore mechanisms for trying to implement in some of the areas, you know, some of the tumour sites, just try and pilot and see how we could get it up and running.

SB 1:01

Yeah. And what is your what's your view of engaging patients prior to surgery?

P20 1:08

Oh it's, it's brilliant, it's, it's a no brainer, if it if it helps to optimise them, the patient before they've had they're having surgery or treatment, any type of treatment, that that, you know, can have a positive impact on their recovery period, and also on their survival outcomes. But it's, it's, that's great.

SB 1:29

Yeah. So could you tell me a wee bit about your role? You sort of hinted at it there a few minutes ago, but can you tell me about your role and the cancer prehabilitation programme, right, from the sort of planning through

P20 1:45

I took the information back into cancer services within SE Trust, and then discussed with a number of colleagues, and then led the development of Working Group engaged with with three tumour sites, and got, the relevant parties, consultants and CNS's and AHPS for those three tumour sites, to start the ball rolling. I chaired, I would have been the chair for that for that working group and would have been driving that forward, you know, from a cancer services point of view. And then the, the clinical teams within the three tumour sites we picked were driving it forward, from our clinical point of view. And then I linked with [HEALTH AND WELLBEING AD], who would be Head of our health and wellbeing services, he's an Assistant Director there and brought him in to the group to try and maximise opportunities. And that worked really well. And then would have been around November 2020, [CNS HNC], I've moved into an Assistant Director role and I'd asked [CNS HNC] if she was willing, she would have been the Lead for the head neck aspects of it. So I'd asked her would she take forward the chairing and, and they do, you know, the, the trust wide programme as chair leadership and she, she took it on from there. We also had numerous calls with [HEALTH AND WELLBEING LEAD, CNS HNC] myself and Manchester, to take the learning from what they had done there. And we replicated that and in our Trust, and and then [CNS HNC] took over from there. And and you know, it's it's up and running on those three tumour sites, albeit it's it's not running at its at its full level of capacity. Because what is running at is what we were able to do within current resource, which was just reconfigured, because this was with no additional money.

SB 4:00

It's It's incredible to hear the various different points of view and one of the themes that's been coming through is the goodwill to make it happen, which is class so it is...

P20 4:14

So what we did, what we did try and do as well as we tried to maximise opportunities that were already there. So for example, we picked colorectal as a tumour site, because we knew that the enhanced recovery programme for patients pre surgery was already operational, not fully operational. But we could piggyback onto that because that's very much about optimising patients on a physiological level. So it was building on that and building in the psychological levels as well. So that worked really well.

SB 4:53

And and you touched there on it, I think I was making it ... you maybe didn't say it so, but in terms of the impact COVID-19 had on the planning, development implementation of the programme from your point of view. What impact did it have?

P20 5:11

What impacts slowed it, but it didn't, didn't pause it by any means, you know, the [CNS HNC] lead the group and lead the group very successfully. And they have been delivering on prehab across colorectal, head and neck and lung. And whilst, it's slowed it and maybe a temporary pause. It's a bit you know, it's it's operational and working very well. And, and the patients are really benefiting from it. We did have a couple of formal launches as well, where the Minister for Communities and the Minister for Health actually spoke at the launch the formal official launch of the programme, and that was when we were born maybe about two months at that point. So we had some, some good data to share.

SB 6:02

Fab, I didn't I hadn't realised that has happened.

P20 6:06

And then then [CNS HNC], with would have linked in with Macmillan, whenever there was s 10 million being released out through their cancer charities, cancer, you know, as part of the Cancer Recovery Programme, so the minister announced that it was £10 million, and the charities could bid up to a million. So, Macmillan had worked with [CNS HNC]. And I helped in the development of the bids that went in and was successful. So that has allowed, I think it was about £900,000 awarded for that. And that will allow the continued rollout here and the establishment of prehab. And some of the other trusts, albeit it's only temporary funding, but at least it gets some, some movement, you know, across some of the other trusts and allows, some of the other trusts have done smaller pilots. And so it allows them to continue those and roll them forward and expand.

SB 7:08

Yeah, yeah. What benefits have you experienced in terms of your professional development and practice from being involved in the cancer prehab programme?

P20 7:18

And, well, it's, you know, it's very much about joining services together. So this was working across HPs, it was working across some specialties within medicine and some specialties within surgery. You know, hooking in the health and wellbeing team, which is a directorate, all of its own, was really instrumental for us as well about gathering pace, because they do a lot of programmes like this. So they had a significant expertise that they could share with us.

SB 7:52

Yeah. And there was the links also with other statutory organisation with councils, i'm thinking

P20 8:00

You know, we used established links. Sorry, if it's a bit of an echo there, we used to establish links with for example, the Macmillan Move More who are sitting within the Councils and then, because Jason White's team had significant and very strong links with the councils, we were able to tap into that as well. So it was brilliant partnership, really brilliant, across so many hospital services, community services, statutory, voluntary services, and the council's you know, and it's worked really, really well. We've been delighted with the success of it.

SB 8:37

Yeah. Yeah. Did enjoy your rule in it? Did you enjoy that?

P20 8:43

Yes, it was part of a number of plates that I was spinning. I'm sure I was delighted to be able to have you know when [CNS HNC] agreed to, to take over the leading of it, and she's done a marvellous job she really has.

SB 9:00

Yeah. What improvements do you think would enhance the sustainability of the cancer prehab programme? Thinking for the SE Trust? But also then when it's rolled out on that wider scale? What would what improvements could be made that would help?

P20 9:16

I'm not sure what you mean by improvements. So you're talking about how we sustain it going forward?

SB 9:26

Yeah. How you sustain it or are the parts of the project through thinking through planning and development. There's other Trusts going to be taking this on, trying to replicate this model, are there were the things that sort of you identified working through, we would do this differently or this could be done better or just any learning you can share, I suppose.

P20 9:50

Well, I suppose the learning would be to make sure you have the right parties at the table. And you know, it's thinking outside of hospital services, you know, so, you know, if you work on hospital services, you tend to think of just hospital services and not thinking of the other areas and directorates within your own trust even so like, that the linkages we made with the health and wellbeing team, I don't I can't remember if that is the correct name for their, their Directorate. But having those links was was invaluable. And I think from a sustainability point of view, you know, for this to make any real difference, and it will make a difference if it's properly invested in, but it needs the investment, and then you know, the longer term investment, because were you need to get to the, to the level three, you know, the specialist level of prehab, which is where you make the real, you can make a real difference. You need the specialist AHPs to be able to deliver that. And, you know, the CNS has to be able to deliver that. Yeah. And you would need the sustainability of the you know, the Macmillan move more programme.

SB 11:08

Yes, yeah, absolutely. Are there any other barriers you've kind of identified to the long term delivery of the programme. Beyond funding.

P20 11:22

While I suppose time, time and effort, you know, as usual, in Northern Ireland, we're doing things on top of the day job and don't have dedicated project support. So having that dedicated project support will be invaluable. And we're, we're, we can't wait to get the, you know, the project manager in so we have secured the funding through Macmillan for that 0.6 of an 8A project management support, to further expand and rollout and that will be invaluable, because it is challenging when you're juggling, what your day to day is plus pushing forward on all the new developments.

SB 12:02

Yes. And whilst there's money there for the project support and for that sort of the leadership there. What about in terms of, I think there's some challenges around allied health professional involvement.

P20 12:17

Yeah. So there's, there's money coming for the project management, as you've alluded to there. And as a clinical project manager, but to progress properly to all three levels, you need the AHP investment, and there isn't the funding common for that, at this point.

SB 12:39

Hopefully, hopefully, that changes in the future.

P20 12:43

Hopefully.

SB 12:45

I don't have any more questions. [PARTICIPANT NAME], is there anything you want to say that I haven't asked the question or any sort of final comments?

P20 12:55

Just really it was really innovative project that we've taken forward, and were delighted with, and we're delighted with the success of it. We also engaged with our planing and performance colleagues, so that we could get some robust and easily readable performance cards. No. So we used Outcomes Based Accountability, performance cards, which was able to show us what we achieved. And I suppose another aspect that was really, really important was agreeing the metrics at the outset. So what what was what was the measure of success? How were we determined on whether it was successful or not? And, and that's key learning so that you if not agreed before you start and then you move through, you might have to tweak and change but if you don't have that, agreed, you don't know the destination. So you could be aimlessly working on wasting people's time. So that was really important. And having the clinical buy in and engagement for that was key.

SB 14:04

You pulled together such a wide range of stakeholders, what do you think was the secret to getting everybody to want to work together and to move it forward at quite a pace?

P20 14:18

Just an agreed aim, you know, everybody signed up to it, and everybody knew that this was the best care that you could give to a patient in terms of getting them ready for either a significant surgery or significant treatment. So we didn't want to just focus on surgery either, you know that it was just surgery. So that's why we worked with lung so patients who were going for, for chemotherapy, which is quite harrowing for patients to deal with. So having them in the optimal health, physically and mentally. You know, using the prehab programme, bearing in mind that its a matter of weeks really before you start, but it helped you know what the feedback from patients was it really helped them mentally, psychologically to deal with what they had ahead of them.

SB 15:15

Yeah, no, its a fab project. Have you any final comments?

P20 15:22

No I don't think so.

SB 15:24

That's okay. I'm just gonna stop the recording then.

# P21

SB

Ah, okay. So, [PARTICIPANT NAME], what did you know about Cancer Rehabilitation before the programme started in South Eastern trust?

P21 0:02

The honest, nothing?

SB 0:15

Yeah, yeah, no, that's fair enough. And in terms of sort of what you've learned over the course of the programme being in place, what's your sort of thoughts on the importance of it, or do you think it's been a good intervention?

P21 0:33

Oh, definitely good intervention and, and also do all our patients.

Well, sort of, yeah, it's offer to all our patients who have a cancer, obviously, and all patients are encouraged to do it. And even depending on what level their exercise would be, you know, because I suppose it's not just about the exercise, it's about all the other parts of it as well. So I think it's great.

SB 0:57

Good, and do think it's an important intervention for patients?

P21 1:02

Definitely, particularly for ones that wouldn't be as fit or well, it's good to just try and get them obviously, to optimise their health beforehand. Just even getting in the right frame of mind, as opposed it just attacks every aspect of the person before the beginning for surgery. So it does help to build them up and just prepare them I suppose.

SB 1:25

Yeah, yeah, yeah. Okay. Um, what can you tell me your role in the cancer prehabilitation programme in SE Trust?

P21 1:34

So my role is just obviously, telling the patient about it. And even through consultations, where the consultants may be chatting that we would, and they were saying, you know, we need to get you a better fitter before surgery. We would definitely, I would definitely pop it in there. You know, and our consultants are quite aware of prehabilitation, as well. So it is something that's just talked about freely now. And my role is then obviously, getting all the credentials and doing the referral editing and prehabilitation.

SB 2:09

Okay, so do you complete the screening form?

P21 2:12

Yes.

SB 2:12

But yeah, all of those. Yeah. Okay. And can you tell me, were you involved in the planning stage of this project, or sort of the design of it?

P21 2:23

No, one of my colleagues was,

SB 2:27

okay, okay.

P21 2:27

So would have been hearing bits and pieces as those meetings were happening?

SB 2:32

Yeah. And were you able to kind of shape that even through your colleague, were able to, you know, do you think what's your conversations you guys had and fed through her to?

P21 2:42

I would say, so yes, we were talking and just talking in the offices general, if things would have, you know, thought things could be done a bit differently, we would have said, and then she would have highlighted that at the meetings.

SB 2:55

Fab, that's good. Did you, before you started and rolling out the programme, did you receive any specific training to roll this out?

P21 3:06

Probably just again, from our team colleague who had been doing it, so we were sort of like in the pilot of doing it. So we've kind of just, you know, gone through the, the screening tools,

SB 3:20

okay,

P21 3:20

Oh, we're giving all the information and all those sorts of things. So yes, we would have seen people to talk through that.

SB 3:27

Okay. Okay. Very good. Very good. Um, was that your other CNS colleague? Or was that other people that were talking to you's about those tools?

P21 3:37

I think it was probably there our CNS because she would have been probably the formal training and the implementation of it all. So then she was just forwarding that information on to us.

SB 3:48

Okay. And on reflection, [PARTICIPANT NAME], do you think you could have been any better prepared for this role? Would there be any information or training that you think would have helped you more?

P21 4:00

No, because I don't think the CNS that I work with is doing anything different to what I'm doing now so I suppose maybe just one wee thing. I don't think I realised the importance of filling in every single detail. You know, instead of, you know, if you were supposed to say, like the HNA, or something completed, you know, some of these things I wouldn't have filled in, and now I realise, you know, haven't had a wee bit of feedback, you need to fill in everything and just how important that is.

SB 4:30

Okay.

P21 4:30

So I think, probably being told them, you know, everything, whether you do it or not just even writing no, if you haven't done that, so that its completed.

SB 4:41

Yes, yes. Very good. Because yes, there's in terms of feedback that there's some issues then with some of the data.

P21 4:49

Yeah,

SB 4:49

So project that's very important, actually going forward then for roll out in other trusts, is communicated well with with the likes of yourself. That's a very important point. Thank you.

And just in terms of then the assessment and referral process for the cancer prehabilitation programme, when do you think is the best time to introduce this programme to patients?

P21 5:12

Well, I know from our point of view, it's when the results are being given. So initially at diagnosis that's what we're sort of talking to patients about it. And I think that's probably the optimum time because sometimes patients only have maybe a few weeks before they're coming in for surgery, although some of our rectal patients will have that bit longer, which works really well for them.

SB 5:34

Okay.

P21 5:35

Em, you know, so I think probably at diagnosis, although the patient can be overwhelmed with what has been told. But again, we're sending home the pack, you know, the resource pack and saying, "Look don't focus too much about that, just at the moment, you will get a phone call, and it'll be all explained better at that time."

SB 5:55

Yeah, I find it quite interesting to hear about how they're given that information at...it's been a few people have said they're given the information but told not to worry. And then that follow up phone call. So it's kind of staged, really,

P21 6:09

Yeah,

SB 6:09

It's been? Yeah. Quite a good model.

P21 6:13

Yes.

SB 6:14

Did you, so you've mentioned there, one of the challenges you have countered with the screening referral consent process and that you weren't sure. And I didn't realise the importance of filling out every aspect?

P21 6:26

Yeah.

SB 6:27

Were there any other challenges you encountered with the forms and screening?

P21 6:31

I just found the only, sometimes when I was trying to forward it on, it wasn't going through it kept in debug on the computer. So that was an issue. And it seems to have with me nearly all the time.

SB 6:44

Ok

P21 6:44

Yeah, so that has been an issue for me, particularly, now, sometimes we thought it was just one of the computer points. But then as you move about the computers, sometimes it happens with all of them. So I don't, that has been a bit of an issue. So then I've just have to email it on to what you call her, [PERFORMANCE MANAGEMENT LEAD] and then whoever the, like, Move More coordinator is or whatever in that area.

SB 7:07

Okay, okay. Right, lots of really good thing to fly, then. It's not something I've heard elsewhere. But that's something actually might ask now that you've flagged that.

P21 7:16

Yeah, that seems to have happened to me quite frequently.

SB 7:19

Yeah. Okay. And that would be quite frustrating and actually complicate the process for you.

P21 7:25

Yeah, that's not like just click it and that its away. It's, like, for flips sake, here we go again, you know, this and then who do I have to send that to now, you know, when you're looking at addresses and all those things, and then, if they, you know, I think for a wee while there was somebody out of post in one of the areas, so its then who do you send it to then but that doesn't happen, you know, all of the time. But,

SB 7:45

Yeah. But when you're when you're in a busy clinic, that can be very

P21 7:50

Yeah.

SB 7:50

..frustrating. Yeah, I fully get that.

P21 7:51

Yeah.

SB 7:51

I fully get that. Are there any components that you think of the screening tools that aren't necessary? So there was there was quite a few there, there was the ECOG, the distress thermometer, the Rockwood scale, looking at smoking, drinking? Was there any aspect you felt was not necessary?

P21 8:11

Not really. But i suppose the distress thermometer can be a bit you know, obviously, the patient is getting bad news at that time when you're filling it in initially. So that could be at that moment in time, it could be off the scale. So but that's not really how the patient is all the time. Yes but I know its important. But sometimes, you know. But yeah, the rest of other things, actually quite good, frailty score, all those things, and the ECOG, I suppose that just gives the person who's receiving the referral, a better picture of how well they are or what they can do themselves. So that's quite good. But again, that's a learning curve for me, because that wouldn't have been scores that we would have used or, you know, screening tools that we would have used all the time. So it's it is very good.

SB 9:01

Okay. And did you feel confident using those tools after you know, you had those conversations with your CNS? Did you feel okay,

P21 9:09

Yeah. And then we had got, like a, wee pack with, you know, your, your MUST, all those sorts of things and the fraility tool. So we've got that to refer to as we're going through the

SB 9:21

Okay,

P21 9:21

the process.

SB 9:23

Very good. Very good. Were there any helpful aspects to screening

P21 9:30

sorry,

SB 9:31

You disappeared there

P21 9:32

Someone is ringing me...

SB 9:34

Were there any helpful aspects to the referral and screening process that you found?

P21 9:40

I suppose, just seeing like sometimes seeing those pictures of the frailty score and the ECOG and those they were quite good, getting the MUST? Because yes, we would do height and weight on our patients but really what we have gone into doing the BMI and the MUST, all those things, you wouldn't really have always been doing those...So that would have been done on the ward but from our point of view, we wouldn't be have been doing those. So it's good to see, you know, just a flag up of a number and you know where that is on the chart and everything. So that was quite good.

SB 10:10

Yeah. And did you find it helpful that the, the sort of how the referral form was all sort of integrated into one was that helpful?

P21 10:21

It was yes. You would click obviously, which one you needed and what was applicable and what wasn't applicable?

SB 10:29

How, interested, did you find patients were in the programme?

P21 10:34

Probably at the time, they were just like, oh right, okay, we'll take this pack away. But after surgery and talking to them, a lot of them really found it very engaging and managed very well with it and thought it was very good.

SB 10:47

Okay, that's, that's good to hear that.

P21 10:49

We did have a patient, I'm sorry, I don't know if I can go off and tell you this. But we had a patient who had a bit of an alcohol problem. And so I don't know it was that through the prehab form, that this patient was admitted into [HOSPITAL NAME], and was detoxed before he got surgery. So two weeks before he was in he got detoxed, which meant that when he came in to have a surgery, he wasn't going to be going into DT's and all those things at that time. So I don't know was that through the form and seeing the alcohol liaison nurses or whatever but that was great.

SB 11:23

Yeah. Interesting. Yeah. It'd be nice to be able to tie that up and you know, find out if it was the same patient? Yeah. Very good. Did you find any resistance from any patients to the programme?

P21 11:37

Sometimes, some of the patients that would have been very fit, might not have wanted to be referred " Oh I'm out doing this and doing that" and they probably didn't really appreciate that, you know, it was everything. Holistic, I suppose the holisticness of it all? And maybe that's on my part, I should be explaining that a bit differently you know, that we're trying to target a lot of different areas within the patient. So I suppose those patients might have been had been resistant to, like the Move More and all those sorts of things. And

SB 12:11

yeah,

P21 12:11

Just a wee bit of physical exercise.

SB 12:14

Yeah.

P21 12:14

Because they thought they were a fit as could be.

SB 12:17

Yeah, yeah, and that's interesting to see what what could be done or, yeah. If you guys were clearer on what could be done, or what the outcomes are for those kinds of patients?

P21 12:29

Yeah,

SB 12:30

It would maybe help you to convince them. What impact did you think COVID had on the service provision of the Cancer Prehabilitation? And I suppose in terms of your end of things so your not actually providing the service I realise, but you are referring on. Did you see COVID having an impact at that point?

P21 12:52

I think that's all our patients. So I think if they were able for prehab I think we still did send them and still refer them on. So I don't know did it have a great impact.

SB 13:08

Did you, were you seeing the same amount of patients this past year, as you would have previously,

P21 13:15

We did, we did get all our patient all our cancer patients through, and actually patients from other trusts. So from that point of view, because we would have patients from Derry and up in that direction. So from my point of view, those patients were sort of missing out a wee bit. Because the prehab wasn't available there,

SB 13:33

okay. Yeah.

P21 13:35

I don't think they have that in place, or there's nowhere for us to refer those patients to. So you felt oh they are missing out a wee bit, as we can't offer them the same care that we're offering patients that are closer to home.

SB 13:46

Yes. And that's actually quite a challenge for the rollout of a programme like that in one area.

P21 13:51

Yeah,

SB 13:52

that that actually issue has come up a few times, in terms of inconsistencies of service provisions.

P21 14:00

And sometimes you feel those patients could really do with it, because they might have been ones have maybe had an alcohol problem or could have done with getting a wee bit more physically active or, you know, all those sorts of things. So

SB 14:11

yeah,

P21 14:12

wee get an inconsistency. Just.

SB 14:14

Mm hmm. Yeah. And and then you find that difficult, therefore, as, the specialist nurse talking to those patients when you know, they could benefit from something in your can't provide it.

P21 14:27

Yeah, because sometimes it would be out of me, you know, we've got prehab and we can refer you for that and then all right, rewind, you live, so sorry, that's not available to you. Yeah.

SB 14:41

What benefits do you think you've experienced in terms of your professional development and practice from being involved in the cancer prehabilitation programme?

P21 14:51

I suppose just knowing that those things are out there, being able to refer our patients to you know you feel you are prepping them better and you're going to have a healthier patient, hopefully coming into hospital. And, and just as increase my knowledge, really, I suppose of that all these things that we work on in prehab can actually improve patient outcomes afterwards.

SB 15:16

Yeah, yeah. I suppose as well, you have mentioned there in terms of development skills around using those tools you've mentioned earlier.

P21 15:25

Yeah, yeah.

SB 15:27

And and what was the discussions and team meetings helpful? Talking with your colleagues was, how did how did you find that?

P21 15:40

Very useful, and we were able to say about, you know, patients that we've had, and, you know, just just talk around that, and that would be really good for that patient, or they've done really well. And that's what's good about, you know, just sharing that, that knowledge, suppose with everybody, and just encouraging everybody to be on board within our team, you know,

SB 16:01

yeah, yeah.

P21 16:02

Yeah.

SB 16:03

Have you enjoy being part of the programme?

P21 16:05

I have, yes.

SB 16:06

Good, good.

P21 16:08

Yeah.

SB 16:09

What improvements do you think could enhance the sustainability of the cancer prehab programme?

P21 16:17

I suppose having it rolled out in other areas. And maybe just even a wee educational update every once in a while, if there's anything has changed within prehab, or if there is anything new that we can offer the patient? I'm sure we would hear that. But it'd be good just to have, just to be update every now and again as to how things are going.

SB 16:43

And can you envisage any barriers to the long-term delivery of the cancer prehab programme?

P21 16:52

Sorry. I suppose it's just everybody's commitment to do it. But I suppose if we get it into normal practice, then that would be great. And I think within our team, it is normal practice now, anybody who has colorectal cancer, yes, they're getting referred to prehabilitation. So hopefully, there won't be any barriers, but it's just every, you know, you don't want anybody to, to slack or to, and just to make sure that the resources are still there. I suppose that could be a barrier if there's no funding for it. And

SB 17:27

yeah, yeah. And what has you know, you've talked there a wee bit about, and the other team members, what has the kind of feeling been within the team about the prehab? programme? Is there? Is there positive vibe? Are people really on board with that?

P21 17:42

Yes, I think they are. They're all pretty on board. But it is a bit sort of time consuming at times. And it's just another nice thing that we have to do following our results clinic. But I suppose we do all the other referrals. So why not just do this other one if this is going to benefit the patient?

SB 18:01

Yeah, yeah. And that's yeah, I mean, if you've kept coming back to that about benefiting the patient, and

P21 18:06

yeah,

SB 18:07

that is important to you and to your job?

P21 18:11

Oh, definitely. Definitely. Because you want them to be, especially with ours, because we get the prehabilitation. And then that patients in hospitals, and they have an enhanced recovery afterwards, you want the patients to be just as well as possible, as quickly as possible and just keep moving.

SB 18:25

Yeah, yeah. And I don't have any more questions for you, [PARTICIPANT NAME]. But is there anything else you want to say? Or anything you were thinking I want to convey that I haven't had the opportunity to... speak freely.

P21 18:41

I don't think so...I think it's a great thing particularly that patient who had had their detox beforehand, I'm not sure if it was prehabilitation that has got that done for them. But that would be good. If that was something that cause more and more patients or have drink problems. smoked all the time. And, it just be good to

SB 19:05

see previously a patient that had a drink problem that was diagnosed with colorectal cancer, what would have happened? Would there have been a referral?

P21 19:17

Probably not

SB 19:18

Okay,

P21 19:18

you know, it would have just been okay, well, you know, they would maybe have been seen by them while they were in hospital, the substance misuse team, but it wouldn't probably have been dealt with prior.

SB 19:30

Okay.

P21 19:31

Yeah. I don't think I ever made a referral beforehand, although that would have been good. But, you know, I suppose the prehab just just brings it to light everything that you know, and because it is on there as a screening tool. You're doing it.

SB 19:49

Yes. And you're identifying the issue.

P21 19:50

Yeah.

SB 19:51

Yeah.

P21 19:51

Yeah, your identifying the issue and then highlighted and hopefully referrals on them.

SB 19:57

Yeah, yeah. No, that's, yeah, that's very interesting and then I'm glad to share that that case there. Thank you so much. Have you anything else or will I stop the recording?

P21 20:09

No no you can go ahead and stop then, I think that's us.

SB 20:12

Okay

# P22

SB 0:03

Okay, [PARTICIPANT NAME] before the Cancer Rehabilitation started, at SE Trust, what did you know about cancer prehabilitation?

P22 0:12

To be honest, I didn't know an awful lot at all. So it was very, very, very new to me, and obviously, when my you know, we're, you know, obviously working within sort of pelvic health, I knew it was probably an area that we could be involved in. But I really didn't have very much knowledge about even the surgery that is involved with cancers, so a big learning curve, really, for myself. And I had no idea what the prehab involved, sort of read wee bits and pieces regarding prehab in different areas, but very, very little knowledge.

SB 0:57

And what are your thoughts on now?

P22 1:01

I think it is a great service. I definitely think that it definitely would be an area that I think should be developed even more. I think just given that, that overall sort of that multidisciplinary teams support to an individual who is facing, maybe surgery, there's so many different areas and probably different questions that they have and the support from a network of, of different professionals, I think is his ideal.

SB 1:41

Yeah, yeah. And how important do you think it is to patient outcomes engaging them prior to surgery?

P22 1:50

Very important, because I think a lot. It's like everything else, I think if a patient is well informed, and educated on, you know, what the process is, and the importance of, of what you are trying to achieve, if the patient has that understanding, then I think you have a better buy in from the individual.

SB 2:20

So can you tell me [PARTICIPANT NAME] about your involvement in the cancer prehabilitation programme? Thinking were you involved in the early days and sort of the planning and creation of it? And then what's your role in sort of the implementation aspect of it?

P22 2:36

Well, I was invited to the the working group, and as I said, sort of previously, I sort of went in, you know, very open minded, I really wasn't really sure what, what this was going to entail. So from a multidisciplinary team, point of view, it was it was great to get everybody round the table. And even from sort of the outside agencies that are going to be involved. So we attended, I can't remember what the would have been maybe monthly meetings and now, it was time consuming, you know, trying to find that in, in a busy, in a busy diary was was was difficult. But I valued the importance of it. And so really, from our point of view, you know, the referral really just come in, or is just coming in from sort of whether it be the consultant or the nurse involved. And really, then we just see them as early on in the process as possible. And I think sometimes maybe just within the colorectal aspect of it, it's sometimes difficult, maybe we don't actually have enough time to prehab prior to their surgery,

SB 4:08

okay,

P22 4:09

Okay so, and that that time period, we may not have enough time, but we will always follow them up maybe post-surgery as well. And just to make sure that, you know, they're getting the outcomes that they they require.

SB 4:25

Yes. So do you think um, first of all, just a question, based on another interview I've done recently. Is there a difference between in terms of colorectal patients in terms of the type of surgery they're having or the placement of the tumour? And are you getting more time with some patients than other patients?

P22 4:48

And I can't, I should have actually looked at not to share is the number of referrals that we have actually received, it probably hasn't been as many as what we had anticipated. And and some ways, you know, that's that's, you know, actually quite good because then obviously, you know, it's not affecting people, you know, in terms of the cancer diagnosis. But sorry, I forgot the question that you were

SB 5:15

just sort of like, you know, based on where the tumour is there some patients are getting more time than others.

P22 5:22

Not No, not necessarily. Not necessarily. But what I probably would, you know, think following all of this about, regardless of where the tumour site is, I do believe that all patients could benefit from from prehab. And, you know, obviously, from a colorectal point of view, it is going to have consequences on to the pelvic floor muscle function. And so I believe that this should be opened up to all colorectal surgeries. An even more developing on from that, I think, from a uro-gynae point of view, if we're looking at other cancers, all pelvic cancers, I think require prehab. Because I do believe that the more informed patient is, and the more support network and where and how well, they are informed and educated. I think you have a better buy in from them. And and you'll get better adherence from an exercise programme.

SB 6:27

Yes. So do you think from a functional point of view, like from the little time that you have with them? Do you think there's any gains being made from the prehab programme from that point of view in that short window that you have? Or do you think it's more of like, you're getting better adherence, you're getting better by and you have a better relationship with the patients would help afterwards? Or do you think there are some wins to be made in that early?

P22 6:52

I think there's definitely wins to be made. And I think just even touching base with, you know, having that patient knowing that there is somebody that they can contact should there be a problem. I think there is definitely room for for that contact prehab. Even if it's just a very short window, even just to say, look, I'm here, this is my job. And we may not have enough time to cover everything or get you to your full potential prehab. But this would be our, our goals on an overall basis. Okay, so yeah, definitely starting to happen starting those conversations. Exactly. Yeah.

SB 7:46

And so what in terms of whenever patients are referred to you, what's their kind of response been like, in terms of engagement?

P22 7:55

And they've been very, anybody that I have had, they've been very open? Yeah, do buy in to it.

SB 8:05

And have you had any of you had any resistance?

P22 8:10

No. No resistance.

SB 8:12

Fabulous. Great.

P22 8:14

Yeah. Which was just great.

SB 8:15

Yeah, yeah. Have you encountered any issues with the referral process with you receiving referrals or communication, any issues with any of that?

P22 8:28

No, I haven't done I suppose maybe, maybe the referrals and this isn't anything to do with the system, it's just the way it is that maybe that you don't, you have such a short window maybe at times, you know, from getting that referral, you don't maybe have that 12-week window that maybe you thought that you had from a prehab point of view. So I suppose, but that's not a system. There's no, you know, in terms of communication, or referral that is just maybe what is happening at that time and time is of the essence in terms from a surgery point of view and that we have no control over that.

SB 9:10

Yeah, I know. I'm hearing in some cases, that very short window between diagnosis and surgery, which I'm sure for the patients is great.

P22 9:18

Exactly, exactly. So that's not something we have really any control over.

SB 9:24

Were there any helpful aspects you find to the referral process?

P22 9:31

I think the fact that it's electronic, is great, and it's all standardised is Yeah, most definitely. I think that's the biggest benefit is that standardisation and the electronic referral.

SB 9:49

And as they're everything on it that you need, there's no gaps and information.

P22 9:54

No, I haven't come across sort of any any gaps. I suppose if there is anything that we are maybe looking to query you know, we have access to sort of ECR, if we need to sort of fill in the gaps, which, again, is is great. I've never had to sort of make contact with anybody to get further information.

SB 10:19

Okay,

P22 10:20

I've made a thought to maybe on one occasion, just to, to get a time frame for maybe a reversal, but that's post surgery.

SB 10:29

Okay,

P22 10:30

so,

SB 10:31

okay. How's COVID-19 had any impact on the programme from your point of view?

P22 10:40

I don't think so. I, you know, from a service point of view, we have kept the service going as much as possible. So, I don't think there's been any sort of delay, you know, from, from a physio pelvic health point of view.

SB 10:57

You were still seeing patients?

P22 10:59

We were still Yeah, we were still making contact. And, you know, maybe in those early stages, gosh seems so long ago now, but we, we kept the waiting list going by even just doing virtual consultations. So we just kept going. So I don't think there has been an impact on on the prehab service.

SB 11:23

Okay,

P22 11:23

as the referrals come in, we acted on them.

SB 11:28

In terms of and then the planning and development, was there any, a lot of that was on Zoom? Was that helpful? Or maybe difficult with building relationships with people? What was your kind of feeling?

P22 11:43

I think initially, you know, we had sort of build up, you know, face to face rapport, you know, pre sort of COVID. You know, so there were quite a few familiar faces, but I think it's like everything, you know, when it came to zoom, it was maybe sometimes difficult to voice your opinion, you know, get get the opportunity to maybe to voice your opinion, because there's maybe so many people, you know, on the call at that time, and I think it's like everything, I think zoom has had its, its its benefits, but it also has its drawbacks, in that sort of personal face to face and sort of getting round a table and discussing things as opposed to virtual, but given the circumstances we just pushed on.

SB 12:36

Yeah. And as fab what yous have achieved, in spite of all of that.

P22 12:41

Yeah.

SB 12:42

What benefits have you experienced in terms of your professional development practice from being involved in the cancer prehab programme?

P22 12:49

I think from collaboration working across multidisciplinary teams, has been fantastic. Whether that be inside the trust and outside agencies, I think that collaboration has been great. And I think that has, has maybe strengthened even relationships, you know, across across the different multidisciplinary teams and probably opened up maybe other maybe other opportunities where you get to know what other services actually do. And so it creates sort of more opportunities, not just sort of, from from a prehab point of view, but if we are thinking of maybe the colorectal that we had been involved in, just any colorectal patient, regardless of cancer diagnosis. So I think it is it's definitely strengthened from from that point of view, multidisciplinary team meeting, and even just going on what other outside agencies are doing? I think it's been great, I think been great.

SB 13:59

And so your, your role is physio, were you, did you have any linkages with the Move More coordinators, for example, did you have any communication or advise them in terms of exercise to encourage or

P22 14:14

We did in the initial planning that their booklet that they developed, we sort of give a wee bit of feedback on on maybe some of the exercises that we thought maybe, you know, maybe slightly modify them, because of x, y and Z. So yes, we did, we gave a wee bit of feedback in terms of that.

SB 14:37

okay, and that was modifying it from was that from the Manchester model or modifying it from from what?

P22 14:46

I can't remember off the top of my head, but I just remember some of the exercises coming through and thinking maybe oh at an early stage that maybe may not be appropriate, and I suppose me maybe I'm thinking it from, you know, a core, you know, a pelvic floor point of view, and maybe the, the increase in abdominal pressure maybe might impact on on sort of the pelvic floor. So there's a bit of feedback and basically on that. I wasn't involved in sort of from the, the general physiotherapy point of view. So I was primarily just focused on on on pelvic floor rehab.

SB 15:25

Yeah. Okay. I've enjoyed being part of the

P22 15:29

I have, I have, I'm actually one of my colleagues now sort of am while I was involved in sort of that initial sort of sort of passed that over, well we both work on it but you know, for any sort of future meetings, my colleague will sort of, maybe take that, that on board now that it's sort of up and running. But I would I would really love to see it opened up to other cancers, I think it's vitally important.

SB 15:59

Yeah. Yeah. And what improvements Am I suppose that that that was and that would be an improvement. And one of those do you think could enhance the sustainability of the prehab programme?

P22 16:15

I suppose Yes, it's, it's opening up on to other cancers, and I suppose it's like everything else, it's workforce. It's, it's having the people on on the ground, that actually can carry it out. And when I know, certainly within our sort of small team, because we are such a specialist area within physiotherapy that it's sometimes hard to actually get the staff with the specialist skills. So I suppose from our point of view, its workforce planning that we need to look at and to make it sustainable. And more funding.

SB 16:57

Yeah. And were yous able to cope with the referrals coming in? I know you said, they were less than expected, but were you able to cope as a team?

P22 17:06

We did, we did prioritise because we valued the project, the project and so we, we made it work. And the staff that we do have, were more than happy to take on on board those, those patients, so we made it work. We made it work because we were quite passionate that this is something that needed to work and the patients deserved it.

SB 17:36

Yeah. And can you think of anything, if this has been rolled out to other trusts, things that might need to be in place to ensure that this is going to be ... so workforce being key...

P22 17:48

Key, and I suppose it's like everything else that you know, everybody is looking for, for funding, but with like the fund and you don't have the workforce, you're not going to be able to deliver everything that you would would like. So I definitely would say we need the funding, and we need the workforce behind to make it sustainable in the future.

SB 18:12

Are they there was something he touched on earlier, just about the timeframe in terms of from diagnosis to surgery? Is that an area for improvement in terms of maybe communication or does prehab need to be prioritised more? Or

P22 18:35

I suppose maybe that's something where, you know, from, you know, on a different level, you know, obviously, that's to do with sort of, you know, how quickly the patients are being sort of diagnosed and picked up, I suppose maybe another area of improvement would be that, you know, the thinking of ourselves that maybe, you know, physio are involved within those clinics. So that may be maybe like a one stop shop, where you know, that the patient actually has seen the consultant that day, the physio, is there, at clinic to meet them and greet them. So that would speed up the whole process as well. So yes, in an ideal world, that would be great. We're working actually out of the same clinic and we can see the patient on the same day.

Yeah, yeah, that's a really, really good suggestion actually to include. I don't have any more questions for you Roisin. Is there anything else you wanted to say about the programme where you kind of thought that you would have brought up today but I haven't asked?

No, I don't think so. No. As I say, I've I've really, really enjoyed it. I think there is definitely you know, plenty of scope for further, further developments. And as i say it's just really just having the manpower and the funding behind us to take it to take it forward and develop it into other areas. Yeah.

SB 20:11

Yeah. And thank you. Thank you so much. I just stopped the recording.

P22 20:16

Okay.

# P23

SB 0:02

Perfect. [Participant name], before the cancer prehabilitation programme started at SE Trust, what did you know about cancer prehabilitation?

P23 0:12

So I had the opportunity, when I just first started my post think it was two or three months in to attend a conference on prehabilitation, through Macmillan. So I had the opportunity there to hear from Macmillan professionals from kind of the Greater UK, about their services. So I was able to get it's kind of quite a bit of in-depth information about it at that stage. And then really, it was kind of not introduced into the trust until I can't even remember when it was now, but I didn't really hear about it for probably about a year from then until [CNS HNC] and [AD] started that kind of the process of integrating it into getting it into SE Trust.

SB 1:05

Right, very good, and so... [cough] sorry. And whenever you first heard about it did that kind of spark an interest, or what was your thinking back then

P23 1:17

I definitely did spark an interest, what I would say is, I feel that we were doing a lot of the principles of prehabilitation, but not in a formal pathway. So it was kind of, we have the professionals there, but it was more patient, kind of dependent, whether we got that that kind of process in in place. But it kind of just brought together that that formal pathway and the clear measurements of where the patient was starting off, and where they were prior to surgery, and then afterwards as well.

SB 1:55

Okay, and what's your kind of thoughts on cancer prehabilitation, about the value of it?

P23 2:02

I think, is really valuable. And certainly with myself, I would probably do the majority of the the One Stop prehabilitation clinic appointments within head and neck. And I think it just gives patients that opportunity, what from my perspective, from a CNS perspective, that mental preparation of coming into hospital and even just asking them those practical questions about what the ward looks like and what to bring into hospital, and then into the greater detail of the more in-depth information about the surgery and the short term and long term side effects of that. But certainly from a kind of a physical point of view as well, the introduction of the Macmillan move more dietetics. And we did have one point speech language therapists, but unfortunately, they weren't able to remain on but just that their input was so valuable, and it just let patients knew where they were at what areas could potentially be improved on and, again, just that that preparation prior to coming into hospital.

SB 3:07

Yep. And you were saying they're just about how those conversations you were having was helping to prepare a person coming into hospital would not have been a different process than what would have happened.

P23 3:20

Not entirely, I think it does, probably highlights the need more, I would say there, we would have done it in a probably a more ad hoc basis with patients. So it just meant that it wasn't a set clinic, not a set clinic time, then [CNS HNC] and I would be working those appointments within our diaries. So a lot more ad hoc, they probably would have been given the same level of information, the same written information, but then there wasn't that incorporation of the other AHPS as well. So there wouldn't have been the necessary the Macmillan Move More or the dietetics. It would have just been in a nurse led appointment. So we probably would have done a bit more of that kind of bespoke information with patients then, and then generated referrals if necessary. They weren't automatic referrals. But really, the specialist nature of all the operations, they really should be seen by a dietician before their operations.

SB 4:26

Okay, okay. So can you tell me a little bit more about your role in the cancer prehabilitation programme and thinking right from the early days, the planning, what sort of involvement have you had right throughout?

P23 4:41

Yeah, so I definitely, [CNS HNC] obviously is taking the lead within cancer services, but also within head and neck as well. So there was a time where I did attend some of the early initial planning meetings with [AD] and colorectal and lung. But [CNS HNC] as I said really probably did take the lead, I was part of kind of the groups where we met with the AHPs such as physio, speech and language, substance misuse to kind of work on those initial measurements that need to be taken the referral criteria, and kind of the who should be involved in prehabilitation. But then really since then, a [CNS HNC] did take the lead, and then probably just more in team discussions about the practicality such as, where the clinic would take place, at what time and what day suited service.

SB 5:47

Yeah, so I'm hearing that from some of the other tumour groups as well, they'll have been a lead, a person that connects with the working group. And, and there's other been others involved in task and finish groups, etc. But, and, and, and kind of how that works. But in terms of you and [CNS HNC], or indeed if there's any other CNS, how did that link work with [CNS HNC] being the lead, and we're able to feed in your your views, your comments, and share those or how did, were able to, do you think that you could inform and shape the project with like, being right at the table all the time?

P23 6:24

Absolutely. You [CNS HNC], I think, you know, before kind of quite a few of the meetings, we would probably had probably quite informal discussions about our thoughts and feelings. But no, I felt even though it wasn't there physically at those meetings, I was able to get any views or thoughts across through chair. Yeah.

SB 6:44

And were you in receipt of any training to roll this out in terms of any of the measurement tools or any aspect?

P23 6:54

No, it not necessarily the ones, probably not formally, but we did discuss them with the relevant other professionals that would be involved. And I think probably just previously, using the majority of the tools within kind of my clinical nursing role there probably wasn't a huge need for that. And then we've had great support through [Performance Management Lead], who was part of the getting through the referrals. And, you know, she's been extremely helpful if there has been any queries in terms of completing the referral. Okay, okay. I'm on reflection, is there any way that you think you could have been better prepared for your role in the programme?

SB 7:40

I don't think there was necessarily anything prepared that could prepare me to improve. But certainly, it was just, obviously a new service, a new ... my, my head words aren't coming out

P23 7:59

That happens me [laughs].

SB 8:02

The process of actually just incorporated into the service, I think it was just continually reminding yourself about it, just because it was was a new service and making patients aware of it and even simple things like part of the referral form is getting the patient's email address. And you know, for that, us that wouldn't be necessarily something that we would need to get from them prior to the prehabilitation. So it was just kind of reminding myself about it. Probably talking about it, if not at diagnosis at that first initial contact, an appropriate time for the patient, and then processing that referral. And yeah, so not necessarily better prepared. I think it was just getting used to a new service.

P23 8:50

Yes. And so there was maybe a process of embedding that into your practice. The normal service that we yeah, yes, yeah.

SB 8:58

Okay. Yeah, sorry. There's a wee bit of a jump there between you and me on the zoom. You mentioned there about the timing of introducing the cancer prehabilitation programme. So some of the tumour groups I'm talking to there are saying its at diagnosis, but I think it is slightly different in head and neck, could you tell me about that?

P23 9:20

So, I think is, again, just patient dependent. Sometimes patients will come with their staging and imaging in hand, meaning that we will have be able to have kind of a more in-depth treatment plan discussion at that initial diagnosis, and that will be something that I would feel that probably a referral would be appropriate. I really feel it's as most appropriate when that clear treatment plan discussion has been made. And that can be you know, that's when we within quite a short period of time, probably one to two weeks following diagnosis. So it's but it's not. Because there are the patients that come and then clinically, things will change quite quickly, meaning that they may not get treatment as initially thought, yeah.

SB 10:14

Okay, right. So yes. So there's no need to wait to know the treatment and then its next steps. And I suppose then theres the balance if that conversation. I'm just thinking in my head. So give me your viewpoint. If they had that conversation, even if they didn't know, their treatment plan, but they had their diagnosis, if they had the, the prehab conversation earlier, would there have been more of a window for prehabilitation?

P23 10:46

I think for our patients, and I'm just thinking kind of in terms of, yes, there is those opportunities where we maybe know that surgery is indicated, but the level of surgery isn't determined yet. So the the recovery from that particular operation would vary from dependent on the level. And that would be the same for our dietitian as well, you know that the level of information that you would give would change depending on the level of surgery. So I feel at that appointment is that the One Stop clinic appointment is probably most appropriate when that treatment plan decision can be made, but in terms of things like the Macmillan move more, but there's really nothing stopping them getting on with that, you know, from from diagnosis, probably.

SB 11:37

Yeah. Okay. So I can see there, there's the need to wait, but advantages with pushing forward with elements of the programme.

P23 11:47

Yes, that's what I would say and things like, yeah, with the Move More, I think that gives them a little bit of control as well, you know, it's that kind of, you know, they're able to do some exercises at home, because that is probably one of the key questions that we get asked that diagnosis, what can I be doing to help this process and things like that, to prepare them even further anaesthetic appointment, as you say, it's just some elements I think, are generic and probably can can start from diagnosis.

SB 12:20

But that's not how it currently the model currently works. It is everything starts at...

P23 12:26

Yes. Yeah.

SB 12:27

Later points. Okay. Yeah, okay, that, right, that's an interesting, have you encountered any challenges with this screening, referral and consent process in terms of, and how the referrals work or the screening tools you're using or anything?

P23 12:46

I think not I haven't had any issues, per se. The one thing that I did have to get clarified was about the emotional well being. So I had a patient who was scoring highly on the distress thermometer. So it was that was that automatic referral to the psychologist assistant. And, but I was able to contact the service, because I wasn't really sure if a psychologist assistant was appropriate for this particular gentleman, because I think his stress was so extreme, that it needed to be kind of like through the GP, mental health services as such. So I was able to contact the department and speak to I forget his name, but is really, really helpful. And he was able to kind of clarify me that he would deal with kind of mild to moderate distress, that that extreme distress does need to go kind of above the prehabilitation programme. Yeah, so that I find that really helpful. Because within head and neck there is, you know, a fair amount going of people who would have either previous mental health conditions or kind of acute because of their diagnosis. So that that was helpful for me.

SB 14:05

Okay, and sorry, I jumped in there. But where did that? Did you have to go then via the GP route for that?

P23 14:12

Yeah. Yeah. We're, unfortunately, as nurses not able to make referrals to mental health community mental health teams ourselves, it has to be through really the GP. So that's, you know, that's a little bit difficult, because you feel kind of like you're the middleman. And I think when you contact the GPs and say that they think, well, why aren't you making that referral, but unfortunately, seeking advice from mental health nurses, for that particular patient within SE Trust, they said, "No, it has to be the GP."

SB 14:47

Yeah. Yeah. That's good to have that clarity. Anyway, were there any technical difficulties you experienced at all?

P23 14:55

I don't, I don't think I had any technical difficulties. I think just there was on a number of occasions where i probably sent through a previous version of the form that [PERFORMANCE TEAM MANAGER] very quickly got back to me and said ........we have forms that can be filled in, because really how I'm filling it out is I'm going back to the original email that [PERFORMANCE TEAM MANAGER] has sent out with the kind of the link to the referral, and make that email ......[unclear word] So I'm able to kind of just pick up on it quite quickly, but it probably would be quite helpful to have you on the internet, like a form that we could just go in and hit like prehabilitation referral. Yeah, just for that kind of quicker access to that form.

SB 15:51

Yes, that's a really good point. And, and only the current form was live then on that.

P23 15:58

Yeah, yeah. No, that's, that's, yeah, very, very good point. Were there any helpful aspects to the form?

SB 16:08

I think it's really good form in the depth that it goes to, and kind of the, you know, kind of prompts you to even if you've forgotten to do something, like, for example, that the email address, and I've started the referral, and then forgotten to ask patient for the email address, and I've been able to then contact the patient just say, you know, can I get it off you? And, you know, it's really good insights, I think it's a good holistic kind of assessment. You know, it goes through the Distress thermometer, but also, you know, the nutritional aspect of things for not, we're a one stop clinic, but the other tumour groups and the smoking and alcohol as well, you know, I think it is

P23 16:54

How interested have you found patients in the Cancer prehabilitation?

SB 16:59

So probably mixed, I would say a lot of people like the opportunity to have that appointment with the CNS, probably away from the consultants that are able to ask questions, and they come prepared with questions, which again, is really helpful. Some of their patients, if they have had quite a number of appointments already with the team, they feel like this, it's just an additional kind of burden, another appointment. There has been I think, one patient recently did decline because of that reason, they were seen on the Friday by the consultant and said, "No, I've been here enough," which which, again, is absolutely fair, fair. But it's, I think the way that you sell the appointment, almost that it is part of their their treatment package as well. But no, the majority people come, they normally come with their next of kin or a family friend, and gives them the opportunity as well to ask questions. And there's the option as well to speak with [CNS HNC] and I would try to incorporate as well, sometimes speaking to a previous patients within that time. And there really isn't, because there is the two slots. I think that's very appropriate, because it gives us that you know that theres not a set time that the appointment needs to finish at, there has been a patient with quite additional information needs, and have found the appointments really worthwhile.

P23 18:40

That's good. That's good. That's interesting as well, that you say with the sort of the diagnosis and your appointment being separated, those patients are coming to you with questions they've had time to formulate.

SB 18:52

Yeah, yeah. I think it's even just appointments like questions. You know, I think for our patients, particularly with getting things like the tracheostomy, knowing what to bring into hospital and visiting is always a very kind of questions thats asked quite frequently. And you know, that that just really recovery period whilst they're in hospital, it gives them the opportunity to ask really if there is any questions that they need about that?

P23 19:23

And how whenever you raise the cancer prehabilitation programme, what is the reaction of relatives? Or the whoever comes with them?

SB 19:33

Yeah, I always tried it. I kind of start off by saying, you potentially heard about it, you know, recovering from a treatment or rehabilitation. But we have introduced something called prehabilitation where we are, it's a service that and you know, people are normally quite intrigued in the sense that they have, again, it's that question, What can I do to prepare for my cancer treatment in it and a lot of our patients that would be in around nutrition, physical activity and that sort of thing. So they are intrigued, some people will probably think, you know, what if this girl going on about, but I think as the process was only under that that greater understanding of the need for it prior to treatment,

P23 20:22

and that's the relatives, or are you meaning or the patients?

SB 20:27

I would say both? I would say both. There's Yeah, it's just, I think, an additional opportunity to be engaged with the team as well. They certainly, you know, they would [CNS HNC] or I at diagnosis, and it's just an additional appointment that they maybe didn't necessarily know about, or think they would have. And it just gives them the opportunity to have that one on one time. Really.

P23 20:56

Yeah. And what sort of, have you noticed any impact that COVID-19 has had on the prehabilitation programme.

SB 21:05

Unfortunately, probably just in terms of number of referrals, that we are receiving more locally advanced disease that unfortunately, people aren't, weren't and aren't getting to surgery, like they would prior to go with there was a time there. And I think there was some query kind of into why the number of referrals had reduced, but there did appear to be a period of time maybe a few weeks that nearly all the patients that we were seeing, were really going straight to palliative care, unfortunately. And those referrals weren't being processed. So that that's what I would say, it's probably something that will be ongoing for some time, unfortunately.

P23 21:52

Yeah. And what about in terms of the planning stage, what impacted COVID-19 on the planning I know like, it sort of forced a lot of people, a lot of this was designed talking to other people on Zoom, would there have been benefits with that or not?

SB 22:12

I think our department has actually been very fortunate that we have continued to see patients and especially new patients through the pandemic, with precautions in place, and that has included our prehabilitation like our one stop prehabilitation clinic. We're very fortunate to have had the outpatient department downstairs, it's quite got quite a lot of space, even compared to our clinic, within the Oral Surgery Department. So we were still able to really see patients face to face, if they were wanting to speak to a previous patient, we probably do that virtually in terms of the patient with the upcoming surgery would be with ourselves in the Ulster and the previous patient would be online. I have actually done one prehabilitation appointment virtually, at the request of the patient, and for childcare reasons. And there really wasn't any, I would say huge deficit in the kind of I was able to kind of discuss all the information needed. And then [name] our dietician was able to jump on after me and do her assessment on zoom as well. So it didn't make and it meant it was more relaxed, because she knew that she wasn't having to travel in and rush back to collect children and things. So it works quite well.

P23 23:33

Yeah. And that's it was COVID-19 has sort of driven that virtual option. Yes. Sure. That's fab when there's still things going on as well for people the flexibility offered. In terms of the the planning with the planning stage and the meetings on Zoom did that mean you were more able to attend? Or do you think it made it more difficult to design because you weren't meeting people, you weren't getting some rapport with people? Or how did you find out?

SB 24:10

Is that thing of prehabilitation that the service, or?

P23 24:15

Yeah.

SB 24:16

No, I don't Well, personally, again, I probably I feel that some of the meetings did place take place face to face. The ones I certainly attended did within the kind rooms that we were able to safely so socially distance and appropriate members from teams were there.

P23 24:37

But I can't really comment on probably using zoom for that that Yeah.

SB 24:45

Okay, no, no worries. What benefits have you experienced in terms of your professional development and practice from being involved in the programme?

P23 24:54

Um, I think it's just been, you know, really great to see the incorporation of, you know, for me being still within what I'm two probably nearly three years within post and that kind of leadership from [CNS HNC] to witness that and be fortunate enough to work alongside [CNS HNC] to see how she managed that at such a high level, like Department of Health and things like that has been really inspiring to me, first of all, but learning on the different kind of stakeholders that you need to incorporate a new service and all the different things that you have to think about even you know, the confidentiality of the referral form, and the external kind of people that were involved in the internal, you know, to me, just showed that actually, it does take a lot of hard work and effort from lots of different people to get this one kind of service in place. And then seeing it just flourish, I think, you know, certainly from my perspective, personally, that that one-on-one time with the patients, just again, just reiterate the, the my love for the job. And the reason that why we do it, which I think is really important in these jobs, especially because they are so difficult at times, just that that positive reinforcement to patients and seeing patients get to surgery as well, during the pandemic as well has been really rewarding as well, because I know that we've been very fortunate compared to other trusts in terms of our theatre capacity. So yeah,

SB 26:42

okay. Okay. And what about working with others? How did you? Do you think that that was you know benefit?

P23 26:53

Yeah, I think I know, within SE Trust especially within cancer services, we are very fortunate to have so many from a CNS point of view, you know, those [CNS LUNG] from lung and [CNS CRC], and just again, to hear about their their wealth of experience and their thoughts and hearing about other services and how they currently run, again, is extremely worthwhile. I know that kind of any meetings I attended, everybody was very, very courteous and listened and took on everybody's points of view, very professionally. And again, it's just been been great to hear about the other teams as well and how they're getting on. And again, just, as I said to [Performance Management Lead], again, with that referral process, and extremely approachable and contactable, if needed. And I think just to be able to this probably relates back to the previous question, but to implement that change during a global pandemic is something it's actually be really proud of ... And they're working to even despite kind of adversity kind of, to develop and optimise patient care.

SB 28:08

Yeah. Yeah. No, it's phenomenal. And to do it with no funding.

P23 28:16

Yeah,

SB 28:17

yeah. No, I'm very impressed. And just no thinking or just sort of thinking now about maybe some of the barriers and to the service moving forward. And improvements that you see are needed. So maybe with first of all think about the barriers that are going to prevent this programme, the sustainability of this programme in SE Trust or maybe barriers for rolling out to other Trusts.

P23 28:52

I think certainly for ourselves, I'm thinking about kind of the physical capacity in terms of the clinic space. I knew [CNS HNC] fought very hard to get us through the three rooms in the outpatient department. But there has been kind of regular, you know, if we haven't had patients, for maybe one or two weeks, it has been kind of contact me in three [CNS HNC] just to ask, was the clinic still running, and we could kind of see if that wasn't something that just because of the unpredictablality of our patient caseload that could be taken away. So I think certainly that physical space, it would be great to have that within our own department. Certainly, the AHP involvement as well with speech and language therapists that would be and again that's so invaluable for patients especially depending on their reconstructive element of their surgery. And there has been a number of patients where you just thought it would be fantastic if speech and language had been there to answer some of those questions and you know, I think that just that AHP input and I know again that [Name] mets, that’s our dietitan makes, I know she is extremely busy, makes her time and if they are thinner on the ground you could almost see that potentially coming away aswell. Again, yeah, potentially just that that running into, I don’t really know if that’s a valid point, just patient caseload as well, the unpredictability of the prognosis of the patients that we are seeing obviously determines how many referrals that we have got through during the month.

SB

Did you expect there to be more referrals than there were?

P23

Yes, well, I think when, when I first started in Plastics and Max Fax as a staff nurse there would have been 2 free flaps a week and we were really fighting for space to get them in and then unfortunately during the pandemic the people actually translating to surgery is probably significantly reduced and it will be interesting to actually see how many people that that would accumulate to. But I probably thought that there would have been more referrals, yeah. But like for example, again it is really patient dependent, we wouldn’t necessarily pop through the patients who are having the wide local incision alone because it is really quite a small procedure, but for example today I did put through a referral for a gentleman because of his information needs so because they would probably need one night in hospital stay and have very little side effects from there treatment but just because of the particular gentleman, I just thought it was appropriate to send through. So, yeah, I probably would have expected more referrals but there seems to be peaks and troughs were we have two patients together or one each week and then for two or three weeks there wouldn’t be anybody.

SB

What improvements do you think would enhance the sustainability of the programme?

I think, I think certainly team promotion of it, and again that’s something again not just, not just probably the CNS’s responsibility but the consultants responsibility aswell to promote it within our consultations, which I know the consultants are very very supportive of it, its just again that remembering that it is there, and I know we have been doing it for quite some time now, but I think when you are speaking about so many other parts of treatment, or, yeah, just that that team promotion of it, again just, seeing it as part of the patient pathway and maybe just incorporating it into more formalised pathway in terms of our referral process and kind of our our decision planning process of treatment, just formalising it more, maybe ultimately making it an opt out service…

SB

Ok, yeah,

P23

Rather than opt-in… so this is an automatic referral that is processed that people will, you know the CNS will discuss with you and if it is not for you, it is not for you, but the referrals are being made anyway. I know with our smoking cessation referrals that that process was changed when I started in the service, and the amount of referrals and the success rate of people quitting smoking did increase significantly.

SB

Right, so that’s very, that’s a very valuable contribution there, yeah, that will be definitely recorded there, so that’s great, yeah, I hadn’t really thought about that, as an opt option, yeah. Are there any other improvements that you think could be made?

P23

I would say just again that in terms of clinic capacity, I am probably asking for a bit much, but again for that patient coming to that appointment, its another department that they have to go to. Ideally it would be fantastic if it could be either within the Oral Surgery Department or within our pre-assessment appointment, because those are the two places that the patient would have to go and prior to the appointment we really have to explain, and I don’t know if that can be helped because of the way the Ulster Hospital is. It is a little bit difficult to find and its then just having to say, if there are any issues just give us a call and we will collect you from the front of the hospital. I think location, if we could some way find a better location, but it very much is, we are so grateful for it in a sense as well the outpatient department are fantastic in letting us have that but in terms of practicalities for the patient, if we could find somewhere a bit more central and somewhere they are more familiar with.

SB

Yeah. Yeah. Yeah. No that’s fab, another really valid point there, I suppose it is, if you had a wand and you could create that space, definitely the physical space is harder, but, yeah, definitely for consideration. I don’t have anymore questions for you [participant name], do you have anything else you want to say about the programme that I haven’t asked

P23

No, I don’t actually think so, trying to think, its been extremely thorough, and we have gone through hopefully lots of really valuable questions to ask, so not at the minute, no I can’t think of anything else additionally yeah.

SB

That’s dead on, I will just stop this recording then.

# P24

SB 0:05

Okay, so can first of all, and can you tell me about your professional background delivering physical activity interventions.

P23 0:19

so I've been moving more coordinator for Belfast for five years. So that's probably taken up the most of my time in terms of like physical activity interventions. And I have a degree in sports science, and a master's in physical activity, and it was population health back then. But I think, health then I went into Action Cancer for six years as health promotion officer so my remit then was sort of about physical activity and cancer prevention and developing and delivering to sort of health promotion, cancer awareness, cancer prevention type programmes. And my remit was sort of overseeing physical activity. So yeah, and then I've sort of been here for five years. So I actually don't deliver the exercise for Move More because we have a full time coach called [NAME], who, he's funded through Active Belfast so I coordinate the whole programming and coordinate, like, oversee, the delivery, but [COACH NAME] delivers on that, if that, if that makes sense. Yeah, so that's okay.

SB 1:37

You know, so that's, that's quite different than the setup in the other areas is that

P23 1:44

is the only one that has a dedicated coach on on the programme for the delivery. It must be just to do with partnerships with Move with Move More Belfast. There was a physical activity cancer rehab coach in place in the Belfast trust before Move More was initiated. So I think that link is just being kept then but it works well. Its because Belfast is so busy. It means then that I can coordinate everything else, and [COACH NAME] does all the delivery there. So it frees me up a good bit of it as well.

SB 2:21

Yeah. Okay. And so you've got quite an extensive experience then working with patients with cancer. And in thinking about any other complex illnesses or as it has a lot of your work centre around cancer then.

P23 2:35

Yeah. When I've been, with Action Cancer, a lot of it was more cancer prevention, cancer awareness, so wasn't actually dealing with a lot of people with cancer diagnosis, but they have a positive living programme for people who have had a cancer diagnosis. So our team would have been involved in delivery of that. There was no, there was no physical activity at that stage. When I was there, there was no physical activity involved. But it was just talking about how physcial activity can help following a cancer diagnosis, but the bulk of it's been five years of being much more then

SB 3:13

okay. And before the cancer prehab service started, what was your understanding of the Move More coordinators role in delivering the programme?

P23 3:25

Sure. You got stuck there

SB 3:28

You know, so before the Cancer prehabilitation Service started, what was your understanding of the Move More coordinators role and delivering the prehab programme?

P23 3:54

Probably very little from the formal prehab pathway, but we've always accepted referrals from the point of diagnosis. So that would some people could have been referred and just as they were diagnosed before treatment started. So that that just would have been more a normal Move More referral and, you know, the reasons for that would be slightly different than a rehab element of it. In terms of sort of a formal prehab pathway was wouldn't have had extensive understanding or knowledge of it as it as it is at the minute, just as referrals just pretreatment as such.

SB 4:43

Okay, I suppose what that I wanted to get a sense of is what you're doing now what you imagined you were going to be doing, or is it very different

P23 4:58

for me because Belfast I'm not sure if you know, but Belfast Move More Belfast came on board slightly later than the other areas. Because Belfast isn't in the SE Trust. But there was a lot of patients who were being treated in the SE Trust but live in Belfast. So I came on board quite a few months after the girls had already started. So I had an idea just from listening to them, sort of what was involved. And so I suppose from that point, I, I kind of I kind of knew, I knew what it was. And then it was just put that into practice, then there wasn't much that I had to learn or much the wasn't already aware of, and needed to be beforehand. So they had given me sort of a heads up as to what was expected. And so I don't know whether that's even answers your question or

SB 5:57

Yeah, no,

P23 5:58

you want to? You want to get?

SB 6:02

Yeah, so you had a very had a slightly different starting point that they did. So that's interesting. Okay, so how, well, did you feel prepared for this role.

P23 6:19

to be honest, at the start, I kind of was a bit apprehensive because there was so much involved, than what we normally do, so I, and I suppose I don't know if I'm saying that I was not prepared or that I wanted to make sure that it was doing the best and more. So it was kind of putting a little bit more pressure on myself. But just in terms of like, all of the forms and you know, everything that needed to be paperwork, and assessments everything that was obviously very new. So I suppose, like, they were very good, like [PERFORMANCE MANAGER] was very good. And, you know, meeting me on my own and going through everything. So like it wasn't thrown in at the deep end and say, right, go fill that form in. And like, there was a lot of preparation, it was probably more just for my own, wanting to make sure that I do do it right, and do it well. But like, we met with Jeff and separately as well need went through like the SOP. And like there was like, I supposed I was prepared. Because the one thing that the guards already went through a couple months before that, and so, yeah, it's just one of those things. Because it's unknown. It's there is always a bit of apprehension as to put that into practice, then, because that wasn't something that we were used to be doing as such as on a normal pathway. But with the help of [PERFORMANCE MANAGER] and [HEALTH AND WELLBEING MANAGER] and those separate meetings, I suppose I pretty I was quite prepared for it and was, you know, I was able to, like there was, I had no issues when when I did start, because I've gone through everything sort of test runs out as well.

SB 8:09

Fab. Right. Okay. And And who did you do the test runs with just out of interest?

P23 8:15

[NAME OF PERFORMANCE MANAGER] shes the Performance Manager...I don't know, her correct title? Yeah, she had taken some time and met via zoom. And we had gone through the form, and she had sent like a wee test form just to make sure that I could get it and what I was filling in, I was able to send back. So that was really helpful. Yeah.

SB 8:36

Brilliant, brilliant. And, and so the programme had already been planned, and the SOP was already in place for the time you're in. So you weren't in those initial stages you weren't involved in the planning and co creation at all.

P23 8:52

No

SB 8:52

From you joined, when did you join what month.. you remember?

P23 8:57

I'm going to say June...

SB 8:58

Okay. From that time, then you would have been involved in the working group?

P23 9:03

Yeah,

SB 9:03

yeah. How how did how do you feel that helped you in your rolel?

P23 9:11

And it was good to be able to put faces to the names sort of be, to be honest, like, I, I don't really be involved in a lot of discussion in that working group. But it's, it's good to hear your discussions on this, you know, and like, if any issues are, like, you know, I can I can bring it to the working group, but it's good to be able to hear the things that are going on, you know, within that group and within the programme itself. But, you know, it's it's been, it's been beneficial and if there's been it's a good opportunity that if there is an issue or there's something that needs to be discussed that platforms there for that hour whatever, and usually all of the representatives or some of the, majority of them are there any way to you're able to get speaking to the person that you need to speak to? I think it's good. I think there were there were more frequent at the start. I think they were definitely more frequent when I first joined. But now they're once a month, whatever. But I think it's good to be able to have that opportunity just to, you know, the few that I've been on, you know, there always has been something that needs to be chatted about. So I think it's good. And because everybody's so, so sort of spread out across this, the trust that it's going to be able to bring everybody together.

SB 10:35

Yeah.

P23 10:36

On zoom, or whatever, you know, that video call platform. To be able to do that.

SB 10:42

Yeah. And you say, whenever you're like so, a lot of your work would be rehab. And I know, you say that you have received some patients from diagnosis. They, was there, how did you feel receiving the prehab patients at diagnosis? Was that something that was quite straightforward for you? I know you've had quite a background there in cancer. So tell me a wee bit about how that was receiving those patients?

P23 11:11

Yeah, it was fine. I suppose my, my biggest fear, fear's too big of a word. But the fact that there were these patients were being diagnosed like, I'm we're phoning them two days later, I suppose that was the only thing that was kind of worrying me that, you know, there was so much for them to have to take on board. And then I'm phoning them and saying "Let's do a physical activity programme," and don't get me wrong. I, I believe in it. And I, you know, that is what I want to do. But I suppose that was just that the only issue if you'd like around receiving those patients was that these people are having so much other things to deal with and put so much in place to get their lives in order that was I suppose more it was more what skills I would need then to kind of have that encouragement and that motivation to try and get them on board then to be able to like there hasn't really been anybody that said no, or like, I think they get a good bit of information from at the CNS point as well. So they're not hearing it for the first time. But in terms of receiving information, like in the form, it's all there. Like it's very clear. And if there's any additional information CNS need to put in they do. And that's good to have that, you know, before you phone them. But that was that would be the only because it's so close to diagnosis that, they probably don't get a long time, or any time to process what I even like, tell the other family members that they need to before my phoning and so that that's the only, I don't know, is it an issue?

SB 12:58

No, but and then you've described that there as like, that wasn't what you're apprehensive about and fearful about in terms of going moving into this project. So how have you found that?

P23 13:15

Okay, and once you get over the first one you hear yourself, like, saying it and having you know, it's been okay. Most people are very receptive to it. And I try not to like bombard them with you know, all the jargon at the start, you know, I'm just saying, I'm part of the prehab team, and whatever, it'll give them the options, and most people have been receptive, or even though it's just in my head, that's what the issue was. But when it comes to the patient themselves, it's maybe not being as big of an issue as I thought it was going to be for them. But yeah, most people have been pretty receptive of it. But I think there's only been one, one patient who had an alcohol dependency and didn't even answer the phone and then sended a message and said, "I don't need your help." So that's been the only one where everybody else has been, you know, in one way or another has been able to get exercising and start exercising or you know, used to help with us and the way packs are given as well. So, just getting over that initial maybe one or two

SB 14:32

that a lot that's really interesting to include and the findings of this and Claire is that once you give it a go, actually patients are really receptive. And and that that's good to communicate to maybe other people who are embarking on rolling this out.

P23 14:51

Yeah, it is like it's,everything, you know, change and unknown, doesn't sit well with a lot of people And especially when you've been so used to doing, like a lot of us move our coordinators have been in post for several years, so we've been doing the same pathway, the same model, the same way for so long. And then when something else comes in, but it's been, it's been grand, and the fact that there's been a couple of us doing it at the one time we've been able to get that support from each other as well, which has been really helpful.

SB 15:26

And do you have wee windows were the four of yous, or I know it was three for a while there, and have been able to, like, discuss more challenging issues? Or have you been able to just between yourselves, outside of the working group been able to share and learn?

P23 15:45

Yeah, we have a wee WhatsApp group, as well with the four of us. And so [MMC who left post] got threw out of it last week, and [NEW MMC] has joined it. So yeah, just if there's any, like, what do you think about this? Or, you know, what do you do this sort of thing. And then we would meet sort of, sometimes just maybe the week before the working group, just if there's anything that we need to kind of discuss ourselves, but we also have a community of practice with all of the Move More coordinators once a month as well. So for a while, the Prehab has been quite a big bit of that just just to discuss that it's been gone. And it because the other coordinators will be coming onboard the next couple months in their own Trust then, just recently, we've been chatting a bit more about it. But yeah, we do have like a more, as much as we're in different council areas, and we're employed by different people, we, we work really closely together, most of the time anyway. We probably work closer with each other than we do with our colleagues in our council. So it's good to have that.

SB 16:49

It is fabulous to have that network and that smaller network, and then not the bigger one. And actually, those conversations that you're having with those other people are actually probably breaking down some barriers that they're going face.

P23 17:02

Yeah, so yeah,

we've all we're, well, maybe a year into it, like, and, but like, nearly a year into it, you know, so we have a year under our belt of doing it, where they're just coming into it. And it's good for us to say look, this works. Do it this way. Try not to do it this way. You know, just to give them a wee bit more reassurance that yes, it's okay.

SB 17:30

Yeah,

P23 17:30

right.

SB 17:32

Yeah. And you'll be on another Whatsapp group getting lots of questions. [laughs] How hav you found collecting the data, the sort of the patient reported, you know, the, the data that you're collecting time points tell me wee bit about that.

P23 17:51

That's the most difficult part, because there's quite a lot of it. If it could be streamlined, I don't know if that's the right word, but condensed down that we don't need so many time points, that I would find that a bit easier. And again, because we're trying to get baseline and then the pre surgery or pre treatment data. And sometimes that's like a week or two apart. So I feel like I'm, you know, unnecessarily asking for information when they're probably trying to get everything in order for going in for treatment or, or for surgery. And I've had quite a few that I couldn't get back in touch with again, so then have to be put down to be put down as a drop out, where I've only really had maybe two of the three month follow up points that hasn't been too onerous. But it's just that the baseline and then the pre pretreatment and again relying on the patient telling me when they're due for surgery, or when they're PCR is, sometimes that doesn't happen. And by the time I get back in touch, then they're already in so that's been difficult to get. The questions questionnaire bit is fine because you can do that over the phone. But the functional assessments is more difficult because you need them to come in to the centre where, with COVID and germs everything at the minute some people are a bit more reluctant and I am not sure what the the girls think, you know, we're swayed, but personally, I don't feel comfortable doing it over zoom. Just because they are even over the phone because I feel like you need to be there more for health and safety reasons than anything else. Yeah, so collecting the data is probably the most important bit the most difficult bit.

Yeah.

Again maybe the time, the timeline for people is just quite short. And as much as they are, they know that exercise is really important in the lead up. But when it comes to it, it's probably not as important as some of the other things that have to get in order and put in place before your treatment starts with their their surgery, but there's definitely been a few that may be come to the centre, so [MMC COACH NAME] would do the exercise with them, he would do the functional assessments with them,

SB 20:29

Oh right, okay,

P23 20:31

and then we're relying on them being able to come back into the centre again for the follow up. Even if they're, if they're happy enough to meet him, even just once or twice, and then do exercise themselves at home, we're still relying on them telling us when, but I try and keep in touch with everybody at least once a week, whether it's a call or text, which is not always easy, easy to do. But it's that reliance on them telling us when they're date is if they don't already have it. Because I did, I had a chat with [HNC CNS] about it, and, you know, we're saying like, because we don't have access to is it ECR, that system so we can't check or, you know, anything like that, ourselves to keep an eye on that. So if it was a way that CNS or somebody could give us and [HNC CNS] would be, would be quite good at doing that as well, letting us know, here's a heads up, this patient is due on this date which is good. And I'd also say to her at one stage, if it was possible for some of the functional assessments to be done pre op or like, when they're, when they're admitted for surgery you know, if it was just a blood pressure, 30 seconds have to stand or at least in it, there there are it can be it could form part of your pre op or that they're the day that they're being brought in for surgery it could still because they'll already have been part of this programme, they'll know, they'll have done some of it so it would not be like, what I have been asked to do a sit to stand for it could just be part of that pathway, if that was an easier way of making sure they're

SB 22:17

Thats very interesting. And that's, that seems like a really plausible solution.

P23 22:26

Am I naive as I'm not a nurse. So I don't know what goes on, like when they're admitted or at pre op. But I was just thinking they're gonna have their blood pressure done anyway, their BMI is hardly going to change drastically even if it's five weeks, or you know, I think because [ADEPT / Doctor name], was the sit to stand is the most one of the biggest, most important parts of the information or the data that we need to collect. So even if it was just a sit to stand, the handgrip test, again, I don't know if that's, you know, as important, but even if it was just this sit to stand they're able to get like, like in my naive head, I would be thinking that could be done.

SB 23:10

Right. Yeah.

P23 23:11

You know,

SB 23:13

But yeah, that's really interesting. So it's the time point so it's time point two that you's are struggling with the most.

P23 23:21

Yeah, for me, anyway. Yeah.

SB 23:25

Time point three is three months post surgery or three months? From baseline?

P23 23:33

You're asking me, I well you know what, there's not I don't think there's much time really between them.

SB 23:40

And that one's easier to get isn't?

P23 23:45

For me, I've had a lot on that I could not get in touch with again. Okay, I've rang left messages, texts, and nobody's got back but I've had two, one what had actually already started our rehab classes. So he was fine. And the other one have been in touch quite regularly with her but her side effects of treatment are really bad. I sent in her the question the questionnaire data, but not the functional data because she just wasn't able to come in but she is really keen so I do hope to get that too. So I've only had two

SB 24:22

okay,

P23 24:23

Out of all them, I don't know if you have the total referrals for each of us but I haven't had obviously as many for Belfast because there's not as its not Belfast trust, you know, its on the periphery. And so but for those, that wasn't too bad, to be able to get that like it was as he was already in our class. So it was easy enough.

SB 24:50

Yeah, yeah. Well, so in the time that you've been involved in the project the last six nine months, I suppose now, how has the programme evolved in that timeframe? What has changed from when you started in June to now, what are you's doing differently?

P23 25:12

That's a good question. The form has changed just recently to capture an idea of what exercise is being prescribed, I suppose that's, I don't know what if there's been really anything that's done?

SB 25:32

Has there been any change to nutritional advice?

Unknown Speaker 25:38

No the advice, not no, everything's stayed the same. There hasn't really been I haven't had sort of any needed to have any major in-depth conversations about nutrition or anything like that. And in terms of the emotional like, I just do, you know, there's the chat, just have a chat, and, you know, whatever. I would always just tell people, you know, if you need any support, or I can be your point of contact for making referrals on or signposting on if you didn't know where else to go? But I don't, I'll probably come off the call and be like, Oh, that's changed but I can't think of the top of my head, that's, em

SB 26:35

That's grand. And in terms of then the exercise prescriptions, you had to be a little bit more and detailed about what was being prescribed. Did that change your practice? Did did you become? Or did you become more intentional about what you were saying to patients as a result of the form asking more?

P23 26:56

Yes, that no, because I actually haven't had to use that form yet. So it will, it probably will, when I have to use it, but we I kind of just take the lead off to patient as well as to kind of what, like a lot of them, I'm finding they're already walking or golfing, and they're happy enough, just to do that. And then I would try and go through, check out some exercises from the wee pack that they're given, and, you know, recommend that they focus on on that, particularly the ones that maybe don't want to come in to the centre.

SB 27:36

Okay.

P23 27:38

But maybe in another couple referrals time, we'll know the answer to that question, because I haven't had to use the form yet.

SB 27:46

Yet know, them. Fair enough. And so what impact and you've touched on this already a little bit, but what impact has COVID had on the service provision of the cancer prehabilitation programme, and you have mentioned, the fact that the sit to stand that the functional assessments are pretty impossible to get when restrictions were in place, but what other what's sort of our impact do you see COVID having on the service.

P23 28:16

it's just the reluctance of people to come in to the centre. Especially, there's, there's been a few that maybe actually haven't been out at all, you know, even before their diagnosis, and now there even more reluctant to go anywhere, like, like, it with all the will in the world, like we have, like mitigations in place within the centres. And, you know, we are explaining that and all the detail, but obviously, it's everybody's personal choice. If, and again, that could be a perception too, for people who haven't really been out and about much. They're just they're thinking, oh, no, there's too many people where we can make it that is just them and [NAME OF MMC COACH] or you know, there's no there's no one else about but that's probably been the hardest part is actually implementing the bit that the whole thing is about is that you know, the exercise, but then some people will are happy enough to do what they can at home and are happy enough maybe just to make [MMC COACH] once they don't want to come into the centre on a regular basis. And then he'll give them something to do at home using their their pack. And and then it's probably again getting that like trust relationship built up as well because what we would have done before COVID just in general, didn't know we'll meet a referral come in. I would have met them and we used to meet them in the leisure centre and gone through like all the chat all the discussion then, now we're doing it all over the phone. So you know, for prehab you're trying to build up more trusting relationship more quick, quicker. And I suppose that's kind of been hindered as well, because that’s, people, things are completely back to normal yet. It’s the face to face stuff that’s probably the hardest for people. You know you are explaining that we have all the risk mitigations in place, but there is probably too much at stake for them to take that risk.

SB

Yeah

P24

Particularly for those who have a short window to their treatment or their surgery.

SB

Yeah, yeah, and you totally appreciate that. What do you think has helped people engage with the programme? And we have talked about some of the barriers to engagement, but what do you think helped patients, what convinced them, or made it possible, or made it easier for them.

P24

Its not like they are hearing it for the first time, the CNS’s, the CNS’s obviously had to get their consent to come on to the programme so there has been a lot of information provided from the CNS at that point so we’re just saying “I’m such and such and your CNS would have explained that this is what we are going to do.” I don’t know, I kinda try to put myself in their shoes, you know, what would I want to hear that would make me, right this is what I need to do before my surgery or my treatment but maybe, I don’t know, maybe it is just the way it explained and that they have that one-to-one opportunity that’s very much just focusing on them and their needs and what exercises would be of benefit to them. I suppose with Move More, it is informal and it is flexible so I try and keep that, so its not, you’re being prescribed this, and this is what you have to do, but its taking into account all their other factors in their life that you have to accommodate too, I don’t know, I think its just, you know what, a lot of our training from Macmillan would put us best placed to be able to do that in terms of motivational interviewing and managing difficult conversations and that sort of thing as well, its just kinda just what we do…

SB

Yeah. And how have you found people’s family, like partners or wider family? Has that influenced participation or not?

P24

For the most part it hasn’t been an issue, it hasn’t, it doesn’t even come in conversation except for two that I can think of, so one was a lady her daughter done all the talking, and it was her daughter that didn’t want her to do it, in fact, this, they were initially were doing it on Zoom with [] and then the daughter cancelled and they were supposed to rearranged but he could never get back in touch with them again, so that was the only one. So I don’t know the background or context. And then the other one was a lady who was a foreign national and her English wasn’t great, but she had a couple of children with quite complex needs and her children’s daddy lived in Newry or somewhere, like this poor girl had no time to have cancer or have a cancer diagnosis never mind come onto a prehabilitation programme so I suppose that wasn’t like someone influencing them but more just her circumstances. So she was able to meet [MMC coach], I think, she didn’t have an awful lot of time either between diagnosis and surgery and she had to do it at school time, but there was loads of appointments to get booked in but she was able to meet him once. But she was supposed to have 5 or 8 weeks, but whenever I rang her to see how she was, she had already had her surgery. So that’s kind of been the only factors that’s been the lady who’s daughter done all the talking for her and then that girl, the single parent with her children with complex needs.

SB

Have you found any families encouraging, sort of any positive influences from families to engage?

P24

Nothing specific that I can think of now.

SB

That’s ok. You have talked already about the support through the working group and being able to meet up with the other MMC’s aswell. Have you any thoughts on the whole referral process, you also mentioned about the CNS’s adding extra little notes that you had found helpful when they were referring patients through. Can you tell me anything more about how you found that process and what has helped or hindered you receiving the referrals?

P24

Yep, like the referral form is so detailed, like there is so much in it so it is really useful and then when the CNS’s are doing their assessments, like that’s all explained in the SOP, like whatever trigger points they meet as to what referral pathway they go on and yeah I think it speaks for itself because there is just so much information in there that I haven’t had to go back and ask for any further information and I think that just speaks for itself in terms of what that information is being captured and sent on. And that wee box for the CNS’s to say, cause I think that some of the head and neck there has been alcohol dependency, so they have been able to give that brief background information and anything else they think we need to know, its there. Its pretty straightforward, as long as they put, whatever information they’ve got, send it to us. I think there is one which has come to me via Lagan Valley and that was put in the wee box as well, that the CNS hadn’t actually met them face-to-face but had sent the pack out, or that they hadn’t received the pack or hadn’t been able to receive the pack so that was useful to know as well. It didn’t change anything but it was just kinda useful to know where they came from in terms of where they were referred.

SB

Yeah, yeah, ok. What learning have you gained from delivering the cancer prehabilitation programme?

P24

Oh [laughs] good question… I suppose just that sort of multi-disciplinary team working that it works and it just shows the benefit of it, God, I don’t know, I hadn’t really thought about that….

SB

Just thinking about knowledge, skills, job satisfaction, what sort of ..

P24

We have, like I said, Macmillan have put us through so much training that we have been very well placed to facilitate this but I have, I think it was called Vicarious Trauma training, at the end of the summer last year and it was dealing with having those difficult conversations so that’s kind of been useful for me in terms of speaking with people, because the referrals we would have got at point of diagnosis would have been few and far between, most would have been rehab, so having those conversations with someone at the point of their diagnosis was pretty new, so I suppose I have been able to learn to manage that you know and that’s good experience to share with my colleagues as well, so that’s probably a big learning, not that its any really different but its just more to keeping them in a safe space and me in a safe space, its pretty emotional conversations and then having to use a bit more motivational skills at that point, cause as I’ve said there is so many competing priorities for them at that point for the people that have just received their diagnosis 24 48 hours before that.

SB

Have you enjoyed being part of the programme?

P24

I have, like as I say, I believe in it and I think its probably the way that a lot of conditions are going to go in terms of prehab, its just the amount of all the data that needs captured, I think theres probably the Move More coordinators have the responsibility for that themselves, on top of their already busy workloads and caseloads and that’s probably the bit I enjoy the least if I am allowed to say that. Or if there was anyway that could be changed so that it wasn’t just on the Move More coordinators to capture that data. But for the few that I’ve had, yeah, its been enjoyable that I am part of a team that’s helping someone prepare for the biggest thing in their life and prepare in a positive way and even if they don’t engage as meaningfully as we would have hoped then its just having that point down the line that if they do feel overwhelmed or that they do need support that they can come back and we will be able to have that point of contact, to be able to signpost them or refer them for any additional support and also then to support them a wee bit sooner then their rehab can start straight away at that appropriate time rather than 2-3 years down the line that they are already in the system.

SB

Yeah, yeah, and its nearly seamless. Just finally, can you think of any improvements that would enhance the sustainability of the programme so you have mentioned a few things throughout the interview, thinking the timepoints and thinking about other ways to collect the data… Are there any other thoughts that you think around improvement?

P24

Probably just those that I have mentioned already but obviously where the main point is to get people to active and to have that exercise to get them to the optimum position for treatment or for surgery. And then we are facing with COVID and as well do we need to have not everybody will have Zoom and not everybody will be able to use Zoom at the appropriate time like maybe more in the evening time when we are not available so do we need to have something put together in terms of a video or something like a bank of exercises that they can use in their own time if they don’t want to go to the centre that they are still able to get the physical activity but obviously it would be us Move More coordinators delivering it pre-recordedly

SB

Yeah yeah.

P24

So that would be the only thing. If there was no Covid there would be no barriers

SB

But even without Covid that hybrid model could meet the needs of the likes of that patient you talked about with the kids with the complex needs and for people that its just not easy to get out never mind Covid, that hybrid model could have an advantage, so yeah

P24

Yeah. In Belfast, anyone who comes on to Belfast Move More, we give them 12 weeks free membership so they can still take that out and go in their own time if they so wanted without [COACH MOVE MORE] or I like there at the evenings or weekends, so that option is there for them but its their probably coming into contact with people that they probably don’t want to you know in the lead up to it, you know its an option its an option for them that they would be able to, and they know they are following something that’s going to be able to be safe for them as well cause Move More coordinators have pre-recorded it.

SB

Yeah, yeah. Have you anything else you want to say, I have no more questions for you.

P24

No, no, I don’t think so, I think I have covered everything, no sort of the only real issues was the collection of the data and then if there was another way for the pre-surgery data to be collected if they can’t come into the centre.

SB

That’s okay, I will stop the recording.

# Patient 1

Pt1

**SB**

*inaudible*

**SB**

That's perfect. Okay, so whenever you were initially offered the cancer prehabilitation pilot, what was your understanding of the benefits of being part of that programme?

**Pt1**

Well, my understanding was that it was first and foremost, to try and keep moving to try and just keep as active as I can. It was explained to me, also, that because I had a lung cancer, and that breathing, breathing exercises, all of that was going to be beneficial to me. And that was part of the programme. And then also was explained that from an emotional perspective, that it would, could possibly be beneficial, because it would be another person reaching out to somebody being able to speak to them about the illness, how I feel, how it's affecting me, physically, how it's affecting me emotionally. Yeah, it was it was. That was that was my understanding when I first, when it was first explained to me.

**SB**

Okay, that's, that's fab. And in some really, some really good things in there in terms of that, that connection with somebody, particularly during COVID when when a lot of our connections were a lot more isolated, what was your understanding of how the programme was going to be delivered at that time.

**Pt1**

At that time, it was explained, obviously COVID was going to impede a lot of what we would have wanted to do. So it was going to be regular check ins with [MMC NAME], my move more coordinator. And that was going to be via WhatsApp or email. And then any sort of couple of the movement classes that we did a couple of wee exercises and movement classes, and they were video call. So, [MMC NAME] demonstrated, I joined in so it was all done virtually. Yeah. And that was explained to me that that would happen.

**SB**

Okay. Okay. And you've probably touched on this a little bit already, about the main reasons you agreed to participate, so whenever the benefits were explained to you, did that influence you? or were there other reasons that you agreed to participate?

**Pt1**

I think it was one of those things. When you get your diagnosis of cancer, you sit there and you get this diagnosis that you spend your entire life, hoping you never do. And then you're the one who sits there and you get that diagnosis, and you just think your world has ended, you know, and being reached out to by somebody who is saying, no, no. You know, we're going to we can stay positive through this. We can stay moving, we can keep in touch, you can stay moving. We don't have to just sit down and say right, that's it. We were gonna keep going. We're gonna keep going along. And I found that even that alone, the reach out, somebody's reaching out and saying, no, no, come on, Pt1 we're going to do this. We're going to keep moving. That was in itself one benefit. That was amazing.

**SB**

Yeah, yeah. Yeah. Very powerful. Yeah. Did you have any concerns about taking part in the programme at the time of referral?

**Pt1**

No, not really. I think I was made to feel, and this is honestly I was made to feel very comfortable with this. And I was always made to feel that I was in control of what could happen or couldn't happen. For instance, we had scheduled a wee exercise thing one day, and I was just feeling I was just after chemo, I was feeling awful, really drained. So instead of exercising, we just had a video call where we talked about how I was feeling how chemo was affecting me. Just been so sick, and weak and even that so I couldn't go ahead with the exercises, but I didn't feel that I needed to over explain it or anything. It was fine. I was able to say I'm physically not able. Yeah, it was okay. So yeah, felt felt very much. I was in control.

**SB**

Yeah. So I'm just interested there so this you were getting chemo and doing the exercise.

**Pt1**

Yeah, so yeah, yeah, I started my diagnosis. So I was diagnosed I went into hospital random pain. x rayed cancer found diagnosed within eight days.

**SB**

Okay.

**Pt1**

I then started chemo. So I was diagnosed on a Tuesday, they started my chemo on the Friday. So it was super fast. So even though I'd been reached out to with the move more, the chemo started really, really quickly.

**SB**

So so your treatment and the intervention, the prehabilitation, they happened concurrently, then they were together. Yep, that's okay. just some of the others are doing it prior to any treatment. So yours is just slightly different. So yeah,

**Pt1**

I think I think that was that was the idea. But we just didn't, there was no time. The consultant got me in and said I have a bed, we're starting you tomorrow, it literally happened like that

**SB**

Which is fabulous. Fabulous. Yeah, absolutely. Did anyone in your clinical team or your support network encouraged you to take part? It sounds like you were very motivated yourself. But was there anybody that kind of helped you or supported you to be involved?

**Pt1**

Yeah, I discussed it. Because at that time, I was spending time in hospital. So I said to you know, there were a couple of the nurses chatting away to me, and I said, I've been invited to take part in this. What do you think? And they said, That sounds amazing. You know, and there's one girl in particular, just chatting to them about it. And they were very positive about taking part. Yeah, yeah, for sure.

**SB**

And you're you said you were married? Is your was your partner keen for you to be involved, or?

**Pt1**

Yeah, yeah, I did. It was good for him. I mean, you know, me standing. Because we I could see nobody you can imagine there was nobody apart from my immediate family allowed near me. And because of COVID I was so vulnerable. And just seeing me doing the exercises here in the living room, jumping around with [MMC NAME] on the video encouraging me. I mean, that was good for him to watch as well, he was saying did you enjoy that. Was that good? Yes. It was great. Yeah.

**SB**

Yeah, very good. Very good. And did the cancer prehabilitation programme meet your expectations?

**Pt1**

Yeah,

**SB**

It did. And in what way?

**Pt1**

If I'm very honest, I thought it was gonna be a wee thing that you get involved with and you can, like could dip in or out or it's, you know, it was the commitment of everybody involved with it, that was really impressed me. And like I said, [MMC NAME], she's still in touch with me, bless her heart. And I've been I've been so you know, I had all my treatment, I became very ill at one point, during and after treatment, I got pneumonia, then I got pleurisy and all these side effects as it were, and these illnesses, so even though I was sort of absent, and [MMC NAME] was touching base with me, how you doing, she's still saying we're still waiting for you at the gym. We're still waiting for you to get involved. And thank goodness I think I'm fit and healthy enough now to start my

**SB**

Good

**Pt1**

You know, so it was prehabilitation and this is going to be for me as well some rehabilitation isn't it? It's going to be kept me going as well yeah.

**SB**

And it's like continuum of care isn't it that you know right from diagnosis right through.

**Pt1**

Yeah. Right from word go right through so you know, to answer your question Yeah, it did it it met expectations or possibly surpass them a little bit, just with the commitment involved in it and how everybody was, I took part in a wee video promoting it, you know, the, they made the video so I was I was asked to speak in that. Do a little speaking bit about what I thought of that. And I look back on that now. And I was unrecognisable I was so sick, I was swollen up I was bald, I was, you know, you know, but even being asked to take part in something like that, and yeah, it was brilliant. Great,

**SB**

Great great. Was there anything that didn't meet your expectations about the programme and within that you kind of thought that hasn't gone quite as I thought it should.

**Pt1**

No, no, not really. I mean, I think it was it literally was what it said it was. It happened, you know what they said it was going to be? That's what it was. Yeah. It was good.

**SB**

Yeah. Okay. And were there any issues that either helped or hindered your involvement in the programme? I suppose this is just to get a gauge for us if there's anything needs fixed about it, or that we need to do more of?

**Pt1**

I think, you see, it's it. It's nobody's fault. Unfortunately, COVID just threw a spanner in all the works, didn't it? I mean, it was just I felt, you know, I really felt for them, because trying to virtually do the get moving, get people moving, trying to and it's not everybody who's going to be comfortable leaping around their living room in exercise gear alone, you know. So it's nothing that the programme did or didn't do. But circumstances just were not the best I could imagine if we I was able to attend walks classes, meet other people, that would have been fantastic, but unfortunately, we couldn't.

**SB**

Yes. And yeah, yeah. And, yeah. What about the information pack that you received? What was your thoughts on it?

**Pt1**

Yeah, I mean, obviously, reading through that, I was thinking, yeah, it caught my attention. I was like, this sounds good. You know, I can you imagine, you receive so much information when you're first diagnosed from so many different people. And there's so many leaflets and things to read. So it was pretty good. Because it was very, it was very clear.

**SB**

Okay, good, good. And, and so you're you didn't you're this next sort of area is about whether you completed the programme or not, and I suppose for you, and it wasn't prehabilitation, per se, it was it was very quickly, nearly early rehabilitation for you. And that's an ongoing process, then listening to you thinking about the future again. what do you see as the benefits that you've gained? And you've touched on some of these in terms of emotional benefits and having that person reaching out to you and that connection through a very difficult time? What other benefits?

**Pt1**

That was huge, that that one alone is? It's very hard to explain, you know, when you're in that position, and you feel utterly lost, like someone's cut the rope, you know, that was huge. That benefit. And that. It's, I mean, I am not the most ideal candidate to speak to, like I say, because I did go into treatment so quickly. And then treatment was, and they said to me, this is going to be brutal. And it was brutal.

**SB**

Yeah.

**Pt1**

You know, so I wasn't able to go walking, go exercising before my treatment to build myself up. It was impossible. So perhaps, you know, I'm not the, the ideal candidate for speaking about prehabilitation.

**SB**

Yeah, yeah.

**Pt1**

But for me, it was it's, it's the hope, because it gives you that hope. When someone is saying, no, no, let's get going. Let's get ready for this. Let's get you stronger. Let's get you fitter. Let's get you ready for your treatment. Let's get so I think I said before you realise that. It's not the prebuilt rotation programme made me think. Alright, this is a process. Treatment is a process. Horrible one, but a process I need to get through and there is an end.

**SB**

Yeah.

**Pt1**

And that it's not you get a diagnosis, the end. Because that's where you go in your head immediately. And then being asked to be involved in the prehabilitation for me, suddenly made me think, okay, hold on a minute, I just have to treatment is something that I have to do. I have to be strong for it. I have to get through it because there is an end. And I need to be stronger at the end. And, you know, there's hope in there for it. Yeah.

**SB**

And did you see any other health benefits from being involved?

**Pt1**

Well, I'm, like I say yes, at the very start, but it was very short. And then I was immediately in treatment and immediately. But I mean, it encouraged me when I could. There was one day I spoke to [MMC NAME] and I said to her, I'd felt... So we'd agree that I would walk. And I would try and keep exercising and I got up. And not even I was physically feeling rotten, but I was also feeling just a bit sorry for myself and I thought, no, because [MMC NAME]'s calling me and I want to be able to say, No, I went on my walk. It helped. I was all right. So yeah, I do it made me get up and go out and bring the dog for a walk and get moving. Because I thought somebody's going to talk to me about this. You know, today or tomorrow? Yeah.

**SB**

So is that motivation [MMC NAME] provided? Incredible. Are there any areas of improvement that you could see that we could make to the programme? Is there anything you think, if they did this, a wee bit more of this or approach this slightly different either at recruitment or during the programme?

**Pt1**

I mean, I was provided with my little pack and that, I got my bands, you know, my exercise bands, because that was going to help me with all of that I was really fine with that. I was really happy with that. I mean, it's, it's hard to, for me to say my experience has been a very positive experience with it. So saying, where you could improve it's quite difficult, really, because it's sorry, that's just my husband banging around. Is that all right.

**SB**

You're fine.

**Pt1**

Okay. So, I mean, like I say, I would love to see what it would have been like, being able to physically be involved. But that's not the fault of the programme. That's not anybody's fault. I think you know, there was I was contacted early, like there was early intervention, after I got my diagnosis. I was speaking to somebody very, very soon. That was all brilliant. I mean, perhaps if you were a person, I'm quite chatty, outgoing person. So I don't know maybe if you weren't like me, if you weren't like that, maybe even just to more on what will I be doing? What will be happening? You know, the way like, can I see some other people doing this? Can I see some other people? What they've been doing? Maybe that would be helpful, but that's just been?

**SB**

Yes, that's it. Yeah. Yeah. But you were happy with yours? So I have no more questions at all. Is there anything else you want to say that you haven't said?

**Pt1**

No, I mean, like I say, very grateful for it. And it was really brilliant. It was a brilliant thing. Just being reached out to like that. It was a brilliant thing. And and like I said, that spark of Oh, right. They're telling to me to just get strong for this process for this small part. You know, the treatment and then and that was I'd say that was the best, best best.

**SB**

Yeah, yeah. Yeah. It just sounds listening to you just that it's just kept you going and going to the next, the next part of your treatment. And yeah, yeah. Thanks. Thank you so much for sharing that Pt1 I'm just going to stop the recording here

# Patient 2

Pt2

*machine voice*

This meeting is being recorded.

**SB**

Oh, it doesn't normally talk to me like that.

**Pt2**

Told me as well.

**SB**

So does just based on what you've just been saying to me, just so I have this on record, you're you've explained that, what you think you've been involved in is through McMillan and it's been rehabilitation? And you may have been called at one stage and, and asked about participating on a virtual exercise programme. But you didn't you choose not to participate on that?

**Pt2**

That's exactly right. I was just gonna say slightly corrected. Somebody called, it wasn't I think I was definitely called by somebody who introduced themselves. No, I thought it was the same thing I was already involved with until they began to explain that this would be virtual exercise. And I said, Well, I wasn't interested in that. Sorry.

**SB**

Yeah. Okay. And you weren't this Cancer prehabilitation wasn't introduced to you just after diagnosis. What you've been involved in has been after treatment has started. Yeah,

**Pt2**

Nothing happened until basically after the surgery and i was recovering. That's when various things began to fall into place.

**SB**

Great. Okay. Okay. Well, I think that's interesting. Just even your perspective there. Whenever the person called about the virtual, you weren't happy with that approach? You preferred the face to face?

**Pt2**

Yeah.

**SB**

Yeah. Yeah. And, and a lot of this cancer prehabilitation programme has been delivered virtually because of COVID. So just that that sort of getting that sense of your perspective on that is, is interesting. So thank you,

**Pt2**

If I might comment on is that I still have no idea what a prehabilitation programme might be. I can understand the rehabilitation. And that's what I think I've been doing. But a prehabilitation programme, I have no idea and the fact that no one was even going to meet with me and talk to me about it was just well sorry, I'm not interested.

**SB**

Yeah, yeah. And just to sort of clarify that for you now, the prehabilitation programme has come about with se trust has been implementing a pilot to optimise patients before surgery or before their chemotherapy, or radiotherapy, as a way to try to ensure patients are healthier at the end of it all at the end of all of their treatment, that and potentially particularly patients that are having surgery and that are going to be in hospital, it can reduce the amount of days that they're in hospital, and can help them recover faster. So they do this by its various different components. There's some exercise, there's emotional support, there's nutritional advice. And there's some behavioural change aspects like smoking, support cessation support, and looking at if there's an alcohol issue, then supporting patients with that. So that's the programme that's the cancer prehabilitation programme. And that happens in those few weeks that there are between diagnosis and treatment starting. So and that's what I'm evaluating.

**Pt2**

Right and I'm afraid that I wasn't involved in any way with any of that

**SB**

That is fine and thank you so much for being open to having this conversation. And clarifying that

**Pt2**

I've always been a bit puzzled as to what the phone call I got was all about didn't like sound like something I was interested in.

**SB**

Yeah, no, listen, thank you so much for your time and I will let you go enjoy the rest of your day.

**Pt2**

And you

**SB**

Okay, bye bye.

# Patient 3

Pt3

**SB**

So that's it now, that's perfect. Okay, so first of all, and can you tell me when you were first offered the cancer prehabilitation programme? What was your understanding of the benefits of it? What did you think? How did you think you would benefit from?

**Pt3**

Well, I mean, the text itself, I don't ever remember it being mentioned that it was, you know, a special pilot scheme or anything like that. I just was offered so many things. And I actually find that quite a hard question to answer. You know, there was no there's nothing to say you're partaking in something here. I thought this was pretty normal that everybody got offered all these different services?

**SB**

Okay. Okay, yeah

**Pt3**

Is that alright?

**SB**

Yes, absolutely. And, yes, so if you weren't aware of it as such, you know, you just were participating, you maybe weren't then aware of like thinking about benefits and things like that at the time. So totally understandable.

**Pt3**

I was completely gobsmacked by the whole diagnosis anyway. So really, it was the flow of things to be honest.

**SB**

Absolutely. Yes. I fully understand that. And at the time whenever they suggested that you take part, what was your understanding of how the programme was going to be delivered? Did you know it was going to be like via video or

**Pt3**

Well I suppose because of COVID I realised a lot of it wouldn't be face to face. But then, of course, so much of my stuff, you know, was face to face, I have to say, you know, with [CONSULTANT’S] team?

**SB**

Yes. Yeah. Yeah, that's, and what were the main reasons you agreed to take part in the cancer prehabilitation programme? Why did you engage with that? Additional, you know, the exercise or the emotional support? Why did you why did you do that?

**Pt3**

Well I think to make recovery better, I suppose. Yeah. The exercise was obviously, I wasn't, I wouldn't have been out and about as much at that particular stage. So at least [MMC] had me working at home here. And yeah, I think just to make me feel better about myself afterwards.

**SB**

Yeah. And did you? Did you take part on exercise before your treatment started?

**Pt3**

Yes. I couldn't. I thought I did. But I couldn't remember. So I looked at my on the phone, and I still had [MMC’S] last message on it's one of the MacMillan girls. And it was March and I didn't have my surgery till April.

**SB**

Oh right. Very good. Very good. Did you have any concerns about taking part in the programme?

**Pt3**

No, as I said, I didn't realise it was a special programme. I just thought this was what happens.

**SB**

Yeah, yeah. And did anybody in your clinical team or your support network, like family members, did they encourage you to exercise and be part of it?

**Pt3**

Everybody encouraged me. Everybody was great, yes.

**SB**

Good, good. And can you tell me a wee bit about the various different parts of the programme that you that you engaged with [MMC]? So it sounds like you did some exercise?

**Pt3**

Yes that's all I would have done with [MMC].

**SB**

Okay. And what about the did she provide any nutritional advice?

**Pt3**

No, but I had, I had a face to face meeting with the dietician. Because at that stage, I was struggling to eat anyway. You know, and sort of, then she prepared me for what I might or might not be able to eat after surgery you know. it was [CNS HNC] I think, she was great.

**SB**

Yes, okay. And that would have been that would have been part of that that as well. Yeah. And in terms of any emotional support, did [MMC] or anybody else provide you with emotional support prior to surgery?

**Pt3**

Well, [CNS HNC] that works along with [CNS HNC] on [CONSULTANT] team, she organised a video call with a girl who had I can't remember her name. She had had similar surgery to me I'm going to say nine months to a year before me, and my daughters, we the three of us went in and [CNS HNC] organised us having the video call with her and it was great. Absolutely great.

**SB**

Yeah. Yeah, no fab, that's good to hear.

**Pt3**

it was very, very reassuring. You know, because I really was scared stiff at that stage. So no that that was that was I think one of the best things that happened, you know, in the programme. I also had the speech and language. I had a face to face with them. Sorry, excuse me. Because they knew I would probably have swallow problems and how to deal with things, you know, as soon as they came out of surgery, and you know,

**SB**

Yeah that's brilliant.

**Pt3**

And also, I saw a Doctor [NAME]. He checked my fitness and did ECGs and all that sort of stuff to make sure I was fit for the surgery and that sort of thing.

**SB**

Okay. And it's interesting Pt3 hearing you say all of that, because a lot of those components are actually part of this prehabilitation programme, but they were happening naturally, you weren't maybe aware that was under that banner?

**Pt3**

I wasn't definitely wasn't.

**SB**

Yeah, so that's because, yeah, the speech and language were part of that programme as well. So great. Were there any barriers to being involved? So did you find the video approach like to the exercise off putting, or did you find it difficult to make it in to attend clinics? like you said, there you were in with the speech and language?

**Pt3**

No, I mean, because I live in [TOWN]. So I'm not far away from the Ulster anyway. So as it happened, I was able to get lifts in and out, and then at some stages, I was able to drive myself in, you know, but then I was I was on such strong painkillers. So I was reliant on this. But I'm not as I said before, I'm not a fan of video and zoom calls. So if I could have got either a telephone conversation or face to face. I was much happier.

**SB**

Yeah. Yeah. And was there anything that helped you engage with the those elements of the programme? So I suppose the getting the lift's helped, somewhat. And you had already mentioned maybe support from family also?

**Pt3**

Yes. Oh, yes.

**SB**

….helped as well. Yeah. You mentioned at the start, as well there that you wanted to get yourself, you know, prepare yourself as well as possible for surgery. And what do you see as the benefits off the elements there of the cancer prehabilitation programme? How did you feel know that you're through surgery and out the other side? What what benefits do you think there was if any?

**Py3**

I think it made me it made me more positive, and made me realise, yes, it's gonna be a long road ahead. It's not once not a case of just once surgery's over that's it that's over, you know, there was always that good support network beforehand, and things like that. So I always knew that there would be people at the end of a phone or I could email people. I mean, I could email [CNS HNC] and [CNS HNC], and dieticians afterwards, if I had any problems. So I think in a way, it increased my confidence of like going through the surgery in the first place. When I knew that would be good backup afterwards.

**SB**

Yes. Yeah. Yeah. And that's a really good point, actually. Yeah. That, yeah, knowing that there's going to be support can

**Pt3**

Oh, god, yes. Because it mean, I think when I was discharged, I know this is after surgery. But when I was discharged, I think it was a bank holiday. And we'd no telephone number for things like district nurses and stuff like that, you know? And that would have been one good thing, if one of my questions is going to be what would I do? Or what would I suggest would be a good idea? Something like a list of telephone numbers or something like that, you know, once you're discharged, you know how to get in contact with people. But sorry, that's nothing to do with this questionnaire. I do apologise. Sorry.

**SB**

No, you're fine.

**Pt3**

Typical me, I go off on a tangent every so often

**SB**

That's fine, because I think you've just pre-empted question, my next question was areas for improvement. So you're already there. So that is interesting. So maybe in terms of exiting from that, that that you're leaving hospital with those contact numbers? And yes, yeah, that's, that's, thank you for sharing that.

**Pt3**

I've just remembered another thing I find really useful. [CONSULTANT]’s team sent me an email with a link to YouTube stuff that takes you through the whole radiotherapy thing as well, which was I thought was was brilliant because I hadn't a clue about that sort of thing. So that reassured me right after surgery as well.

**SB**

No, that's great.

**Pt3**

So I have to say the team they were just amazing. I just cannot fault the back up I got was really good.

**SB**

And I suppose one thing I wanted to ask was about was there any areas for improvement around recruitment and just listening to you and you weren't clear that it was a programme as such, a pilot programme?

**Pt3**

No

**SB**

There's maybe something there about helping patients to be more aware.

**Pt3**

Yeah, well, I mean, I was never I don't remember. But in saying that a lot of stuff beforehand was a blur to me because it really knocked me for six. I don't ever remember being asked, Would I be willing to partake? You know, in this prehabilitation thing, I really don't. I just took everything I must admit, took everything for granted and I'm just so pleased everybody was doing everything to help me. But I didn't realise it was a special programme. I really have to emphasise that I didn't realise this was something that wasn't normally or beforehand anyway, I offered to people like myself, so I, I just thought it was great. Everything was just worked so well for me.

**SB**

That's great. Well, I don't have any more questions as such for you. And before I stop the recording, do you have anything else you want to say before I stop the recording?

**Pt3**

No, no, I mean, I think you probably realise how positive I feel about all the stuff that was available to me and I can't as I say apart from a list of maybe telephone number or something like that. I really can't think of anything else that they could have done for me and I think nothing more that they could have done for me. I mean, they were they were able to contact my GP and arrange stronger painkillers, you know, to get a prescription quickly and no, I thought it was very good. So great team really is a great team.

**SB**

Okay, well, I'm just going to stop this recording and then talk to you. Hold on.

# Patient 4

Pt4

**SB**

That's it recording now. Okay. So Pt4, whenever you were first offered and consented to take part in the cancer prehabilitation programme, what was your understanding of the benefits of being part of the programme?

**Pt4**

Really, it was designed to make me as the patient as fit and healthy as I possibly could be prior to the surgery. So I think that's about it. Yeah, really, just to get you fit as you possibly could be before you went on to the went into surgery.

**SB**

Yeah. Yeah. And at that stage at that early stage, what was your understanding of how the programme was going to be delivered?

**Pt4**

Because I think the, we were in a COVID pandemic I wasn't entirely sure, I had understood that you would normally have attended classes. But that didn't really pan out for obvious reasons. So it wasn't, I wasn't absolutely sure if the truth be told.

**SB**

Okay. Okay. But you were still okay to move ahead. And, you know, say that you wanted to participate, even though you weren't sure.

**Pt4**

Yeah, I thought conceptually, it was a good thing to do. I was aware it was a pilot and i was quite happy to take part in the pilot to try to *inaudible*

**SB**

Hello, hello. You disappeared there. Okay. No worries. So what were the main reasons why you agree to take part. So one of them was there in terms of getting yourself ready to be fit for surgery? Were there any other reasons why you agreed?

**Pt4**

Well, I guess, as I said, as you might not have heard it. I was aware it was a pilot. I personally thought it was a good idea. So I wanted to participate in the pilot with a view to hopefully this being expanded and used more generally. Conceptually, I understood what they were aiming for. And, you know, part of the goal, I guess, was to reduce the amount of time that patients had to stay in hospital to aid recovery, to benefit the patients, but also the benefit of the health service. So it seemed to be a win win. So I was very happy to participate.

**SB**

And did you have any concerns about participation?

**Pt4**

None at all

**SB**

Okay, you weren't worried it was going to add any extra appointments? Or that you were maybe at any an increased risk in terms of like contact with anybody in terms of COVID? Or there was no concerns around that?

**Pt4**

No, no, not really, because all of the appointments that I had had in the run up to this, we we were COVID screened and we would go to test centres and have a test and you weren't allowed to attend the hospital unless your test had cleared. So I just assumed that if that principle was applied across the piece, then there wouldn't be any more any more risk. actually didn't even think about the risks.

**SB**

Sure. No, and that's okay. Yep. Yep. did anyone in your clinical team or your support network encourage you to take part in the cancer prehab programme? I know you said there [CNS LUNG] introduced it to you, did she encourage you or was there any family family support there

**Pt4**

Complete family support. [CNS LUNG] and the team did encourage me but not in a pressured way. It was very much voluntary. I didn't feel obliged. But they did. They did encourage me. Yes. And my my family were very supportive. I was a pretty heavy smoker actually. So part of this was to stop smoking and they also put me in touch with who were also fantastic, the smoking cessation team. So that all seemed to be entirely sensible, and a good thing to do.

**SB**

Have you managed to keep off the cigarettes?

**Pt4**

Sharon, hang on, can you hear me?

**SB**

Yes, I can hear you. Can you hear me now?

**Pt4**

Yeah.

**SB**

Have you managed to stay off the cigarettes?

**Pt4**

Yes, I have. I we ditched we emptied the house. you know I went back to the office for the first time about three weeks ago and it's full of tobacco *laughs* I had to do a complete sweep and get rid of it all. I do use a vape. But I haven't been anywhere near tobacco since then. The consultant basically said to me if you don't stop smoking I won't do the operation, so I stopped.

**SB**

Oh yeah, yeah. So can you tell me Pt4 just all you said there you did the you engaged with the smoking cessation what other elements of the programme Did you engage with? Was the exercise was that one part?

**Pt4**

Yes. The there was a lady. My apologies I can't remember her name who because of COVID we would normally have met at a gym

**SB**

Hello sorry Pt4, you've disappeared there hello I'm just waiting now for Pt4 to phone back, hopefully he does.... *space while waiting to reconnect* Hello Pt4?

**Pt4**

Hi hello there. Sorry, I don't know what happened. I think we got disconnected.

**SB**

Yeah, just it disconnected there. Sorry. So you were telling me about the elements of the programme. So you weren't sure of the person who was your move more coordinator. And you were just you were talking about that and talking about the various elements of the programme you've been involved in?

**Pt4**

Yes. So the, I wasn't as diligent on the Exercise Front. And that was an exercise regime involving resistance bands and things of that nature. And we sort of agreed that I was doing basically a five mile walk every day with hills involved to try and push myself a wee bit, and we more or less agreed, well, I certainly agreed that that was a better way to prep myself than the exercises with the resistance bands. I just push. I pushed myself harder than I think I would have been required to do.

**SB**

Okay, very good. So they were able to tailor that then to to work around what suited you.

**Pt4**

Yeah, yeah.

**SB**

Very good. And in terms of was there any other elements of support provided in terms of I'm thinking nutritional advice, emotional support.

**Pt4**

Everything was offered, certainly emotional support and nutritional advice. I mean, on the latter, I guess I not only did I stop smoking but I stopped drinking alcohol as well. I just put everything to bed basically stopped. And we would be reasonably healthy eaters in any event, so whilst we had quite a lot of support and literature we were probably there or there abouts even before the programme. There was a lot of emotional support offered. The Macmillan team were really fabulous but I'm I'm lucky in that I have a lot of support at home. I'm a fairly upbeat person so I didn't, I didn't avail of the services. They were, they were certainly offered.

**SB**

That's okay. And did the cancer prehabilitation programme Pt4 meet your expectations?

**Pt4**

I'm gonna say yes. Although I don't know that I really had any expectations if you if I'm making sense here. But yeah, and you know, for the wider, one thing that really impressed me was when the exercise sort of the instructor, actually I've found her name I found her on WhatsApp. When she came on, it's abundantly clear that they, they tailor the exercise regime to the abilities of the individual concerned, which was really impressive. So if you had been a little bit older, or perhaps not, so, excuse me, not so good on your feet, you'd have been the exercise regime would have been designed around you sitting down. So they, they they, they clearly, [NAME OF MMC] actually was the lady who looked after me. So she she was very, very capable and very clearly had a very wide knowledge of how to assist people to exercise across a vast range of abilities and disabilities. It was all very impressive, actually.

**SB**

Yeah. And was there anything that didn't meet? You're saying you didn't really how you're quite open minded about it. But was there anything that you thought didn't quite meet your expectation?

**Pt4**

No, no, I, I would say my lasting impression was that I was very impressed by everything, the support, the fact that somebody came out to the house to teach me how to do exercises, the constant calls from the smoking cessation team. Because that started off weekly, and then I think went to, after a while it went to monthly, and now it's every quarter I think, so a lot of a lot, a lot of effort was clearly being put into it. And I felt just very fortunate to be... it felt like the health service and put its arms around me if I could describe it that way.

**SB**

Yeah that's great.

**Pt4**

And at that point in time, when it was stretched to the limit. That was that was particularly impressive.

**SB**

Yeah. And that's so good to hear where there any things that any anything that helped you with your involvement in the programme? So you've mentioned there, you've good family support. And did that help you engage better? I'm just thinking, did they walk with you? Did you know?

**Pt4**

Yeah, no, absolutely. The family support health. Again, I, I'm a I'm a fan of this programme, I really do see how it can benefit patients and the health service both, so I was keen for it to succeed. I am keen for it to succeed in this sort of wider context, SB, so I was quite highly motivated to begin with. And also, I guess, you know, the diagnosis itself and the fact that I had to undergo surgery, that's a bit of a motivator in and of itself.

**SB**

Absolutely. Absolutely. And in terms of that, the fact that Laura came out to you, and to show you some of those exercises, Pt4 is quite unique, given the COVID sort of backdrop and those other coordinators that weren't able to do that. And do you think that that helped the fact that she was there in person?

**Pt4**

Yes, I do. Yes, I do. And in actual fact, I would say the personalities of the staff involved, all of them. And I would include the hospital and clinical and staff and in the Royal as well. Just shockingly nice people all very very good at their job, but great interpersonal skills really friendly, really affable, very upbeat. Just, you know, I personally am quite optimistic and upbeat anyway, but I can see that being if you weren't and you were having a bad day, just being in their company would would give you a boost. You know, I can't speak highly enough of everybody who was involved in that programme.

**SB**

And just asking, I just want to ask about the information pack that you received? How did you find that? Or what were your thoughts on that?

**Pt4**

Well, a lot of it was to do with the cancer. And then the risks involved in the surgery, the follow up, all that sort of stuff. And frankly, that was all very scary. But the the pilot programme? Yeah, it, you know, I read through it and that that's probably what made me philosophically commit even further, because I really could see and believe that this is a good thing. And the whole programme is worth embarking on so positively, I guess is the answer.

**SB**

Okay. And you have mentioned already, I'm just going to ask you about, what do you see what do you perceive as a benefit from you being involved? Now, looking back, you have mentioned there that you've stopped smoking and stayed off them, that you have also given up alcohol. Is there other benefits you think that you feel have come a result of being part of the programme?

**Pt4**

I think it actually did make me fitter. The fact that you get into a routine, now because I'm back at work, I can't obviously go seven days a week. But I would still do that hike, maybe three or four days a week now and I think I would always have walked a dog, but I don't think I'd have gone quite so far or made it quite so difficult. So I think there's probably a benefit from that as well.

**SB**

It sounds like there's a benefit for your dog as well.

**Pt4**

Yeah lol

**SB**

Put a smile on his face, too. Are there any areas that you see could be improved for the programme? thinking about this is hopefully going to be rolled out across Northern Ireland, and maybe other tumour groups as well? Is there anything that you would like to say that, you know, we could think about or we could look at changing or doing better? Because we're really keen to hear that at recruitment stage or also during the programme's life. So just anything you can think of that we could do

**Pt4**

To be honest, Sharon, not really. I mean, I was, as I said, very impressed with the people their attitude, their just general demeanour. And I think the information I was provided was was fine. No, no. I thought it was well done. I mean, I'm sure there are areas for improvement, but none that would come to mind to me.

**SB**

Okay. Okay. Well, Pt4, I don't have any more questions for you. Is there anything that you want to say that I haven't asked you about that you were kind of thinking before you come on the call?

**Pt4**

Well, you know, once again, to express my gratitude to everybody involved in all aspects of this, you know, the guy who cleaned the wards in the hospital, he was great craic, you know, from the consultant down and the Macmillan team and [CNS LUNG] and the smoking cessation team, and the exercises. No, I just just I think they're, I think it's a great concept. I think it's something that will benefit patients and the health service both. I thought it was well managed, well run, and just just say thank you, I guess is about it really.

**SB**

Yeah, absolutely. And that will be fed back into the in terms of the anonymized, obviously, but just in terms of, they will, they will see the outcome of this research. So that that's, that's thank you for your comments. And but yeah, that's the end of the interview. I'm just going to stop the recording here first.

# Patient 5

Pt5

**SB**

Okay, that's it recording now, so we're all good to go. So, first of all Pt5, when you were first offered the cancer prehabilitation programme, what was your understanding of the benefits of the programme?

**Pt5**

Well, Lesley explained that it was a method of ensuring a quicker recovery after the operation.

**SB**

Yeah, yeah, absolutely. And were there any other benefits that she explained?

**Pt5**

Well, obviously she said that in general, the more exercise you do the fitter you will become in the long term. But the most of the the conversation revolved around my upcoming operation.

**SB**

Okay. And at that stage, whenever you were offered the programme, what was your understanding of how the programme would be delivered?

**Pt5**

Well, she said, you know, being in she, she gave me booklets, which gives the the various stages of the, the programme, and she also mentioned that about the drinks, and how often they would have to be taken before the the the operation. That was it as far as I can remember, it was a year ago, you know? My mind *inaudible*

**SB**

So the girl, I'm not sure who your move more coordinator was, whether it was Laura, maybe she came out to you and she, did you know that she would be coming to visit you in person? Because this would have been during COVID time so did you know that she was going to be coming in person? Or did you expect it to be virtually or what was your thinking?

**Pt5**

I was quite happy that she was gonna come out personally, to be perfectly honest, the COVID thing didn't really cross my mind I think we'd been over the hill at that time. I was quite happy for it to, for her to come into the house. And she did all the social distancing and the mask thing and all the rest of it. So no, no, the whole thing was very relaxed.

**SB**

Fab That's good, what were the main reasons and you said there about getting you fit or for surgery? Were there any other reasons that you agreed to take part?

**Pt5**

I think two or three years ago, I took part in a Queen's University, what was it, SIPPS thing, the exercises and the exercising, that they went through with me about balance about this that and the other, I've kind of carried those on. So I know that your systems worked out of Queens as well. So I quite happy to take part in another not survey but to continue the programme. Should we say

**SB**

Okay, okay. And did you have any concerns about taking part?

**Pt5**

Absolutely, no, no

**SB**

No, that's great. And in terms of, you didn't feel overwhelmed at all about too many appointments, or anything like that?

**Pt5**

No not at all, no. I'm very sanguine about the whole thing, I'm a very pragmatic sort of person. And, you know, the, I think a lot of people say, oh, my god, cancer and all the rest of it So I just said, get this thing out of me. Sooner I can recover and get back on my feet the better.

**SB**

Absolutely. Yeah. Did anyone in your clinical team or your own home support network encourage you to take part?

**Pt5**

No, no, not at all. It was just the opportunity arose and I took it

**SB**

Was your wife supportive?

**Pt5**

Oh, absolutely. Oh my god, she's been through cancer three times. She's made of iron girders my wife

**SB**

Gosh, gosh. so then can you tell me a wee bit about what parts of the programme you completed

**Pt5**

All of it really I did the exercise the stretching the legs up the legs I just followed through the brochure. Most days. Some days I didn't but most days and then at the end, the pre op drinks you know, managed to get all those down me so yeah. I'm a reasonably disciplined self-disciplined sort of person. I'm ex RAF, so you have it beaten into you from 15 years of age as an apprentice so it's no big deal following following instructions.

**SB**

Okay. So you completed there you were took part in the exercise element. There was other elements and you talk there about nutrition in terms of your pre-op drinks. What about emotional support? Was there emotional support provided

**Pt5**

It was there if I needed it, you know. But as I said I'm a very pragmatic sort of person, you know, you know, the poo stuff happens and that's the way I looked at these things. So let's just get on with them. Get us sorted, you know, and get on with the rest of your life.

**SB**

And were you offered… I don't I don't know anything about your background Pt5, but we offered anything in terms of smoking cessation, or any sort of around alcohol? These maybe aren't issues for you. So I'm just asking generally

**Pt5**

The only problem with alcohol is I don't get enough *laughs* You're asked how much you drink, and of course you lie about that. Very fond of a glass of wine. I haven't smoked for 22 years.

**SB**

So that's not an issue for you

**Pt5**

So that wasn't an issue at the time, but I suppose I think I limited myself to about it wouldn't even have been half a glass of wine for about a month or so before the the operation. And you know it all worked out ok.

**SB**

Okay, good. Good. And in terms of the programme from what was introduced to you, to you taking part, did it meet your expectations? Or were there parts that didn't

**Pt5**

Bearing in mind that it's a new experience to me so I couldn't say that I had any preconceptions about it. You know, the lassie that came out, she explained what it was all about and I was quite happy to go along with it.

**SB**

Okay. Okay. And were there any barriers that you experienced to engaging in the programme?

**Pt5**

No not at all

**SB**

Okay, that's great. And was there anything that you think particularly helped you? And I think one thing that's coming through that I'm hearing from you is that you're quite a disciplined and motivated person. And that probably helped.

**Pt5**

I didn't need anybody standing behind me saying do this, do that, because obviously, I realised it was for my own benefit. So I just got on with it.

**SB**

Yeah, yeah. And you mentioned at the start about the information pack, did you find that helpful, or?

**Pt5**

Oh yes it was it was very interesting, actually.

**SB**

And what in terms of can you remember back? What did you particularly find interesting?

**Pt5**

It was, it was the concept that this is something that I’d never heard of before that, the better you look after yourself before the operation, the quicker you're going to recover from it. I found that fascinating. It's logic, when you think of it now but at the time, it just never occurred to me.

**SB**

Yeah, yeah. And in terms of what benefits did you experience from participating in the programme, did you feel that you recovered well after your operation

**Pt5**

Oh very quickly. I mean the day I came out of hospital. The day after I came out of the hospital I came out on the Monday, my daughter came over from Edinburgh to because I'm also my wife's carer. And she came over to kind of help out. And she took me for a walk, I live in a place called Ashbridge Court and to get out of it, there's quite a hill to walk up. On day one, I was able to walk up the hill with her. It was exhausting. But you know, after that, and then day two we walked a bit further, day three and so on and so forth. So yeah, I think it was very, very beneficial. I had a reasonable core fitness but I think the, your programme definitely helped me.

**SB**

Okay. Okay. And Pt5, are there area... any areas for improvement? We are constantly wanting to learn and make things better for future patients. And this is quite this was quite new for us. So is there any areas that you think we could do better in relation to that cancer prehab programme?

**Pt5**

Not really. As I said the whole thing we have obviously it was my first experience.... I think perhaps you might want to do is get a panel of volunteers. I mean, some I took it on the chin. Other people don't. Perhaps it might be an idea if someone is really uncomfortable with the whole idea is to speak to someone who's been through it.

**SB**

Okay, yes.

**Pt5**

It might be too difficult you know confidentiality and all that sort of crap. But it might be helpful to other people to speak to someone who's been through it.

**SB**

Yes. And that does form part of other, like preoperatively patients would have opportunities in other areas to do that. So that's not not impossible. So that's a great, thank you for that suggestion, that could be woven into a programme like this. So yeah,

**Pt5**

I mean I would be very happy to talk to someone. And that's another thing as well. A lot of people internalise god this is an Americanism but they internalise the whole thing. Whereas my my wife said, look, go tell everybody tell as many people as you can. So that it gets it out of your system by talking about.

**SB**

Absolutely

**Pt5**

Therefore, I'd be very happy to talk to anybody that's going through that sort of trauma.

**SB**

That's great, Pt5, thank you. And I'll maybe make the CNS Leads in Colorectal know that, so that if that is something they're going to do that they could contact you if that's okay.

**Pt5**

Absolutely. No problem.

**SB**

That's great. Thank you so much. Well, listen Pt5, I don't have any more questions for you. Is there anything I haven't asked that you would like to say?

**Pt5**

No, not at all. The system worked for me and the drinks were quite palatable. You have absolutely no input into the food in the Ulster hospital *laughs*.

**SB**

No I don't sorry

**Pt5**

I'm sure you've heard about it. God, I lost a stone in the week i was in there. It's inedible. And it's one of those stupid things, they won't let you go until you've had a bowel movement. You're never ever going to have a bowel movement if you're not eating anything. So it's a kind of a catch 22. But apart from that, no the whole system worked for me from the camera up the bum to put it crudely, to being on the table was six weeks, in which it just blew me away. The whole system it was like a production line this, and then that and then bang, bang, bang, bang, and then all of a sudden, you're on the table. I feel it's absolutely incredible. Incredible.

**SB**

And did you do you feel that that I mean that sounds very process oriented? Did you feel that you were acknowledged in that process? And that you're do you know that you weren't treated as a number going through but that you felt that you were you got the appropriate care going through?

**Pt5**

Well, the only problem I had was, we arranged for my daughter to come across paid air fares for her and all the rest of it because someone in the system, and I have to be upfront with you here I did put in a massive complaint about it, told me that the operation was going to be a week before it actually took place. So as I said, I'm my wife's carer. My daughter was coming across to look after Rosie while I was in hospital. Everything was ready, and then she by 10 minutes after I got everything arranged paid all this money out, the hospital come back and said Oh, sorry. We made a mistake. You're actually in next week. I was absolutely I was furious and was also you get yourself kind of emotionally ready, right? Tomorrow morning or next week I'm going through this. And then some bloody civil servant says it's not my fault. It's the system's fault. I was absolutely crushed. But apart from that the the system was brilliant. This this woman I'm nearly sure I know her name, up in the Ulster hospital she needs to be taken aside and told people are not numbers. They are people.

**SB**

Yeah. Yeah.

**Pt5**

I'm sorry. I'm beating your ear here about it but it's frustrating.

**SB**

All right. And well, we hope that in terms of the cancer prehabilitation programme that you did feel that...

**Pt5**

Yes that was perfect, absolutely perfect

**SB**

Yeah, that's that's good. That's good. Listen Pt5, thank you so much for your time today. I'm just gonna stop.

**Pt5**

Sorry for running off there at the end, but whenever I get that particular bee in my bonnet I get really, really, really angry.

**SB**

It’s ok. I'm just going to stop the recording here.

# Patient6

Pt6

**SB**

That's it on now, that's super. So Pt6, first of all, whenever you were first offered the cancer prehabilitation programme, what was your understanding of the benefits of being part of the programme?

**Pt6**

I'd no real thoughts on it either way, I just know, I just know, I felt that it couldn't do any harm.

**SB**

Okay. Yep. That's, that's, yep. That's sometimes the best way to look at it, isn't it? Yeah. And what was your understanding about how the programme would be delivered?

**Pt6**

Again I wasn't 100% sure, you know, I just took it as it came, you know?

**SB**

Okay. Okay. And would you have had any concerns around that time you know, like, in terms of like, COVID, about meeting up with people in person? Or would you have preferred it to be all done over the telephone? Or did you have any thoughts on that at the time?

**Pt6**

Well at the time, the way I was looking at it, they're nurses, so you'd probably be safer with them that you would with somebody else?

**SB**

Yeah. Yeah. Yeah. And what were the main reasons that you agreed to take part on the programme?

**Pt6**

You're talking about the exercises. And at one stage they were talking about taking a lung out, but I needed to be fit to do it and they didn't think I would be

**SB**

Okay. Okay. So it was kind of it was like, a motivation to get the surgery then.

**Pt6**

Yes

**SB**

Okay. And what other parts of the programme then were you involved in? There was obviously the the exercise element of it? And did you meet up with ... now I don't know anything about you Pt6 at all? was there a smoking cessation conversation? And, or alcohol conversation? I I'm just sort of inquiring because I'm enquiring the same with everybody, but

**Pt6**

It was mostly exercise, but I did have the smoking from the Ulster hospital.

**SB**

Okay.

**Pt6**

Again it was on the phone. I remember speaking to them

**SB**

Okay. Okay. And did anybody offer you emotional support? As you know, through your move more coordinator or?

**Pt6**

No

**SB**

No. And was there any nutritional advice offered?

**Pt6**

Not really, no,

**SB**

No. Okay. Everybody gets a slightly different version. So that's what I'm just I'm just checking. Okay. And so it was mainly exercise, and then your somebody talked to you about smoking? Was did anybody talk to you about alcohol?

**Pt6**

No, no.

**SB**

Okay. And okay, so did you have any concerns about taking part?

**Pt6**

No, not at all

**SB**

No, and you said there just about, you know, that it because it was led by healthcare professionals, it was sort of like you trusted that?

**Pt6**

I was quite happy with it yeah

**SB**

Yeah. Okay. And did anyone in your clinical team or your support network at home? Did they encourage you to take part or was it just yourself do you think?

**Pt6**

Well, a bit of both

**SB**

Okay. Okay. And in terms of your clinical team, what was? How did they encourage you to take part?

**Pt6**

It was just it's going to do you the world of good, it's going to help you it's going to help with the surgery if you need it

**SB**

Yeah. Yeah, yeah. And did you have somebody at home helping you? Supporting you?

**Pt6**

Yeah.

**SB**

And what was their views on the programme? Did they think it was a good idea?

**Pt6**

Yes.

**SB**

Very good. Very good. And that that's, that's great that you had that support? And what did you think about the programme then after having done it? Did you think it met your expectations of you know what you were told at the start?

**Pt6**

Yes, it did. made a hell of a difference.

**SB**

It did. did. And was there anything that didn't meet your expectations?

**Pt6**

No, not at all

**SB**

Not at all. Okay. And what do you think, what do you think helped you benefit the most Pt6?

**Pt6**

Well, it was it was the exercises that the nurse was doing with me.

**SB**

Okay.

**Pt6**

You know all in all it seemed dead simple

**SB**

Yeah

**Pt6**

You can get help whenever you want. There's just so simple, but the difference it was unbelievable.

**SB**

Really, really, that's really good to hear. And do you so I mean, that in terms of you … my next sort of question was going to be about what helped you participate in not having to go out of the house. Was that … did that help that the exercises were easy to do at home?

**Pt6**

Yeah, but it helped me get out of the house too

**SB**

Good good, right. And what did you do in terms of exercise?

**Pt6**

Well, they were all pretty simple exercises. There was maybe about 30 of them, you know, and Eimer was the nurse, she was giving me sort of a five a week to do sort of you know in my own time, to take it easy and if I couldn't do them don't do them. You know, that sort of thing?

**SB**

Yeah.

**Pt6**

Changing, and they were changing every week. So it was different part of your body

**SB**

Oh right. Very good. And did you have a wee bit of time between your diagnosis and your surgery was there a wee bit of a few weeks there was there?

**Pt6**

Yes, it was about six weeks. They ended up not doing the surgery but it was nothing to do with my fitness because he actually said, my fitness was fine to do surgery.

**SB**

Oh, good. Right. That's right. And then did you have other treatment Pt6?

**Pt6**

No.

**SB**

Instead of … you didn't, okay.

**Pt6**

Not yet.

**SB**

Not yet. Okay. How long ago were you did you get your diagnosis?

**Pt6**

About eight weeks ago

**SB**

Oh, you're very alright, you’re very early on.

**Pt6**

I only had my planning day there on Tuesday to see what we're going to do

**SB**

Awk right okay. All right. Okay. Apologies then sorry, a lot of the people I've been speaking to had their diagnosis a year ago. So you're the you're the shortest length of time since diagnosis. Okay. And so, yes. So in terms of perceived benefits of the prehabilitation programme, one thing that's come across very clearly is that you feel that you the exercise is really helped. And you feel if you needed surgery, you're you're fit for it.

**Pt6**

Yeah, yeah.

**SB**

Yeah. And is there any another benefit there you said about encouraging you to go out of the house? And was there any other benefits that you saw from the programme?

**Pt6**

Oh, probably mentally. Made me feel better.

**SB**

Okay. Okay. And how or in what way does….

**Pt6**

You know, I was thinking poor me and then I realised I have to get myself together because there's other people round you

**SB**

Yeah. Yeah. And that sense of having other people there to support you, that that helped.

**Pt6**

Yeah.

**SB**

Yeah. Okay. Um, and that you would have received an information pack about the cancer prehabilitation programme do remember that? Did you look through it? Was it helpful?

**Pt6**

Yeah it was helpful. Me and my wife both went through it

**SB**

Oh, good, good. And what parts of it did you think were more helpful? Or what did you look at more?

**Pt6**

Well we just read it from start to finish.

**SB**

Okay.

**Pt6**

And we found the whole thing helpful.

**SB**

Okay. Yeah.

**Pt6**

Even maybe wee things that were obvious. It was just all reading it, you know looking at it

**SB**

Yep, no, that's, that's good. And did you do you think it informed you better about what was to come?

**Pt6**

Yeah, definitely yeah. Yeah.

**SB**

Okay. Okay. So your it's all very much in your head at the minute, some of the other people I've been talking to, it's they're having to think back about the project and maybe don't really see any areas of improvement because it was maybe a while back. But now that you're you've just you've been in the programme, can you see things that we could do better? Are there any areas for improvement?

**Pt6**

Because I've only went through the exercises I mean, I can find no fault at all with it whatsoever.

**SB**

Okay. That's good. But if there if there is anything in terms of how you were introduced to the programme, was it [CNS LUNG] introduced you to it?

**Pt6**

Yes it was.

**SB**

Yes, [CNS LUNG]. Well, in terms of what information she provided you with at that time, is there any way she could say was there anything more that you would have liked to know about the programme at that point?

**Pt6**

No, not really. No.

**SB**

Okay. Okay.

**Pt6**

I think she covered it well, yeah.

**SB**

Okay. And did you like that you were able to see the move more coordinator I see. I find that word hard to say coordinator to say that right. Was it good to see her in person?

**Pt6**

Well, I actually only met [CNS LUNG] the once and that was the day I was diagnosed. But I've spoken to her on the phone.

**SB**

Yeah. And then do you have a move more coordinator that would give you the exercises?

**Pt6**

Yes.

**SB**

Yes. And you've seen her in person.

**Pt6**

She comes every week.

**SB**

Brilliant. And do you like that she comes out rather than ... because some people during COVID would have had that maybe over zoom or over the phone or something?

**Pt6**

No, no, I'm quite happy for her to come out.

**SB**

Yeah, yeah. That's That's great. And in terms of the smoking conversation, did you feel that was a benefit?

**Pt6**

Yes, well, they put me on Nicorette.

**SB**

Oh Okay,

**Pt6**

Now because, I was just speaking to a girl there, she was telling me, you know, I was explaining to her it was more having something in my hand, so she gave me one of those wee inhalers. I can't say I've stopped but I've cut down so much I really really have and I'm hoping to stop in the near future.

**SB**

Okay, great. Yeah, very good. Very good. And do you know like, or do you have the ability then to go back to the smoking cessation nurse to have that more support?

**Pt6**

Yes. Yeah.

**SB**

Yeah. That's ongoing is it?

**Pt6**

Yeah, that's great.

**SB**

Yeah, that's that's brilliant. Pt6 I don't have any more questions as such for you. But is there anything I haven't asked that you would like to say about the programme?

**Pt6**

No, no. I like everyone who comes, especially the girl that comes out and does the exercises. Unbelievable. For them to be so simple and to help so much?

**SB**

Yeah. That's really great. I'm really, really pleased that you had a really positive experience and that you see the benefit. That’s brilliant. Yeah. Good to hear. Listen Pt6 I'm just going to stop the recording.

**Pt6**

Right

# Patient 7

**SB**

That's us now, perfect. Okay. So whenever you were first told about the cancer prehabilitation programme, what was your understanding of the benefits that being part of the programme would bring?

**Pt7**

It was... Yeah, I think it was primarily my head about making sure you know, you're as fit as possible for operations and treatment? You know, in terms of getting you physically ready for it as best as possible. So yeah, that was probably the main thing I thought.

**SB**

Okay. Okay. And what was your understanding of how the programme would be delivered?

**Pt7**

Yeah, I knew it would be a sort of a bit of, you know, regular contact plus advice around exercises?

**SB**

And did you know it was going to be on the telephone and virtual?

**Pt7**

Yeah, thanks to COVID. Yeah,

**SB**

Yeah. So and, and how did you feel about that?

**Pt7**

I was fine. Because, you know, I would have sort of regularly exercised and stuff, you know, it wasn't like I was starting from scratch or wondering, you know, but obviously, you know, there could have been some sort of regular thing that would just, you know, that would just help. Because it's not a good, it's not a good time to be all excited about, you know, jumping out of bed and doing exercises every day, you know, see if you've got the bad news, and you probably need some, some, you know, chivvying up to, to get you going. But I knew that wasn't possible with COVID, so I was fine.

**SB**

You would see that potentially as an advantage for the programme going forward if people were able to meet in person with other people.

**Pt7**

Yeah, I mean, even even the likes of myself, you know? Not, you know that I'm familiar with doing exercises most days. Yeah, you're, you're maybe not in a good headspace at that time. So, yeah. It's good to get some encouragement.

**SB**

Yeah. Yeah. And what would you have said, were your main reasons for taking part and you mentioned there, like your understanding was to get as fit as possible. Did that influence your your reason to take part then?

**Pt7**

No, I was keen. I think the whole thing is, is such a good idea. Yeah, I mean, when they asked, when they asked me to take part like it was a no brainer, because I just think it's people talk about post post rehabilitation and trying to get back to you know, yeah. Getting ready for treatment and surgery just sounds like a no brainer. Yeah, happy to take part because of that.

**SB**

Yeah. And what elements of the programme, the programme is tailored to the patient and the tumour group etc, I'm sure you're aware of that but what parts then the programme, would you have participated in obviously, there was exercises there, what other parts were offered to you?

**Pt7**

Was there other parts?

**SB**

Well, there, so you would have had contact with your move more coordinator, they might have provided some emotional support if that was part of their referral, and no, nope. Okay.

**Pt7**

No, it was the it was the physical side of things

**SB**

Nutritional advice? Were you referred to dietetics at all?

**Pt7**

No, I don't remember that now no.

**SB**

So just the exercise really beforehand. And any any discussion about smoking cessation, or alcohol or anything like that?

**Pt7**

No, no, but it wasn't an issue for me. So, maybe that's why it wasn't mentioned.

**SB**

Yep, no, that's fine. So just exercise. No, that's grand. And so did you have any concerns about taking part?

**Pt7**

No, no.

**SB**

And it didn't feel at all it was going to be like, in terms of just happened the diagnosis and additional appointments. That wasn't a concern at all.

**Pt7**

No, I mean, we had you know, there's the Colorectal Nurse as well, who, you know, was available. So I could talk to her about anything, medical. And then there was the Oncologist not so no, I mean, I wasn't expecting anything else.

**SB**

But that so you didn't feel overwhelmed at the addition of that of the programme.

**Pt7**

Oh, no, no, no, not at all.

**SB**

Okay. Did anyone in your clinical team or your support network encouraged you to take part? Or was it just you …. were you were fine just making that decision yourself?

**Pt7**

Yeah, I was fine. Just yeah. It just was a no brainer for me.

**SB**

Okay. And did the programme meet your expectations? In terms of the exercise then?

**Pt7**

Yeah, the exercises were, you know, they were sort of geared. So, I thought there were, I thought there were really good, appropriate ones. So it wouldn't have necessarily been, you know, I mean, the fact that I would have done some exercise, I'd probably just like, go out running or something. You know, so it was good to have much more specific exercises that were relevant. So that that was good,

**SB**

Good. Um, was there anything in terms of the programme that didn't meet your expectations?

**Pt7**

No, as I say you know, some person to person stuff would have been good, or, you know, in a small group or something, but I guess that just wasn't, wasn't feasible. So

**SB**

Yeah, yeah. And hopefully it is for future participants.

**Pt7**

Yeah.

**SB**

Absolutely. It definitely … you were at a unique time in terms of the whole the whole COVID.

**Pt7**

Yeah.

**SB**

Were there any issues, anything that helped you with your involvement in the programme? And then on the flip side, was there anything that's hindered you? So we're just looking at sort of barriers facilitators there?

**Pt7**

Well, no, I think I've forgotten their name now, the move more coordinator, but yes, she was very, very positive, enthusiastic, you know, so it was. Yeah, I can't think of anything you know. Other than it just sounded like a brilliant idea. I didn't, and I can't think of anything that would have blocked me or got in my way.

**SB**

Did you find what did you think about the information pack that you would have been given?

**Pt7**

Yeah, yeah. It was good. Yeah. I think, you know, all in I felt from everybody who was yeah, loads of information, which was great.

**SB**

Okay. Yeah. Yeah. And what have you seen as your, the benefits from being part of the programme?

**Pt7**

Well, I mean, although I was, you know, used to doing exercise, obviously, for the months before, you know, and I was... I really hadn't got the energy, you know. For me, it was like, I'd been losing blood, and, you know, I was low iron and all that. So, you know, just being able to focus on you know, because it couldn't go out for a run. So, you couldn't do that sort of nonsense, but the exercises that she gave me were, were ideal. So, the benefit to me ultimately, I thought, then I bound to have been a quicker recovery after afterwards, after the operation, for a start. And then obviously, I kept up so so I was in touch again after that, and she gave me some bit more specific because obviously now I've got a I've got a wound and all to deal with. So I'm able to do something specific prior starting to chemo, so it's bound to have helped with recovery.

**SB**

And in terms of having that person then as well to support you, like as an as an additional person to the normal sort of like, journey and through cancer treatment, emm did you feel it that was helpful even? I know they didn't offer emotional support, per se. But yeah, was that a support to you that added connection?

**Pt7**

Yeah, definitely. I mean, you know, just that, try to keep, you know, I thought move, move more as a great title for it, you know, and just to keep moving. That's so important, really, when you probably, you know, to be honest, you don't feel like it, you just want to curl up in a ball somewhere. And so, yeah, you know, it was I mean, important. Yeah. So for me important part of the overall support and part of the clinical team, I suppose.

**SB**

Yeah. And going forward for us Pt7, with the likes of this cancer prehabilitation programme, and hopefully, in the future, it'll receive more funding and, and maybe be widened out for other tumour groups as well. Can you think of any areas for improvement for it, anything you would like to have seen that wasn't there? In terms of even how you were introduced to the programme? Or what you were offered through it?

**Pt7**

Yeah, I think it's hard to think. Would I have? I mean, yeah, maybe it's, you know, you think of these things, like, peloton or something where you're, you're online and doing exercises with other people at the same time. But the chances of that meeting, are, you know, being useful to maybe a lot of people who wouldn't be into into using zoom and so on, but I don't know. Yeah, maybe just just more, I mean like again, back to regular contact, where I mean some of the exercises you know you wonder, am I doing this right, you know, because it's only it's only sketches, and you think you know, am I doing this right? Or is this should I twist further, or what, you know, and all that, but so good to have had some group sessions where you could just get into these things. That's the only thing I can think of.

**SB**

Yeah. Okay. So if if it had to continue virtually for for any reason, in the future, that there was some way to get feedback on how you were doing?

**Pt7**

Yeah.

**SB**

And what you were doing. Okay, that's, that's useful. And, yeah, and I think one thing that you've highlighted throughout was just that if there was a way to have more contact with people and I know at the minute things are changing, and people will be able to. Yeah, it's about how to make it more social, as well. Yeah.

**Pt7**

But yeah, I mean, even even, like, my mom's in her 80s, and she's whacking away at, you know, online everything. So she's, she's got no problem with all that sort of thing. She did a video consultation with somebody in the hospital there a while back, and it was, she was more than happy. You know, it was great. Because she couldn't be bothered going in trying to get parked and all *laughs* and she was delighted. So, you know, maybe something like that. Were were you could, you could have an interactive session of just making sure you're, you know, you're you're working through your exercises, something like that.

**SB**

Yeah, no, that's, that's useful. Thank you for that feedback. I don't have any more questions for you. Do you have anything you want to say that I haven't asked about before I stopped the recording?

**Pt7**

Yeah. No, no, I mean, I suppose, you know, maybe I'm not your best subject for this because I would just be totally positive about it. So I can't really, you know, that's maybe not giving you any anything to build on.

**SB**

If it's working, then that's great. Absolutely. And, and it's good to hear that it's been a positive experience. So no, that's, that's great and valid. Absolutely.

**Pt7**

That's all I can really say it's just, you know, 100% it's something that just makes so much sense to do. I think it'd be brilliant if it was extended and expanded, for sure.

**SB**

Thank you.

# Patient 8

**SB**

Oh, there it is now that's super. Okay, so, Pt8, I want to just for you to think back to whenever you first heard about the cancer prehabilitation programme. What was your understanding? Whenever [CNS HNC] met you that day and introduces what did you understand where the benefits of taking part on the programme?

**Pt8**

I, I wasn't sure. I wasn't sure if it was any benefit to me. But I agreed to be filmed, interviewed and to let people see exactly the progress of whatever was happening to me. And I thought, if I don't get anything out of this, perhaps it will be beneficial to others

**SB**

Okay.

**Pt8**

That's why I agreed to do the film and do the interviews. And, yes, I talking to my, my daughter in law, who, unfortunately has terminal cancer and has been living with us for a few years now.

**SB**

Oh, oh dear

**Pt8**

She said it was nice, that I was able to talk to these people have the support and know exactly what was ahead and support I could have whatever that she didn't have. She didn't have that facility.

**SB**

Okay. Okay. So did your daughter in law did her saying that to you? Did that sort of influence maybe your decision a little?

**Pt8**

No, no, not at all. As soon as I was approached, I had no idea whether I would survive my cancer or not a bit tearful, I said, anything I could do, to try and give something back to the team, I would be prepared to do that.

**SB**

That's very, very generous. Whenever [CNS HNC] initially introduced this to you, then [Pt8], what was your understanding of how the programme would be delivered. So I think, just from prior to me starting the recording there, you'd mentioned you had met with [CNS HNC], you've met with the dietician.

**Pt8**

And I just can't remember all the names. But yes.

**SB**

And did you meet with the speech and language therapy as well?

**Pt8**

Yes, I did. You see, I forgot to mention that as well. Speaking to that, that team as well, yes.

**SB**

And just wondering as well about any emotional?

**Pt8**

I can't, I am remembering now about being told to do exercises, exercises with things to position my neck and head and different things that they suggested that would be helpful to me during the whole process.

**SB**

Okay, so then you did ….

**Pt8**

I think sorry, I think that was so I think that was for the not physiotherapy but that type of trying to, because once I once I've lost a piece of my tongue and different things they were trying to just trying to explain things that may help me get through this.

**SB**

And did you have any concerns about taking part in the programme?

**Pt8**

No, no, not at all. I went to secondary school, I wasn't in the lower classes, I did do my O levels and did okay, but I decided to start work rather than continued education, but I'm not academic. I'm not scared to answer the questions but maybe I am not answering the questions in the way you would like

**SB**

Oh, no, not at all. You're answering, your answers are really like, yeah, really, really good. Pt8, I suppose what I'm asking there in terms of if you had a concern, sometimes people after they've had a diagnosis of cancer, and then maybe they're introduced to this programme. They might feel Gosh, I've already got a lot of appointments already. This might be too much.

**Pt8**

No, no that never entered my mind because it's my upbringing, but if I say I'll do a thing I'll do it wholeheartedly. I would no, I would just do it. If I said I would do it, there's no way I was ever going to back out from from doing it. I'm trying to do it as honestly, as I tell you answer the questions as honestly as I can.

**SB**

Yeah. And Pt8 did you feel supported by your clinical team in going forward with the programme?

**Pt8**

Very much so. Yes. [CNS HNC] and [CNS HNC] Yes, they told me any concerns at any time. And also I must add that the staff, nurses and the team doctors at the city hospital when I was having my radiotherapy I just can't remember the doctor's name, but he said anything at all. [Pt8], just ring me if you have any concerns, please don't feel that you cannot get in touch with us and ask for advice or whatever. The team? Absolutely first class. Yes.

**SB**

So good to hear that you've had a really good positive experience.

**Pt8**

I just can't remember, I mentioned the two girls in the Ulster. I'm so annoyed I can't not, even the dietician at the City was lovely. they've all been so so nice. Yeah.

**SB**

Yeah. That's, that is so good. So Pt8, you mentioned there that you were given some exercises to do I'm imagining maybe by the speech and language therapist, I think they might give exercises to do

**SB**

Yes I think it was I had to lie on my back and try and get my neck. I just forget what I had to do now. But it was I'm not sure if that was to help with the swallowing to build up muscles.

**SB**

Were you able to do them? And did you do them?

**Pt8**

I being honest again, I didn't do them probably as routinely as I should have. They have not been doing my exercises, as I should be doing for my lymphoedema. I just get into my head that because I'm active I am arms, driving and doing all the things I did before I had this I stopped because I'm so active but you know something I think maybe I should be doing more to try and get rid of my lymphoedema the swelling inside my mouth or whatever.

**SB**

Did is sort of going into that programme. I know you it sounds like you didn't have much ex went in the way of expectation of the programme. But do you think the programme met what you thought it might do? Or do you think it wasn't quite good enough? Or what were your thoughts?

**Pt8**

This is where it gets difficult now for me to answer. yes, it was definitely worthwhile. And as I say the people in it were just, you know, so helpful. Did I get much out of it. Probably yes. I knowing that there was someone someone that was just a phone call away, I didn't I didn't partake of that. But then my my surgery, my surgery I went in on a Thursday and was out on a Monday. Everything just went so well. It's maybe maybe you know, if things have not gone maybe so well. I would maybe have been depending more on I know they call it prehabilitation but honestly, as soon as I come out of hospital I I just was driving within a couple of days. The surgeon phoned me and I was like no I wasn't driving that day, but he just couldn't believe that I was doing so well, like three or four days after my surgery.

**SB**

That's fabulous.

**Pt8**

Yes, I have they are delighted with my my progress. I saw my I saw my surgeon [CONSULTANT] last Wednesday. And he is going to see me again. He wants me to go and have a scan, it's a routine scan that they do but delighted and said I can have my holiday if that's what I want. I have to more or less get go on and enjoy your life. Yeah. It's been it's been really, really very positive what they have done? You know?

**SB**

Yeah, yeah. That is so good. And, Pt8, were there anything? Was there anything that kind of prevented you engaging with the programme or sort of acted as a bit of a barrier or anything that helped you?

**Pt8**

Sorry. No, I, I know I've done anything that was asked of me, I have had meetings and radio interviews. I think I've participated as honestly, I've tried to help as much as possible.

**SB**

But I was just, I was just sort of asking there Pt8, was there anything that made it difficult for you to maybe like in terms of like getting to the appointments, prior to surgery? Or did that go okay, was that? Was that easy enough to attend that clinic to see the team?

**Pt8**

No, everything everything was... No, there was nothing too much trouble, okay. either for the staff or for me, as I said, there, once they agreed to take part in this programme, I no, nothing was any trouble to me

**SB**

That's good.

**Pt8**

I have agreed I have agreed to any interviews any filming that they want to do. There's videos, even the surgeon took a video of the surgery, but I wouldn't be seeing that one because it was too gory *laughs*

**SB**

Sometimes better not to see that *laughs*. You had mentioned there in terms of benefits about maybe helping you cope better with your treatment and just being prepared and for treatment. I'm just wondering, as well did you notice any sort of differences or improvements in terms of like maybe around your movement of your neck? Or I'm wondering as well, did you I don't know, I don't have any of your medical history or anything Pt8, but I'm just wondering, did you have any engagement?

**Pt8**

Maybe not so much from the team. But [CONSULTANT] told me the first time he met me, it was only a couple of weeks after the surgery, how to try and manipulate the wound and try and break down the scar tissue and all this type of thing you know, but that was from the surgeon, although the [CNS HNC] was in attendance when when he was saying and she I must say there's something I have omitted here. They asked me about counselling and [CNS HNC] on more than one occasion. And she saw that in one or two of the interviews that I just didn't seem as ... it's not depression but because they'll say everything went well. The trouble I'm having is eating. That is my biggest problem and after talking to [CONSULTANT] on Wednesday because he not only did the surgery, he had to remove two of my molars and I had one removed by my dentist on the other side of my mouth around six months before my cancer diagnosis. So I'm left with no real back chewing teeth and eating has been difficult. And that is the thing that gets me down and [CNS HNC] had suggested counselling but I'm not sure I think it's just because I was a bit down. I'm still I'm gonna say everything's gone everything has gone great for me medically but eating is, is an issue, like I was 16 and a half stone and I've dropped to 13. I am I am holding my weight but it is... I have to just try and force myself to. I was a great eater before now I just eat to keep my weight up. I've lost the joy the experience of it all.

**SB**

Have you been able to? You've mentioned that whenever you were introduced to the likes of the dietician and the speech and language therapist who would have supported you at the beginning and they said that you could you know, lift the phone Have you been able to go back to them?

**Pt8**

No, but you know something, maybe. But the girls there again did contact me all the time. But they I was taking supplements the Ensures and that type of thing. Whenever I was losing they were saying that don't be giving up on the supplement drinks because you have to really keep your energy levels up. I must say yes. The dietitians were on me all the time because they were concerned about maybe losing too much weight too quickly. They were definitely on with me on a regular basis after my radiotherapy

**SB**

That was good good good that they've kept you know communication with you.

**Pt8**

They have they have since that about maybe two months ago because I told them that I had got to a weight and to be honest, I'm five foot a wee bit less than five foot nine and my ideal weight should be 11 and a half / 12 stone, well I'm 13, but all the clothes I had when I was 16 and a half stone are lying in the wardrobe they're no good to me *laughs*

**SB**

Pt8 did you engage with and I don't know your background as such as I was saying but in terms of smoking cessation or anybody in terms of alcohol use, did anybody speak to you by either those things and there maybe neither are relevant possibly but I'm just checking

**Pt8**

I hope I Yes, I enjoyed a drink before the surgery. I that was one of the questions I asked [CONSULTANT] and he said because I read about my diagnosis and it said that it could have been down to abuse of alcohol or sunshine I have enjoyed both things. This cancer I have is a type of melanoma. Yes, he warns me that I have to protect myself in the sun but he said on Wednesday past go and have your holiday just protect yourself but use your factor 50 and all this, but the alcohol thing. Yes, I enjoyed a drink at the weekends. I didn't drink every day but I I enjoy drink Friday, Saturday and Sunday I'm a member of the golf club down at Kirkiston and having having lost my wife when she was 53 I was on my own for eight years before I met my partner and yes I probably found that I oh I had some great friends. One of them since deceased, took me out to Spain to his holiday home. Yes, I enjoyed a drink

**SB**

I'm just checking

**Pt8**

The guys when I was on my own.

**SB**

Did anybody did the anybody speak to you about reducing alcohol as part of the cancer prehabilitation?

**Pt8**

Yes [CONSULTANT] says everything in moderation. Yes, no, I I I didn't have a drink all through my treatment you couldn't with the radiotherapy. One of the doctors up there said I wouldn't even enjoy it, it wouldn't test like anything with the radiotherapy but I am enjoying a pint of Guinness again, as the radiotherapy it will be a year since I finished my treatment on the 21st of June. So I'm about 10 and a half months after my radiotherapy, so, no, I have an odd pint at the weekend and a glass of wine. I don't think I'm abusing it.

**SB**

No, no, I just I was just wanting to check if that that's spoken to you as part of that, you know, the programme there. My last question Pt8 just is, if you've any suggestions for us for any areas for improvement in the cancer prehabilitation programme. So we hopefully will be rolling this out to other trusts other health and social care trusts in the future. And we just want to know, is there anything you have identified that we could do better so that we could make sure we do it better in the next in the next areas?

**Pt8**

Honestly, I think as I say as I say, I didn't maybe I didn't ring the girls because I felt that things were going so well. It would have been nice to be able to say that it was great just to lift the phone have reassurance from [CNS HNC] and [CNS HNC], but I just because things went so well after my surgery I, I maybe didn't, didn't participate enough. I just, you know, perhaps got on with it rather than,

**SB**

No everybody has a different approach so they do, so it's what works for you. And what about the information pack that you would have received was that helpful?

**Pt8**

Yes, yes it's over a year ago. Yeah, it's hard to remember that, yes, everything was in it, like what they were going to help me with you know, with talking to dieticians talking to the Physiotherapists you know

**SB**

Yeah, yeah. No, that was just it. Just wanted to check if that yeah, if there was anything there that would have prompted you to think about improvements but that is good to hear that the information was there.

**Pt8**

No I think, I honestly, think that what you are doing is really worthwhile. People knowing and any way it can help them get get through this. As I said, I lost my wife to cancer when she was 53. My daughter in law is living with this terminal cancer. Cancer is no stranger to me. But maybe someone hasn't had the experience of cancer and is really frightened. This, just getting information, knowing that there's so much help yes, is a positive thing.

**SB**

Yeah, yeah. And that's so good. So good to hear. Pt8, I am just going to stop the recording here if that's okay.

**Pt8**

Okay.

# Patient 9

**SB**

Oh, there it is now. Okay. Right. So whenever you were first introduced to the cancer prehabilitation programme Pt9, what was your understanding of it?

**Pt9**

My understanding was that if I did the exercises it would strengthen my core and therefore help me through the operation, which it did.

**SB**

brilliant, brilliant, and at that time, whenever you were first offered the programme, how did you think it was going to be delivered, like, what in what way? Did you think it was going to be over the telephone? Did you think somebody was going to come out? Was it? Was it before COVID or after COVID that you were diagnosed?

**Pt9**

It was before COVID

**SB**

Before COVID. Right. So that question is probably more relevant then to somebody who's diagnosed during COVID, actually.

**Pt9**

Right.

**SB**

So did you understand then that you would have been seen in person?

**Pt9**

Yeah

**SB**

That's okay. And what influenced your decision to take part in the programme?

**Pt9**

Just anything I could do to help towards my recovery I would do

**SB**

Yeah, yep. And did you have any concerns about taking part?

**Pt9**

No

**SB**

No, you weren't worried about any additional appointments?

**Pt9**

No

**SB**

Or that it was difficult enough to cope and this was something more there wasn't none of that sort of concern for you?

**Pt9**

No

**SB**

Great, great.

**Pt9**

My husband's very good, he's always here at my beck and call he takes me wherever I need to go.

**SB**

Brilliant. So that would have helped you then

**Pt9**

Yeah

**SB**

Yep. Interesting. Very good. And did anybody, you mentioned your husband there, but did anybody your husband or your clinical team, did they encourage you to take part in the programme?

**Pt9**

Yes, the girl that was speaking to me about it

**SB**

Yeah

**Pt9**

She said that's a good idea, because it would strengthen your core and therefore help you through the operation and help your recovery

**SB**

And what did your husband think about it?

**Pt9**

Yes, he encouraged me to do it. He helped me put me through my paces.

**SB**

*laughs* keep you on your toes. Brilliant. Brilliant. And from what was outlined about the programme at the start did it the the programme then that you received that that meets your expectations?

**Pt9**

Yes. Yes I thought it was great that sort of thing was in operation.

**SB**

Yeah. And what what parts of the programme did you complete? And the reason I'm asking you that is everybody does something slightly different depending on their diagnosis and what they need. So I can tell you what the different elements are and you can tell me which parts then apply to you. So there's the exercise, but there is some some people will get nutritional advice. Some people will get emotional support. Some people will have somebody speak to them about smoking cessation, some people about alcohol, reducing alcohol intake. And I'm trying to think that for some other patients, then outside of your tumour group, there's a few other things offered like speech and language, but that wouldn't apply to you. But which, which parts of the programme did

**Pt9**

The exercise, mostly the exercise.

**SB**

Okay, and were there any other parts? Like the emotional support?

**Pt9**

No, I didn't really need that.

**SB**

Okay. Okay. And was there any part of the programme that didn't meet your expectations?

**Pt9**

To be quite truthful, I never knew really that I had cancer if you know what I mean?

**SB**

Okay.

**Pt9**

I knew there was something there, but I didn't realise it was actually cancer…..

**SB**

Yeah. And was the programme then…..

**Pt9**

..... until I had had the operation.

**SB**

Oh, really? Until you had the operation?

**Pt9**

Yes. And then they told me that what they had removed was cancerous.

**SB**

Right. Okay. So you were offered this prehabilitation programme prior to surgery, but you weren't sure at that stage it was definitely cancer.

**Pt9**

No

**SB**

Yeah, right. Okay. Yep. And and you said there earlier about your husband taking you to your appointments, so that helped. Were there anything else? Was there anything else that helped you take part in the programme?

**Pt9**

Oh, just I always had his support, whatever I was doing for all my scans and cameras, and all that. I had never been in hospital apart from my stroke that time. All my life. That was the first time I was in.

**SB**

You did well.

**Pt9**

Apart from having my first baby, and then, after that, I was round about three different hospitals getting the different scans done. I had the camera down my throat and the camera up the bottom end.

**SB**

Yeah.

**Pt9**

In different hospitals.

**SB**

Yeah. Was there anything else that helped in terms of like, I'm just thinking about the move more coordinator who would have been doing the exercises with you? Was there anything about that that sort of helped you participate that made you want to participate? Or?

**Pt9**

So what made me want to participate was that I knew it would do me good, and prepare me for the operation.

**SB**

And was there anything that made it more difficult for you to participate?

**Pt9**

Just the fact of having to go and have them cameras down and up

**SB**

Yeah.

**Pt9**

That was very frightening.

**SB**

I'm sure. I'm sure.

**Pt9**

You know that and not being used to hospitals really.

**SB**

Yeah, yeah. What benefits do you think you had as a result of being part of the cancer prehabilitation programme?

**Pt9**

I think it's helped me to recover quicker.

**SB**

Okay.

**Pt9**

And it's helped me through the operation.

**SB**

Yeah. Yeah.

**Pt9**

Everybody, no matter what hospital I was in, everybody was so nice to me

**SB**

Which just makes it so much easier, doesn't it?

**Pt9**

Yeah.

**SB**

And....

**Pt9**

They explained as they went along what was happening and what it was for, like I was really naive.

**SB**

Yeah, yeah. But in terms of the exercise element, which would be part of the prehabilitation programme, as opposed to, you know, the surgical input, the prehabilitation, you felt helped you got more ready and helped you recover better?

**Pt9**

Definitely, it sort of strengthened my core

**SB**

And Pt9, are there any areas for improvement in the cancer prehabilitation programme? So thinking about you were offered that at whenever you were first sort of introduced that you might have to have surgery. And so you were given information about the programme, you might have been given a pack to take away with you. And the move more coordinator, then would have been in contact with you and then met with you about exercises? Is there any part of that, that you feel that we could have done better and thinking I'm asking you the question, because we want to do this with this will be going on in the future and we want to, we want to make improvements where we can. So is there any suggestions for improvements?

**Pt9**

No, I found it all very good. Very helpful. I couldn't think of anything more you could have done for me.

**SB**

Okay. Okay. No, that's, that's great, but yeah, that's fine. And thank you for your feedback. Just we are we're keen to learn and you know, make improvements where we can and it's good that you've had a positive experience. Yeah, that's okay Pt9, I don't have any more questions for you. Is there anything you want to say that I haven't asked you?

**Pt9**

No, no.

**SB**

Okay, right. Well, I'm gonna stop the recording then.
